# Comparison of Preprint Postings of Randomized Clinical Trials on COVID-19 and Corresponding Published Journal Articles: A Systematic Review

**DOI:** 10.1001/jamanetworkopen.2022.53301

**Published:** 2023-01-27

**Authors:** Anthony D. Bai, Yunbo Jiang, David L. Nguyen, Carson K. L. Lo, Isabella Stefanova, Kevin Guo, Frank Wang, Cindy Zhang, Kyle Sayeau, Akhil Garg, Mark Loeb

**Affiliations:** 1 Division of Infectious Diseases, Department of Medicine, Queen’s University, Kingston, Ontario, Canada; 2 Faculty of Health Sciences, Queen’s University, Kingston, Ontario, Canada; 3 Division of Infectious Diseases, Department of Medicine, University of Toronto, Toronto, Ontario, Canada; 4 Department of Medicine, McMaster University, Hamilton, Ontario, Canada; 5 Faculty of Health Sciences, McMaster University, Hamilton, Ontario, Canada; 6 Mental Health and Addictions Care Program, Kingston Health Sciences Centre, Kingston, Ontario, Canada; 7 Department of Medicine, Queen’s University, Kingston, Ontario, Canada; 8 Division of Infectious Diseases, Department of Medicine, McMaster University, Hamilton, Ontario, Canada; 9 Division of Medical Microbiology, Department of Pathology and Molecular Medicine, McMaster University, Hamilton, Ontario, Canada

## Abstract

**Importance:**

Randomized clinical trials (RCTs) on COVID-19 are increasingly being posted as preprints before publication in a scientific, peer-reviewed journal.

**Objective:**

To assess time to journal publication for COVID-19 RCT preprints and to compare differences between pairs of preprints and corresponding journal articles.

**Evidence Review:**

This systematic review used a meta-epidemiologic approach to conduct a literature search using the World Health Organization COVID-19 database and Embase to identify preprints published between January 1 and December 31, 2021. This review included RCTs with human participants and research questions regarding the treatment or prevention of COVID-19. For each preprint, a literature search was done to locate the corresponding journal article. Two independent reviewers read the full text, extracted data, and assessed risk of bias using the Cochrane Risk of Bias 2 tool. Time to publication was analyzed using a Cox proportional hazards regression model. Differences between preprint and journal article pairs in terms of outcomes, analyses, results, or conclusions were described. Statistical analysis was performed on October 17, 2022.

**Findings:**

This study included 152 preprints. As of October 1, 2022, 119 of 152 preprints (78.3%) had been published in journals. The median time to publication was 186 days (range, 17-407 days). In a multivariable model, larger sample size and low risk of bias were associated with journal publication. With a sample size of less than 200 as the reference, sample sizes of 201 to 1000 and greater than 1000 had hazard ratios (HRs) of 1.23 (95% CI, 0.80-1.91) and 2.19 (95% CI, 1.36-3.53) for publication, respectively. With high risk of bias as the reference, medium-risk articles with some concerns for bias had an HR of 1.77 (95% CI, 1.02-3.09); those with a low risk of bias had an HR of 3.01 (95% CI, 1.71-5.30). Of the 119 published preprints, there were differences in terms of outcomes, analyses, results, or conclusions in 65 studies (54.6%). The main conclusion in the preprint contradicted the conclusion in the journal article for 2 studies (1.7%).

**Conclusions and Relevance:**

These findings suggest that there is a substantial time lag from preprint posting to journal publication. Preprints with smaller sample sizes and high risk of bias were less likely to be published. Finally, although differences in terms of outcomes, analyses, results, or conclusions were observed for preprint and journal article pairs in most studies, the main conclusion remained consistent for the majority of studies.

## Key Points

**Question:**

How do preprints of randomized clinical trials (RCTs) on COVID-19 differ from their corresponding published journal articles?

**Findings:**

In this systematic review of 152 COVID-19 RCT preprints posted in 2021, 119 (78%) were subsequently published in a scientific, peer-reviewed journal. When preprint and journal article pairs were compared, there were differences in terms of outcomes, analyses, results, or conclusions in 65 of 119 studies (55%); however, the main conclusion remained consistent for all but 2 studies (2%).

**Meaning:**

These findings suggest that although there were differences in the outcomes, analyses, results, or conclusions between RCT preprint and journal article pairs in most cases, the main conclusion remained consistent for the majority of studies.

## Teaser

This systematic review compares differences in time to publication, outcomes, analyses, results, or conclusions of randomized clinical trials on COVID-19 posted as preprints in 2021 and corresponding published journal articles.

## Introduction

COVID-19 is an infectious disease caused by SARS-CoV-2.^1^ The World Health Organization (WHO) declared the COVID-19 outbreak a global pandemic on March 11, 2020.^2^ In response, researchers have focused their efforts on COVID-19 worldwide, leading to an unprecedented number of published studies.

Of the different study designs, randomized clinical trials (RCTs) provide the highest-quality evidence to guide disease management.^3^ There is an impetus to make COVID-19 RCT findings publicly available as soon as possible so that they can be applied in clinical settings. Publishing an article in a scientific journal is a lengthy process of peer review, revisions, and resubmissions. In contrast, preprints can make study findings publicly available before publication in peer-reviewed journals, which facilitates dissemination and hastens implementation in clinical practice.^4^ For example, the preprint of the Randomised Evaluation of COVID-19 Therapy (RECOVERY) trial on the benefits of dexamethasone led to implementation of this agent as the standard of care for COVID-19 even before the official journal publication.^5^ However, preprints may not meet the required standards for scientific publication without peer review, they may be subject to change because the manuscript is not yet finalized, and they may contain errors or false information.^6^ As a result, preprints can mislead the public.^6^

The availability of preprints provides an opportunity to assess for different reporting biases that may occur during the time from manuscript completion to journal publication.^7^ First, publication bias may occur,^7^ where some preprints may never be published in a journal. Second, time-lag bias may occur,^7^ where preprints with negative results take longer to be published. Third, outcome reporting bias may occur,^7^ where the outcomes, subgroups, or methods of analysis are inconsistent between the preprint and published journal article.

To date, few studies have assessed differences in COVID-19 RCT preprint and corresponding journal article pairs. Prior research included mainly descriptive studies of a small number of preprints^4,8,9^ and did not focus on a detailed comparison between preprints and journal articles for a large sample of COVID-19 RCTs. We performed a systematic review with a meta-epidemiologic approach to answer the following research question: In RCTs on interventions to prevent or treat COVID-19 that were published as preprints first, how did the preprints and corresponding published journal articles differ in terms of time to publication, outcomes, analyses, results, or conclusions? We hypothesized that there would be a substantial time lag from preprint posting to journal publication and that there would be notable differences between preprints and corresponding published journal articles.

## Methods

The study protocol was registered on the Open Science Framework.^10^ This was a meta-epidemiologic study that adopted a systematic review to describe and analyze reporting of RCTs in preprints and journal articles. The unit of analysis was a study and the outcome was the reporting of methods and results; therefore, ethics approval and informed consent were not applicable. We followed the Preferred Reporting Items for Systematic Reviews and Meta-analyses (PRISMA) reporting guideline modified for the purpose of meta-epidemiologic studies.^11^

### Search Strategy

A literature search was conducted using the WHO COVID-19 database^12^ and Embase for preprints posted between January 1 and December 31, 2021. In the WHO COVID-19 database, we searched for medRxiv preprints related to COVID-19 posted during 2021. In Embase, we used medical subject headings (MeSH) and non-MeSH terms related to COVID-19 and RCTs and restricted the search to preprints (eAppendix in Supplement 1). There was no language restriction for either search. The WHO database and Embase were used instead of medRxiv because they allowed more sophisticated combinations of different Boolean operators and themes. In addition, the Embase search included preprint servers other than medRxiv, such as bioRxiv.

### Study Eligibility Criteria, Screening, and Selection

Preprints were included in this study if they described COVID-19 RCTs with human participants and research questions regarding the treatment or prevention of COVID-19. Research questions could focus on preventative measures such as vaccinations to prevent COVID-19 or therapeutic interventions to treat patients diagnosed with COVID-19.

Studies that did not report primary data, such as commentaries, reviews, and secondary analyses, were excluded. Phase 1, pilot, and feasibility studies were also excluded because their objectives differ substantially from other RCTs. Finally, studies with only children were excluded because trials for this population have different challenges and implications. For example, COVID-19 is milder in children, so the appropriate sample size, primary outcome, and clinical significance may differ from trials in adults. Two reviewers (among A.D.B., Y.J., D.L.N., I.S., K.G., and A.G.) independently screened each title and abstract on Covidence.

### Retrieval of Corresponding Published Journal Articles

For each included preprint, we searched for the corresponding published journal article if it existed. In addition to the website link to the corresponding journal article publication on the preprint server, we searched the WHO database, MEDLINE, Embase, and the Cochrane Database of Systematic Reviews using the study title, keywords, and author names in the preprint, with the end of follow-up on October 1, 2022.

### Data Extraction

After screening for relevant studies, 2 reviewers (among A.D.B., Y.J., D.L.N., C.K.L.L., I.S., K.G., F.W., C.Z., or A.G.) independently read the full text of both the preprint and published journal article if it existed. They then extracted the data in duplicate onto a standardized form. Reviewers resolved disagreements by discussion to reach consensus and, if necessary, by adjudication by a third reviewer.

The data extraction sheet included the following for both the preprint and corresponding journal article: author names, online publication date, journal, journal impact factor, funding, study location, research question, sample size, primary outcomes, secondary outcomes, primary analysis for the primary outcome, secondary analyses for the primary outcome, subgroup analyses, results, and author conclusions.

Journal impact factors from the 2021 Journal Citation Reports^13^ were used. Journal impact factor was dichotomized into journals with an impact factor below 50 and journals with an impact factor of 50 or above. Study location was based on where patients were recruited for the trial. Primary analysis was defined as the main analysis for the primary outcome. In contrast, secondary analysis for the primary outcome included all statistical methods used to analyze the primary outcome, including sensitivity analyses. The RCT was defined as a positive trial with notable results if the intervention was shown to be significantly beneficial in the treatment or prevention of COVID-19 (ie, statistical significance based on *P* < .05 and/or 95% CIs excluding no benefit). Conversely, the RCT was defined as a negative trial if the intervention was not shown to be beneficial (eg, did not reach statistical significance).

### Risk of Bias Assessment

Two reviewers (among A.D.B., Y.J., D.L.N., C.K.L.L., I.S., K.G., F.W., C.Z., or A.G.) independently assessed risk of bias using the Cochrane Risk of Bias 2 (RoB 2) tool,^14^ with respect to the primary outcome in the trial. The RoB 2 tool was used to rate each study as having a low risk of bias, some concerns (hereafter described as medium risk with some concerns for bias), or a high risk of bias for each domain and overall.^14^

### Outcomes

The primary outcome was the time from preprint posting to journal publication. Time was calculated based on the online publication of the first version of the preprint to the online publication date of the corresponding journal article.

Secondary outcomes were the substantial differences in study characteristics between the preprint and published journal articles. The study characteristics were as follows: (1) primary outcome, including change to another outcome as the primary outcome altogether or change in how the primary outcome was defined (eg, time point or event criteria); (2) secondary outcomes, including the addition of new secondary outcomes or the deletion of secondary outcomes previously reported in the preprint; (3) primary analysis for the primary outcome, including change in the statistical analysis method or principle (eg, intention to treat or per protocol); (4) secondary analyses for the primary outcome, which was the addition or deletion of a sensitivity analysis or the change in any of the statistical methods used to analyze the primary outcome; (5) subgroups analyzed, including the addition or deletion of a subgroup analysis as well as a change in how a subgroup was defined; (6) sample size, where the overall sample size across all intervention groups for the study was not exactly the same between the preprint and journal article; (7) results in terms of primary outcome, in which the number of events, sample size, effect estimate, CI, or *P* value was not the same in the preprint and in the journal article that was not attributed to rounding; and (8) the study conclusion, in which the authors concluded that there was a notable benefit of the intervention based on the results in the preprint but not in the journal article, or vice versa.

For cases in which results for the primary outcome differed between the preprint and journal article, we compared the effect estimates if the primary outcome was reported in terms of either odds ratios (ORs) or relative risks (RRs) in both the preprint and journal article. We converted all ORs and RRs such that values less than 1 signified benefit and those greater than 1 signified harm. We then calculated a ratio of OR or RR. For example, the ratio of OR was calculated as the OR reported in the journal article divided by the OR reported in the preprint.

### Statistical Analysis

For the descriptive analysis, medians (IQRs) were used for continuous variables, whereas counts and frequencies were used for categorical variables.

The primary outcome of time from preprint posting to journal publication was assessed using a Kaplan-Meier survival analysis, in which the event was publication in a scientific journal. A Cox proportional hazards regression model was used to identify study characteristics associated with publication in a scientific journal. Potentially associated factors were selected a priori and included the country where the study was conducted, whether the first author lived in an English-speaking country, funding, research question, sample size, positive or negative trial, and overall risk of bias.

In the subset of preprints with corresponding published journal articles, descriptive statistics were used for the secondary outcomes of differences in study characteristics between the preprints and corresponding published journal articles. A Fisher exact test was used to compare preprints published in journals with a higher impact factor vs those published in journals with a lower impact factor.

All tests were 2-sided, with significance at *P* < .05. All analyses were performed using R, version 4.1.2 (R Foundation for Statistical Computing). Statistical analysis was performed on October 17, 2022.

## Results

**Figure 1.**
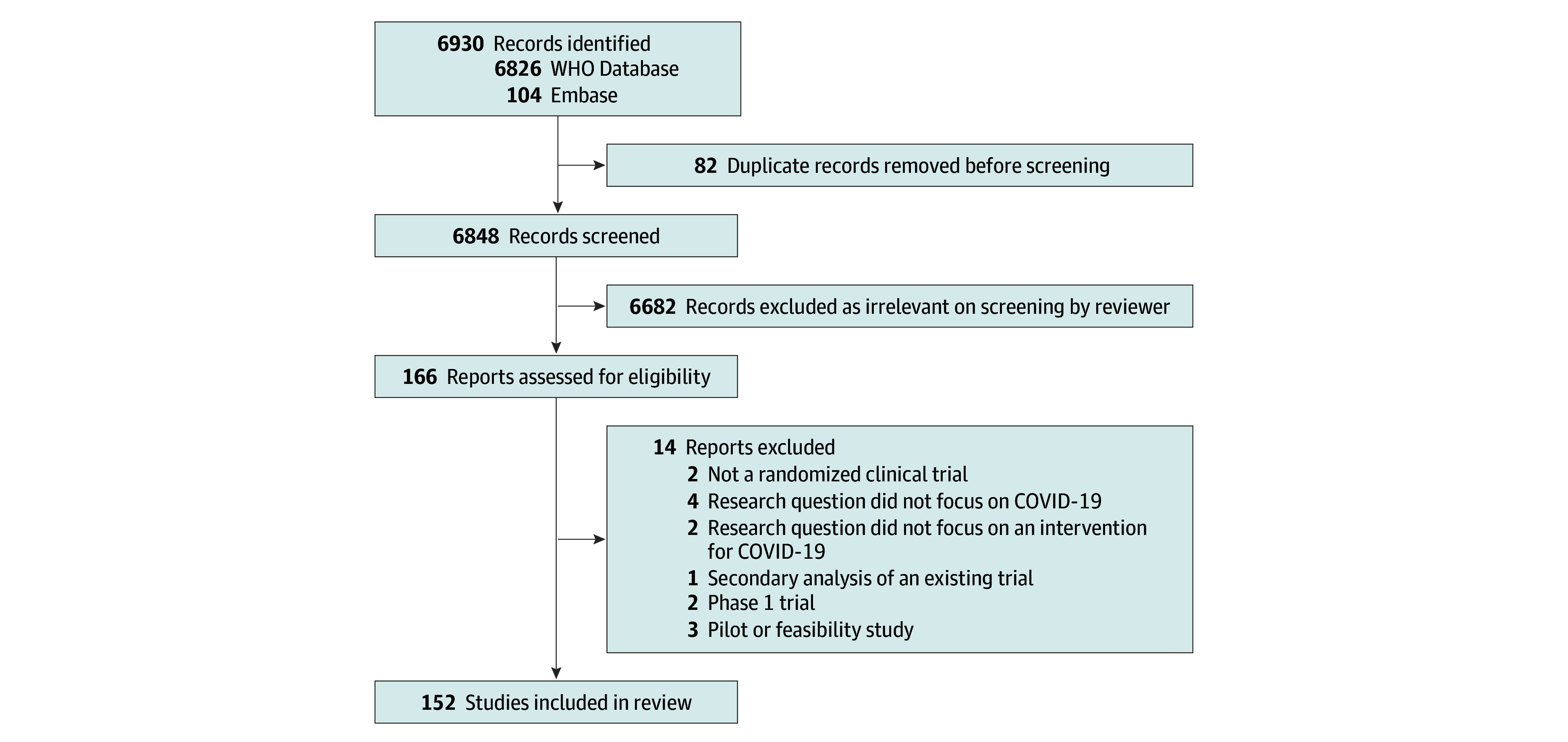
Study Flow Diagram WHO indicates World Health Organization.

The literature search yielded 6930 records (Figure 1). A total of 82 duplicates were removed, resulting in 6848 unique records that were screened. After abstract screening and full-text reading, 152 RCTs were included in the analysis. All 152 RCTs were posted on the medRxiv server.^15,16,17,18,19,20,21,22,23,24,25,26,27,28,29,30,31,32,33,34,35,36,37,38,39,40,41,42,43,44,45,46,47,48,49,50,51,52,53,54,55,56,57,58,59,60,61,62,63,64,65,66,67,68,69,70,71,72,73,74,75,76,77,78,79,80,81,82,83,84,85,86,87,88,89,90,91,92,93,94,95,96,97,98,99,100,101,102,103,104,105,106,107,108,109,110,111,112,113,114,115,116,117,118,119,120,121,122,123,124,125,126,127,128,129,130,131,132,133,134,135,136,137,138,139,140,141,142,143,144,145,146,147,148,149,150,151,152,153,154,155,156,157,158,159,160,161,162,163,164,165,166^

### Study Characteristics

The study characteristics are described in Table 1. Details for each study are described in eTable 1 in Supplement 1. Overall, 34 preprints (22.4%) were international trials that were conducted in more than 1 country. The most common research question was related to therapeutics (105 preprints [69.1%]) followed by vaccines (32 preprints [21.1%]).

**Table 1.** Study Characteristics of Preprints

Characteristic	Preprints, No. (%) (N = 152)
Trial type	
Individual randomized	150 (98.7)
Cluster randomized	2 (1.3)
Country in which the trial was conducted	
International (>1 country)	34 (22.4)
US	23 (15.1)
India	14 (9.2)
United Kingdom	13 (8.6)
Brazil	8 (5.3)
Mexico	8 (5.3)
China	8 (5.3)
First author lives in an English-speaking country	66 (43.4)
Pharmaceutical industry funding	57 (37.5)
Research question	
Therapeutics	105 (69.1)
Vaccines	32 (21.1)
Other^a^	15 (9.9)
Trial sample size	
Small (≤200)	63 (41.5)
Medium (201-1000)	54 (35.5)
Large (>1000)	35 (23.0)
Positive trial^b^	97 (63.8)

^a^ Other studies included 1 study on diagnostic testing followed by isolation and 14 studies on infection prevention or control.

^b^ Indicates the intervention was shown to be significantly beneficial in the treatment or prevention of COVID-19 (ie, statistical significance based on *P* < .05 and/or 95% CIs excluding no benefit).

The median sample size was 272.5 (IQR, 109.0-810.5). Sample size was categorized as small (≤200) for 63 preprints (41.5%), medium (201-1000) for 54 preprints (35.5%), and large (>1000) for 35 preprints (23.0%).

Of the 152 preprints, 2 (1.3%) were later withdrawn by the authors due to data analysis errors that led to incorrect interpretation of results. Furthermore, 2 preprints (1.3%) are being investigated for methodologic concerns and medical ethics violations.^167^

### Risk of Bias Assessment

The risk of bias assessment for individual studies is described in eTable 2 in Supplement 1. The overall risk of bias assessment was judged to be low in 58 preprints (38.2%), medium with some concerns for bias in 63 preprints (41.5%), and high in 31 preprints (20.4%).

### Time to Publication

**Figure 2.**
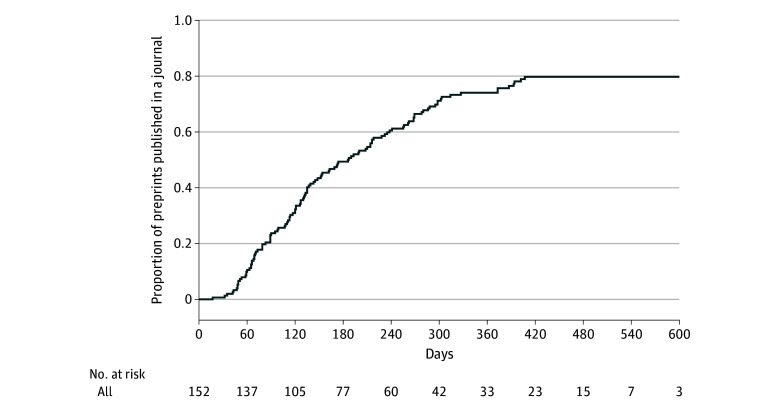
Time From Preprint Posting to Journal Article Publication

As of October 1, 2022, 119 preprints (78.3%) had been published in a scientific, peer-reviewed journal (Figure 2 and eTable 3 in Supplement 1).^168,169,170,171,172,173,174,175,176,177,178,179,180,181,182,183,184,185,186,187,188,189,190,191,192,193,194,195,196,197,198,199,200,201,202,203,204,205,206,207,208,209,210,211,212,213,214,215,216,217,218,219,220,221,222,223,224,225,226,227,228,229,230,231,232,233,234,235,236,237,238,239,240,241,242,243,244,245,246,247,248,249,250,251,252,253,254,255,256,257,258,259,260,261,262,263,264,265,266,267,268,269,270,271,272,273,274,275,276,277,278,279,280,281,282,283,284,285^ Two preprints^66,166^ were published in a single journal publication.^237^ The median time to publication was 186 days (range, 17-407 days). For preprints that were not published, the median follow-up time was 471 days (IQR, 402-524 days). Results of the univariate Cox proportional hazards regression models of time to publication are described in Table 2. Time to publication was similar for preprints posted during the first quarter (January 1 to March 31), second quarter (April 1 to June 30), third quarter (July 1 to September 30), and fourth quarter (October 1 to December 31) (eFigure in Supplement 1).

**Table 2.** Univariate Cox Proportional Hazards Regression Model for Publication in a Journal

Variable	HR (95% CI)	*P* value
International trial (>1 country)	1.70 (1.13-2.58)	.01
First author lives in an English-speaking country	1.61 (1.12-2.31)	.01
Pharmaceutical industry funding	1.20 (0.83-1.73)	.34
Research question		
Therapeutics	1 [Reference]	NA
Vaccines	1.52 (0.98-2.36)	.06
Other^a^	1.37 (0.76-2.47)	.29
Trial sample size		
Small (≤200)	1 [Reference]	NA
Medium (201-1000)	1.53 (1.00-2.33)	.05
Large (>1000)	2.67 (1.67-4.25)	<.001
Positive trial^b^	1.13 (0.77-1.64)	.54
Overall risk of bias		
High	1 [Reference]	NA
Medium with some concerns	1.84 (1.06-3.20)	.03
Low	3.42 (1.97-5.93)	<.001

Abbreviations: HR, hazard ratio; NA, not applicable.

^a^ Other studies included 1 study on diagnostic testing followed by isolation and 14 studies on infection prevention or control.

^b^ Indicates the intervention was shown to be significantly beneficial in the treatment or prevention of COVID-19 (ie, statistical significance based on *P* < .05 and/or 95% CIs excluding no benefit).

In the final multivariable model of time to publication, only sample size and overall risk of bias remained significantly associated with publication in a journal. For time to publication with small sample size as the reference, a medium sample size (201-1000) had a hazard ratio (HR) of 1.23 (95% CI, 0.80-1.91; *P* = .35) and a large sample size (>1000) had an HR of 2.19 (95% CI, 1.36-3.53; *P* = .001). For overall risk of bias with high risk of bias as the reference, medium risk with some concerns for bias had an HR of 1.77 (95% CI, 1.02-3.09; *P* = .04) and low risk had an HR of 3.01 (95% CI, 1.71-5.30; *P* < .001).

### Differences Between Preprints and Published Journal Articles

In the comparison of the 119 preprints published in a journal, there were substantial differences between the preprints and published journal articles in 65 cases (54.6%) (Table 3). Of the 8 studies (6.7%) that had different primary outcomes in the preprint and journal article, 5 (62.5%) had a small sample size (<200), 6 (75.0%) were positive trials, and 5 (62.5%) were published in a journal with an impact factor of less than 2.

**Table 3.** Differences Between Preprints and Published Journal Articles

Area of substantial difference	Preprints published, No. (%)			*P* value for low-impact vs high-impact journals
	All (n = 119)	In journals with an impact factor ≥50 (n = 49)	In journals with an impact factor <50 (n = 70)	
Primary outcome	8 (6.7)	3 (6.1)	5 (7.1)	>.99
Secondary outcome	28 (23.5)	13 (26.5)	15 (21.4)	.52
Primary analysis for primary outcome	16 (13.5)	6 (12.2)	10 (14.3)	.79
Secondary analyses for primary outcome	26 (21.9)	14 (28.6)	12 (17.1)	.18
Subgroups analyzed	20 (16.8)	16 (32.7)	4 (5.7)	<.001
Sample size	7 (5.9)	5 (10.2)	2 (2.9)	.12
Results in terms of primary outcome	33 (27.7)	16 (32.7)	17 (24.3)	.41
Study conclusion	2 (1.7)	0 (0)	2 (2.9)	.51
None (same in all of the above aspects)	54 (45.4)	15 (30.6)	39 (55.7)	.009

For the 7 studies (5.9%) with differences in sample size, the sample size in the journal article was larger than the preprint for 5 studies by ratios ranging from 1.01 to 1.21. For the other 2 studies, the sample size in the journal article decreased by ratios of 0.71 and 0.90. Importantly, results in terms of the primary outcome were different in 33 cases (27.7%). Of these 33 studies, the difference was due to a change in primary outcome for 4 (12.1%), a change in statistical analysis methods for 17 (51.5%), a change in total sample size for 4 (12.1%), and for unclear reasons for 8 (24.2%). For studies with differences in results that reported ORs, the median ratio of OR was 1.01 (IQR, 1.00-1.04). For studies with differences in results that reported RRs, the median ratio of RR was 1.00 (IQR, 0.99-1.00).

In 2 studies (1.7%), the differences in results changed the study conclusion. In one, the preprint^64^ reported a negative trial, whereas the published journal article^210^ reported a positive trial after a change in the statistical analysis method. In the other, the preprint^85^ reported a positive trial, but then the authors withdrew the preprint due to errors in the statistical analysis. The published journal article^223^ for this study reported a negative trial.

Differences for each pair of preprints and published journal articles are described in eTable 4 in Supplement 1. As a sensitivity study, differences between preprints and published journal articles for each quartile of journal impact factor are described in eTable 5 in Supplement 1.

## Discussion

In this meta-epidemiologic systematic review of 152 preprints of COVID-19 RCTs, there was a substantial time lag from preprint to publication, with a median time to publication of 186 days. Preprints of studies that had a small sample size or high risk of bias were less likely to be published in a journal. For preprints that were published as journal articles, there were substantial differences in terms of outcomes, analysis, sample size, and results for the majority of studies. However, the main conclusion remained consistent in the majority of studies. These observations reflect, in part, journal peer-review processes, which contribute to the substantial time lag and the selective process that prohibits studies at high risk of bias from being published. Requests for manuscript revision during the peer-review process likely account for some of the differences found between preprints and published journal articles.

To our knowledge, this is the largest comparison of COVID-19 RCT preprint and published journal article pairs conducted to date. We found that sample size and risk of bias were associated with publication. In a recent retrospective review of COVID-19 preprints and journal articles, Zeraatkar et al^286^ did not find sample size or risk of bias to be associated with publication. The difference was likely due to a difference in power. In the prior review, the authors found journal articles for only 42.3% of preprints and included 74 pairs of preprints and journal articles.^286^ In contrast, we found journal articles for 78.3% of preprints and included 119 pairs of preprints and journal articles. This difference may be attributed to the more exhaustive search of various databases for journal articles and much longer follow-up period in our study. As a result, our analysis of time to publication had more events and greater power to predict estimators of journal publication. One notable difference between our study and that of Zeraatkar et al^286^ was the risk of bias assessment. We used the original RoB 2 tool, whereas Zeraatkar et al^286^ used a revised RoB 2 tool. In the revised tool, the overall risk of bias is assessed to be either high or low. Based on these stricter criteria, almost all of the trials in our study assessed to be at medium risk with some concerns for bias would have been reclassified as high risk. As a result, 38.2% of trials in our study would have had low risk of bias and 61.8% would have had high risk, which is similar to the proportion of low- and high-risk trials reported by Zeraatkar et al^286^ (33.8% and 66.2%, respectively).

In a smaller cross-sectional study of 67 observational and interventional studies that were posted as preprints and published as journal articles, there was a discrepancy in results reporting in 44 cases (65.7%),^9^ which was similar to our estimate of 65 studies (54.6%). In 2 prior studies of both non–COVID-19 and COVID-19 studies of various designs, the main conclusion was contradictory in 0.5% to 2.2% of preprint and journal article pairs,^287,288^ which is similar to our estimate of 1.7%. In contrast to prior studies,^9,287,288^ our study focuses on COVID-19 RCTs. As a study design, RCTs should provide high-quality evidence for knowledge synthesis. Our study included preprints when they were first published online and then followed them until journal article publication or end of follow-up, so our study included preprints that were not published as journal articles. These studies would be omitted in prior studies.^9,287^ As a result, our study can describe time to publication and factors associated with a preprint being published in a journal. In addition, our study included a detailed comparison of each preprint and journal article after 2 independent reviewers performed a thorough reading of the full text and supplementary materials and also conducted a risk of bias assessment, which was not done in prior studies.^9,287,288^ For example, a prior study compared preprint and journal article pairs in terms of the abstract, tables, and figures only.^287^ Therefore, our study adds substantially to the existing evidence.

Our findings have several important implications. First, we observed that there was a substantial time lag from preprint to publication, with a median time to publication of 186 days. As a result, preprints serve their intended purpose of making research findings available to the public much earlier. Second, given the time lag observed and the fact that many preprints may never be published in a journal, knowledge synthesis of the existing evidence should consider including preprints to be comprehensive and current. However, if investigators include preprints, they should use caution in synthesizing and interpreting knowledge and should acknowledge that unpublished preprints are likely to have a higher risk of bias. Third, given the substantial differences observed between preprints and published journal articles, any knowledge translation process that includes preprints should be updated when a preprint is published in a journal. In this study, it was reassuring that the primary outcome estimate and conclusion typically remained consistent in preprints and corresponding journal articles, so these updates likely would not change the overall conclusion.

### Strengths and Limitations

Our study has several strengths. First, the methodology of this meta-epidemiologic study was rigorous. Data extraction and risk assessment were done by 2 independent reviewers. Second, we included a large sample of trials over a 1-year period with a minimum follow-up of 9 months for publication, making our estimates more precise. Third, the analysis of each study was detailed, from reading of the full text and supplementary materials to performing an assessment of risk of bias.

This meta-epidemiologic study also has several limitations. First, our literature search yielded mainly medRxiv preprints. However, medRxiv is currently the most popular and accessed preprint server.^4^ Second, our study findings pertain to only RCTs, which were chosen because they are considered the gold standard for evidence of treatment or prevention. However, RCTs constitute only a small portion of COVID-19 studies and preprints. Our study conclusions cannot be generalized to studies that are not RCTs. Moreover, authors, peer reviewers, journal editors, and readers tend to scrutinize COVID-19 studies and hold them to a higher standard. As a result, our study findings may not be generalized to RCTs not related to COVID-19, which may have different risks of bias and reporting biases.

## Conclusions

This study found substantial time lag between the posting of preprint articles on COVID-19 and their eventual publication in scientific journals. While important differences were found in outcomes, analysis, sample size, and results for most studies, the main conclusions remained consistent in all but a few published articles. Preprints make evidence publicly available much earlier than published journal articles. However, preprints may have a higher risk of bias, and their results may change when they are eventually published in a journal. Therefore, it is important to critically appraise preprints before applying the trial results.

## Notes

Supplement 1. **eAppendix.** Embase Search Strategy **eTable 1.** Preprint Study Characteristics
eReferences 1
**eTable 2.** Risk of Bias Assessment **eTable 3.** Preprints and Corresponding Published Journal Articles **eTable 4.** Differences Between Preprints and Corresponding Journal Articles
eReferences 2
**eTable 5.** Differences Between Preprints and Corresponding Journal Articles for Each Journal Impact Factor Quartile **eFigure.** Time to Publication Based on Date of Preprint Posting

Supplement 2.
Data Sharing Statement
