# Supplementary material for: Comparison of Preprint Postings of Randomized Clinical Trials on COVID-19 and Corresponding Published Journal Articles: A Systematic Review
Source: JAMA Netw Open. 2023 Jan 27;6(1):e2253301. doi: 10.1001/jamanetworkopen.2022.53301 (PMC12543410; doi:10.1001/jamanetworkopen.2022.53301)
Supplement: Supplement 1. — eAppendix. Embase Search Strategy eTable 1. Preprint Study Characteristics eReferences 1 eTable 2. Risk of Bias Assessment eTable 3. Preprints and Corresponding Published Journal Articles eTable 4. Differences Between Preprints and Corresponding Journal Articles eReferences 2 eTable 5. Differences Between Preprints and Corresponding Journal Articles for Each Journal Impact Factor Quartile eFigure. Time to Publication Based on Date of Preprint Posting [file jamanetwopen-e2253301-s001.pdf]

Supplementary Online Content

Bai AD, Jiang Y, Nguyen DL, et al. Comparison of preprint postings of randomized clinical trials on COVID-19 and corresponding published journal articles: a systematic review. *JAMA Netw Open*. 2023;6(1):e2253301. doi:10.1001/jamanetworkopen.2022.53301

eAppendix. Embase Search Strategy

eTable 1. Preprint Study Characteristics

eReferences 1

eTable 2. Risk of Bias Assessment

eTable 3. Preprints and Corresponding Published Journal Articles

eTable 4. Differences Between Preprints and Corresponding Journal Articles

eReferences 2

eTable 5. Differences Between Preprints and Corresponding Journal Articles for Each Journal Impact Factor Quartile

eFigure. Time to Publication Based on Date of Preprint Posting

This supplementary material has been provided by the authors to give readers additional information about their work.

© 2023 Bai AD et al. *JAMA Network Open*.

eAppendix. Embase Search Strategy

Database: Embase Classic+Embase <1947 to 2022 April 11>
Search Strategy:
----
1 (nCoV* or 2019nCoV or 19nCoV or COVID19* or COVID or SARS-COV-2 or SARSCOV-2 or SARS-COV2 or SARSCOV2 or SARS coronavirus 2 or Severe Acute Respiratory Syndrome Coronavirus 2 or Severe Acute Respiratory Syndrome Corona Virus 2).ti,ab,kw,hw,ot. (251621)
2 (coronavirinae/ or betacoronavirus/ or coronavirus infection/) and (epidemic/ or pandemic/) (10843)
3 sars-related coronavirus/ (499)
4 ((coronavirus* or corona virus* or betacoronavirus*) adj3 (pandemic* or epidemic* or outbreak* or crisis)).ti,ab,kw,ot. (13937)
5 1 or 2 or 3 or 4 (255113)
6 random:.tw. or placebo:.mp. or double-blind:.tw. (2069638)
7 5 and 6 (10721)
8 limit 7 to (yr="2021" and "preprint (unpublished, non-peer reviewed)") (104)

****

© 2023 Bai AD et al. *JAMA Network Open*.

eTable 1. Preprint Study Characteristics

First author name [Reference #]	Trial type<br>Setting<br>Time period	Patient population	Intervention (I)<br>Comparison (C)	Main outcome<br>Primary analysis<br>Main result	Conclusion
Abdulamir [1]	Open label<br>2 hospitals in Iraq<br>Jan to April, 2021	COVID-19 infection of all severity confirmed on diagnostic testing	I: Niclosamide PO 4g on first day then 3g/day thereafter on TID regime for 7 days (N=75)<br>C: Standard of care (N=75)	Cure rate i.e. survived<br>Chi-square test of cure rate<br>Cure rate was 72/75 (96%) in both groups (p=1.0)	Niclosamide did not improve survival rate, but decreased time to recovery.
Lima [2]	Double blinded<br>Habitants of city of Fortaleza in Brazil<br>Nov 2021 to July 2021	Mild to moderate COVID-19 infection based on clinical suspicion, some tested negative	I 1: Tenofovir (TDF) 300mg/day PO for 10 days (N=75)<br>I 2: Tenofovir with Emtricitabine (TDF/FTC) 300mg and 200mg/day PO for 10 days (N=74)<br>C: Placebo Vitamin C 500mg/day for 10 days (N=77)	Score of symptoms and signs of COVID-19 infection on day 7<br>Chi-square test for number of symptoms<br>No difference in clinical scores across groups	TDF or TDF/FTC did not change the symptoms score in mild to moderate COVID-19 infection
Hosseinzadeh [3]	Open label<br>Single centre in Iran<br>"During 2020"	Healthcare workers without COVID infection who is at high risk for getting infection	I: 3% dimethyl sulfoxide (DMSO) and 20% ethanol as an intranasal inhalation Q8H for 4 weeks (N=116)<br>C: Routine care (N=116)	COVID infection confirmed on PCR testing<br>Relative risk (RR) and absolute risk reduction (ARR)<br>RR of 0.12 (0.02-0.97) and ARR of 0.06 (0.01-0.11)	DMSO and ethanol nasal spray decreased risk of COVID infection.
Shiri [4]	Double blinded<br>Single centre in Iran<br>April to June 2020	COVID-19 infection requiring admission to hospital	I: herbal supplement of black myrobalan, matic ad sugarcane 3g twice daily by mouth (N=37)<br>C: Placebo (N=35)	Time to significant difference in 4 symptoms<br>Rate ratio<br>RR of 0.285 (0.173-0.427), 0.5 (0.271-0.921), 0.285 (0.169-0.480), 0.333 (0.185-0.598) for cough, fever, dyspnea and myalgia.	Herbal supplement is effectiveness in treating symptoms and reduce hospital length of stay.
Baxter [5]	Open label<br>Single center in US<br>Sept to Dec 2020	Mild COVID-19 infection diagnosed in ER	I 1: Povidone-iodine 10% plus saline rinse twice daily for 14 days (N=37)<br>I 2: sodium bicarbonate plus saline nasal rinse twice daily for 14 days (N=42)<br>C: CDC public data on hospitalization	Hospitalization for COVID-19 infection within 28 days<br>Hospitalization occurred in 0/37 in Povidone-iodine group and 1/42 in sodium bicarbonate group, with ha odds ratio of 0.05 when compare to CDC ata.	Saline nasal irrigation decrease risk of hospitalization when compared to national rate.
Bhatt [6]	Phase 2, open-label<br>12 hospitals in India<br>June 2019 to Sept 2020	Moderate to severe COVID-19 infection, confirmed by RT-PCR NP swabs.	I: 2-deoxy-D-glucose at 63 (N=22), 90 (N=22) and 126 (N=21) mg/kg/day<br>C: standard of care (N=44)	Time to maintaining oxygen saturation >=94% within 28 days.<br>Cox proportional hazards model<br>HR for 90mg group 2.3 (1.14, 4.64) compared to standard of care and 2.6 (1.49, 4.70) to pooled standard of care group.	2-deoxy-glucose 90mg/kg/day I beneficial in treatment of moderate to severe COVID-19.
Gupta [7]	Phase 3, double blind<br>37 sites across US, Canada, Brazil and Spain<br>Aug 2020 to March 2021	Non-hospitalized patients with COVID-19 infection and at least 1 risk factor for progression	I: Sotrovimab 500mg intravenous once (N=291)<br>C: placebo (N=292)	Hospitalization or death due to any cause through day 29<br>ITT Relative risk<br>Relative risk of 0.15 (97% CI 0.04 to 0.56)	Sotrovimab reduced risk of progression of COVI-19 requiring hospitalization in mild to moderate COVID-19 infection.

© 2023 Bai AD et al. *JAMA Network Open*.

Gupta [8]	Phase 3, double blind<br>57 centres across 5 countries (US, Canada, Brazil, Spain, Peru)<br>Aug 2020 to March 2021	Non-hospitalized patients with COVID-19 infection and at least 1 risk factor for progression	I: Sotrovimab 500mg intravenous once (N=528)<br>C: placebo (N=529)	Hospitalization or death due to any cause through day 29<br>ITT Relative risk reduction (RRR) by Poisson regression model<br>RRR of 79% (95% CI 50% to 91%)	Sotrovimab prevented progression of COVID-19 infection, reducing hospitalization and need for supplemental oxygen.
Gupta [9]	Open label<br>Single centre in India<br>May to Sept 2020	Healthcare workers at risk for COVID-19	I: Chyawanprash 12g PO twice daily for 30 days (N=98)<br>C: standard preventive regimen (N=95)	Incidence of RT PCR confirmed COVID-19 cases<br>0% were positive for COVID at end of follow up in both groups.	Chyawanprash was well tolerated, but bigger study needed to confirm its efficacy.
Chopra [10]	Open label<br>3 hospitals in India	Hospitalized adults with mild to moderate symptomatic COVID-19 confirmed by RT-PCR	I: Ayurevdic drug AYUSH 64 500mg PO twice daily (N=69)<br>C: standard of care (N=70)	Time to clinical recovery by Student's T-test<br>Proportion with clinical recovery at 28 days by Chi-square test<br>Time to recovery 6.5 + 2.4 days in AYUSH 64 group and 8.3 + 4.4 days with difference in means -3.03 to -0.59 days. Recovery proportion was 64 (69.8%) in AYUSH 64 group and 52.9% in standard of care group (p=0.046).	AYUSH 64 hastened recovery, reduced hospitalization and improved overall health in mild to moderate COVID-19.
Biber [11]	Double blinded<br>Israel<br>May 2020 to Jan 2021	Non-hospitalized patients with COVID-19 infection with or without symptoms	I: Ivermectin 0.2mg/kg for 3 days (N=47)<br>C: placebo (N=42)	Reduction of viral load on day 6 based on CT level >30<br>Chi-square and multivariate logistic regression model<br>34/47 (72%) in Ivermectin group and 21/42 (50%) in placebo arm reached primary endpoint. In the multivariate regression model, the adjusted odds ratio was 2.62 (95% CI 1.06 to 6.45).	Ivermectin lowered viral loads and viable culture.
Fisher [12]	Phase 2, open label<br>9 hospitals in UK<br>June 2020 to Feb 2021	Hospitalized patients with COVID pneumonia and CRP >=40mg/L	I 1: Namilumab 150mg IV once (N=57)<br>I2: Infliximab 5mg/kg IV once (N=35)<br>C: usual care (N=54)	Improvement in inflammation measured by CRP until day 14<br>Modified ITT, Bayesian multi-level model<br>Probability that namilumab and infliximab were superior to usual care in reducing CRP over time were 97% and 15% respectively.	Namilumab, and not infliximab, reduced inflammation in hospitalized patients with COVID-19 pneumonia, so it should be prioritized for further investigation.
Gaborit [13]	Phase 2a, double blind<br>4 sites in France<br>Aug to Dec 2020	Adults with moderate COVID-19 pneumonia requiring supplemental oxygen.	I 1: XAV-19 (swine glycol-humanized polyclonal antibody against SARS-CoV-2) 0.5mg/kg at day 1 and 5 (N=7)<br>I 2: XAV-19 2mg/kg at day 1 and 5 (N=1)<br>I 3: XAV-19 2mg/kg at day 1 (N=5)<br>C: placebo (N=5)	Pharmacokinetic measurement of serum concentration of XAV-19 at day 8 and tolerability at 29 days<br>ITT, Kruskal-Wallis test for pharmacokinetics and Fisher's test for adverse events<br>XAV-19 serum concentrations (μg/mL, median, range) at Cmax and at day 8 were 9.1 (5.2-18.1) and 6.4 (2.8-11.9), 71.5 and 47.2, and<br>50.4 (29.1-55.0) and 20.3 (12.0-22.7) for groups 1, 2 and 3, respectively (p=0.012). There were no hypersensitivity or infusion-related reactions, no discontinuation for adverse events and no serious adverse events.	Single intravenous dose of 2mg/kg of XAV-19 had high serum concentrations predictive of neutralizing activity

© 2023 Bai AD et al. *JAMA Network Open*.

Young [14]	Open label, cluster, non-inferiority trial<br>Secondary schools in England<br>April to June 2021	Staff and students at secondary schools	I: voluntary daily lateral flow device testing for school contacts with test-negative contacts remaining at school<br>C: Self isolation of COVID-19 contacts for 10 days	Students and staff who have symptomatic COVID-19, adjusted for community case rates, to estimate within-school transmission<br>COVID-19 related school absence<br>ITT, quasi-Poisson regression<br>Symptomatic COVID-19 infection occurred in 61.8/100k/week in the intervention arm and 59.1/100k/week in the control arm (incidence rate ratio (IRR) 0.96 95% CI 0.75-1.22 P=0.72).<br>COVID-related absences occurred in 1.5% in the intervention arm and 1.8% in the control arm with adjusted IRR of 0.80 95% CI 0.53 to 1.21)	Daily contact testing of school based contacts were non-inferior to self-isolation for control of COVID-19 transmission.
Clemency [15]	Phase 3, double blind<br>10 centres in US<br>June to Nov 2020	Non-hospitalized patients with symptomatic COVID-19 infection.	I: Ciclesonide metered-dose inhaler 160ug 2 actuations twice daily for 30 days (N=197)<br>C: placebo for 30 days (N=203)	Alleviation of all COVID-19 related symptoms by day 30<br>ITT, Cox proportional hazard model<br>Time to alleviation of all symptom was 19 days (95% CI 14.0 to 21.0) in ciclesonide arm and 19.0 days (95% CI 16. To 23.0) in placebo arm with hazard ratio of 1.08 (95% CI 0.84 to 1.38).	Ciclesonide did not achieve primary efficacy endpoint of time to alleviation of all COVID-19 symptoms.
Araujo [16]	Open label<br>Single centre in Brazil	Adult with rheumatoid arthritis with low disease or remission and on stable Methotrexate dose.	I: Withdraw methotrexate for 2 weeks after each vaccine dose (N=60)<br>C: maintain methotrexate after each vaccine dose (N=69)<br>Both groups received vaccine on day 0 and 28.	Anti-SARS-CoV2 IgG seroconversion and neutralizing antibody positivity at day 69<br>Generalized estimating equations.<br>Seroconversion occurred in 29 (78.4%) in withdrawal group and 30 (54.5%) in maintain group, p=0.019. For neutralizing antiboy positivity, there was no significance between the two groups (23 (62.2%) vs. 27 (49.1%) p=0.217.<br>In a multivariate analysis, methotrexate withdrawal had odds ratio of 4.6 (95% CI 1.43-15.04 p=0.010) with seroconversion.	Withdrawing methotrexate increased seroconversion in rheumatoid arthritis patients in whom disease is well controlled.
Tornero [17]	Open label<br>Single centre in Spain<br>April 2020 to Feb 2021	Adults hospitalized with COVID-19.	I: vagus nerve stimulation for 2 minutes 3 times daily (N=47)<br>C: standard of care (N=50)	No defined primary outcomes. Described incidence of specific clinical events and level of cytokines ad other biomarkers.<br>Per protocol. Chi-square or Fisher's exact test.<br>Greater decrease in CRP and procalcitonin level in vagus nerve stimulation group.	Vagus nerve stimulation led to significant reductions in inflammatory markers.
Hernandez-Cardenas [18]	Phase 3, double blind<br>Single centre at Mexico<br>April to July 2020	Patient with severe COVID-19 infection requiring hospitalization.	I: Hydroxychloroquine 400mg/day PO for 10 days (N=106)<br>C: placebo (N=108)	30-day mortality<br>ITT, Cox proportional hazard model<br>30-day mortality rate was 38% in Hydroxychloroquine group and 41% in placebo group with hazard ratio of 0.88 95% CI 0.51-1.53.	There was no beneficial effect or harm in use of Hydroxychloroquine to treat severe COVID-19.
Mok [19]	Blinding unclear<br>Community vaccine centres in Hong Kong China<br>March to Aug 2021	Adults who had received 2 doses of CoronaVac with low antibody response.	I: BNT162b2 as booster dose (N=40)<br>C: CoronaVac as booster dose (N=40)	Humoral immunogenicity as measured by surrogate virus neutralization test (sVNT) in plasma samples collected 1 month after 3rd dose of vaccination.<br>Unpaired T-test.<br>Mean % inhibition in<br>the sVNT test in the plasma for the BNT1626 and CoronaVac groups was 96.83%	BNT162b2 booster dose is significantly more immunogenic than CoronaVac booster for people who responded poorly to previous CoronaVac vaccination.

© 2023 Bai AD et al. *JAMA Network Open*.

				(SD 2.74) and 57.75% (SD 24.68), respectively (p<0.0001).	
Portal-Celhay [20]	Phase 2, double blind<br>47 sites across US	Outpatient with symptomatic or asymptomatic COVID-19 infection without risk factors for severe COVID-19.	I 1: REGEN-COV 300mg IV (N=80)<br>I 2: REGEN-CoV 600mg IV (N=68)<br>I 3: REGEN-CoV 1200mg IV (N=72)<br>I 4: REGE-CoV 2400mg IV (N=62)<br>I 5: REGEN-CoV 600mg SC (N=75)<br>I 6: REGEN-CoV 1200mg SC (N=73)<br>C: placebo IV or SC (N=77)	Time weighted average daily change from baseline in viral load from day 1 through 7.<br>Analysis of subset who were seronegative at baseline using ANCOVA.<br>Each REGEN-CoV treatment showed significant virologic reduction through day 7 whether in the IV or SC group compared to placebo.	In asymptomatic and low-risk symptomatic outpatients with COVID-19 infection, REGEN-CoV significantly reduced viral load at all IV and SC doses.
Singh [21]	Phase 2, double blind<br>Sites in UK, Moldova and Ukraine	Patients with PCR confirmed COVID-19 infection requiring hospitalization and supplemental oxygen.	I: pan-Janus kinase (JAK) inhibitor TD-0903 1mg (N=6), 3mg (N=7) and 10mg (NN=6) once daily for 7 days<br>C: placebo (N=6)	Safety, pharmacokinetics and oxygen saturation / fraction of inspired oxygen ratio.<br>ITT analysis.<br>Serious adverse events occurred in 5 patients (3 in placebo group; 2 in drug group). Mean change in SaO2/FiO2 were -49.5 (65.3) in placebo group, 108.9 (87.9) in 1mg group, 106.4 (87.8) in 3mg group, and 11.2 (106.3) in 10mg group.	Inhaled JAK inhibitor was generally well tolerated with trends toward improvement in SaO2/FiO2 ratio.
Sullivan [22]	Double blind<br>23 sites across US<br>June to Oct 2021	Symptomatic adults with COVID-19 infection regardless of risk factors for disease progression or vaccine status.	I: High titer convalescent plasma (N=592)<br>C: placebo (N=589)	COVID-19 related hospitalization within 28 days<br>Modified ITT, risk difference ad restricted mean survival time<br>Hospitalization occurred in 37/589 (6.3%) in placebo group and 17/592 (2.9%) with relative risk of 0.46 (one sided 95% CI 0.733 P=0.004).	High titer convalescent plasma significant decreased risk of hospitalization.
Huang [23]	Adaptive trial<br>40 sites in US<br>July to Sept 2021	Outpatients with COVID-19 who would be eligible for monoclonal antibody therapy as per EUA.	I: casirivimab-imdevimab (N=2454)<br>C: sotrovimab (N=1104)	Hospital-free days within 28 days<br>ITT Bayesian cumulative logistic model<br>Median hospital free days were 28 (IQR 28-28) for both groups. the median adjusted odds ratio for hospital-free days was 0.88 (95% credible interval, 0.70–1.11) for sotrovimab. This odds ratio yielded 86% probability of inferiority of sotrovimab versus casirivimab and imdevimab, and 79% probability of equivalence.	The two monoclonal antibody therapy appeared similar, but did not meet the prespecified criteria for inferiority or equivalence.
Weinreich [24]	Phase 3, double blind, adaptive trial<br><br>Sept 2020 to Jan 2021	Non-hospitalized adults with confirmed COVID-19 infection with symptoms for 7 days or less who had 1 or more risk factors for disease progression	I 1: casirivimab-imdevimab 2400mg IV once (N=1355)<br>I 2: casirivimab-imdevimab 1200mg IV once (N=736)<br>C: placebo (N=2089)	COVID-19 related hospitalization or death due to any cause through day 29<br>Modified ITT, stratified Cochran-Mantel-Haenszel test, Cox regression model<br>Both REGEN-COV 2400mg and 1200mg significantly reduced Covid-19-related hospitalization or all-cause death compared to placebo (71.3% reduction [1.3% vs 4.6%; p<0.0001] and 70.4% reduction [1.0% vs 3.2%; p=0.0024], respectively).	Casirivimab-imdevimab was effective in reducing risk for hospitalization and all-cause deaths.

© 2023 Bai AD et al. *JAMA Network Open*.

Parikh [25]	Open label<br>7 sites in India	Hospitalized patients with moderate to severe COVID-19 infection.	I: COVID-19 hyper-immune globulin (HIG) solution IV on day 1 and 2 (N=30)<br>C: standard of care (N=29)	Change in ordinal scale by day 8<br>Modified ITT, Wilcoxon rank-sum test<br>Mean change in ordinal scale was 1.7 (1.6) for test arm and 2.0 (1.68) for control arm (P=0.3677)	HIG was safe and well-tolerated. There was no significant difference between HIG and standard of care with respect to improvement in ordinal scale.
Ely [26]	Phase 3, double blind<br>18 sites in Argentina, Brazil, Mexico and US<br>Dec 2020 to April 2021	Critically ill patients with confirmed COVID-19 on baseline mechanical ventilation or ECMO.	I: Baricitinib 4mg PO daily for 14 days (N=51)<br>C: placebo PO daily for 14 days (N=50)	All-cause mortality through 28 and 60 days<br>ITT, log-rank tet and Cox proportional hazard model<br>Mortality at 28 days was 39.2% in Baricitinib group and 58% in placebo group with hazard ratio of 0.54 (95% CI 0.31 to 0.96 p=0.030). There was also significant reduction in 60-day mortality (45·1% vs 62·0%; HR=0·56 [95%CI 0·33–0·97]; p=0·027).	Baricitinib decreased mortality in critically ill patients already on mechanical ventilation or ECMO.
Fedrizzi [27]	Double blinded<br>Single centre at Brazil<br>July 2020 to Feb 2021	Healthy healthcare workers or volunteers at risk for COVID-19 infection	I: Measles-Mumps-Rubella (MMR) vaccine for 2 doses 8 weeks apart (N=246)<br>C: placebo vaccine for 2 doses 8 weeks apart (N=178)	Proportion with symptomatic COVID-19 infection<br>ITT, relative risk<br>Relative risk of 0.52 95% CI 0.33 to 0.83 p=0.004 for symptomatic COVID-19.	MMR vaccine significantly reduced risk of symptomatic COVID-19 infection.
Balint [28]	Open label<br>Single centre in Germany<br>Feb to July 2021	Hospitalized patients with moderate to severe COVID-19 infection.	I: slow paced 20-minute breathing exercise 3 times a day with 6 breaths per minute (N=23)<br>C: standard of care (N=23)	IL-6 level<br>ITT, multi-level mixed effect linear regression model<br>IL-6 trajectory was lower in intervention group (effect size Cohens f2=0.11, LR-test p=.040).	Slow-paced breathing is effective in reducing IL-6 in COVID-19 pneumonia, though with uncertain clinical importance.
Kolev [29]	Open label<br>Single centre in Bulgaria<br>Nov 2020 to May 2021	Healthy volunteers	I: Echinacea purpurea extract daily (N=59)<br>C: No treatment and observation (N=60)	Incidence of viral respiratory tract infections<br>ITT, Chi-square, RR and OR<br>Viral infection was diagnosed in 11 patients for the treatment group and 14 in the control group RR=0.748 p=0.196. COVID-19 infection, the RR was 0.369 p=0.030	Echinacea decreased risk of respiratory viral infections including COVID-19 infection.
Breza [30]	Cluster RCT<br>13 states in US	General public. Counties were allocated to high-intensity or lower-intensity.	I: High intensity counties with more messages encouraging viewers to stay home for holidays, which were disseminated to Facebook users (N=410)<br>C: Low intensity counties with less messages to Facebook users (N=410)	Aggregate holiday travel measured using mobile phone location data and COVID-19 infection at zip-code level.<br>ITT, regression model<br>Average distance travelled in high-intensity counties<br>decreased by -0.993 percentage points (95% CI -1.616, -0.371, p-value 0.002). COVID-19 infections recorded in the two-week period starting five days post-holiday<br>declined by 3.5 percent (adjusted 95% CI [-6.2 percent, -0.7 percent], p-value 0.013).	High coverage Facebook ad campaigns that encourage people to stay home was effectiveness in reducing travelled distance before holidays and new COVID-19 infections.
Lattman [31]	Open label<br>2 hospitals in India	Patients with symptomatic COVID-19 infection that is not severe (oxygen	I: PNB001 (a CCK-A agonist and CCK-B antagonist) 10mg PO 3 times daily for 14 days (N=20)<br>C: standard of care (N=20)	Mean change in 8 point WHO ordinal scale by day 14 and 28-day mortality<br>Paired Wilcoxon rank-sum test and time to event with Kaplan-Meier survival curves.<br>Mean ordinal scale was 0.22 in PNB001 arm and 1.12 in standard of care arm, where mean change	PNB001 resulted in significant clinical improvement in moderate COVID-19 infection.

© 2023 Bai AD et al. *JAMA Network Open*.

		saturation >94% on room air).		in scale from baseline to day 14 was statistically significant (p=0.0421). 1 patient died I PNB001 arm and 2 patients died in standard of care arm (hazard ratio 2.0 95% CI 0.18 to 22.05 p=0.5637).	
Gaughan [32]	Phase 2, open label<br>2 hospitals in Edinburgh, Scotland<br>July 2020 to Feb 2021	Adults with symptomatic COVID-19 infection requiring oxygen and pneumonia on chest X-ray.	I: GB0139 (inhaled galectin-3 inhibitor) 10mg twice daily for 48 hours, then once daily for 14 days plus standard of case (N=20)<br>C: standard of care including dexamethasone, tocilizumab, remdesivir, heparin and antibiotics as indicated (N=21)	Composite primary endpoint (safety): vitals, examination, ECG, laboratory markers.<br>Change in oxygen requirement from baseline<br>Modified ITT, Bayesian generalised linear mixed effects model<br>There were no safety or tolerability concerns. The rate of FiO2 (% per day) decline was significantly<br>greater with GB0139, with a posterior mean difference of −1·51 (95% HPD −2·90, −0·189) versus control.	GB0139 was well tolerated, achieved relevant plasma concentrations and is associated with decrease in inflammatory markers and reduction in inspired oxygen requirement.
McCreary [33]	Open label, platform trial<br>Sites within integrated network in Pennsylvania, US<br>March to June 2021	Patients who met Emergency Use Authorization (EUA) criteria for monoclonal antibody for COVID-19 infection. These patients had positive PCR test, symptoms for under 10 days and risk factors for progression to severe COVID-19 infection.	I 1: Bamlanivimab (N=128)<br>I 2: Bamlaivimab-etesevimab (N=885)<br>I3: Casirivimab-imdevimab (N=922)	Hospital free days (days alive and free of hospital) within 28 days<br>Modified ITT, Bayesian cumulative logistic model<br>Median hospital–free days were 28 (IQR, 28 to 28) for 27 each mAb group. Relative to casirivimab-imdevimab, the 29 median adjusted odds ratios were 0.58 (95% credible interval (CI), 0.30 to 1.16) and 0.94 (95% 30 CI, 0.72 to 1.24) for the bamlanivimab and bamlanivimab-etesevimab groups, respectively. These odds ratios yielded 91% and 94% probabilities of inferiority of bamlanivimab versus bamlanivimab-etesevimab and casirivimab-imdevimab respectively, and an 86% probability of equivalence between bamlanivimab-etesevimab and casirivimab-imdevimab, at the prespecified odds ratio bound of 0.25.	No monoclonal antibody met prespecified criteria for statistical inferiority or equivalence.
Sobngwi [34]	Open-label, non-inferiority trial<br>Single centre in Cameroon<br>March to April 2021	Patients with mild COVID-19 infection with or without symptoms who had oxygen saturation >94%..	I: Doxycycline 100mg PO twice daily for 7 days (N=97)<br>C: Hydroxychloroquine 400mg PO daily for 5 days and Azithromycin 500mg PO at day 1 ad 250mg PO daily form day 2 through 5 (N=97)	Proportion of clinical cure at day 3, 10 and 30.<br>Non-inferiority margin set at within 20% margin.<br>ITT, absolute risk difference<br>At day 3, 74/92 (80.4%) participants on Doxycycline<br>versus 77/95 (81.1%) on Hydroxychloroquine-Azithromycin -based protocols were asymptomatic<br>(p=0.91). At day 10, 88/92 (95.7%) participants on Doxycycline versus 93/95 (97.9%) on Hydroxychloroquine-Azithromycin were asymptomatic (p=0.44). At day 30 all participants were<br>asymptomatic.	Doxycycline was as effective and safe a Hydroxychloroquine-Azithromycin for mild COVID-19 infection.

© 2023 Bai AD et al. *JAMA Network Open*.

Kyriazopoulou [35]	Double blind<br>37 sites in Greece and Italy<br>Dec 2020 to March 2021	Hospitalized patients with moderate to severe COVID-19 pneumonia and plasma urokinase plasminogen activator receptor (suPAR) >=6ng/mL.	I: anakinra 100mg subcutaneous once daily for 10 days (N=405)<br>C: placebo subcutaneous once daily for 10 days (N=189)	Overall clinical status based on the 11-point World Health Organization ordinal Clinical Progression Scale (WHO-CPS) at day 28<br>ITT, ordinal regression analysis using logit function<br>Anakinra-treated patients were distributed to lower strata of WHO-CPS by<br>day 28 (adjusted odds ratio-OR 0.36; 95%CI 0.26-0.50; P<0.001); anakinra protected<br>from severe disease or death (6 or more points of WHO-CPS) (OR: 0.46; P: 0.010).<br>The median absolute decrease of WHO-CPS in the placebo and anakinra groups<br>from baseline was 3 and 4 points respectively at day 28 (OR 0.40; P<0.0001)	Anakinra treatment guided by suPAR leads to improvement of clinical status in moderate and severe COVID-19 pneumonia.
Mikhaylov [36]	Open label<br>Single centre in Russia<br>May to Aug 2020	Medical staff managing patients with suspected or confirmed COVID-19	I: Bromhexine hydrochlorie 8mg PO 3 time daily (N=25)<br>C: controls	Positive PCR test for SARS-CoV-2 or signs of clinical infection within 28 days and at week 8.<br>ITT, Mann-Whitney U test<br>Endpoint occurred in 2/25 (8%) of treatment group and 7/25 (28%) of control group P=0.07.	Bromhexine hydrochloride prophylaxis did not decrease combined rate of positive SARS-CoV-2 test or clinical infection.
Goligher [37]	Open-label, adaptive trial<br>International<br>April to Dec 2020	Patients with severe COVID-19 defined as requirement for organ support defined as provision of ICU-level respiratory or cardiovascular organ support.	I: Therapeutic anticoagulation with heparin (N=529)<br>C: Thromboprophylaxis as per usual care (N=545)	Ordinal scale combining in-hospital mortality and days free of organ support to day 21<br>ITT, Bayesian cumulative logistic model<br>Median organ support free days were 3 days (IQR -1, 16) in treatment group and 5 days (IQR -1, 16) in control group with adjusted odds ratio of 0.87 95% CI 0.70 to 1.08). Posterior probability of futility 99.8%.	Therapeutic anticoagulation did not improve hospital survival or days free of organ support compared to usual care pharmacological thromboprophylaxis.
Syed [38]	Phase 2, placebo controlled<br>2 sites in Pakistan<br>Started in May 2020	Healthcare workers at high risk for COVID-19 exposure.	I 1: Hydroxychloroquie 400mg PO twice daily on day 1 followed by 400mg weekly (N=48)<br>I 2: Hydroxychloroquine 400mg PO every 3 weeks (N=51)<br>I 3: Hydroxychloroquie 200mg PO every 3 weeks<br>C: placebo (N=46)	COVID-19 free survival by end of study<br>Chi-square, ANOVA<br>During the treatment, 15 out of 48 in group 1, 19 out<br>183 of 51 in group 2, 8 out of 55 in group 3, and 7 out of 46 in control group tested positive for<br>184 COVID-19.	In conclusion, there was no significant reduction in the SARS-CoV-2 transmission with PrEP administration of Hydrochloroquine among the enrolled healthcare personnel.
Cadegiani [39]	Double blind<br>8 sites in Brazil<br>Feb to April 2021	Hospitalized patients with COVID-19 infection, but not requiring mechanical ventilation	I: proxalutamide 300mg for 14 days (N=317)<br>C: placebo for 14 days (N=328)	Recovery rate defined as score 1 and 2 on the 8-point COVID-19 ordinal scale on day 14<br>ITT, Wilcoxon-rank sum test<br>The 14-day median ordinal scale score<br>in the proxalutamide group was 1 (interquartile range [IQR]=1–2) versus 7 (IQR=2–8) for placebo, P<0.001. The 14-day recovery rate was 81.4% for proxalutamide and 35.7% for<br>placebo (recovery ratio, 2.28; 95% CI 1.95–2.66 [P<0.001]).	Proxalutamide resulted in higher recovery rate and lower mortality rate for patients hospitalized for COVID-19 infection.

© 2023 Bai AD et al. *JAMA Network Open*.

Cadegiani [40]	Double blind trial<br>2 sites in Brazil<br>Jan to Feb, 2021	Female outpatients with confirmed COVID-19 infection	I: proxalutamide 200mg/day for 7 days (N=75)<br>C: placebo for 7 days (N=102)	Hospitalization rates through 30 days after randomization.<br>ITT, Chi-square<br>The 30-day hospitalization rate was 2.7% in the proxalutamide<br>arm and 18.6% in the placebo arm (p<0.001), with a hospitalization risk ratio (RR) of<br>0.14 [95% confidence interval (CI), 0.03-0.59].	Proxalutamide reduce hospitalization for outpatients with COVID-19 infection.
Ader [41]	Open label, adaptive trial<br>32 sites in France and Luxembourg<br>March to June 2020	Inpatients with COVID-19 requiring oxygen and / or ventilatory support.	I 1: lopiavir/ritonavir (400/100mg) PO every 12 hours for 14 days (N=145)<br>I 2: lopiavir/ritonavir (400/100mg) PO every 12 hours plus IFN-beta-1a 44ug subcutaneous on days 1, 3 and 6 (N=145)<br>I 3: hydroxychloroquine 400mg PO twice daily on day 1 then 400mg PO daily for 9 days (N=145)<br>C: standard of care (N=148)	The primary outcome was the clinical status at day 15, measured by the<br>WHO 7-point ordinal scale.<br>ITT, proportional odds model<br>Adjusted Odds Ratio (aOR) were not in favor of investigational treatments: lopinavir/ritonavir versus control, aOR 0.83, 95%CI, 0.55 to 1.26, P=0.39; lopinavir/ritonavir-IFN-β-1a versus control, aOR 0.69, 95%CI, 0.45 to 1.04, P=0.08; hydroxychloroquine versus control, aOR 0.93, 95%CI, 0.62 to 1.41, P=0.75	None of the study drugs resulted in improvement of clinical status.
Fralick [42]	Open label, pragmatic trial<br>15 hospitals in Canada and US<br>May 2020 to May 2021	Inpatients with confirmed COVID-19 infection requiring supplemental oxygen.	I: instructions for prone positioning (N=126)<br>C: standard of care without instruction to prone position (N=122)	Composite of in-hospital death, mechanical ventilation or worsening respiratory failure.<br>ITT, multivariable logistic regression model.<br>The rate of<br>the primary outcome was similar between the prone group (18 [14.3%] events) and the standard of care group (17 [13.9%] events), odds ratio 0.92 (95% CI 0.44 to 1.92).	Among hypoxic but not critically patients with COVID-19 in hospital, a multifaceted intervention to increase prone positioning did not improve outcomes.
Hernandez-Bernal [43]	Phase 1/2, double blind<br>Single hospital at Cuba<br>Dec 2020 to April 2021	Healthy volunteers	I 1: SARS-CoV-2 recombinant spike protein vaccine (RBD) 25ug intramuscular on short (N=171) or long (N=115) schedule<br>I 2: RBD 50ug intramuscular on short (N=173) or long (N=113) schedule<br>C: placebo intramuscular on short (N=175) or long (N=111) schedule	Proportion with seroconversion of anti-RBD IgG antibody and safety.<br>PP, descriptive with Chi-square<br>Severe adverse events were not reported. At day 56, seroconversion of anti-RBD IgG was seen in 95.2 % of the<br>participants (20/21) for the 50μg group and 81 % of the participants (17/21) for the 25μg group, and none in the placebo group (0/22). At day 70, seroconversion of anti-<br>RBD IgG was seen in 100% of the participants (21/21) for the 50μg group and 94.7% of the participants (18/19) for the 25μg group, and none in the placebo group (0/22). In the phase 2 trial, global seroconversion<br>rates of anti RBD-IgG measured at day 42 were, 79.5% and 89.6% for the 25 μg and 50 μg group, respectively (p=0.00) and at day 56, were very similar with 77.7% and 89.2% for the 25 μg and 50 μg group, respectively (p=0.00).	The tested vaccine was safe, well tolerated and induced humoral immune response against SARS-CoV-2.

© 2023 Bai AD et al. *JAMA Network Open*.

Lescure [44]	Phase 2/3, double blind<br>45 sites across 11 countries<br>March to July 2020	Hospitalized patients with severe COVID-19 infection and pneumonia	I 1: Sarilumab 400mg subcutaneous once (N=173)<br>I 2: Sarilumab 200mg subcutaneous once (N=159)<br>C: placebo subcutaneous (N=84)	Time to >=2 point improvement on 7-point scale at day 29.<br>Modified ITT, log-rank test, Cox proportional hazards model<br>There were no significant differences in median (95% CI) time to ≥2-point improvement between placebo (12·0 [9·0–15·0] days) and sarilumab groups (200 mg: 10·0 [9·0–12·0] days, p=0.96, log-rank test; 400 mg: 10·0 [9·0–13·0] days, p=0.34)	Sarilumab did not result in significant improvement for patients hospitalized with COVID-19 infection in this trial.
Perkins [45]	Open label, adaptive trial<br>48 hospitals in UK<br>April 2020 to May 2021	Hospitalized adults with acute respiratory failure due to COVID-19 defined as oxygen saturation 95% or below despite receiving oxygen of at least 0.4. .	I 1: continuous positive airway pressure (CPAP) (N=380)<br>I 2: High flow nasal oxygenation (HFNO) (N=417)<br>C: conventional oxygen therapy (N=475)	Tracheal intubation or mortality within 30 days.<br>ITT, logistic regression models<br>Composite endpoint occurred in 137/377 (36.3%) for CPAP, 184/414 (44..4%) for HFNO and 158/356 (44/4%) for conventional oxygen therapy with unadjusted OR of 0.72 95% CI 0.53 to 0.96 P=0.03 for CPAP vs. conventional oxygen therapy and 0.97 95% CI 0.73 to 1.29 P=0.85 for HFNO vs. conventional oxygen therapy.	CPAP reduced risk of intubation or death within 30 days in hospitalized adults with respiratory failure due to COVID-19. HFNO had similar outcome as conventional oxygen therapy.
Roozen [46]	Open label<br>Single centre at Netherlands<br>April to May 2021	Healthy adults	I 1: 10ug mRNA-1273 vaccine intradermal on day 1 and 29 (N=10)<br>I 2: 20ug mRNA-1273 vaccine intradermal on day 1 and 29 (N=15)<br>C: 20ug mRNA-1273 vaccine intramuscular on day 1 and 29 (N=15)	Tolerability and safety<br>Note immunogenicity was a secondary outcome based on seroconversion of IgG antibody.<br>Modified ITT, descriptive<br>There were no serious adverse events across all groups.<br>All participants seroconverted after first vaccine dose.	Intradermal administration of 10 µg and 20 µg mRNA-1273 vaccine was well tolerated and safe, and resulted in a robust antibody response.
Tornling [47]	Double blind<br>8 sites in India<br>July to Sept 2020	Hospitalized patients with COVID-19 infection with CRP of 50-150mg/L and not requiring mechanical ventilation	I: novel oral angiotensin 2 type 2 receptor agonist C21 100mg PO twice daily for 7 days (N=51)<br>C: placebo PO twice daily for 7 days (N=55)	CRP change from baseline to end of treatment<br>ANCOVA<br>CRP decreased by 81% in C21 group and 78% in placebo group (p=0.489)	Although the primary endpoint (reduction in CRP) was not different between C21 and placebo<br>treated patients after 7 days of treatment, secondary analyses of<br>clinical outcomes suggest that C21 treatment may be beneficial in<br>reducing the extended need for supplemental oxygen therapy.
Resende [48]	Phase 2, open label<br>Single centre in Brazil<br>Sept to Dec 2020	Adult patients admitted to hospital with confirmed COVID-19 infection and severe acute respiratory syndrome.	I: secukinumab 300mg subcutaneously on ay 0 and 7 (N=25)<br>C: standard of care (N=25)	Ventilator free days by day 28, with score of 0 for mortality<br>PP and ITT, Mann-Whitney test<br>Ventilatory free days was 23.7 (SD 9.9) in treatment group and 23.8 (SD 9.6) in control group (P=0.62).	Secukinumab was not efficacious in treatment of COVID infection.
Dai [49]	2 RCTs	UCLA health patients who are	First RCT	Proportion of patients who scheduled first dose appointment within 6 days.	Text-based nudges can substantially increase and

	Outpatients withi UCLA health in US<br>Jan to Feb 2021	eligible for COVID-19 vaccination	I: text message on first reminder date to schedule vaccination<br>I 1: simple text (N=22,588)<br>I 2: ownership text (N=22,651)<br>I 3: simple text with video (N=22,687)<br>I 4: ownership text with video (N=22,624)<br>C: holdout arm with no text message (N=22,679)<br>Second RCT<br>I: text message on secondary reminder date to schedule vaccination<br>I 1: simple self (N=12,958)<br>I 2: simple prosocial (N=12,964)<br>I 3: early access self (N=12,960)<br>I 4: early access prosocial (N=12,938)<br>I 5: fresh start self (N=12,952)<br>I 6: fresh start prosocial (N=12,926)	Ordinary least squares regression that control for patient factors.<br>In the Holdout arm, 6.01% of patients made the first-dose appointment within six days of the first reminder date. Receiving a text message boosted appointment rates within six days by 5.14 percentage points (p<0.001). Within the Text Message arm, adding the ownership language increased both<br>appointment and vaccination rates by 1.23 and 0.82 percentage points,<br>respectively (p's<0.001). By contrast, inviting patients to watch an educational video did not improve either outcome variable (p's>0.52).<br>Getting a second reminder increased patients' likelihood of scheduling the first-dose appointment within six days by 1.26 percentage points (a 52.28% increase; p<0.001). All message types (those highlighting personal<br>benefits, prosocial benefits, early access, and opportunity for a fresh start) boosted appointments rates (p's<.001) and vaccinations (p's<0.03).	accelerate COVID-19 vaccinations.
Gaitan-Duarte [50]	Open label pragmatic trial<br>Aug 2020 to March 2021<br>6 hospitals in Colombia	Adults hospitalized with COVID-19 pneumonia.	I 1: emtricitabine 200mg + tenofovir disoproxil 300mg PO daily for 10 days (N=160)<br>I 2: colchicine 0.5mg PO twice daily plus roxuvastatin 40mg PO daily for 14 days (N=153)<br>I 3: emtricitabine 200mg + tenofovir disoproxil 300mg PO daily for 10 days plus colchicine 0.5mg PO twice daily plus roxuvastatin 40mg PO daily for 14 days (N=159)<br>C: usual standard of care (N=161)	All-cause mortality in 28 days.<br>Modified ITT, relative risk (RR) and risk differences (RD) were calculated using log-binomial general estimating equation<br>The cumulative incidence of death through day 28 was 17/159 (10.7%) in the emtricitabine/tenofovir + colchicine + rosuvastatin arm, 22/153 (14.4%) in the colchicine + rosuvastatin arm, 22/160 (13.8%) in the emtricitabine/tenofovir arm, and 28/161 (17.4%) in the standard of care arm, with adjusted risk differences (aRD) against the standard<br>treatment of -0.07 (95% confidence interval [CI], -0.17 to 0.04), aRD -0.03 (95%CI: -0.11 to 0.05) and aRD: -0.05 (95%CI: -0.15 to 0.05), respectively.	Use of emtricitabine/tenofovir + colchicine + rosuvastatin may be a treatment alternative, because it reduced need for invasive mechanical ventilation by 6% compared to standard of care.
Arnardottir [51]	Single blind (participants)<br>Single centre in Sweden<br>June to Dec 2020	COVID-19 infection requiring hospitalization	I: omega 3 polyunsaturated fatty acid 2mg/kg IV infusion daily for 5 days (N=10)<br>C: placebo IV infusion for 5 days (N=12)	Changes in inflammatory biomarkers (white blood cell count (WBC), C-reactive protein (CRP), lipidomic profiling and cytokines) after 5 days treatment or study end.<br>Paired T-test, linear mixed model<br>Neutrophil to lymphocyte ratio was significantly decreased in treatment group. CRP decreased from 62 (20-75) to 19 (3.8-27) mg/L after n-3 PUFA (P=0.08) and from 65 (40-92) to 32 (24-	n-3 PUFA changed biomarkers of WBC and lipid metabolites, but not CRP or cytokines.

© 2023 Bai AD et al. *JAMA Network Open*.

				40) mg/L after placebo treatment (P=0.08). EPA- and DHA concentrations were increased 3.7 (2.5-5.4) fold and 2.0 (1.5-2.5) fold from baseline in treatment group. In contrast, the EPA and DHA plasma levels were not significantly altered in the placebo group; EPA 1.0 (0.8-1.2) and DHA 1.0 (0.9-1.2) -fold at the end of the study as compared to baseline.There were no significant changes in plasma cytokines.	
Pan [52]	Phase 1 & 2, double blind<br>China	Healthy adults age 18 to 59 years who are seronegative for SARS-CoV-2 and had negative PCR for SARS-CoV-2.	Phase 1<br>I 1: KCONVAC 5ug on day 0 and 14 (N=24)<br>I 2: KCONVAC 10ug on day 0 and 14 (N=24)<br>C: placebo on day 0 and 14<br>Phase 2 (N=12)<br>I 1: KCONVAC 5ug on day 0 and 14 (N=100) or 28 (N=100)<br>I 2: KCONVAC 10ug on day 0 and 14 (N=100) or 28 (N=100)<br>C: placebo on day 0 and 14 (N=50) or 28 (N=50)	Safety and seroconversion / titre of neutralization antibody, seroconversion of receptor binding domain (RBD)-IgG 28 days after second dose<br>Safety analysis for adverse events, PP for seroconversion. Immunogenicity was expressed using seroconversion percentage, geometric mean titre (GMT), and the associated 95% confidence interval (CI).<br>No adverse event of grade 3 or more was reported. One serious adverse vent (foot fracture) was reported in phase 1 trial.<br>KCONVAC induced significant antibody response. 87·5% (21/24) to 100% (24/24) of participants in the phase 1 trial and 83·0% (83/100) to 100% (99/99) of participants in the phase 2 trial seroconverted for neutralising antibody to live virus, neutralising antibody to pseudovirus, and RBD-IgG after receiving two doses. In contrast, in placebo group no (0/12) participant seroconverted for the three antibodies in the phase 1 trial, and only two (2/48) participants<br>at Day 0/14 regimen and one (1/49) participant at Day 0/28 regimen seroconverted in the phase 2 trial/ The differences in seroconversion percentages between vaccine groups and placebo groups are statistically significant (p<0.0001) for both dosages and both regimens, in both phase 1 and phase 2<br>246 trials.	KCONVC is well tolerated and able to induce a robust antibody response.
Almanza-Reyes [53]	Open label<br>Single centre in Mexico<br>April to June 2020	Health workers at high risk of acquiring COVID-19 infection	I: ARGOVIT silver nanoparticles mouthwash and nose rinse (N=114)<br>C: Mouthwash and nose rinse with conventional mouthwash (N=117)	Incidence of SARS-CoV-2 infections<br>Chi-square test, Fisher's exact test and likelihood ratio test, logistic regression test.<br>COVID infection occurred I 2/114 (1.8%) in the treatment group and 33/117 (28/2%) in the control group (P<0.001).	Silver nanoparticles in mouthwash and nose rinses reduce risk of COVID-19 infections in high risk healthcare workers.
Faramarzi [54]	Phase 3 double blind<br>Single centre in Iran<br>March to June 2021	120 patients with COVID-19 pneumonia in intensive care units	I: novel herbal antiviral preparation (*Zataria multiflora Boiss*, *Glycyrrhiza glabra*, *Cinnamomum Vermont*, *Allium sativuml*, and *Syzygium*	COVID-19 associate deaths through day 14<br>Chi-square, logistic regression, Cox-proportional hazard model<br>Death occurred in 5 (8.3%) in the treatment group and 36 (60%) in the control group. Risk ratio 0.14	The novel antiviral preparation tested in this trial significantly improved the survival rate and reduced mortality in critically ill patients with COVID-19.

© 2023 Bai AD et al. *JAMA Network Open*.

			aromaticum) every 3 hours for up to 2 weeks (N=60)<br>C: placebo for up to 2 weeks N=60)	95% CI 0.05 to 0.32 P<0.001. Age adjusted odds ratio for placebo to treatment group was 14.63 95% CI 4.95 to 43.24 P<0.001. Cox regression model showed hazard ratio of 0.12 95% CI 0.04 to 0.31 P<0.001.	
Patel [55]	Double blind<br>108 sites across 17 countries<br>May to Nov 2020	Adults hospitalized with severe COVID-19 respiratory failure and systemic inflammation.	I: Otilimab single 90mg IV infusion (N=395)<br>C: placebo (N=398)	Proportion of patients alive and free of respiratory failure at day 28.<br>Modified ITT, logistic regression adjusting for treatment, age group and clinical status at baseline.<br>The proportion of being alive and free of respiratory failure was 71% in Otilimab group and 67% in placebo group (risk difference 5.3% 95% CI -0.8 to 11.4 p=0.09). In the moel, there was significant model adjusted difference of 19.1% 95% CI 5.2 to 33.1 p=0.009 in patients >=70 years.	Otilimab demonstrated benefit in patients age >=70 years.
Tardif [56]	Double blind<br>Across 6 countries<br>March to Dec 2020	Non-hospitalized patients with COVID-19 infection and at least one risk factor for progression.	I: colchicine 0.5mg PO twice daily for 3 days and then daily for 30 days (N=2235)<br>C: placebo for 30 days (N=2253)	Composite of death or hospitalization for COVID-19 in 30 days.<br>ITT, chi-square, odds ratio<br>Primary endpoint occurred in 104 (4.7%) of patients in colchicine group and 131 (5.8%) in placebo group (odds ratio 0.79 95% CI 0.61 to 1.03 P=0.08)	In non-hospitalized patients with COVID-19, colchicine reduced composite rate of death or hospitalization.
Rossignol [57]	Double blind<br>36 centers in US ad Puerto Rico<br>Aug 2020 to Jan 2021	Outpatients with mild or moderate laboratory confirmed COVID-19	I: Nitazoxanide extended release 600mg PO twice daily for 5 days (N=184)<br>C: placebo for 5 days (N=195)	Time to sustained response based on symptoms<br>Modified ITT, stratified Gehan-Wilcoxon test<br>Median time to sustained response was 13.28 (IQR 6.3 to 21) in Nitazoxanide group and 12.4 (7.2 to 21) for placebo group (p=0.88).	Nitazoxanide is well tolerated and reduce risk of progression to severe illness.
Low [58]	Phase 1 and 2 double blind trial<br>Single centre in Singapore	Healthy adult volunteers	I: ARCT-021 vaccine one dose in phase 1, 2 doses separated by 28 days in phase 2 (N=80)<br>C: placebo (N=26)	Local and systemic solicited and unsolicited adverse events up to 14 days.<br>Descriptive only.<br>Numbers and percentages described in Figure 2. 62.8% participants in vaccine group and 46.4% in placebo group reported systemic adverse events. There were no grade 4 events reported. There were 5 severe adverse events occurring in the vaccine group mainly in the 10ug group.	The ARCT-021 vaccine is generally safe up to 7.5ug dose and is also immunogenic.
Lundgren [59]	Double blind<br>31 sites in US, Denmark and Singapore<br>Aug to Oct 2021	Hospitalized patients with COVID-19 infection not o mechanical ventilation.	I: Bamlanivimab 7000mg IV once (N=163)<br>C: placebo (N=151)	Time to sustained recovery through 90 days of follow-up<br>Modified ITT, recovery rate ratio (RRR) using Fine-Gray model<br>Sustained recovery was achieved by 144 (88%) in bamlanivimab group and 136 (90%) in placebo group. RRR was 0.99 (95% CI 0.79 to 1.22, p=0.89). RRR was 1.24 (95% CI 0.90 to 1.70) for those without antibody and 0.74 (95% CI 0.54 to 1.00) for those with antibody at study entry (p=0.02 for interaction). Among those who were nAb negative, the difference between	Treatment benefit from bamlanivimab compared to placebo differed according to the presence of neutralizing antibodies at study entry, with possible benefit in seronegative patients with high plasma antigen or high nasal viral RNA levels.

© 2023 Bai AD et al. *JAMA Network Open*.

				bamlanivimab and placebo were more evident if plasma antigen or nasal-swab viral RNA were above the median entry levels (RRRs=1.48; 95% CI: 0.99-2.23 and 1.94; 95% CI: 1.25-3.00, respectively).	
Liu [60]	Open label<br>Single centre in China<br>Feb to March 2020	Ault with confirmed COVID-19 infection	I: Chinese medicine Q-14 10g granules twice daily for 14 days (N=99)<br>C: standard of care (N=96)	Conversion of SARS-CoV-2 viral assay<br>ITT, Wilcoxon-rank sum test, log rank test, Cox regression model<br>No difference between the two groups in conversion time (FAS: Median (IQR): 10.00 (9.00-11.00) vs. 10.00 (9.00-11.00); Mean rank: 67.92 vs. 81.44; P=0.051.). Hazard ratio was 1.165 95%CI: 0.84-1.61, P=0.27.<br>P=0.31 by log rank test.	Administration of Q-14 on standard care for COVID-19 was useful for improvement of 65 symptoms (such as fever, cough, fatigue and chest discomfort), while did not result in<br>66 a significantly higher probability of negative conversion of SARS-CoV-2 viral assay.
Li [61]	Observer blinded<br>Single centre in Liashui County, Jiangsu Province, China<br>May 2021	Healthy adults age 18-59 years who were primed with 1 or 2 doses of CoronaVac	I: Booster of Convidecia (heterologous dose) including 96 who were primed with 2 doses and 51 who were primed with 1 dose.<br>C: Booster of CoronaVac (homogeneous dose) including 102 who were primed with 2 doses and 50 who were primed with 1 dose.	Adverse reactions within 28 days. Geometric mean titres of neutralizing antibody against live SARS-CoV-2 at 14 days<br>Chi-square test for adverse reactions and T-test for log transformed antibody titers as well as Pearson's correlation.<br>In both two-dose and three-dose regimen cohorts, Convidecia recipients reported more adverse reactions than CoronaVac recipients with p values of <0.001 and 0.019,<br>Respectively, mainly injection site reactions.<br>GMTs (PRNT50) of neutralizing antibodies at day 0 before the vaccination were about 2.5 (95%CI 2.3, 2.7) and 2.2 (2.1, 2.3) in the three-dose regimen cohort, increasing to 197.4 (167.7, 232.4) and 33.6 (28.3, 39.8) at day 14 after receiving Convidecia and CoronaVac, respectively (<0.0001).	The heterologous prime-boost regimen with Convidecia after the priming with CoronaVac was safe and significantly immunogenic than a homogeneous boost with CoronaVac
Dupuis [62]	Phase 2 double blind<br>Single centre at Montreal, Quebec, Canada<br>Feb to May 2021	Non-hospitalized patients with symptomatic and confirmed COVID-19 infection	I: hesperidin 100mg once daily for 14 days (N=107)<br>C: placebo for 14 days (N=109)	Proportion of patients with fever, cough, shortness of breath or anosmia at day 3, 7, 10 and 14.<br>ITT, generalized linear mixed model (binary logistic regression model and Poisson regression model).<br>The proportion for primary outcome in the hesperidin group compared to the placebo group were, 88.5% vs 88.8% (day 1), 91.2% vs 87.4% (day 3), 81.3% vs 75.2% (day 7), 64.4% vs 60.6% (day 10) and 49.4% vs 58.5% (day 14). At 14 days, there was a 9.1% absolute reduction in group A symptoms in the hesperidin group (OR: 0.69, p = 0.2328).	Hesperidin therapy is safe and may help reduce a composite of selected COVID-19 symptoms including fever, cough, shortness of breath and anosmia.
Haran [63]	Open label<br>16 sites in US	Non-hospitalized patients with mild to	I: KB109 (novel synthetic glycan to increase production of	Proportion of patients who had treatment emergent averse events (TEAE)	KB109 was well tolerated ad reduced medically-attended

© 2023 Bai AD et al. *JAMA Network Open*.

	July to Dec 2020	moderate COVID-19	gut microbial metabolites) for 14 days (N=169)<br>C: standard of care for 14 days (N=181)	ITT, descriptive<br>36.1% (61/169) of patients receiving KB109 and SSC experienced ≥1 TEAE compared with 26.5% (48/181) of patients in the SSC-alone group. Five patients receiving KB109 experienced TEAEs that led to discontinuation of KB109; in 2 patients, these TEAEs were related to KB109. Treatment emergent serious AEs were reported in 2 patients receiving KB109 and SSC (hypoxia, pneumonia, and COVID-19; none related to KB109) and 3 patients receiving SSC alone (COVID-19 and pneumonia).	visits especially in high risk population (age >=45 years and >=1 comorbidity).
Song [64]	Single blind<br>Unclear location<br>Unclear date and time	Adult with symptomatic COVID-19 infection.	I: clevudine 120mg PO daily for 14 days (N=41)<br>C: placebo for 14 days (N=20)	Proportion with negative PCR result at day 15.<br>ITT, Chi-square or Fisher's exact test.<br>The proportion of patients with negative test was 10 (25.6%) in the clevudine group and 5 (25%) in the placebo group p=0.9573.	There was no significant difference<br>in the proportion of patients with negative result and the clinical efficacy outcomes compared to placebo.
Rojas-Serrano [65]	Double blind<br>Single centre in Mexico<br>April to July 2020	Healthcare workers caring for severe COVID-19 patients.	I: hydroxychloroquine 200mg PO daily for 60 days (N=62)<br>C: placebo for 60 days (N=65)	Time to symptomatic respiratory infection with positive test for SARS-CoV2 by RT-PCR over 60 days.<br>ITT, Kaplan-Meier survival curves and log-rank test, Cox proportional hazard model<br>COVID-19 infection occurred in 1 (1.6%) in the hydroxychloroquine group and 6 (9.2%) in the placebo group (log rank test p=0.09).	Although the number of symptomatic infections in health personnel was lower in the hydroxychloroquine group, the difference was not statistically significant.
Beltran-Gonzalez [66]	Double blind<br>Single centre in Mexico<br>Aug 2020	Hospitalized patients with moderate COVID-19 infection but no severe respiratory failure.	I 1: hydroxychloroquine 400mg PO twice daily on first ay then 200mg PO twice daily for 4 days (N=33)<br>I 2: ivermectin 12 or 18mg based on weight PO once (N=36)<br>C: placebo (N=37)	Hospital stay until discharge due to patient improvement. Safety outcomes were hospital stay until respiratory deterioration or death.<br>Comparison done by ANOVA or Kruskal-Wallis test. Kaplan Meier curves and log rank test for time to respiratory deterioration or death.<br>No difference in hospitalization duration was found between the<br>treatment groups (hydroxychloroquine: 7 (IQR 3-7) vs ivermectin: 6 (IQR 4-11) vs placebo: 5 (IQR 4-7), p =0.43). Time to death or respiratory deterioration was not different between groups.	In non-critical hospitalized patients with COVID-19 pneumonia, neither ivermectin nor hydroxychloroquine decreases the number of in-hospital days, respiratory deterioration, or deaths.
Gonzalez [67]	Open label<br>May to Oct 2020<br>Single centre in Mexico	Patients with COVID-19 pneumonia that is severe or critical	I 1: IVIG at dose of 0.3g/kg infusion daily for 5 days (N=60)<br>I 2: 200cc of convalescent plasma for 2 days (N=130)	Hospitalization duration and mortality at 28 days.<br>ITT, hospital duration analyzed using Mann-Whitney U test. Survival analysis was conducted with Kaplan Meier curve with comparison between groups by log-rank test.<br>No comparison of hospitalization duration.<br>Mortality was 46.2% in the convalescent plasma group and 43% in IVIG group, log rank test p=0.83.	In critically ill patients with COVID-19 pneumonia, convalescent plasma is not superior to IVIG in terms of hospitalization duration or mortality.
Figueroa [68]	Double blind<br>10 hospitals in Argentina	Healthy hospital personnel dedicated	I: iota-carrageenan nasal spray 4 times daily for 21 days (N=196)	Clinical COVID-19 infection confirmed by PCR in period of 21 days.	Iota-carrageenan nasal spray significantly reduced SARS-CoV2 infection in hospital

© 2023 Bai AD et al. *JAMA Network Open*.

	July to Dec 2020	to care of COVID-19 patients	C: placebo nasal spray for 21 days (N=198)	ITT, relative risk reduction, proportion compared with chi-square or Fisher's test.<br>Clinical COVID-19 infection occurred in 2/196 (1%) in the Iota-Carrageenan group and 10/198 (5%) with odds ratio of 0.19 95% CI 0.05 to 0.77 p=0.03. Risk reduction was 95% (95% CI 6% to 99.7% p=0.04).	personnel dedicate to care of patients with COVID-19 infection.
Chew [69]	Phase 2 double blinded platform trial<br>38 sites in US<br>Aug to Nov 2020	Non-hospitalized adults with symptomatic COVID-19 infection.	I 1: bamlanivimab 7000mg IV once (N=48)<br>I 2: bamlanivimab 700mg IV once (N=111)<br>C: placebo (N=158)	Detection of SARS-CoV2 RNA on nasopharyngeal swab, improvement of 13 targeted COVID-19 symptoms and grade >=3 treatment emergent adverse events through day 28<br>Modified ITT. Adverse events compare using log-binomial regression and summarize with risk ratio. Proportion of undetectable SARS-CoV-2 RNA compared using Poisson regression and summarized with RR. Participant specific symptom durations from ay 0 to 28 compared using Wilcoxon rank sum test.<br>Proportion of undetectable NP for SARS-CoV-2 RNA (risk ratio compared to placebo, 0.82-1.05 for 7000 mg dose [overall p=0.88] and 61 0.81-1.21 for 700 mg dose [overall p=0.49]. Time to symptom improvement was median of 21 vs 18.5 days, p=0.97, for 7000 mg bamlanivimab vs placebo and 24 vs 20.5 days, p=0.08, for 700mg bamlanivimab vs placebo. Grade >=3 adverse events I not differ between groups.	Treatment with bamlanivimab 7000 mg and 700 mg was safe. There was no improvement was observed with bamlanivimab in the primary outcomes of proportion of participants with undetectable SARS<br>CoV-2 RNA from NP swabs or time to improvement of COVID-19-related symptoms.
Winthrop [70]	Phase 2 double blind<br>8 sites in US<br>July to Dec 2020	Adult with COVID-19 infection confirmed by PCR testing and pneumonia on chest X-ray.	I: Opaganib PO twice daily for 14 days (N=23)<br>C: placebo for 14 days (N=19)	Supplemental oxygen requirement from baseline to day 14 based on area under the curve.<br>ITT, descriptive<br>The Kaplan-Meier analysis for no longer requiring supplemental oxygen for at least 24 hours by Day 14 showed a higher estimated cumulative incidence in patients on opaganib (50%)<br>compared to patients on placebo (22.2%). The relative benefit derived for each group was calculated as 61.6% (-770/-1250) for<br>opaganib (n=21) and 46.7% (-583.6/-1250) for placebo (n=18)	Results demonstrated clinical improvement as measured by reduced need for supplemental oxygen, improved WHO level in the scale for clinical improvement and in time to discharge. The safety profile of opaganib was not materially different than that of placebo.
Wanaratna [71]	Double blind<br>2 sites in Thailand<br>Dec 2020 to March 2021	Adults with laboratory confirmed COVID-19 infection and mild symptoms	I: Andrographis paniculate extract (APE) 60mg PO 3 times daily for 5 days (N=29)<br>C: placebo (N=28)	Occurrence of pneumonia by day 5, detection of SARS-CoV2 by day 5.<br>PP, Comparison by Student t-test for continuous data and chi-square for categorical data.<br>Pneumonia occurred in 0/29 (0%) in APE group and 3/28 (10.7%) in placebo group (p-0.039).<br>SARS-CoV 2 was detected in 10/29 (34.5%) in the APE group and 16/28 (57.1%) in the placebo group (p=0.086).	APE is potentially effective and safe in adults with mild COVID-19 infection.

© 2023 Bai AD et al. *JAMA Network Open*.

Damle [72]	Phase 1 & 2, single blinded<br>2 sites in India<br>March to June 2021	Phase 1: health volunteers<br>Phase 2: COVID-19 patients	Phase 2<br>I: plant formulation ATRICOV 452 for 14 days (N=50)<br>C: placebo for 14 days (N=50)	Time to resolution of symptoms. Change in inflammatory markers in bloodwork (NLR, CRP, ferritin, D-dimer, IL-6 and PCT).<br>PP, statistical method not described.<br>Time to resolution of symptoms in patients assigned to ATRICOV 452 vs placebo was not significantly different (median, 6 days vs 7 days). The decrease in NLR, CRP, ferritin, D-dimer, IL-6 and PCT levels in ATRICOV 452 group, which was better than the placebo group, though no p-value or statistical comparison described.	ATRICOV I safe and resulted in faster improvement of inflammatory markers in COVID-19 infection.
Huang [73]	Evaluator blinded<br>Single centre in China<br>Feb to April 2020	Patients with moderate to severe COVID-19 infection and acute respiratory syndrome.	I: Ultra-short wave diathermy (USWD) to anterior a posterior of trunk for 10 minutes twice daily for 12 days (N=25)<br>C: Standard of care (N=25)	Length of recovery from symptoms measured by 7-category ordinal scale, SIRS scale and negative conversion rate of SARS-CoV-2 by PCR at 7, 14, 21 and 28 days.<br>T-test for length of recovery. Chi-square for SIRS scale and conversion of PCR result.<br>The time to clinical recovery (days) in the USWD group was significantly shortened than the control group (36.84±9.93 vs 43.56±12.15, P = 0.037). The SARS-CoV-2 nucleic acid test negative conversion rate showed no significant difference between the USWD and control group at day 7 (P = 0.054), day14 (P = 0.239), day 21 (P = 0.279), and day 28 (P = 0.730). SIRS scale showed a statistically significant difference between the two groups at day 7 (P= 0.030), day14 (P = 0.002), day 21 (P =0.003), and day 28 (P = 0.011).	USWD could shorten recovery course and enhance clinical improvement without aggravating pulmonary fibrosis.
Dunkle [74]	Phase 3, observer blinded<br>113 sites in US and Mexico<br>Dec 2020 to Feb 2021	Healthy adults or those with stable chronic medical conditions.	I: 2 doses of NVX-CoV2373 21 days apart (N=19,714)<br>C: 2 doses of placebo 21 days apart (N=9,868)	Vaccine efficacy against PCR confirmed COVID-19 infection >=7 days after second dose administration<br>PP, vaccine efficacy as (1 – relative risk) with CI estimated by Poisson regression with robust error variance<br>There were 14 COVID-19 cases in the vaccine group and 63 COVID-19 cases in the placebo group. The vaccine efficacy was 90.4% 95% CI 82.9% to 94.6% P<0.001). The vaccine efficacy was 100% (95% CI 87% to 100%) against moderate to severe disease.	NVX-CoV2373 was well tolerated and demonstrated a high overall VE (>90%) for prevention of Covid-19, with most cases due to variant strains.
Maskin [75]	Open label<br>4 ICUs in Argentina<br>June 2020 to March 2021	Patients with acute respiratory distress syndrome due to COVID-19	I: dexamethasone 16mg/day IV for 5 days then 8mg/day for 5 days (N=49)<br>C: dexamethasone 6mg/day IV for 10 days (N=51)	Ventilator free days during first 28 days. Time to complete and successfully discontinue mechanical ventilation or death.<br>Modified ITT. Wilcoxon rank sum test with basic bootstrap. Competing risk regression model.<br>The ventilator free days was 0 (IQR 0-14) day in high dose group and 0 (IQR 0-1) day in low dose group (P=0.231). The hazard ratio of successful	Among patients with C-ARDS, the use of higher doses of dexamethasone compared with the recommended low-dose treatment did not show an increase in VFD. However, the higher dose significantly improved the time

© 2023 Bai AD et al. *JAMA Network Open*.

				discontinuation from mechanical ventilation was 1.84 (95% CI 1.31 to 2.5 P<0.001).	required to liberate them from the ventilator.
Mammen [76]	Phase 2, double blinded<br>16 locations in US<br>Nov 2020 to Feb 2021	Healthy adults with high risk of exposure to SARS-CoV-2	I 1: DNA vaccine targeting full length spike antigen (INO-4800) 1mg dose followed by electroporation on day 0 and 28 (N=151)<br>I 2: INO-4800 2mg dose followed by electroporation on day 0 and 28 (=147)<br>C 1: placebo 1 injection followed by electroporation on day 0 and 28 (N=50)<br>C 2: placebo 2 injections followed by electroporation on day 0 and 28 (N=51)	Immunogenicity based on antigen-specific cellular immune response measured by IFNy ELISpot assay and neutralizing antibody response as measured by pseudovirus based neutralization assay at week 6.<br>Descriptive, ELISpot compared by differences, neutralizing antibody titer compared using ratios of geometric mean fold rises and t-distribution 95% CIs.<br>At week 6, the geometric mean titers (GMT) (SD of log10) of binding antibody in the 1.0 mg and 2.0 mg dose groups were 938.8 (0.76) and 2210.0 (0.75)<br>Units/ml (U/ml), respectively, compared with baseline GMT (SD) of 123.3 (0.68) and 93.5 (0.48)<br>U/ml, respectively; the GMT (SD) of binding antibody in the 1- and 2-injection placebo groups at<br>this timepoint were 92.8 (0.43) and 145.6 (0.54) U/ml, respectively, compared with baseline GMT (SD) of 110.2 (0.51) and 123.8 (0.52) U/ml, respectively. Sera were also tested for the ability to neutralize SARS-CoV-2-DeltaCT pseudovirus. At week 6, the geometric mean titers (GMT) (SD of log10) of neutralizing antibody in the 1.0 mg and 2.0 mg<br>dose groups were 93.6 (0.47) and 150.6 (0.46), respectively, compared with baseline GMT (SD) of 32.2 (0.38) and 35.8 (0.45), respectively. ELISpot assay showed median increase of 0 for both placebo groups. In In the INO-4800 vaccinated groups, the median (min<br>– max) increase from baseline to Week 6 was 3.40 (0.0 – 90.0) SFU per 106 PBMC in the<br>1.0 mg dose group and 12.75 (0.0 – 465.0) SFU per 106 PBMC in the 2.0 mg dose group.	INO-4800 at both the 1.0 mg and 2.0 mg doses when administered in a 2-dose regimen appeared to be safe and well-tolerated in all adult ages. However, the comparative immunogenicity analysis favored selection of INO-4800 2.0 mg dose for advancement into a Phase 3 efficacy evaluation.
Silveira [77]	Open label<br>Single centre in Brazil<br>June to Aug 2020	Adult patients hospitalized for COVID-19 infection.	I 1: standardized propolis product 400mg/day PO for 7 days (N=40)<br>I 2: standardized propolis product 800mg/day PO for 7 days (N=42)<br>C: standard of care (N=42)	Time to clinical improvement defined as length of hospital stay or oxygen therapy dependency by day 28<br>ITT, mean difference by generalized linear model<br>Mean difference low dose vs. control was -3 days (95% CI -6.2 to -0.07 p=0.049) and high dose vs. control was -3.9 days (95% CI -7.0 to -1.09 p=0.009). The mean difference in oxygen was not significant for low dose (-2 days 95% CI -7.8 to 3.6 days p=0.470) or high dose (-0.99 days 95% CI -6.1 to 4.1 days p=0.710).	Propolis for 7 days was safe and reduced hospital stay.

© 2023 Bai AD et al. *JAMA Network Open*.

Toledo-Romani [78]	Phase 1/2, open label<br>Single site in Cuba<br>Nov 2020 to Jan 2021	Healthy volunteers	Phase 1<br>I 1: SOBERANA vaccine (RBD conjugated to carrier protein tetanus toxoid) 15ug intramuscular on day 0 and 28 (N=20), half received 3rd dose on day 56<br>I 2: SOBERANA vaccine 25ug intramuscular on day 0 and 28 (N=20), half received 3rd dose on day 56<br>Phase 2<br>I 1: SOBERANA vaccine 25ug intramuscular on day 0, 28 and 56 (N=50)<br>I 2: SOBERANA vaccine 25ug intramuscular on day 0 and 28 (N=50)	Occurrence of vaccine related serious adverse events during 28 days after each dose.<br>Descriptive only with frequency, percentage and confidence interval<br>No vaccine-related serious event occurred in phase 1 or 2 of the trial.	SOBERANA vaccine is safe and immunogenic. Highest immune response were elicited with the heterologous 3 doses schedule.
Toledo-Romani [79]	Phase 3, double blind<br>48 sites in Cuba<br>March 2021	Healthy volunteers	I 1: SOBERANA 02 for 2 doses (N=14,679)<br>I 2: SOBERANA 02 for 3 doses followed by SOBERANA plus ((N=14,677)<br>C: placebo (N=14,675)	Vaccine efficacy in terms of confirmed symptomatic COVID-19 infection 14 days after lats injection.<br>PP, efficacy defined as 1 – hazard ratio from Cox proportional hazards model.<br>Symptomatic COVID-19 infection occurred in 43/14,371 in 2-dose group vs. 155/14,403 in placebo group with vaccine efficacy of 71% (95% CI 58.9%-79.1%). Symptomatic COVID-19 infection occurred in 15/13,833 in the 3-dose group with vaccine efficacy of 92.4% (95% CI 86/9%-95.6%).	Two doses of SOBERANA 02 were safe and attained efficacy of 71.0% in the adult population 19-80 y/o; incorporating SOBERANA Plus increased efficacy from 71.0% to 92.4%.
Tsilika [80]	Phase 3, double blind<br>11 internal medicine departments in Greece<br>June 2020 to April 2021	Adults age 50 years or more with comorbidities with negative serology for COVID-19.	I: BCG revaccination intradermal once (N=148)<br>C: placebo (NaCl) intradermal once (N=152)	Incidence of COVID-19 infection in first 90 days.<br>Chi-square test, logistic regression analysis<br>At 90 days, 2 patients in BCG group and 10 patients in the placebo group had COVID-19 (p=0.086). At 6 months, the multivariate analysis showed a OR of 0.32 (95% CI 0.13 to 0.79, p=0.014).	These data argue that BCG revaccination is safe and protects the elderly against COVID19.
Holubar [81]	Phase 2 double blind<br>Stanford healthcare in US<br>July 2020 to March 2021	Patients with asymptomatic or uncomplicated COVID-19 infection confirmed by PCR.	I: favipiravir 1800mg PO twice daily on ay 1, then 800mg PO twice daily on day 2 to 10 (N=65)<br>C: placebo for 10 days (N=70)	SARS-CoV-2 shedding cessation<br>Modified ITT, Cox proportional hazard model<br>There was no difference in time to shedding cessation (hazard ratio 0.76 favoring placebo 95% CI 0.48 to 1.20).	The results from this trial do not support favipiravir use at commonly used doses in outpatients with uncomplicated COVID-19 infection.
O'Donnell [82]	Phase 2 double blind<br>5 hospitals in US and Brazil<br>April to Nov 2020	Adults hospitalized for COVID-19 infection with hypoxemia.	I: convalescent plasma 1 unit IV (N=150)<br>C: control normal plasma 1 unit IV (N=73)	Clinical status based on ordinal scale at 28 days<br>ITT, Mann-Whitney test, proportional odds model<br>Odds ratio of 1 point improvement in scale 1.5 favoring convalescent plasma 95% CI 0.83 to 2.68 P=0.18.	In adults hospitalized with severe COVID-19, use of convalescent plasma was not associated with significant improvement in 28 days clinical status. The significant reduction in

© 2023 Bai AD et al. *JAMA Network Open*.

					mortality associated with convalescent plasma, however, may warrant further evaluation.
O'Brien [83]	Phase 3, double blind<br>112 sites in US, Romania and Moldova<br>Jan to March 2021	Individuals >12 years within 96 hours of a household contact diagnose with COVID-19 infection.	I: REGEN-COV 1200mg subcutaneous injection once (N=753)<br>C: placebo subcutaneous injection once (N=752)	Proportion of participants without evidence of infection or prior immunity who subsequently developed symptomatic SARS-CoV-2 infection within 28 days.<br>Logistic regression<br>11/753 (1.5%) in the REGEN-COV and 59/752 (7.8%) had symptomatic COVID-1i infection with 81.4% risk reduction and OR 0.17 P<0.0001.	Administration of subcutaneous REGEN-COV prevented symptomatic Covid-19 and asymptomatic SARS-CoV-2 infection in uninfected household contacts of infected individuals.
O'Brien [84]	Phase 3, double blind<br>112 sites in US, Romania and Moldova<br>Jan to March 2021	Asymptomatic adults with positive test for SARS-CoV-2	I: REGEN-COV 1200mg subcutaneous injection once (N=155)<br>C: placebo subcutaneous injection once (N=156)	Proportion of participants without evidence of infection or prior immunity who subsequently developed symptomatic SARS-CoV-2 infection within 14 days.<br>Not described.<br>Symptomatic COVID-19 infection occurred in 29/100 (29%) in REGEN-COV group and 44/104 (42.3%) in placebo group with relative risk reduction of 31.5% P=0.038.	Subcutaneous REGEN-COV prevented progression from asymptomatic to symptomatic infection, reduced duration of high viral load and symptom.
Puskarich [85]	Blinded trial<br>13 hospitals in US<br>April 2020 to Feb 2021	Hospitalized patients with COVID-19 infection and respiratory SOFA score of at least 1 not on RAAS inhibitor.	I: losartan 20mg PO twice daily for 10 days (N=101)<br>C: placebo for 10 days (N=104)	Imputed O1O2/FiO2 ratio at 7 days.<br>ITT, predictive modeling, Welch's t-test.<br>Difference in PaO2/FiO2 ratio was -24.8 (95% CI -55.6 to 6.1; p=0.12) favoring placebo.	Losartan did not improve PaO2/FiO2 ratio at 7 days in COVID-19 and acute lung injury.
Bonelli [86]	Blinded<br>Single centre in Austria<br>May to Aug 2021	Patients on rituximab treatment who did not seroconvert after primary mRNA vaccination.	I: vector vaccine as 3rd booster, which is the heterologous group (N=27)<br>C: same mRNA vaccine as 3rd booster, which (N=28)	SARS-CoV-2 seroconversion at week 4<br>Chi-square test<br>Seroconversion rate was 6/27 (22%) in the heterologous and 9/28 (32%) in the homologous group (P=0.6).	The additional SARS-CoV-2 booster vaccination evaluated in this trial resulted in the development of a humoral response in 27% of the population regardless of heterologous or homologous booster.
Sholzberg [87]	Open label<br>28 sites in 6 countries<br>May 2020 to April 2021	Moderately ill hospitalized ward patients with COVID-19 and elevated D-dimer	I: therapeutic heparin (N=228)<br>C: prophylactic heparin (N=237)	Composite of death, invasive mechanical ventilation, non-invasive mechanical ventilation or ICU admission.<br>ITT, logistic regression, Chi-square test<br>Composite outcome occurred in 37/228 (16.2%) in therapeutic heparin group and 52/237 (21.9%) in prophylactic heparin group (odds ratio 0.69 95% CI 0.43 to 1.10; P=0.12)	In moderately ill ward patients with Covid-19 and elevated D-dimer level, therapeutic heparin did not significantly reduce the primary outcome but decreased the odds of death at 28 days.
Li [88]	Phase 2, double blind<br>Single centre in China<br>May 2020 to April 2021	Healthy adults >=60 years	I 1: 1.5ug 3rd dose booster (N=85)<br>I 2: 3ug 3rd dose booster (N=90)<br>I 3: 6ug 3rd dose booster (N=81)	Antibody titer level after 3rd dose. Vaccine related adverse event within 28 days after 3rd dose administration.	Neutralizing antibody titres declined substantially six months after two doses of CoronaVac among older

© 2023 Bai AD et al. *JAMA Network Open*.

			C: placebo (N=47)	PP, ANOVA was used to compare log-transformed antibody titres. Chi-square test or Fisher's exact test for categorical outcomes.<br>Antibody levels (GMT) increased across all vaccine groups. Adverse reactions were grade 1 or 2 in all vaccine groups. None of the serious adverse events were related to vaccine.	adults. A booster dose rapidly induces robust immune responses.
Elgohary [89]	Single blind (patient)<br>Centre in Egypt<br>April to June 2020	Patients with moderate COVID-19 pneumonia.	I: sofosbuvir/ledipasvir PO once daily for 15 days (N=125)<br>C: oseltamivir 150mg PO twice daily for 10 days, hydroxychloroquine 400mg PO twice daily for 1 ay then 200mg twice daily for 9 days and azithromycin 500mg PO once then 250mg PO daily for 6 days (N=125)	Cure rate over time, hospital stay and incidence of serious adverse events within 15 days.<br>Survival analysis and log-rank test for cure rate over time. Cox regression model. Chi-square for categorical outcomes and T-test for continuous outcomes.<br>90 (72%) in experimental group and 50 (40%) in control group were cured. Adjusted relative risk was 2.3 95% CI 1.54 to 3.5. Median length of stay was 16 in the experimental group and 24 in the control group. All 6 deaths occurred in control group (P=0.013). 7 (5.6%) in the experimental group and 6 (4.8%) in the control group were admitted to ICU (P=0.776).	Sofosbuvir/ledipasvir is effective in treating moderate COVID-19 infection.
Javaherian [90]	Single blind (assessor)<br>Single centre in Iran<br>April 2020	Hospitalized non-intubated patients with oxygen saturation in free air breathing <=90% and COVID-19 pneumonia	I: 6 sessions of pulmonary physiotherapy with breathing exercises and airway clearance techniques<br>C: basic care	Venous blood O2(pO2) and CO2(pCO2) pressures, Spo2, and three minute walking test(3MWT) that were assessed before and end of sixth session<br>ITT, ANCOVA for continuous variables, Chi-square test for categorical variables<br>In the crude model analysis, the mean pO2 (MD: 5.81 [1.51, 10.11], SDM: .9 [.21, 1.57]), Spo2 (MD: 3.99 [1.5, 6.47], SMD: 1.07 [.37, 1.76]), 3MWT distance (MD: 81.82 [47.93, 115.7], SMD: 1.61 [.86, 2.35]), level of dyspnea (MD: -2.08 [-2.96, -1.19], SMD: -1.57 [-2.31, -.82]), RPE after walking (MD: -4.04 [-5.76, -2.32], SMD: -1.56 [-2.3, -.82]) and, PH (MD: .06, [.01, .12], SMD: .74 [.71, 1.41]) were statistically significant different between groups in all analysis models (P ≤ .03). Adjusted analysis in Table 2.	Early pulmonary physiotherapy is a safe and effective therapeutic choice.
Formica [91]	Phase 2 observer blinded<br>17 sites in Australia and US<br>Aug to Sept 2020	Healthy adult age 18 to 84 years	I 1: NVX-CoV2373 5ug intramuscular for 1 dose (N=256)<br>I2: NVX-CoV2373 5ug intramuscular for 2 doses 21 days apart (N=258)<br>I 3: NVX-CoV2373 25ug intramuscular for 1 dose (N=255)	IgG anti-spike protein response at day 35, solicited reactogenicity in 7 days and unsolicited adverse events<br>Descriptive with 95% CI<br>Local reactogenicity more predominant in vaccine groups, frequently tenderness and pain. Solicited systemic adverse events other than muscle pain were reported similarly across all groups. Unsolicited adverse events occurred similarly across groups. IgG geometric mean titers at ay 35 were 44,421 [95% CI, 37,929 to	Two-dose regimen of 5-μg NVX-CoV2373 is highly immunogenic and well tolerated in both younger and older participants.

© 2023 Bai AD et al. *JAMA Network Open*.

			I 4: NVX-CoV2373 25ug intramuscular for 2 doses 21 days apart (N=259)<br>C: placebo for 2 doses (N=255)	52,024) and 46,459 (95% CI, 40,839 to 52,853) for the two-dose regimens of 5-μg and 25-μg NVX-CoV2373, respectively. Seroconversion were 98% and 100% compared to 1.3% in placebo group.	
Morici [92]	Open label<br>9 sites in Italy<br>April 2020 to April 2021	Adult patients admitted to medical ward with PCR confirmed COVID-19 infection.	I: enoxaparin 40mg SC twice daily until hospital discharge (N=91)<br>C: enoxaparin 40mg SC once daily until hospital discharge (N=92)	In-hospital incidence of venous thromboembolism (VTE).<br>ITT, absolute risk reduction and risk ratio with 95% CI.<br>VTE occurred in 0 (0%) in the twice daily group and 6 (6.2%) in the once daily group with absolute risk reduction of 6.5 95% CI 1.5 to 11.6.	VTE occurred less frequently in the twice daily group compared to once daily group.
Holubovska [93]	Phase 3, double blind<br>15 sites in Ukraine<br>Unclear date	Adult patients hospitalized for COVID-19 infection	I: enisamium 500mg 4 times daily for 7 days<br>C: placebo for 7 days	Time to improvement of WHO severity ordinal rating scale by 2 points.<br>ITT, log rank test<br>The interim analysis showed no difference in time-to-recovery and median time–to-recovery between the two groups.	Enisamium improved the recovery of COVID-19 needing supplementary oxygen (SR 4) by more than 2 days and could be used a viable therapeutic option.
Babalola [94]	Double blind<br>Single centre in Nigeria<br>May to Nov 2020	Patients with PCR confirmed COVID-19 infection who were either asymptomatic or had mild to moderate symptoms.	I 1: ivermectin 6mg IV every 84 hours twice a week for 2 weeks (N=21)<br>I 2: ivermectin 12mg IV every 84 hours for 2 weeks (N=21)<br>C: lopinavir/ritonavir daily for 2 weeks (N=20)	Time to SARS-CoV-2 negativity based on PCR testing.<br>Student's T-test, survival analysis with log rank test and Cox proportional hazard.<br>Mean days to negative was 9.15 (95% CI 5.68 to 12.62) in control arm and 5.33 (95% CI 4.33 to 6.32) in ivermectin arms (P=0.0066). Mean days to negative was shortened by 4.5 days in the 12mg arm and 3.15 days in the 6mg arm (P=0.0179).	Ivermectin 12mg regimen have superior efficacy for treatment of COVID-19.
Bhardwaj [95]	Open label<br>Single centre in India<br>Unclear time frame	Patients with mild COVID-19 infection.	I: AYUSH 64 1000mg PO 3 times daily for 7 days (N=30)<br>C: standard of care (N=30)	Negative PCR on 5th day<br>Fisher's exact test<br>On day 5, 21 (70%) in the AYUSH 64 group and 16 (54%) in the control group tested negative (P=0.28).	Conversion of RT-PCR from positive to negative was not statistically different between AYUSH 64 and control group
ATTACC investigators [96]	Open label adaptive trial<br>International trial<br>April 2020 to Jan 2021	Non-critically ill patients hospitalized for COVI-19.	I: therapeutic dose anticoagulation with heparin for 14 days or until recovery (N=1181)<br>C: usual care pharmacological thromboprophylaxis for 14 days or until recovery (N=1050)	Organ support free days, an ordinal outcome<br>Bayesian cumulative logistic model<br>Probability that therapeutic anticoagulation increased organ support-free days compared to thromboprophylaxis was 99.0% (adjusted odds ratio 1.29, 95% credible interval 1.04 to 1.61). The adjusted absolute increase in survival to hospital discharge without organ support with therapeutic-dose anticoagulation was 4.6% (95% credible interval 0.7 to 8.1).	In non-critically ill patients with Covid-19, an initial strategy of therapeutic-dose anticoagulation with heparin increases the probability of survival to hospital discharge with reduced use of organ support.
Goepfert [97]	Phase 1/2 double blind<br>10 centres in US	Healthy seronegative adults	I: stabilised prefusion S (preS) protein vaccine CoV2 preS dTM<br>C: placebo<br>Randomization to 11 different treatment groups to receive one of 5 candidate vaccine	Adverse events (solicited and unsolicited local and systemic). Neutralizing capacity of vaccine induced antibodies.<br>Modified ITT, descriptive with 95% CI.<br>Adjuvanted vaccine groups had more frequent injection reactions and systemic reactions especially second doses. There were 3 serious	Vaccine was safe. In general, 2 doses of adjuvanted vaccine were needed to generate a meaningful antibody response.

© 2023 Bai AD et al. *JAMA Network Open*.

			formulations or placebo as a single or 2-dose schedule.<br>N=269 in 2 dose cohort<br>N=170 in single dose cohort	averse events, none were related to vaccine. Neutralising antibody GMT increased by day 36. GMT did not differ form placebo after 2 doses. Adjuvanted vaccine yielded 3-4 fold higher GMT.	
Gutierrez-Castrellon [98]	Quadruple blinded<br>Single centre at Mexico<br>Aug to Dec 2020	Symptomatic outpatients with COVID-19 infection	I: probiotic PO once daily for 30 days (N=150)<br>C: placebo PO daily for 30 days (N=150)	Proportion in complete remission by day 30 or progressing to moderate or severe disease requiring hospitalization. Death and ICU admission.<br>ITT, Chi-square and binomial logistic regression.<br>Remission occurred in 78/147 (53.1%) in probiotic group and 41/146 (28/1%) in placebo (P<0.0001, ARR 25% 95% CI 14.1 to 35.9%).<br>No hospitalization or death occurred during the study.	Probiotics improved remission rate for outpatients with COVID-19 infection.
RECOVERY Collaborative Group [99]	Open label platform trial with factorial design<br>177 hospitals in UK, 2 hospitals in Indonesia, 2 hospitals in Nepal<br>Nov 2020 to March 2021	Patients hospitalized with COVID-19	I: aspirin 150mg PO once daily until discharge (N=7351)<br>C: standard of care alone (N=7541)	28 day mortality<br>ITT, log-rank test, RR<br>1222/7351 (17%) in aspirin group and 1299/7541 (17%) in usual care group died (RR 0.96 95% CI 0.89 to 1.04 P=0.35).	Aspirin was not associated with reduction in 28 day mortality or risk of progressing to invasive mechanical ventilation or death.
RECOVERY Collaborative Group [100]	Open label platform trial with factorial design<br>177 hospitals in UK<br>May 2020 to Jan 2021	Patients hospitalized with COVID-19	I: convalescent plasma for 2 days (N=5795)<br>C: usual care (N=5763)	28-day mortality<br>ITT, log-rank test, RR<br>1398/5795 (24%) in convalescent plasma group and 1408/5763 (24%) in usual care group died (RR 1.0 95% CI 0.93 to 1.07 P=0.93)	Among patients hospitalised with COVID-19, high-titre convalescent plasma did not improve survival or other prespecified clinical outcomes.
RECOVERY Collaborative Group [101]	Open label platform trial with factorial design<br>127 sites in UK<br>Sept 2020 to May 2021	Patients hospitalized with COVID-19	I: REGEN-COV (casirivimab 4g an imevimab 4g) IV infusion (N=4839)<br>C: usual care (N=4946)	28-day mortality<br>ITT, log-rank test, RR<br>In all randomized patients, 944/4839 (20%) treated with REGEN-COV and 1026 (21%) treated with usual care die (RR 0.94 95% CI 0.86 to 1.03 P=0.17). In seronegative patients, 396/1633 (24%) treated with REGEN-COV and 451/1520 (30%) treated with usual care died (RR 0.80 95% CI 0.70 to 0.91 P=0.0010).	In patients hospitalised with COVID-19, the monoclonal antibody combination of casirivimab and imdevimab (REGEN-COV) reduced 28-day mortality among patients who were seronegative at baseline.
RECOVERY Collaborative Group [102]	Open label platform trial with factorial design<br>177 hospitals in UK, 2 hospitals in Indonesia, 2 hospitals in Nepal<br>Nov 2020 to March 2021	Patients hospitalized with COVID-19	I: colchicine 1mg PO then 500mcg PO twice daily for 10 days or until discharge (N=5610)<br>C: usual care (N=5730)	28 day mortality<br>ITT, log-rank test, RR<br>1173 (21%) in colchicine group and 1190 (21%) in usual care died (rate ratio 1.01 95% CI 0.93 to 1.10 P=0.77).	In adults hospitalised with COVID-19, colchicine was not associated with reductions in 28-day mortality, duration of hospital stay, or risk of progressing to invasive mechanical ventilation or death.
RECOVERY Collaborative Group [103]	Open label platform trial with factorial design<br>131 hospitals in UK	Patients hospitalized with COVID-19 who had progression (oxygen	I: toxilizumab IV based on body weight for up to 2 doses 12-24 hours apart (N=2022)<br>C: usual care (N=2094)	28 day mortality<br>ITT, log-rank test, RR	In hospitalised COVID-19 patients with hypoxia and systemic 53 inflammation,

© 2023 Bai AD et al. *JAMA Network Open*.

	April 2020 to Jan 2021	saturation <92% on room air or on oxygen therapy an CRP >=75mg/L)		596/2022 (29%) in tocilizumab group and 694/2094 (33%) in usual care group die (RR 0.86 95% CI 0.77 to 0.96 P=0.007).	tocilizumab improved survival and other clinical outcomes.
Gobeil [104]	Phase 2, observer blinded, placebo controlled<br>Sites in Canada and US<br>Ended in April 2021	Healthy adults	I: CoVLP + AS03 vaccine for 2 doses (N=494)<br>C: placebo vaccine for 2 doses (N=94)	Safety, tolerability and immunogenicity up to 42 days.<br>ITT, GMT analyzed by ANOVA, Fisher's exact test for seroconversion.<br>Majority of adverse events are mild or moderate and transient. No grade 4 adverse events were reported. There was significant increase in GMT and IFN-y response at 21 days after first dose and further increase 21 days after second dose. There were little difference in GMT between age groups after 2nd dose.	Vaccine was well tolerated and had milder reactogenicity in older adults. It produced a robust immune response after 2 doses regardless of age.
Begin [105]	Open label<br>72 sites in Canada, US and Brazil<br>May 2020 to Jan 2021	Adults with COVID-19 receiving oxygen within 12 days of symptom onset	I: convalescent plasma 1 or 2 units from 1 or 2 doors (N=625)<br>C: standard of care (N=313)	Composite outcome of intubation or death by 30 days.<br>ITT, RR, logistic regression<br>Composite outcome occurred in 199/614 (32.4%) in the convalescent plasma arm ad 86/307 (28.0%) in the standard of care arm with RR of 1.16 (95% CI 0.94 to 1.43 P=0.18).	Convalescent plasma did not reduce the risk of intubation or death at 30 days among hospitalized patients with COVID-19.
Mallory [106]	Phase 2, placebo controlled, observer blinded<br>17 sites in Australia and US<br>Unclear timeline	Healthy adults who already participated in the original trial of primary series vaccine	I: NVX-CoV2373 single booster after completing primary vaccination series of 2 doses of the same vaccine (N=105)<br>C 1: placebo after completing primary vaccination series of 2 doses of the same vaccine (N=102)<br>C 2: placebo for 3 doses (N=172)	Safety and immunogenicity parameters<br>Modified ITT for safety analysis, PP for immunogenicity analysis, descriptive only<br>Solicited local and systemic reactogenicity increase after each dose. Grade 4 local reactions occurred in 2 patients in the booster group. There were no serious adverse events related to vaccine. After booster, IgG and MN50 increased robustly.	Administration of a single booster dose of the vaccine approximately 6 months following the primary two-dose series resulted in an incremental increase in reactogenicity events along with significantly enhanced immunogenicity.
Ella [107]	Phase 3, double blind<br>25 hospitals in India<br>Nov 2020 to Jan 2021	Healthy adults	I: inactivated vaccine BBV152 2 intramuscular doses 4 weeks apart (N= 12,221)<br>C: placebo 2 intramuscular doses 4 weeks apart (N=12,198)	Laboratory confirmed symptomatic COVID-19 infection occurring at least 14 days after seco dose<br>PP, vaccine efficacy based on per-time incidence rates<br>COVID-19 symptomatic infection occurred in 24 cases in the vaccine group and 106 cases in the placebo group. Overall vaccine efficacy was 77.8% (95% CI 65.2% to 86.4%).<br>Severe COVID-19 infection occurred in 1 patient in the vaccine group and 15 in the placebo group, giving vaccine efficacy of 93.4% (95% CI 57.1% to 99.8%)	BBV152 was immunogenic and highly efficacious against symptomatic and asymptomatic COVID-19 variant associated disease, particularly against severe disease in adults.
Lazarus [108]	Double blind<br>UK<br>Dec 2020 to Jan 2021	Healthy volunteers aged 18 to 55 years	I 1: VLA 2001 vaccine low dose (3AU) on day 0 and 21 (N=51)	Solicited adverse reactions 7 days after vaccination and neutralizing antibody GMT 2 weeks after second vaccination. Seroconverion based on 4 fold increase in GMT.	VLA2001-201 was well tolerated and produced both humoral and cellular immune

© 2023 Bai AD et al. *JAMA Network Open*.

			I 2: VLA 2001 vaccine medium dose (7AU) on day 0 and 21 (N=51)<br>I 3: VLA 2001 vaccine high dose (35AU) on day 0 and 21 (N=51)	Modified ITT for safety and PP for immunogenicity. Adverse events descriptive and compared with Fisher-Freeman-Halton exact test. GMTs were compared by Kruskal-Wallis test.<br>Solicited reactions were similar between dose groups. GMT reached 520 (95% CI 421 to 667) in high dose group, 222 (95% CI 172 to 288) in medium dose and 161 (95% CI 121 to 214) in low dose group.<br>Seroconversion occurred in 100% (95% CI 93%, 100%) in the high dose group, 89% (95%CI 78%, 97%) in the<br>medium dose group and 84% (95%CI 71%, 93%) in the low dose group.	responses, with a clear dose-response effect.
Amaravadi [109]	Double blinded<br>Single centre in US<br>April to July 2020	Outpatients with symptomatic COVID-19 and age of 40 years or older	I: hydroxychloroquine 400mg PO twice daily for 14 days (N=17)<br>C: placebo PO twice daily for 14 days (N=17)	Time to release from quarantine<br>PP, Cox regression and one sided z-test on hazard ratio.<br>Median time to release from quarantine was 8 (range 4-19) days in hydroxychloroquine group and 11 (4-18) days in placebo group with hazard ratio of 1.10 (95% CI 0.51 to 2.34 P=0.28)	Since this study was terminated early due to a lack of feasibility, no conclusion can be made about the efficacy of hydroxychloroquine.
Ravikirti [110]	Double blind<br>Single centre in India<br>Aug to Oct 2020	Adult admitted to hospital with mild to moderate COVID-19 disease.	I: ivermectin 12mg PO on day 1 and 2 of admission (N=55)<br>C: placebo PO for 2 days (N=57)	Negative PCR test for SARS-CoV-2 on day 6 of admission<br>Fisher's exact test and rate ratio.<br>13/55 (23/6%) in the ivermectin group and 18/57 (31.6%) in the placebo group had negative PCR on day 6 (RR of 0.8 95% CI 0.4 to 1.4 P=0.347).	Ivermectin and placebo group had no difference in primary outcome.
Ravichandran [111]	Open label<br>Single centre in India<br>Unclear dates	Hospitalized adults with positive PCR test for COVID-19 with oxygen saturation of 94% or more.	I: indomethacin 75mg once to twice daily with usual care (doxycycline and ivermectin) (N=102)<br>C: standard of care (paracetamol, doxycycline and ivermectin) (N=108)	Development of hypoxia / desaturation<br>Descriptive<br>Hypoxia occurred in 0/102 (0%) in the indomethacin group and 20/108 (19%) in the standard of care group	Indomethacin is a safe and effective rug for treatment of mild and moderate COVID-19.
Elzein [112]	Quadruple blinded<br>Single centre in Lebanon<br>June to Sept 2020	Patients who have COVID-19 by nasopharyngeal swab.	I 1: gargle with 1% povidone-iodine mouthwash (N=25)<br>I 2: mouthrinse with 0.2% chlorhexidine (N=27)<br>C: mouthrinse with distilled water (N=9)	Difference in Ct value of saliva saliva PCR testing before and 5 minutes after mouthwash.<br>Paired and unpaired T-test.<br>Delta Ct was significantly different between distilled water wash and each of the 2 solutions Povidoneiodine<br>and Chlorhexidine 0.2% (p value 0.012 and 0.0024 respectively). Mean Ct value was 29.88 (6.2) before and 34.36 (6.3) after in povidone-iodine group (P<0.0001). It was 27.69 (7.16) before and 33.9 (7.08) after in 0.2% chlorhexidine group (P<0.0001). There was no different before and after in the distilled water group (P=0.566).	Chlorhexidine 0.2% and 1% Povidone-iodine oral solutions are effective preprocedural mouthwashes against salivary SARS-COV-2 in dental treatments.

© 2023 Bai AD et al. *JAMA Network Open*.

Sablerolles [113]	Participant blinded<br>4 hospitals in Netherlands<br>Dates unclear	Healthcare workers without severe comorbidities an no prior history of COVID-19 infections who received a single Ad2.COV2.S vaccine	I 1: Ad26.COV2.S booster (N=106)<br>I 2: mRNA-1273 booster (N=112)<br>I 3: BNT162b2 booster (N=111)<br>C: no booster (N=105)	SARS-CoV-2 specific binding antibody at day 0 and 28 after boost<br>PP, Kruskal-Wallis and Fisher's exact test<br>There was no significant difference in proportion without detectable binding antibodies across groups (12.4% for no boost, 8.4% for Ad26.COV2.S boost, 11.6% for mRNA-1273 boost and 9% for BNT162b2 boost (P=0.75).<br>Booster vaccination led to significant increase in antibody compared to baseline (P<0.001) that is higher than single shot regimen. The antibody levels were higher for heterologous than homologous boost (P<0.001)	Boosters are well tolerated and immunogenic. Heterologous boosters are more immunogenic.
Chahla [114]	Open label<br>Argentina<br>Unclear dates	Healthcare personnel who perform patient care and administrative tasks.	I: ivermectin 12mg po weekly and iota-carrageenan 6 sprays per day for 4 weeks (N=117)<br>C: No prophylactic treatment (N=117)	COVID-19 infection<br>Chi-square, logistic regression model.<br>COVID-19 infection occurred in 4/117 (3.4%) in the experimental group and 25/117 (21.4%) in the control group (P<0.0001) with odd ratio of 0.13 95% CI 0.03 to 0.33 P<0.0001	Ivermectin and iota-carrageenan decreased number of health workers infected with COVID-19.
Chahla [115]	Open label<br>Argentina<br>Sept 2020 to Jan 2021	Outpatients with mild COVID-19 disease.	I: ivermectin 24mg PO weekly for 4 weeks plus symptomatic treatment (N=110)<br>C: symptomatic treatment only (N=62)	Proportion of participants with symptoms reported from 5 to 9th day. Medical discharge.<br>Chi-square test and logistic regression model.<br>48/98 in experimental group an 22/27 in control group had symptoms on day 5 to 9 (P=0.0026).<br>30% in experimental group an 49.9% in control group remained in score 2 of WHO scale (P=0.039).<br>Medical discharge occurred in 108/110 in experimental group and 54/62 in control group (P=0.003) with OR of 7.99 (95% CI 1.64 to 38.97 P=0.003).	Ivermectin could prevent evolution of COVID-19 infection to severe disease requiring hospitalization.
Izikson [116]	Phase 2 open label<br>6 sites in US<br>July to Aug 2021	Adults of 65 years or older who have received a second mRNA-1273 dose at least 5 months ago	I 1: quadrivalent influenza vaccine (QIV-HD) plus mRNA-1273 vaccine co-administration (N=100)<br>I 2: QIV-HD booster alone (N=92)<br>I 3: mRNA-1273 booster alone (N=104)	Adverse events to day 22. Haemagglutination inhibition (HAI) and SARS-CoV-2 binding antibody response<br>Modified ITT for safety analysis, PP for immunogenicity endpoints.<br>Descriptive only with 95% CI.<br>Most common grade 3 reactions were pain, erythema, fatigue, malaise and myalgia. Unsolicited adverse events were similar across groups. There were no grade 3 unsolicited adverse reactions and no serious adverse events or deaths. HAI rose to similar levels for co-administration group and QIV-HD group. SARS-CoV-2 binding antibody rose to similar levels in the co-administration group and mRNA-1273 group.	There were no safety concerns or immune interferences with concomitant administration of QIV-HD and mRNA-1273 booster.
Ramakrishnan [117]	Phase 2, open label<br>Community in Oxfordshire, UK	Adults with mild COVID-19 infection and within	I: budesonide 800ug inhaled twice daily until recovery (N=70)	COVID-19 related urgent care visit, emergency department assessment or hospitalization.<br>PP and ITT, Chi-square test	Early administration of inhaled budesonide reduced likelihood of needing urgent medical care

© 2023 Bai AD et al. *JAMA Network Open*.

	July to Dec 2020	7 days of symptom onset	C: usual care (N=69)	Primary outcome occurred in 10 patients in the usual care group (14%) and 1 patient (1%) in the budesonide group (ARR 13% 95% CI 4.3% to 21.8% P=0.004)	and reduced time to recovery following early COVID-19 infection.
Sridhar [118]	Phase 2 double blind<br>20 sites in US and Honduras<br>Feb to March 2021	Adults including those at increased risk of severe COVID-19	I 1: AS03-ajuvated SARS-CoV-2 recombinant protein vaccine CoV2 preS dTM 5ug for 2 doses on day 1 and 22 (N=240)<br>I 2: preS dTM 10ug for 2 doses on day 1 and 22 (N=239)<br>I 3: preS dTM 15ug for 2 doses on day 1 and 22 (N=242)	Safety, reactogenicity and neutralising antibodies against D614G variant.<br>Modified ITT for safety analysis, PP for immunogenicity. Descriptive with 95% CI.<br>There were no vaccine related serious adverse events. Solicited reaction were reported similarly between groups, mostly mild to moderate. 96.9%, 97.0% and 97.6% of participants had ≥4-fold-rise in NAb titres from baseline in the 5μg-, 10μg- and 15μg-dose groups, respectively.	Two injections of the vaccine demonstrate acceptable safety and reactogenicity, and robust immunogenicity.
Somersan-Karakaya [119]	Phase 1/2/3 double blind<br>103 sites in US, Brazil, Chile, Mexico, Moldova and Romania<br>Ended on April, 2021	Hospitalized patients on low flow or no supplemental oxygen	I 1: Casirivimab and imdevimab (REGEN-COV) 2.4g IV once (N=406)<br>I 2: REGEN-COV 8g IV once (N=398)<br>C: placebo IV once (N=393)	Time weighted average (TWA) daily change from baseline viral load in NP swab day 1 to 7. Proportion of patients who died or required mechanical ventilation from day 6 to 29.<br>Modified ITT, virologic analysis using analysis of covariance model. Clinical endpoint analyzed using an exact method for binomial distribution or asymptotic normal approximation method.<br>TWA daily change in viral load was -1.03 log10 copies/mL (CI: -1.22, -0.84) in the placebo group compared with –1.31 log10<br>copies/mL (CI: -1.43, -1.18) in the REGEN-COV combined dose group, with an LS mean difference vs. placebo of -0.28 log10 copies/mL (CI: -0.51, -0.05; P=0.0172). Proportions of patients who died or required mechanical ventilation was 28/211 (13.3%) in placebo group and 44/445 (9.9%) in the REGE-COV group with relative risk reduction of 25.5% 95% CI -16.2% to 52.2% P=0.2048).	In hospitalized patients with COVID-19 infection on low-flow or no oxygen, REGEN-COV reduced viral load, and risk of mechanical ventilation and mortality.
Fragoso-Saavedra [120]	Double blind<br>2 hospitals in Mexico<br>May 2020 to Jan 2021	Hospitalized patients with COVID-19 infection confirmed by PCR and risk factor for requiring mechanical ventilation or death.	I: pyridostigmine 60mg PO per day until pre-specified outcome, discharge or 14 days (N=94)<br>C: placebo PO per day until pre-specified outcome, discharge or 14 days (N=94)	Composite of initiation of invasive mechanical ventilation and all-cause mortality in 28 days<br>ITT, survival analysis and Cox proportional hazard model<br>Composite outcome occurred in 11 (11.7%) in pyridostigmine group and 22 (23.4%) in the placebo group with hazard ratio of 0.47 (95% CI 0.23 to 0.96 P=0.03).	Pyridostigmine significantly reduced mechanical ventilation and death in hospitalized patients with severe COVID-19 infection.
Heath [121]	Phase 3, observer blinded, placebo controlled<br>33 sites in UK<br>Sept to Nov 2020	Adults aged 18 to 84 years who were healthy or had stable chronic medical conditions.	I: recombinant nanoparticle vaccine NVX-CoV2373 5ug intramuscular for 2 doses 21 days apart (N=7,569)<br>C: placebo intramuscular for 2 doses (N=7,570)	Virologically confirmed symptomatic COVID-19 with onset 7 days after second vaccination in serologically negative participants.<br>PP, vaccine efficacy based on RR estimated based on Poisson regression<br>Symptomatic COVID-19 infection occurred in 10 people within the vaccine group and 96 people within placebo group with vaccine efficacy of	A two-dose regimen of NVX-CoV2373, given 21 days apart, was found to be safe and 89.7% effective against symptomatic Covid-19 caused by both prototype and B1.1.7 variants.

© 2023 Bai AD et al. *JAMA Network Open*.

				89.7% (95% CI 80.2% to 94.6%). There were 5 cases of severe COVID-19, which all occurred in the placebo group.	
Panda [122]	Open label, stratified trial<br>Single centre in India<br>March to Oct 2020	Adults with symptomatic COVID-19 disease confirmed by positive PCR	Severe category:<br>I 1: hydroxychloroquine plus ribavirin for 10 days (N=23)<br>I 2: hydroxychloroquine, ribavirin, and lopinavir/ritonavir for 10 days (N=24)<br>C: standard treatment (N=24)<br>Non-severe category<br>I: hydroxychloroquine plus ribavirin for 10 days (N=20)<br>C: standard treatment (N=20)	Serious adverse events, clinical recovery at 72 hours of therapy, laboratory recovery of organ involvement, time to negative PCR testing<br>PP, Chi-square or Fisher's test for categorical variables. ANOVA or t-test for continuous variables.<br>2 participants developed elevated liver enzyme due to ribavirin. 1 participant developed prolonged QT due to hydroxychloroquine.<br>Time to clinical and laboratory recovery were not different between the groups. Time to negative PCR were not significant.	There was no significant difference in clinical outcome across groups.
Madhi [123]	Phase 1b/2, double blinded<br>Multicentre in South Africa<br>June to Nov 2020	Adults aged 18 to 65 years with no or well controlled chronic medical conditions.	I: ChAdOx1 nCoV-19 containing 5x10^10 viral particles intramuscular for 2 doses 21-35 days apart (N=1011)<br>C: placebo intramuscular for 2 doses 21-35 days apart (N=1010)	Safety and vaccine efficacy >14 days following second dose against laboratory confirmed symptomatic COVID-19.<br>PP, vaccine efficacy based on RR<br>Adverse event and serious adverse event rates were similar between the groups.<br>COVID occurred in 19/750 (2.5%) within vaccine group and 23/717 (3.2%) in placebo group with vaccine efficacy of 21.9% (95% CI -49.9% to 59.8%).	A two-dose regimen of ChAdOx1-nCoV19 did not show protection against mild-moderate Covid-19<br>due to B.1.351 variant, however, VE against severe Covid-19 is undetermined.
Shenoy [124]	Phase 3 double blind<br>3 hospitals in Kuwait<br>Aug 2020 to Jan 2021	Adults with PCR confirmed moderate to severe COVID-19 requiring hospitalization	I: favipiravir 1800mg PO twice daily on day 1 then 800mg PO twice daily for day 2 to 10 (N=175)<br>C: placebo PO for 10 days (N=178)	Time to resolution of hypoxia (WHO ordinal scale of 4 or less over 5 days)<br>ITT, Cox proportional hazards model<br>Median time to resolution of hypoxia was 7 days in favipiravir group and 8 days in placebo group with HR of 0.991 (95% CI 0.767 to 1.280 P=0.94).	The trial did not find favipiravir to be effective in moderate to severe, hospitalized COVID-19 patients.
Shoham [125]	Phase 2 double blind<br>19 sites in US<br>June 2020 to March 2021	Asymptomatic adults with close contact exposure to a person with confirmed COVID-19 in previous 120 hours and negative ARS-CoV-2 test within 24 hours	I: high titer (>=1:320) convalescent plasma 1 unit IV (N=87)<br>C: standard plasma 1 unit IV (N=93)	Incident symptomatic COVID-19 infection by day 28 based on PCR testing<br>Modified ITT, time to event analysis to compare restricted mean survival time, risk difference.<br>COVID-19 infection occurred in 12/81 (14.8%) patients treated with convalescent plasma and 13/87 (14.9%) treated with placebo with restricted mean survival time of 25.3 days for convalescent plasma and 25.2 days for control (P=0.47). The risk difference was -0.01 (P=0.42).	Post-exposure prophylaxis with convalescent plasma did not prevent COVID-19 infection.
Mendez-Flores [126]	Double blind<br>Single centre in Mexico<br>Aug to Nov 2020	Outpatients with symptomatic COVID-19	I: polymerized type I collagen (PTIC) intramuscular every 12 hours for 3 days, then daily for 4 days (N=45)<br>C: placebo intramuscular with same schedule as above (N=44)	Mean reduction in level of IP-10 at day 8 compared to baseline.<br>ITT, Wilcoxon-rank sum test<br>IP-10 decreased by 75% in the intervention group (P<0.001) and 40% in the placebo group (P=0.015). Reduction was greater in the PTIC group (P=0.0047).	Treatment with PTIC decreased IP-10, and other inflammatory markers, resulting in lower proportion of hypoxia and shorter duration of symptoms.

© 2023 Bai AD et al. *JAMA Network Open*.

Korper [127]	Open label<br>13 hospitals in Germany<br>Aug to Dec 2020	Hospitalized adults with COVID-19 requiring respiratory support or intensive care treatment.	I: high dose convalescent plasma 3 units IV once (N=53)<br>C: standard treatment alone (N=52)	Composite outcome of survival and no longer fulfilling criteria for severe COVID-19 on day 21<br>ITT, Fisher's exact test<br>Composite outcome occurred in 43.4% within convalescent plasma group an 32.7% within control group (P=0.32)	Convalescent plasma did not result in significant difference in primary and secondary outcomes.
Betka [128]	Single blind, cross over trial<br>Single centre in Switzerland<br>Nov 2020 to April 2021	Patients recovering from COVID-19 pneumonia who has refractory breathlessness and MoCA score of >=24	I: synchronous feedback of breathing, embodied via an avatar in immersive virtual reality (iVR)<br>C: asynchronous feedback of breathing, embodied via an avatar in immersive virtual reality<br>Patients can be randomized to asynchronous then synchronous (N=12), or synchronous then asynchronous (N=14)	Breathing discomfort score on a 7-point Likert scale, feedback questionnaire.<br>Linear mixed model<br>The mean comfort rating was 0.75 ±1.78 during the asynchronous condition and 1.29± 1.37 during the synchronous condition, with an estimated difference of 0.54 (95% CI 0.05 to 1.04) (P<0.05). 91.2% of the patients were satisfied by the intervention (Satisfaction: mean score = 1.75 ± 1.65; t = 5.20, p < 0.0001, 95% CI 1.17 to inf). In addition, 66.7% rated the iVR intervention as beneficial for their breathing (Respiratory benefit: 0.71 ± 1.92, t = 1.81, p < 0.05, 95% CI 0.04 to inf).	Immersive virtual reality is a feasible and safe neuro-rehabilitation tool to improve breathing comfort in patients recovering from COVID-19 infection.
Thomas [129]	Phase 2/3, placebo controlled, observer blinded<br>152 sites in US, Argentina, Brazil, South Africa, Germany and Turkey<br>July to Oct 2020	People aged 12 years or older who were healthy or had stable chronic medical conditions	I: BNT162b2 30ug intramuscular 2 doses 21 days apart (N=22,026)<br>C: saline placebo intramuscular 2 doses 21 days apart (N=22,021)	Vaccine efficacy against laboratory confirmed COVID-19 with onset >=7 days post 2nd dose and safety data up to 6 months.<br>Modified ITT, vaccine efficacy as 1 – incidence rate ratio, descriptive with 95% CI<br>There were 77 cases of COVID-19 in the vaccine group and 850 cases in the placebo group. Vaccine efficacy was 91% (95% CI 89.0% to 93.2%) up to 6 months.<br>More BNT162b2 recipients reported local and systemic reactions. There were no new safety signals. Few had serious adverse events.	BNT162b2 vaccine was safe and highly efficacious in preventing COVID-19.
Sivapalasingam [130]	Phase 2/3 adaptive trial, double blinded<br>65 sites in US<br>March to July 2020	Adults hospitalized with laboratory confirmed COVID-19 requiring supplemental oxygen and / or assisted ventilation.	Phase 2<br>I 1: sarilumab 400mg (N=180)<br>I 2: sarilumab 200mg (N=187)<br>C: placebo (N=90)<br><br>Phase 3<br>I 1: sarilumab 400mg (N=582)<br>I 2: sarilumab 200mg (N=489)<br>C: placebo (N=294)	Proportion of patients with >=1 point improvement in clinical status by day 22<br>ITT, stratified Cochran–Mantel–Haenszel (CMH) test for two proportions.<br>Proportion with improvement was 43.2% in sarilumab group and 35.5% in placebo with risk difference of 7.5% (95% CI -7.4% to 21.3% P=0.3261).	In hospitalized patients with Covid-19 receiving MV, numerical benefits with sarilumab did not achieve statistical significance.
Patankar [131]	Double blind<br>Single centre in India<br>Aug to Sept 2020	Adults with laboratory proven COVID-19 admitted to hospital for mild to moderate disease.	I: polyherbal formulation 1 capsule PO twice daily for 30 days (N=39)<br>C: placebo 1 capsule PO twice daily for 30 days (N=33)	Reduction in viral load by day 4<br>Mann-Whitney U test<br>Median viral load decrease from 662,081 viral copies/mL (IQR: 56,904 to 19,485,712) to 48,963 copies/mL (IQR 8,774 to 1,248,257) in the intervention group (P=0.002). In the placebo group, viral load decreased from 385,670 (IQR 30,807 to 131,511,846) to 66,845 (IQR 11,978 to 836,124) (P=0.106).	Polyherbal formulation reduces viral load, contributes to immunomodulation and leads to clinical improvement.

© 2023 Bai AD et al. *JAMA Network Open*.

Naggie [132]	Double blind<br>34 centres in US<br>April to Nov 2020	Adult healthcare workers at risk for COVID-19 infection	I: hydroxychloroquine 600mg PO twice daily on day 1 followed by 400mg PO daily on day 2 to 30<br>C: placebo PO with the same schedule	Composite of confirmed or suspected COVID-19 clinical infection by day 30<br>Risk difference with 95% CI using Miettinen-Nurminen method, Fisher's exact test, logistic regression<br>Composite endpoint occurred in 41 (6%) in hydroxychloroquine group and 53 (7.8%) in placebo group (risk difference of -1.8% 95% CI -4.6% to 0.9% P=0.20).	Hydroxychloroquine did not have clinical benefit in prevention of COVID-19.
Bukhari [133]	Open label<br>Single centre in Pakistan<br>March to June 2020	Adults with PCR proven COVID-19 infection	I: ivermectin 12mg PO once (N=41)<br>C: standard of care (N=45)	Days to negative PCR<br>Statistical method not described<br>At day 14, 25/45 in control arm were negative and 4/45 in intervention arm were negative as per Table 3 (likely a number error in the pre-print) P=0.001.	Ivermectin resulted in early viral clearance and is a potential treatment option.
Hsieh [134]	Phase 2, double blind<br>10 medical centres and 1 hospital in Taiwan<br>Dec 2020 to April 2021	Adults aged >=20 years who were generally healthy or had stable medical conditions.	I: a recombinant protein vaccine MVC-COV1901 15ug intramuscular for 2 doses 28 days apart (N=3,295)<br>C: placebo saline intramuscular for 2 doses 28 days apart (N=549)	Safety, tolerability and immunogenicity (GMT, GMT ratio and seroconversion rate of neutralising) by day 57.<br>Modified ITT for safety, PP for immunogenicity.<br>Descriptive and t-test or Wilcoxon rank sum test for continuous variables and Chi-square or Fisher's exact test for categorical variables.<br>There were no vaccine related serious adverse events. Common adverse events include injection site pain, malaise/fatigue. At 28 days, the GMT was 662.3 (95% CI 628.7 to 697.7), GMT ratio was 163.2 (95% CI 155.0 to 171.9) and seroconversion rate was 99.8%. The seroconversion rate was 0% with placebo.	MVC-COV1901 was safe and immunogenic, which supports it being tested in phase 3 trials.
Munch [135]	Blinded trial<br>26 hospitals in Europe and India<br>Aug 2020 to May 2021	Adults with confirmed COVID-19 receiving at least 10 L/min of oxygen or mechanical ventilation	I: dexamethasone 12mg IV daily for 10 days (N=497)<br>C: dexamethasone 6mg IV daily for 10 days (N=485)	Number of days alive without life support at 28 days<br>ITT, Kryger Jensen and Lange test adjusted for stratification variables.<br>Median number of days without life support was 22.0 (IQR 6.0 to 28.0) in the 12mg group and 20.5 (IQR 4.0 to 28.0) in the 6mg group. The adjusted mean difference was 1.3 (95% CI 0.0 to 2.6; P=0.066).	Dexamethasone 12mg did not result in more days alive without life support in COVID-19 infection and severe hypoxia.
Dorward [136]	Open label, adaptive platform trial<br>UK<br>March to May 2021	Outpatient adults age >=65 years, or 18-65 years with comorbidities or shortness of breath, who have been unwell for 14 days or less with COVID-19	I: colchicine 500ug PO daily for 14 days (N=156)<br>C: usual care (N=1145)	Time to first self-reported recovery, and hospitalization / death related to COVID-19, within 28 days<br>Bayesian piecewise exponential model and Bayesian logistic regression model.<br>Time to first self reported recovery was similar between the two groups with hazard ratio of 0.919 (95% credible interval 0.72 to 1.16) with increase of 1.14 days [-1.86 to 5.21] in median time to self-reported recovery for colchicine.<br>COVID-19 related hospitalization/deaths were	Colchicine did not improve time to recovery in people at higher risk of complications with COVID-19 in the community.

© 2023 Bai AD et al. *JAMA Network Open*.

				similar in both groups with odds ratio of 0.76 [0.28 to 1.89] and estimated difference of -0.4% [-2.7% to 2.4%].	
Yu [137]	Open label, adaptive platform trial<br>UK<br>Nov 2020 to March 2021	Outpatient adults age >=65 years, or 50-65 years with comorbidities, who have been unwell for 14 days or less with COVID-19	I: budesonide 800ug inhaled twice daily for 14 days (N=805)<br>C: usual care (N=1100)	Time to first self reported recovery, and hospitalization / death related to COVID-19 within 28 days<br>Bayesian piecewise exponential model and Bayesian logistic regression model.<br>Time to first self-reported recovery was shorter in budesonide group with hazard ratio of 1.208 (95% credible interval 1.076 to 1.356) with median benefit of 3.011 (95% credible interval 1.134 to 5.410 days), probability of superiority of 0.999. Hospitalization/deaths occurred in 59/692 (8.5%) in the budesonide group and 100/968 (10.3%) in usual care with estimated percentage benefit of 2.1% (95% credible level -0.7% to 4.8%) and probability of superiority of 0.928.	Inhaled budesonide reduced time to recovery.
REMAP-CAP investigators [138]	Open label, adaptive platform trial<br>113 sites across 6 countries<br>April to Nov 2020	Adult patients with COVID-19 within 24 hours of commencing organ support in ICU	I 1: tocilizumab 8g/kg up to 800mg IV once an then a second dose 12 to 24 hours if needed {N=353)<br>I 2: sarilumab 400mg IV once (N=48)<br>C: standard of care (N=402)	Ordinal scale combining in-hospital mortality and days free of organ support to day 21.<br>Bayesian cumulative logistic model<br>Median organ support free days were 10 (IQR -1, 16) for tocilizumab, 11 (IQR 0, 16) for sarilumab, and 0 (IQR -1, 15) for control. Relative to control, the adjusted OR was 1.64 (95% CrI 1.25 to 2.14) for tocilizumab with >99.9% probability of superiority and 1.76 (95% CrI 1.17 to 2.91) for sarilumab with 99.5% probability of superiority.	In critically ill patients with Covid-19 receiving organ support in intensive care, treatment with the IL-6 receptor antagonists, tocilizumab and sarilumab, improved outcome, including survival.
REMAP-CAP investigators [139]	Open label, adaptive platform trial<br>International<br>Ended in April 2021	Hospitalized adults with moderate to severe COVID infection.	I 1: tocilizumab 8mg/kg maximum 800mg IV once with second dose in 12-24 hours if necessary (N=972)<br>I 2: sarilumab 400mg IV once (N=485)<br>I 3: anakinra 300mg IV loading dose then 100mg IV every 6 hours for 14 days (N=378)<br>I 4: interferon-beta1a (N=21)<br>C: standard of care (N=418)	Ordinal scale combining in-hospital mortality and days free of organ support by day 21.<br>Bayesian model<br>Median organ support free days were 7 (IQR -1 to 16) in tocilizumab group, 9 (IQR -1 to 17) in sarilumab group, 0 (IR 1 to 15) in anakinra group and 0 (IQR -1 to 15) in placebo group. The median adjusted odds ratio was 1.46 (95% CrI 1.13 to 1.87) for tocilizumab with 99.8% probability of superiority over control, 1.50 (95% CrI 1.13 to 2.00) for sarilumab with 98.8% probability of superiority over placebo, 0.99 (95% CrI 0.74 to 1.35) for anakinra with 46.6% probability of superiority over control.	In patients with severe COVID-19 receiving organ support, tocilizumab and sarilumab are similarly effective at improving survival and reducing duration of organ support. Anakinra is not effective in this population.
REMAP-CAP investigator [140]	Open label, adaptive platform trial<br>129 sites in Australia, Canada, UK and US<br>March 2020 to Jan 2021	Critically ill adult patients with confirmed COVID-19 who were receiving intensive care level organ support.	I: high-titer convalescent plasma IV once (N=1084)<br>C: standard of care (N=916)	Ordinal scale combining in-hospital mortality and days free of organ support by day 21.<br>Bayesian cumulative logistic model<br>Median organ support free days were 0 (IQR -1 to 16) for convalescent plasma group and 3 (IQR -1 to 16) for control group. The median adjusted OR was 0.97 (95% CrI 0.83 to 1.15) and posterior probability of futility was 99.4%.	In critically ill adults with confirmed Covid-19, treatment with convalescent plasma, did not improve clinical outcomes.

© 2023 Bai AD et al. *JAMA Network Open*.

Weinreich [141]	Phase 1/2, double blind, adaptive trial<br>54 sites in US<br>June to Sept 2020	Outpatients who were adults and had COVID-19 infection confirmed by PCR testing.	I 1: REGEN-COV (casirivimab and imdevimab) 2400mg IV once (N=266)<br>I 2: REGE-COV 8000mg IV once (N=267)<br>C: placebo (=266)	Time weighted change in viral load from day 1 to 7.<br>Analysis of covariance model<br>The least squares mean difference at day 7 compared to placebo was -0.38 (95% CI -0.61 to -0.15; P=0.0011) for REGEN-COV 2400mg group and -0.34 (95% CI -0.57 to -0.11; P=0.0035) for REGEN-COV 8000mg group.	REGEN-COV treatment of outpatients significantly reduced SARS-CoV-2 viral load and Covid-19-related medically-attended visits.
Nguyen [142]	Phase 1/2, double blind<br>2 sites in Vietnam<br>Dec 2020 to	Healthy adults	Phase 2<br>I 1: Nanocovax 25ug intramuscular on day 0 and 28 (N=160)<br>I 2: Nanocovax 50ug intramuscular on day 0 and 28 (N=160)<br>I 3: Nanocovax 75ug intramuscular on day 0 and 28 (N=160)<br>C: placebo intramuscular on day 0 and 28 (N=80)	Safety through day 56 and anti-S IgG antibody response up to 42 days<br>Descriptive for safety, descriptive and T-test for anti-S IgG titer.<br>Adverse event rates were similar across all groups. There were no serious adverse events related to vaccine.<br>On day 42, geometric mean concentration (GMC) increased to 60.48 U/ml (95% CI 51.12 to 71.55) in 25ug group, 49.11 U/ml (95% CI 41.26 to 58.46) in 50ug group, and 57.18 (95% CI 48.4 to 67.5) in 75ug group. GMC was 0.29 (95% CI 0.25 to 0.32) in placebo group. Seroconversion occurred in 99% to 100% in all vaccine groups.	Nanocovax is safe and immunogenic.
Hinks [143]	Open label<br>19 centres in UK<br>June 2020 to Jan 2021	Adult outpatient with clinical diagnosed highly probable or confirmed mild to moderate COVID-19 infection within 14 days of symptoms.	I: azithromycin 500mg PO daily for 14 days (N=147)<br>C: standard care (N=148)	Proportion of participants with death or hospitalization over 28 days<br>ITT, Chi-square, logistic regression, time to event, RR and ARR<br>15/145 (10.3%) in azithromycin group and 17/147 (11.6%) in control group were hospitalized or died. Adjusted OR was 0.91 (95% CI 0.43 to 1.92; P=0.80).	In mild to moderate COVID-19 in outpatients, azithromycin did not reduce hospitalization or death.
Reis [144]	Placebo controlled, adaptive platform trial<br>11 cities in Brazil<br>Jan to Aug 2021	Symptomatic outpatient adults positive for COVID-19 within 7 days of symptom onset who had known risk factor for progression to severe disease.	I: fluvoxamine 100mg PO twice daily for 10 days (N=739)<br>C: placebo PO twice daily for 10 days (N=733)	Composite outcome of emergency room observation for >6 hours or hospitalization from COVID-19 by 28 days<br>ITT, Bayesian beta-binomial model<br>Composite occurred in 77/739 for fluvoxamine and 108/733 for placebo with RR of 0.71 (95% CrI 0.54 to 0.93) with probability of superiority of 99.4%.	Fluovoxamine reduced need for emergency room observation or hospitalization in high-risk outpatients with early diagnosed COVID-19.
Quinn [145]	Phase 1b/2 open label platform trial<br>2 hospitals in UK<br>Sept 2020 to Feb 2021	Hospitalized patients with confirmed COVID-19 pneumonitis	I: nafamostat continuous infusion 0.2mg/kg/hr IV for 7 days(N=21)<br>C: standard of care (N=21)	Safety and tolerability<br>Modified ITT, descriptive, Bayesian generalised linear mixed effects model for continuous variables, Bayesian logistic generalized linear model (GLM) for binary outcomes, and Bayesian Poisson GLM used for rates.<br>Adverse events occurred in 78% of nafamostat group and 57% of control group. There were no serious averse events. 15/21 stopped prior to 7 day planned course in nafamostat group. The	In hospitalised patients with COVID-19, we did not observe evidence of anti-inflammatory, anticoagulant or antiviral activity with intravenous Nafamostat.

© 2023 Bai AD et al. *JAMA Network Open*.

				most common reason was hyperkalemia in 6/21. One patient developed pulmonary embolism and one patient suffered an ischemic stroke on nafamostat.	
Marconi [146]	Phase 3, double blind<br>101 sites in 12 countries<br>June 2020 to Jan 2021	Adults hospitalized with laboratory confirmed COVID-19 infection with symptoms or evidence of pneumonia plus elevated inflammatory markers.	I: baricitinib 4mg PO daily for 14 days (N=764)<br>C: placebo PO daily for 14 days (N=761)	Progression to high-flow oxygen, non-invasive ventilation, invasive mechanical ventilation or death by 28 days.<br>ITT, logistic regression<br>Endpoint occurred in 27.8% in the baricitnib group and 30.5% in placebo group with odds ratio of 0.85 (95% CI 0.67 to 1.08; P=0.18).	Reduction of disease progression did not reach statistical significance, but baricitinib significantly reduced mortality.
Shinde [147]	Phase 2, placebo controlled, observer blinded<br>16 sites in South Africa<br>Aug to Nov 2020	HIV negative adults or medically stable people living with HIV	I: VVX-CoV2373 intramuscular 2 doses 21 days apart (N=2199)<br>C: placebo intramuscular 2 doses 21 days apart (N=2188)	Safety and vaccine efficacy >=7 days after 2nd dose against laboratory confirmed symptomatic COVID-19.<br>Modified ITT and descriptive for safety. PP, vaccine efficacy as 1 – RR by Poisson regression.<br>Medically attended adverse events and serious adverse events were infrequent but occurred slightly more often in vaccine group, none related to vaccine.<br>There were 15 and 29 mild to moderate COVID-19 cases in vaccine and placebo group respectively. Vaccine efficacy was 49.4% (95% CI 6.1% to 72.8%). The only severe case occurred in placebo group.	NVX-CoV2373 was efficacious in preventing COVID-19.
Trieu [148]	Phase 4, open label<br>3 sites in India<br>Oct to Nov 2020	Adults with symptomatic mild to moderate COVID-19 not on oxygen therapy	I: gelatin capsule formulation of the Artemisia extract Ayurveda (ARTIVeda/PulmoHeal) orally for 5 days (N=39)<br>C: standard of care alone (N=21)	Recovery from symptoms and signs of COVID-19 based on WHO score of 1 by day 5.<br>Fisher's exact test<br>31/39 (79.5%) in the treatment group and 12/21 (57.1%) in the control group became asymptomatic (P=0.028)	ARTIVeda/PulmoHeal resulted in faster recovery in patients with mild to moderate COVID-19.
Fischer [149]	Phase 2, double blind<br>10 sites in US<br>June 2020 to Jan 2021	Outpatients with confirmed COVID-19 infection and symptom onset within 7 days.	I 1: molnupiravir 200mg PO twice daily for 5 days (N=23)<br>I 2: molnupiravir 400mg PO twice daily for 5 days (N=62)<br>I 3: molnupiravir 800mg PO twice daily for 5 days (N=55)<br>C: placebo PO twice daily for 5 days (N=62)	Time to undetectable level of viral RNA by PCR from nasopharyngeal swabs.<br>Kaplan Meier and log-rank test, Fisher's exact test, exact Cochran-Armitage trend test.<br>The median time to clearance was 14 days in molnupiravir 800mg group and 15 days in placebo group (P=0.001). At end of study, proportion of participants who achieved SARS-CoV-2 negativity was 92.5% in 800mg group, 91.3% in 200mg group, 78.7% in 400mg group, and 80.3% in placebo group.	Molnupiravir is highly effective at reducing nasopharyngeal SARS-CoV2 infectious virus and viral RNA.
Feng [150]	Open label<br>China<br>Jan to May 2021	Adults aged 18 to 59 years who were airport ground staff and public security officers	I 1: inactivated SARS-CoV-2 vaccine 4ug per dose intramuscular at 0 and 14 days (N=270)	Neutralization antibody seroconversion and geometric mean titer (GMT) at 28 days after 2nd dose. Occurrence of adverse reactions within 7 days of vaccine dose.	The inactivated vaccine is safe and immunogenic.

© 2023 Bai AD et al. *JAMA Network Open*.

			I 2: inactivated SARS-CoV-2 vaccine 4ug per dose intramuscular at 0 and 21 days (N=270)<br>I 1: inactivated SARS-CoV-2 vaccine 4ug per dose intramuscular at 0 and 28 days (N=269)	Modified ITT and descriptive for safety. PP and modified ITT, descriptive, ANOVA and logistic regression model for immunogenicity.<br>Seroconversion was 100% in all groups. GMT was 98.41 (95% CI 88.39 to 1.08.40) in 0-14 group, 134.40 (95% CI 123.10 to 145.70) in 0-21 group (P<0.001 vs. 0-14 group) and 145.50 (95% CI 131.30 to 159.60) in 0-28 group (P<0.001 vs. 0-14 group). Reaction were mostly mil and local. There was no serious adverse reactions.	
Temesgen [151]	Phase 3, double blind<br>28 sites in US and Brazil	Adults hospitalized for COVID-19 pneumonia with oxygen saturation of 94% or less on room air or on supplemental oxygen but not on invasive mechanical ventilation.	I: lenzilumab 600mg via 3 IV infusions administered 8 hours apart (N=261)<br>C: placebo via 3 IV infusions administered 8 hours apart (N=259)	Ventilator free survival by 28 days<br>Modified ITT, Cox proportional hazard model<br>Lenzilumab improved likelihood of ventilator free survival with HR of 1.54 (95% CI 1.02 to 2.31; P=0.041). Failure to achieve ventilator free survival occurred in 15.6% for lenzilumab group and 22.1% for placebo group.	Lenzilumab significantly improved survival without ventilation in hospitalized, hypoxic subjects with COVID-19 pneumonia over and above treatment with remdesivir and/or corticosteroids.
Pan [152]	Phase 2, double blind<br>Single centre in China<br>May 2020	Healthy adults aged 18-59 year old.	Patients were randomized to 4 schedules to receive 3ug, 6ug or placebo CoronoVac. Following are the four schedules.<br>Schedule 1 on day 0, 14, 42<br>Schedule 2 on day 0, 14, 194<br>Schedule 3 on day 0, 28, 56<br>Schedule 4 on day 0, 28, 208<br>In each schedule, there were 60 assigned to 3ug, 60 assigned to 6ug and 30 assigned placebo.	Geometric mean titers (GMTs), seropositivity, and seroconversion rate of neutralizing antibodies to live SARS-CoV-2.PP, T-test of log-transformed GMT<br>Higher GMT were observed with longer dose spacing between first 2 doses. For all 2-dose schedule, neutralizing antibody titers declined to below seropositive cutoff after 6 months. After a 3r dose given 6 months after second dose, GMT significantly increased to 137.9 [95%CI 99.9-190.4] for Schedule 2, and 143.1 [95%CI 110.8-184.7] for Schedule 4. Seropositivity for all 4 schedules were >90% on day 28 after both second ad third dose. Similar patterns were observed for the 6 µg group. Significant differences in GMT were observed between the 3 µg and 6 µg group in only a few visits for the four schedules during the study time	A third dose of CoronaVac administered 6 or more months after a second dose effectively recalled specific immune response to SARS-CoV-2, resulting in a remarkable increase in antibody levels, and indicating that a two-dose schedule generates good immune memory.

ARR = absolute risk reduction; CI = confidence interval; CrI = credible interval; GMT = geometric mean titres; HR = hazard ratio; IQR = interquartile range; ITT = Intention-to-treat; IV = intravenous; NP = nasopharyngeal; OR = odds ratio; PO = by mouth; PP = per-protocol; RR = relative risk; RT-PCR = real-time reverse transcriptase polymerase chain reaction; UK = United Kingdom; US = United States

© 2023 Bai AD et al. *JAMA Network Open*.

eReferences 1

1. Abdulamir AS, Gorial FI, Saadi SJ, Maulood MF, Hashim HA, Alnuaimi AS, Abdulrrazaq MK. Effectiveness and Safety of Niclosamaide as Add-on Therapy to the Standard of Care Measures in COVID-19 Management: Randomized controlled clinical trial. medRxiv 2021.06.10.21258709; doi: https://doi.org/10.1101/2021.06.10.21258709
2. Lima AA, Arruda EA, Pires-Neto RJ, Medeiros MS, Quirino-Filho J, Clementino MA, Gondim RN, Magalhaes LM, Cavalcante KF, Viana VA, Perdigao L. Clinical trial of efficacy and toxicity of disoproxil tenofovir fumarate and emtricitabine for mild to moderate SARS-CoV-2 infections. medRxiv 2021.09.28.21264242; doi: https://doi.org/10.1101/2021.09.28.21264242
3. Hosseinzadeh A, Emamian MH, Tavakkolian A, Kia V, Ebrahimi H, Sheibani H, Binesh E, Jafari R, Mirrezaie SM, Jafarisani M. Application of nasal spray containing dimethyl sulfoxide (DSMO) and ethanol during the COVID-19 pandemic may protect healthcare workers: A randomized controlled trials. medRxiv 2021.07.06.21259749; doi: https://doi.org/10.1101/2021.07.06.21259749.
4. Shiri AH, Raiatdoost E, Afkhami H, Ravanshad R, Hosseini SE, Kalani N, Raoufi R. The herbal combination of Sugarcane, Black Myrobalan, and mastic as a supplementary treatment for COVID-19: a randomized clinical trial. medRxiv 2021.04.27.21256221; doi: https://doi.org/10.1101/2021.04.27.21256221
5. Baxter AL, Schwartz KR, Johnson R, Rao A, Gibson RW, Cherian E, Kuchinski AM, Lyon M, Schwartz RB. Rapid initiation of nasal saline irrigation: hospitalizations in COVID-19 patients randomized to alkalinization or povidone-iodine compared to a national dataset. medRxiv 2021.08.16.21262044; doi: https://doi.org/10.1101/2021.08.16.21262044
6. Bhatt AN, Shenoy S, Munjal S, Chinnadurai V, Agarwal A, Kumar AV, Shanavas A, Kanvar R, Chandna S. 2-Deoxy-D-Glucose as an Adjunct to Standard of Care in the Medical Management of COVID-19: A Proof-of-Concept & Dose-Ranging Randomised Clinical Trial. medRxiv 2021.10.08.21258621; doi: https://doi.org/10.1101/2021.10.08.21258621
7. Gupta A, Gonzalez-Rojas Y, Juarez E, Casal MC, Moya J, Falci DR, Sarkis E, Solis J, Zheng H, Scott N, Cathcart AL, Hebner CM, Sager J, Mogalian E, Tipple C, Peppercorn A, Alexander E, Pang PS, Free A, Brinson C, Aldinger M, Shapiro AE, for the COMET-ICE Investigators. Early Covid-19 Treatment With SARS-CoV-2 Neutralizing Antibody Sotrovimab medRxiv 2021.05.27.21257096; doi: https://doi.org/10.1101/2021.05.27.21257096
8. Gupta A, Gonzalez-Rojas Y, Juarez E, Casal MC, Moya J, Falci DR, Sarkis E, Solis J, Zheng H, Scott N, Cathcart AL. Effect of the Neutralizing SARS-CoV-2 Antibody Sotrovimab in Preventing Progression of COVID-19: A Randomized Clinical Trial. medRxiv 2021.11.03.21265533; doi: https://doi.org/10.1101/2021.11.03.21265533
9. Gupta A, Madan A, Yadav B, Singhal R, Mundada PS, Pandey YK, Agarwal R, Rana R, Tripathi A, Sharma BS, Rao BC. Chyawanprash for the prevention of COVID-19 infection among healthcare workers: A Randomized Controlled Trial. medRxiv 2021.02.17.21251899; doi: https://doi.org/10.1101/2021.02.17.21251899
10. Chopra A, Tillu G, Chaudhary K, Reddy G, Srivastava A, Lakdawala M, Gode D, Reddy H, Tamboli S, Saluja M, Sarmukkaddam S. Coadministration of AYUSH 64 as an adjunct to Standard of Care in mild and moderate COVID-19: A randomised, controlled, multicentric clinical trial. medRxiv 2021.06.12.21258345; doi: https://doi.org/10.1101/2021.06.12.21258345
11. Biber A, Mandelboim M, Harmelin G, Lev D, Ram L, Shaham A, Nemet I, Kliker L, Erster O, Schwartz E. Favorable outcome on viral load and culture viability using Ivermectin in early treatment of non-hospitalized patients with mild COVID-19–A double-blind, randomized placebo-controlled trial. medRxiv 2021.05.31.21258081; doi: https://doi.org/10.1101/2021.05.31.21258081

© 2023 Bai AD et al. *JAMA Network Open*.

12. Fisher BA, Veenith T, Slade D, Gaskell C, Rowland M, Whitehouse T, Scriven J, Parekh D, Balasubramaniam M, Cooke G, Morley N. Namilumab or infliximab compared to standard of care in hospitalised patients with COVID-19 (CATALYST): a phase 2 randomised adaptive trial. medRxiv 2021.06.02.21258204; doi: https://doi.org/10.1101/2021.06.02.21258204
13. Gaborit B, Dailly E, Vanhove B, Josien R, Lacombe K, Dubee V, Ferre V, Brouard S, Ader F, Vibet MA, Le Thuaut A. Pharmacokinetics and safety of XAV-19, a swine glyco-humanized polyclonal anti-SARS-CoV-2 antibody, for COVID-19-related moderate pneumonia: a randomized, double-blind, placebo-controlled, phase IIa study. medRxiv 2021.04.15.21255549; doi: https://doi.org/10.1101/2021.04.15.21255549
14. Young BC, Eyre DW, Kendrick S, White C, Smith S, Beveridge G, Nonnemacher T, Ichofu F, Hillier J, Diamond I, Rourke E. A cluster randomised trial of the impact of a policy of daily testing for contacts of COVID-19 cases on attendance and COVID-19 transmission in English secondary schools and colleges. medRxiv 2021.07.23.21260992; doi: https://doi.org/10.1101/2021.07.23.21260992
15. Clemency BM, Varughese R, Gonzalez-Rojas Y, Morse CG, Phipatanakul W, Koster DJ, Blaiss MS. A randomized controlled trial of inhaled ciclesonide for outpatient treatment of symptomatic COVID-19 infections. medRxiv 2021.09.07.21261811; doi: https://doi.org/10.1101/2021.09.07.21261811
16. Araujo CS, Medeiros-Ribeiro AC, Saad CG, Bonfiglioli KR, Domiciano DS, Shimabuco AY, Silva MR, Yuki EF, Pasoto SG, Pedrosa T, Kupa LD. A randomized clinical trial of 2-week methotrexate discontinuation in rheumatoid arthritis patients vaccinated with inactivated SARS-CoV-2 vaccine. medRxiv 2021.11.23.21266785; doi: https://doi.org/10.1101/2021.11.23.21266785
17. Tornero C, Pastor E, del Mar Garzando M, Orduña J, Forner MJ, Bocigas I, Cedeño DL, Vallejo R, Staats P, Liebler EJ. Non-invasive Vagus Nerve Stimulation for Respiratory Symptoms of COVID-19: Results From a Randomized Controlled Trial (SAVIOR I). medRxiv 2021.09.24.21264045; doi: https://doi.org/10.1101/2021.09.24.21264045
18. Hernandez-Cardenas C, Thirion-Romero I, Rivera-Martinez NE, Meza-Meneses P, Remigio-Luna A, Perez-Padilla R. Hyroxychloroquine for the treatment of severe respiratory infection by COVID-19: A randomized controlled trial. medRxiv 2021.02.01.21250371; doi: https://doi.org/10.1101/2021.02.01.21250371
19. Mok CK, Cheng SM, Chen C, Yiu K, Chan TO, Lai KC, Ling KC, Ho LL, Peiris M, Hui DS. A RCT of a third dose CoronaVac or BNT162b2 vaccine in adults with two doses of CoronaVac. medRxiv 2021.11.02.21265843; doi: https://doi.org/10.1101/2021.11.02.21265843
20. Portal-Celhay C, Forleo-Neto E, Eagan W, Musser BJ, Davis JD, Turner KC, Norton T, Hooper AT, Hamilton JD, Pan C, Mahmood A. Phase 2 dose-ranging study of the virologic efficacy and safety of the combination COVID-19 antibodies casirivimab and imdevimab in the outpatient setting. medRxiv 2021.11.09.21265912; doi: https://doi.org/10.1101/2021.11.09.21265912
21. Singh D, Bogus M, Moskalenko V, Lord R, Moran EJ, Crater GD, Bourdet DL, Pfeifer ND, Woo J, Kaufman E, Lombardi DA. A phase 2 study of the inhaled pan-JAK inhibitor TD-0903 in severe COVID-19: Part 1. medRxiv 2021.03.09.21252944; doi: https://doi.org/10.1101/2021.03.09.21252944
22. Sullivan DJ, Gebo KA, Shoham S, Bloch EM, Lau B, Shenoy AG, Mosnaim GS, Gniadek TJ, Fukuta Y, Patel B, Heath SL. Randomized Controlled Trial of Early Outpatient COVID-19 Treatment with High-Titer Convalescent Plasma. medRxiv 2021.12.10.21267485; doi: https://doi.org/10.1101/2021.12.10.21267485
23. Huang DT, McCreary EK, Bariola JR, Minnier TE, Wadas RJ, Shovel JA, Albin D, Marroquin OC, Kip KE, Collins K, Schmidhofer M. Effectiveness of casirivimab and imdevimab, and sotrovimab during Delta variant surge: a prospective cohort study and comparative effectiveness randomized trial. medRxiv 2021.12.23.21268244; doi: https://doi.org/10.1101/2021.12.23.21268244

© 2023 Bai AD et al. *JAMA Network Open*.

24. Weinreich DM, Sivapalasingam S, Norton T, Ali S, Gao H, Bhore R, Xiao J, Hooper AT, Hamilton JD, Musser BJ, Rofail D. REGEN-COV antibody cocktail clinical outcomes study in Covid-19 outpatients. medRxiv 2021.05.19.21257469; doi: https://doi.org/10.1101/2021.05.19.21257469
25. Parikh D, Chaturvedi A, Shah N, Patel P, Patel R, Ray S. Safety and efficacy of COVID-19 hyperimmune globulin (HIG) solution in the treatment of active COVID-19 infection-Findings from a Prospective, Randomized, Controlled, Multi-Centric Trial. medRxiv 2021.07.26.21261119; doi: https://doi.org/10.1101/2021.07.26.21261119
26. Ely EW, Ramanan AV, Kartman CE, de Bono S, Liao R, Piruzeli ML, Goldman JD, Saraiva JF, Chakladar S, Marconi VC, Alatorre-Alexander J. Baricitinib plus Standard of Care for Hospitalised Adults with COVID-19 on Invasive Mechanical Ventilation or Extracorporeal Membrane Oxygenation: Results of a Randomised, Placebo-Controlled Trial. medRxiv 2021.10.11.21263897; doi: https://doi.org/10.1101/2021.10.11.21263897
27. Fedrizzi EN, Girondi JB, Sakae TM, Steffens SM, de Souza Silvestrin AN, Claro GS, Iskenderian HA, Hillmann B, Gervasi L, Trapani A, de Amorim Rodrigues P. Efficacy of the measles-mumps-rubella (MMR) vaccine in the reducing the severity of covid-19: An interim analysis of a randomised controlled clinical trial. medRxiv 2021.09.14.21263598; doi: https://doi.org/10.1101/2021.09.14.21263598
28. Balint EM, Gruener B, Haase S, Kaw-Geppert M, Thayer JF, Guendel HO, Jarczok MN. A randomized clinical trial to stimulate the cholinergic anti-inflammatory pathway in patients with moderate COVID-19-pneumonia using a slow-paced breathing technique. medRxiv 2021.12.03.21266946; doi: https://doi.org/10.1101/2021.12.03.21266946
29. Kolev E, Mircheva L, Edwards M, Johnston SL, Kalinov K, Stange R, Gancitano G, Berghe WV, Kreft S. Echinacea purpurea for the Long-term Prevention of Viral Respiratory Tract Infections during COVID-19 Pandemic: A Randomized, Open, Controlled, Exploratory Clinical Study. medRxiv 2021.12.10.21267582; doi: https://doi.org/10.1101/2021.12.10.21267582
30. Breza E, Stanford FC, Alsan M, Alsan B, Banerjee A, Chandrasekhar AG, Eichmeyer S, Glushko T, Goldsmith-Pinkham P, Holland K, Hoppe E. Doctors' and Nurses' Social Media Ads Reduced Holiday Travel and COVID-19 Infections: A Cluster Randomized Controlled Trial. National Bureau of Economic Research. medRxiv 2021.06.23.21259402; doi: https://doi.org/10.1101/2021.06.23.21259402
31. Lattman E, Bhalerao P, ShashiBhushan BL, Nargundkar N, Lattmann P, Balaram PN. Randomized, Comparative, Clinical Trial to Evaluate Efficacy and Safety of PNB001 in Moderate COVID-19 Patients. medRxiv 2021.04.16.21255256; doi: https://doi.org/10.1101/2021.04.16.21255256.
32. Gaughan E, Sethi T, Quinn T, Hirani N, Mills A, Bruce AM, MacKinnon A, Aslanis V, Li F, O'Connor R, Parker RA. GB0139, an inhaled small molecule inhibitor of galectin-3, in COVID-19 pneumonitis: a randomised, controlled, open-label, phase 2a experimental medicine trial of the safety, pharmacokinetics, and potential therapeutic value. medRxiv 2021.12.21.21267983; doi: https://doi.org/10.1101/2021.12.21.21267983
33. McCreary EK, Bariola JR, Minnier T, Wadas RJ, Shovel JA, Albin DL, Marroquin OC, Kip KE, Collins K, Schmidhofer M, Wisniewski MK. A learning health system randomized trial of monoclonal antibodies for COVID-19. medRxiv 2021.09.03.21262551; doi: https://doi.org/10.1101/2021.09.03.21262551
34. Sobngwi E, Zemsi S, Guewo-Fokeng M, Katte JC, Kouanfack C, Mfeukeu-Kuate L, Zemsi A, Wasnyo Y, Assiga-Ntsama A, Ndi-Manga JA, Tambekou JS. Doxycycline is a safe alternative to Hydroxychloroquine+ Azithromycin to prevent clinical worsening and hospitalization in mild COVID-19 patients: An open label randomized clinical trial (DOXYCOV). medRxiv 2021.07.25.21260838; doi: https://doi.org/10.1101/2021.07.25.21260838
35. Kyriazopoulou E, Poulakou G, Milionis H, Metallidis S, Adamis G, Tsiakos K, Fragkou A, Rapti A, Danoulari C, Fantoni M, Kalomenidis I. Early Anakinra Treatment for COVID-19 Guided by Urokinase Plasminogen Receptor. medRxiv 2021.05.16.21257283; doi: https://doi.org/10.1101/2021.05.16.21257283

© 2023 Bai AD et al. *JAMA Network Open*.

36. Mikhaylov EN, Lyubimtseva TA, Vakhrushev AD, Stepanov D, Lebedev DS, Vasilieva EY, Konradi AO, Shlyakhto EV. Bromhexine hydrochloride prophylaxis of COVID-19 for medical personnel: a randomized open-label study. medRxiv 2021.03.03.21252855; doi: https://doi.org/10.1101/2021.03.03.21252855
37. Goligher EC, Bradbury CA, McVerry BJ, Lawler PR, Berger JS, Gong MN, Carrier M, Reynolds HR, Kumar A, Turgeon AF, Kornblith LZ. Therapeutic anticoagulation in critically ill patients with Covid-19-preliminary report. medRxiv 2021.03.10.21252749; doi: https://doi.org/10.1101/2021.03.10.21252749
38. Syed F, Arif MA, Niazi R, Baqar JB, Hashmi UL, Batool S, Ashraf S, Arshad J, Musarrat S. Pre-Exposure Prophylaxis with Various Doses of Hydroxychloroquine among high-risk COVID 19 Healthcare Personnel: CHEER randomized controlled trial. medRxiv 2021.05.17.21257012; doi: https://doi.org/10.1101/2021.05.17.21257012
39. Cadegiani FA, Fonseca DN, McCoy J, Zimerman RA, Mirza FN, Correia MN, Barros RN, Onety DC, Israel KC, Almeida BG, Guerreiro EO. Efficacy of proxalutamide in hospitalized COVID-19 patients: a randomized, double-blind, placebo-controlled, parallel-design clinical trial. medRxiv 2021.06.22.21259318; doi: https://doi.org/10.1101/2021.06.22.21259318
40. Cadegiani FA, Zimerman RA, do Nascimento Fonseca D, do Nascimento Correia M, McCoy J, Wambier CG, Goren A. Proxalutamide (GT0918) reduces the rate of hospitalization in mild-to-moderate COVID-19 female patients: a randomized double-blinded placebo-controlled two-arm parallel trial. medRxiv 2021.07.06.21260086; doi: https://doi.org/10.1101/2021.07.06.21260086
41. Ader F, Peiffer-Smadja N, Poissy J, Bouscambert-Duchamp M, Belhadi D, Delmas C, Saillard J, Dechanet A, Mercier N, Dupont A, Alfaiate T. Antiviral drugs in hospitalized patients with COVID-19-the DisCoVeRy trial. medRxiv 2021.01.08.20248149; doi: https://doi.org/10.1101/2021.01.08.20248149
42. Fralick M, Colacci M, Munshi L, Venus K, Fidler L, Hussein H, Britto K, Fowler R, Da Costa B, Dhalla I, Dunbar-Yaffe R. Prone positioning of patients with moderate hypoxia due to COVID-19: A multicenter pragmatic randomized trial [COVID-PRONE]. medRxiv 2021.11.05.21264590; doi: https://doi.org/10.1101/2021.11.05.21264590
43. Hernandez-Bernal, F., del Carmen Ricardo-Cobas, M., Martin-Bauta, Y., Navarro-Rodriguez, Z., Piñera-Martínez, M., Quintana-Guerra, J., Urrutia-Pérez, K., Urrutia-Perez, K., Chavez-Chong, C.O., Azor-Hernandez, J.L. and Rodriguez-Reinoso, J.L., 2021. Safety, tolerability, and immunogenicity of a SARS-CoV-2 recombinant spike protein vaccine: a randomised, double-blind, placebo-controlled, phase 1-2 clinical trial (ABDALA Study). medRxiv 2021.11.30.21267047; doi: https://doi.org/10.1101/2021.11.30.21267047
44. Lescure FX, Honda H, Fowler RA, Lazar JS, Shi G, Wung P, Patel N, Hagino O. Sarilumab treatment of hospitalised patients with severe or critical COVID-19: a multinational, randomised, adaptive, phase 3, double-blind, placebo-controlled trial. medRxiv 2021.02.01.21250769; doi: https://doi.org/10.1101/2021.02.01.21250769
45. Perkins GD, Ji C, Connolly BA, Couper K, Lall R, Baillie JK, Bradley JM, Dark P, Dave C, De Soyza A, Dennis AV. An adaptive randomized controlled trial of non-invasive respiratory strategies in acute respiratory failure patients with COVID-19. medRxiv 2021.08.02.21261379; doi: https://doi.org/10.1101/2021.08.02.21261379
46. Roozen GV, Prins ML, van Binnendijk RS, den Hartog G, Kuiper VP, Prins C, Janse JJ, Kruithof AC, Feltkamp MC, Kuijer M, Roosendaal FR. Tolerability, safety and immunogenicity of intradermal delivery of a fractional dose mRNA-1273 SARS-CoV-2 vaccine in healthy adults as a dose sparing strategy. medRxiv 2021.07.27.21261116; doi: https://doi.org/10.1101/2021.07.27.21261116

© 2023 Bai AD et al. *JAMA Network Open*.

47. Tornling G, Batta R, Porter J, Bengtsson T, Parmar K, Kashiva R, Hallberg A, Cohrt AK, Westergaard K, Dalsgaard CJ, Raud J. The angiotensin type 2 receptor agonist C21 restores respiratory function in COVID19-a double-blind, randomized, placebo-controlled Phase 2 trial. medRxiv. 2021 Jan 1. medRxiv 2021.01.26.21250511; doi: https://doi.org/10.1101/2021.01.26.21250511
48. Resende GG, da Cruz Lage R, Lobê SQ, Medeiros AF, e Silva AD, Sá AT, de Assis Oliveira AJ, Sousa D, Guimarães HC, Gomes IC, Souza RP. Blockade of Interleukin Seventeen (IL-17A) with Secukinumab in Hospitalized COVID-19 patients–the BISHOP study. medRxiv 2021.07.21.21260963; doi: https://doi.org/10.1101/2021.07.21.21260963
49. Dai H, Saccardo S, Han MA, Roh L, Raja N, Vangala S, Modi H, Pandya S, Croymans DM. Behavioral nudges increase COVID-19 vaccinations: Two randomized controlled trials. medRxiv 2021.04.12.21254876; doi: https://doi.org/10.1101/2021.04.12.21254876
50. Gaitán-Duarte HG, Álvarez-Moreno C, Rincón-Rodríguez CJ, Yomayusa-González N, Cortés JA, Villar JC, Bravo-Ojeda JS, García-Peña Á, Adarme-Jaimes W, Rodríguez-Romero VA, Villate-Soto SL. Effectiveness of Rosuvastatin plus Colchicine, Emtricitabine/Tenofovir and a combination of them in Hospitalized Patients with SARS Covid-19. medRxiv 2021.07.06.21260085; doi: https://doi.org/10.1101/2021.07.06.21260085
51. Arnardottir H, Pawelzik SC, Sarajlic P, Quaranta A, Kolmert J, Religa D, Wheelock CE, Back M. Immunomodulation by intravenous omega-3 fatty acid treatment in older subjects hospitalized for COVID-19: a single-blind randomized controlled trial. medRxiv 2021.12.27.21268264; doi: https://doi.org/10.1101/2021.12.27.21268264
52. Zhang Y, Zeng G, Pan H, Li C, Kan B, Hu Y, Mao H, Xin Q, Chu K, Han W, Chen Z. Immunogenicity and safety of a SARS-CoV-2 inactivated vaccine in healthy adults aged 18-59 years: report of the randomized, double-blind, and placebo-controlled phase 2 clinical trial. medRxiv 2020.07.31.20161216; doi: https://doi.org/10.1101/2020.07.31.20161216
53. Almanza-Reyes H, Moreno S, Plascencia-López I, Alvarado-Vera M, Patrón-Romero L, Borrego B, Reyes-Escamilla A, Valencia-Manzo D, Brun A, Pestryakov A, Bogdanchikova N. Evaluation of silver nanoparticles for the prevention of SARS-CoV-2 infection in health workers: in vitro and in vivo. medRxiv 2021.05.20.21256197; doi: https://doi.org/10.1101/2021.05.20.21256197
54. Faramarzi H, Sahebkar A, Hosseinpour A, Khaloo V, Chamanpara P, Heydari MR, Najafi S, Khankahdany FF, Movahedpour A. Efficacy and safety of a novel antiviral preparation in ICU-admitted patients with COVID-19: a phase III randomized controlled trial. medRxiv 2021.11.20.21266229; doi: https://doi.org/10.1101/2021.11.20.21266229
55. Patel J, Beishuizen A, Ruiz XB, Boughanmi H, Cahn A, Criner GJ, Davy K, de-Miguel-Díez J, Fernandes S, François B, Gupta A. A randomized trial of otilimab in severe COVID-19 pneumonia (OSCAR). medRxiv 2021.04.14.21255475; doi: https://doi.org/10.1101/2021.04.14.21255475
56. Tardif JC, Bouabdallaoui N, L'Allier PL, Gaudet D, Shah B, Pillinger MH, Lopez-Sendon J, da Luz P, Verret L, Audet S, Dupuis J. Efficacy of colchicine in non-hospitalized patients with COVID-19. medRxiv 2021.01.26.21250494; doi: https://doi.org/10.1101/2021.01.26.21250494
57. Rossignol JF, Bardin M, Fulgencio J, Mogelnicki D, Brechot C. Early treatment with nitazoxanide prevents worsening of mild and moderate COVID-19 and subsequent hospitalization. Vanguard Study, Early Treatment With Nitazoxanide Prevents Worsening of Mild and Moderate COVID-19 and Subsequent Hospitalization. medRxiv 2021.04.19.21255441; doi: https://doi.org/10.1101/2021.04.19.21255441
58. Low JG, de Alwis R, Chen S, Kalimuddin S, Leong YS, Mah T, Yuen N, Tan HC, Zhang SL, Siim J, Chan Y. A phase 1/2 randomized, double-blinded, placebo controlled ascending dose trial to assess the safety, tolerability and immunogenicity of ARCT-021 in healthy adults. medRxiv 2021.07.01.21259831; doi: https://doi.org/10.1101/2021.07.01.21259831
59. Lundgren JD, ACTIV-3/TICO Bamlanivimab Study Group. Clinical and virological response to a neutralizing monoclonal antibody for hospitalized patients with COVID-19. medRxiv 2021.07.19.21260559; doi: https://doi.org/10.1101/2021.07.19.21260559

© 2023 Bai AD et al. *JAMA Network Open*.

60. Liu J, Yang W, Liu Y, Lv C, Ruan L, Zhao C, Huo R, Shen X, Miao Q, Lv W, Li H. Chinese medicine (Q-14) in the Treatment of Patients with Coronavirus Disease 2019 (COVID-19): A Single-center, Open label, Randomised Controlled Trial. medRxiv 2021.01.25.21249417; doi: https://doi.org/10.1101/2021.01.25.21249417
61. Li J, Hou L, Guo X, Jin P, Wu S, Zhu J, Pan H, Wang X, Song Z, Wan J, Cui L. Heterologous prime-boost immunization with CoronaVac and Convidecia. medRxiv 2021.09.03.21263062; doi: https://doi.org/10.1101/2021.09.03.21263062
62. Dupuis J, Laurin P, Tardif JC, Hausermann L, Rosa C, Guertin MC, Thibaudeau K, Gagnon L, Cesari F, Robitaille M, Moran JE. Fourteen-days Evolution of COVID-19 Symptoms During the Third Wave in Non-vaccinated Subjects and Effects of Hesperidin Therapy: A randomized, double-blinded, placebo-controlled study. medRxiv 2021.10.04.21264483; doi: https://doi.org/10.1101/2021.10.04.21264483
63. Haran JP, Zheng Y, Knobil K, Alonzo-Palma N, Lawrence J, Wingertzahn M. Targeting the Microbiome With KB109 in Outpatients with Mild to Moderate COVID-19 Reduced Medically Attended Acute Care Visits and Improved Symptom Duration in Patients With Comorbidities. medRxiv 2021.03.26.21254422; doi: https://doi.org/10.1101/2021.03.26.21254422
64. Song JY, Kim YS, Eom JS, Kim JY, Lee JS, Lee J, Choi WS, Heo JY, Sohn JW, Lee KD, Cho D. Oral antiviral clevudine compared with placebo in Korean COVID-19 patients with moderate severity. medRxiv 2021.12.09.21267566; doi: https://doi.org/10.1101/2021.12.09.21267566
65. Rojas-Serrano J, Ireri AM, Thirion-Romero JV, Alejandra FM, Ramírez-Venegas KM, Pérez-Padilla R. Hydroxychloroquine For Prophylaxis Of COVID-19 In Health Workers: A Randomized Clinical Trial. medRxiv 2021.05.14.21257059; doi: https://doi.org/10.1101/2021.05.14.21257059
66. Beltran-Gonzalez JL, Gonzalez-Gamez M, Mendoza-Enciso EA, Esparza-Maldonado RJ, Hernanez-Palacios D, Duenas-Campos S, Ovalle-Robles I, Macias-Guzman MJ, Diaz AL, Pena CM, Martinez-Medina L. Efficacy and safety of ivermectin and hydroxychloroquine in patients with severe COVID-19. A randomized controlled trial. medRxiv 2021.02.18.21252037; doi: https://doi.org/10.1101/2021.02.18.21252037.
67. Gonzalez JL, Gamez MG, Enciso EA, Maldonado RJ, Palacios DH, Campos SD, Robles IO, Guzman MJ, Diaz AL, Pena CM, Escalera AL. Efficacy and safety of convalescent plasma and intravenous immunoglobulin in critically ill COVID-19 patients. A controlled clinical trial. medRxiv 2021.03.28.21254507; doi: https://doi.org/10.1101/2021.03.28.21254507
68. Figueroa, J.M., Lombardo, M., Dogliotti, A., Flynn, L., Giugliano, R.P., Simonelli, G., Valentini, R., Ramos, A., Romano, P., Marcote, M. and Michelini, A., 2021. Efficacy of a nasal spray containing Iota-Carrageenan in the prophylaxis of COVID-19 in hospital personnel dedicated to patients care with COVID-19 disease. A pragmatic multicenter, randomized, double-blind, placebo-controlled trial (CARR-COV-02). medRxiv 2021.04.13.21255409; doi: https://doi.org/10.1101/2021.04.13.21255409.
69. Chew KW, Moser C, Daar ES, Wohl DA, Li JZ, Coombs R, Ritz J, Giganti M, Javan AC, Li Y, Malvestutto C. Bamlanivimab reduces nasopharyngeal SARS-CoV-2 RNA levels but not symptom duration in non-hospitalized adults with COVID-19: A Phase 2 Randomized Clinical Trial. medRxiv. 2021.12.17.21268009. doi: 10.1101/2021.12.17.21268009.
70. Winthrop KL, Skolnick AW, Rafiq AM, Beegle SH, Suszanski J, Koehne G, Barnett-Griness O, Bibliowicz A, Fathi R, Anderson P, Raday G. Opaganib in COVID-19 pneumonia: Results of a randomized, placebo-controlled Phase 2a trial. medRxiv 2021.08.23.21262464; doi: https://doi.org/10.1101/2021.08.23.21262464
71. Wanaratna K, Leethong P, Inchai N, Chueawiang W, Sriraksa P, Tabmee A, Sirinavin S. Efficacy and safety of Andrographis paniculata extract in patients with mild COVID-19: A randomized controlled trial. medRxiv 2021.07.08.21259912; doi: https://doi.org/10.1101/2021.07.08.21259912
72. Damle L, Damle H, Bharath BR. Plant Formulation ATRICOV 452 in Improving the Level of COVID-19 Specific Inflammatory Markers in Patients. medRxiv 2021.10.07.21264491; doi: https://doi.org/10.1101/2021.10.07.21264491

© 2023 Bai AD et al. *JAMA Network Open*.

73. Huang L, Li Q, Sayed SZ, Mohammad N, Chen B, Iftikhar MA, Xie L, Hu J, Chen H. Ultra–short-wave diathermy shortens the course of moderate and severe COVID-19: a randomized trial. medRxiv 2021.01.28.21250163; doi: https://doi.org/10.1101/2021.01.28.21250163
74. Dunkle LM, Kotloff KL, Gay CL, Anez G, Adelglass JM, Hernandez AQ, Harper WL, Duncanson DM, McArthur MA, Florescu DF, McClelland S. Efficacy and Safety of NVX-CoV2373 in Adults in the United States and Mexico. medRxiv 2021.10.05.21264567; doi: https://doi.org/10.1101/2021.10.05.21264567
75. Maskin LP, Bonelli I, Olarte GL, Palizas F, Velo AE, Lurbet MF, Lovazzano P, Kotsias S, Attie S, Saubidet IL, Baredes ND. High-Versus Low-Dose Dexamethasone for the Treatment of COVID-19-related Acute Respiratory Distress Syndrome: A Multicenter and Randomized Open-label Clinical Trial. medRxiv 2021.09.15.21263597; doi: https://doi.org/10.1101/2021.09.15.21263597
76. Mammen MP, Tebas P, Agnes J, Giffear M, Kraynyak KA, Blackwood E, Amante D, Reuschel EL, Purwar M, Christensen-Quick A, Liu N. Safety and immunogenicity of INO-4800 DNA vaccine against SARS-CoV-2: a preliminary report of a randomized, blinded, placebo-controlled, Phase 2 clinical trial in adults at high risk of viral exposure. medRxiv 2021.05.07.21256652; doi: https://doi.org/10.1101/2021.05.07.21256652
77. Silveira MA, De Jong D, dos Santos Galvão EB, Ribeiro JC, Silva TC, Berretta AA, Amorim TC, San Martin RL, da Conceição LF, Gomes MM, Teixeira MB. Efficacy of propolis as an adjunct treatment for hospitalized COVID-19 patients: a randomized, controlled clinical trial. medRxiv 2021.01.08.20248932; doi: https://doi.org/10.1101/2021.01.08.20248932.
78. Toledo-Romani ME, Sanchez LV, Gonzalez MR, Noda LR, Silva CV, Moreno BP, Ramirez BS, Nicado RP, Mugica RG, Garcia TH, Baez GB. Safety and Immunogenicity of anti-SARS CoV-2 vaccine SOBERANA 02 in homologous or heterologous scheme. medRxiv 2021.11.14.21266309; doi: https://doi.org/10.1101/2021.11.14.21266309
79. Toledo-Romani ME, Garcia-Carmenate M, Silva CV, Baldoquin-Rodriguez W, Perez MM, Gonzalez MC, Moreno BP, Hernandez IM, Romero RG, Tabio OS, Villares PV. Efficacy and Safety of SOBERANA 02, a COVID-19 conjugate vaccine in heterologous three doses combination. medRxiv 2021.10.31.21265703; doi: https://doi.org/10.1101/2021.10.31.21265703
80. Tsilika M, Taks E, Dolianitis K, Kotsaki A, Leventogiannis K, Damoulari C, Kostoula M, Paneta M, Adamis G, Papanikolaou IC, Stamatelopoulos K. Activate-2: a double-blind randomized trial of BCG vaccination against COVID19 in individuals at risk. medRxiv 2021.05.20.21257520; doi: https://doi.org/10.1101/2021.05.20.21257520
81. Holubar M, Subramanian AK, Purington N, Hedlin H, Bunning B, Walter K, Bonilla H, Boumis A, Chen M, Clinton K, Dewhurst L. Favipiravir for treatment of outpatients with asymptomatic or uncomplicated COVID-19: a double-blind randomized, placebo-controlled, phase 2 trial. medRxiv 2021.11.22.21266690; doi: https://doi.org/10.1101/2021.11.22.21266690
82. O'Donnell MR, Grinsztejn B, Cummings MJ, Justman J, Lamb MR, Eckhardt CM, Philip NM, Cheung YK, Gupta V, João E, Pilotto JH. A randomized, double-blind, controlled trial of convalescent plasma in adults with severe COVID-19. medRxiv 2021.03.12.21253373; doi: https://doi.org/10.1101/2021.03.12.21253373
83. O'Brien MP, Forleo-Neto E, Musser BJ, Isa F, Chan KC, Sarkar N, Bar KJ, Barnabas RV, Barouch DH, Cohen MS, Hurt CB. Subcutaneous REGEN-COV Antibody Combination for Covid-19 Prevention. medRxiv 2021.06.14.21258567; doi: https://doi.org/10.1101/2021.06.14.21258567
84. O'Brien MP, Forleo-Neto E, Sarkar N, Isa F, Hou P, Chan KC, Musser BJ, Bar KJ, Barnabas RV, Barouch DH, Cohen MS. Subcutaneous REGEN-COV antibody combination in early SARS-CoV-2 infection. medRxiv 2021.06.14.21258569; doi: https://doi.org/10.1101/2021.06.14.21258569

© 2023 Bai AD et al. *JAMA Network Open*.

85. Puskarich MA, Ingraham NE, Merck LH, Driver BE, Wacker DA, Black LP, Jones AE, Fletcher CV, South AM, Nelson AC, Murray TA. Effect of losartan on hospitalized patients with COVID-19-induced lung injury: A randomized clinical trial. medRxiv 2021.08.25.21262623; doi: https://doi.org/10.1101/2021.08.25.21262623.
86. Bonelli M, Mrak D, Tobudic S, Sieghart D, Koblischke M, Mandl P, Kornek B, Simader E, Radner H, Perkmann T, Haslacher H. Additional heterologous versus homologous booster vaccination in immunosuppressed patients without SARS-CoV-2 antibody seroconversion after primary mRNA vaccination: a randomized controlled trial. medRxiv 2021.09.05.21263125; doi: https://doi.org/10.1101/2021.09.05.21263125
87. Sholzberg M, Tang GH, Rahhal H, AlHamzah M, Kreuziger LB, Áinle FN, Alomran F, Alayed K, AlSheef M, AlSumait F, Pompilio CE. Heparin for moderately ill patients with Covid-19. medRxiv 2021.07.08.21259351; doi: https://doi.org/10.1101/2021.07.08.21259351
88. Li M, Yang J, Wang L, Wu Q, Wu Z, Zheng W, Wang L, Lu W, Deng X, Peng C, Han B. A booster dose is immunogenic and will be needed for older adults who have completed two doses vaccination with CoronaVac: a randomised, double-blind, placebo-controlled, phase 1/2 clinical trial. medRxiv 2021.08.03.21261544; doi: https://doi.org/10.1101/2021.08.03.21261544
89. Elgohary MA, Hasan EM, Ibrahim AA, Abdelsalam MF, Abdel-Rahman RZ, Zaki AI, Elaatar MB, Elnagar MT, Emam ME, Hamada MM, Abdel-Hamid TM. Efficacy of Sofosbuvir plus Ledipasvir in Egyptian patients with COVID-19 compared to standard treatment: a randomized controlled trial. medRxiv 2021.05.19.21257429; doi: https://doi.org/10.1101/2021.05.19.21257429
90. Javaherian M, Shadmehr A, Keshtkar A, Beigmohammadi MT, Dabbaghipour N, Syed A, Moghaddam BA. Safety and efficacy of Pulmonary physiotherapy in hospitalized patients with severe COVID-19 pneumonia (PPTCOVID): A prospective, randomised, single-blind, controlled trial. medRxiv 2021.04.24.21255892; doi: https://doi.org/10.1101/2021.04.24.21255892.
91. Formica N, Mallory R, Albert G, Robinson M, Plested J, Cho I, Robertson A, Dubovsky F, Glenn GM. Evaluation of a SARS-CoV-2 vaccine NVX-CoV2373 in younger and older adults. medRxiv 2021.02.26.21252482; doi: https://doi.org/10.1101/2021.02.26.21252482
92. Morici N, Podda GM, Birocchi S, Bonacchini L, Merli M, Trezzi M, Massaini G, Agostinis M, Carioti G, Serino FS, Gazzaniga G. Enoxaparin for thromboprophylaxis in hospitalized COVID-19 patients: comparison of 40 mg od vs 40 mg bid The X-COVID19 Randomized Clinical Trial. medRxiv 2021.11.17.21266488; doi: https://doi.org/10.1101/2021.11.17.21266488
93. Holubovska O, Bojkova D, Elli S, Bechtel M, Boltz D, Muzzio M, Peng X, Sala F, Cosentino C, Mironenko A, Milde J. Enisamium is an inhibitor of the SARS-CoV-2 RNA polymerase and shows improvement of recovery in COVID-19 patients in an interim analysis of a clinical trial. medRxiv 2021.01.05.21249237; doi: https://doi.org/10.1101/2021.01.05.21249237
94. Babalola OE, Bode CO, Ajayi AA, Alakaloko FM, Akase IE, Otrofanowei E, Salu OB, Adeyemo WL, Ademuyiwa AO, Omilabu S. Ivermectin shows clinical benefits in mild to moderate COVID19: A randomised controlled double blind dose response study in Lagos. medRxiv 2021.01.05.21249131; doi: https://doi.org/10.1101/2021.01.05.21249131
95. Bhardwaj P, Godatwar PK, Charan J, Sharma S, Shafi S, Chauhan N, Vyas P, Dutt N, Midha N, Jalandra R, Sharma M. Efficacy and Safety of Ayurveda Intervention AYUSH 64 as add-on therapy for patients with COVID 19 infections: An open labelled, Parallel Group, Randomized controlled clinical trial. medRxiv 2021.08.10.21261836; doi: https://doi.org/10.1101/2021.08.10.21261836
96. The ATTACC, ACTIV-4a, and REMAP-CAP Investigators, Patrick R. Lawler, Ewan C. Goligher, Jeffrey S. Berger, Matthew D. Neal, Bryan J. McVerry, Jose C. Nicolau, Michelle N. Gong, Marc Carrier, Robert S. Rosenson, Harmony R. Reynolds, Alexis F. Turgeon, Jorge Escobedo, David T. Huang, Charlotte Ann Bradbury, Brett L. Houston, Lucy Z. Kornblith, Anand Kumar, Susan R. Kahn, Mary Cushman, Zoe McQuilten, Arthur S. Slutsky, Keri S. Kim, Anthony C. Gordon, Bridget-Anne Kirwan, Maria M. Brooks, Alisa M. Higgins, Roger J. Lewis, Elizabeth Lorenzi, Scott

© 2023 Bai AD et al. *JAMA Network Open*.

M. Berry, Lindsay R. Berry, Derek C. Angus, Colin J. McArthur, Steven A. Webb, Michael E. Farkouh, Judith S. Hochman, Ryan Zarychanski. Therapeutic Anticoagulation in Non-Critically Ill Patients with Covid-19 medRxiv 2021.05.13.21256846; doi: https://doi.org/10.1101/2021.05.13.21256846.

97. Goepfert PA, Fu B, Chabanon AL, Bonaparte MI, Davis MG, Essink BJ, Frank I, Haney O, Janosczyk H, Keefer MC, Koutsoukos M. Safety and immunogenicity of SARS-CoV-2 recombinant protein vaccine formulations in healthy adults: a randomised, placebo-controlled, dose-ranging study. medRxiv 2021.01.19.20248611; doi: https://doi.org/10.1101/2021.01.19.20248611
98. Gutierrez-Castrellon P, Gandara-Martí T, y Abreu AT, Nieto-Rufino CD, Lopez-Orduna E, Jiménez-Escobar I, Jiménez-Gutiérrez C, Lopez-Vazquez G, Espadaler-Mazo J. Efficacy and safety of novel probiotic formulation in adult Covid19 outpatients: a randomized, placebo-controlled clinical trial. medRxiv 2021.05.20.21256954; doi: https://doi.org/10.1101/2021.05.20.21256954
99. RECOVERY Collaborative Group. Aspirin in patients admitted to hospital with COVID-19 (RECOVERY): a randomised, controlled, open-label, platform trial. medRxiv 2021.06.08.21258132; doi: https://doi.org/10.1101/2021.06.08.21258132
100. RECOVERY Collaborative Group. Convalescent plasma in patients admitted to hospital with COVID-19 (RECOVERY): a randomised, controlled, open-label, platform trial. medRxiv 2021.03.09.21252736; doi: https://doi.org/10.1101/2021.03.09.21252736
101. RECOVERY Collaborative Group. Casirivimab and imdevimab in patients admitted to hospital with COVID-19 (RECOVERY): a randomised, controlled, open-label, platform trial. medRxiv 2021.06.15.21258542; doi: https://doi.org/10.1101/2021.06.15.21258542
102. RECOVERY Collaborative Group. Colchicine in patients admitted to hospital with COVID-19 (RECOVERY): a randomised, controlled, open-label, platform trial. medRxiv 2021.05.18.21257267; doi: https://doi.org/10.1101/2021.05.18.21257267
103. RECOVERY Collaborative Group. Tocilizumab in patients admitted to hospital with COVID-19 (RECOVERY): preliminary results of a randomised, controlled, open-label, platform trial. medRxiv 2021.02.11.21249258; doi: https://doi.org/10.1101/2021.02.11.21249258
104. Gobeil P, Pillet S, Séguin A, Boulay I, Mahmood A, Vinh DC, Charland N, Boutet P, Roman FP, Van Der Most R, Perez MD. Interim report of a phase 2 randomized trial of a plant-produced virus-like particle vaccine for Covid-19 in healthy adults aged 18-64 and older adults aged 65 and older. medRxiv 2021.05.14.21257248; doi: https://doi.org/10.1101/2021.05.14.21257248
105. Bégin P, Callum J, Jamula E, Cook R, Heddle NM, Tinmouth A, Zeller MP, Beaudoin-Bussières G, Amorim L, Bazin R, Loftsgard KC. Convalescent plasma for hospitalized patients with COVID-19 and the effect of plasma antibodies: a randomized controlled, open-label trial. medRxiv 2021.06.29.21259427; doi: https://doi.org/10.1101/2021.06.29.21259427
106. Mallory R, Formica N, Pfeiffer S, Wilkinson B, Marcheschi A, Albert G, McFall H, Robinson M, Plested J, Zhu M, Cloney-Clark S. Immunogenicity and Safety Following a Homologous Booster Dose of a SARS-CoV-2 recombinant spike protein vaccine (NVX-CoV2373): A Phase 2 Randomized Placebo-Controlled Trial. medRxiv 2021.12.23.21267374; doi: https://doi.org/10.1101/2021.12.23.21267374
107. Ella R, Reddy S, Blackwelder W, Potdar V, Yadav P, Sarangi V, Aileni VK, Kanungo S, Rai S, Reddy P, Verma S. Efficacy, safety, and lot to lot immunogenicity of an inactivated SARS-CoV-2 vaccine (BBV152): a double-blind, randomised, controlled phase 3 trial. medRxiv 2021.06.30.21259439; doi: https://doi.org/10.1101/2021.06.30.21259439
108. Lazarus R, Taucher C, Duncan C, Faust S, Green CA, Finn A. Immunogenicity and safety of inactivated whole virion Coronavirus vaccine with CpG (VLA2001) in healthy adults aged 18 to 55: a randomised phase 1/2 clinical trial. medRxiv 2021.08.13.21262021; doi: https://doi.org/10.1101/2021.08.13.21262021

© 2023 Bai AD et al. *JAMA Network Open*.

109. Amaravadi RK, Giles L, Carberry M, Hyman MC, Frank I, Nasta SD, Walsh J, Wileyto EP, Gimotty P, Milone M, Teng EM. Hydroxychloroquine for SARS-CoV-2 positive patients quarantined at home: The first interim analysis of a remotely conducted randomized clinical trial. medRxiv 2021.02.22.21252228; doi: https://doi.org/10.1101/2021.02.22.21252228
110. Ravakirti, Roy R, Pattadar C, Raj R, Agarwal N, Biswas B, Majhi PK, Rai DK, Kumar A, Sarfaraz A. Ivermectin as a potential treatment for mild to moderate COVID-19–a double blind randomized placebo-controlled trial. medRxiv 2021.01.05.21249310; doi: https://doi.org/10.1101/2021.01.05.21249310
111. Ravichandran R, Mohan SK, Sukumaran SK, Kamaraj D, Daivasuga SS, Ravi SO, Vijayaraghavalu S, Kumar RK. Use of Indomethacin for mild and moderate Covid-19 patients A Randomized Control Trial. medRxiv 2021.07.24.21261007; doi: https://doi.org/10.1101/2021.07.24.21261007
112. ElZein R, Fakhreddine S, Abi Hanna P, Feghali R, Hamad H, Ayoub F. In vivo evaluation of the virucidal efficacy of Chlorhexidine and Povidone-iodine mouthwashes against salivary SARS-CoV-2. medRxiv 2021.03.07.21252302; doi: https://doi.org/10.1101/2021.03.07.21252302
113. Sablerolles R, Rietdijk W, Goorhuis B, Postma D, Visser L, Geers D, Schmitz K, Garrido HG, Koopmans M, Dalm V, Kootstra NA. Immunogenicity and reactogenicity of booster vaccinations after Ad26. COV2. S priming. medRxiv 2021.10.18.21264979; doi: https://doi.org/10.1101/2021.10.18.21264979
114. Chahla RE, Ruiz LM, Ortega ES, Morales MF, Barreiro F, George A, Mansilla C, D'Amato SP, Barrenechea G, Goroso GD. A randomized trial-intensive treatment based in ivermectin and iota-carrageenan as pre-exposure prophylaxis for COVID-19 in healthcare agents. medRxiv 2021.03.26.21254398; doi: https://doi.org/10.1101/2021.03.26.21254398
115. Chahla RE, Ruiz LM, Mena T, Brepe Y, Terranova P, Ortega ES, Barrenechea GG, Goroso DG. Ivermectin reproposing for COVID-19 treatment outpatients in mild stage in primary health care centers. medRxiv 2021.03.29.21254554; doi: https://doi.org/10.1101/2021.03.29.21254554
116. Izikson R, Brune D, Bolduc JS, Bourron P, Fournier M, Moore TM, Pandey A, Perez L, Sater N, Shrestha A, Wague S. Safety and immunogenicity of a high-dose quadrivalent influenza vaccine administered concomitantly with a third dose of the mRNA-1273 SARS-CoV-2 vaccine in adults ≥ 65 years of age: a Phase II, open-label study. medRxiv 2021.10.29.21265248; doi: https://doi.org/10.1101/2021.10.29.21265248
117. Ramakrishnan S, Nicolau Jr DV, Langford B, Mahdi M, Jeffers H, Mwasuku C, Krassowska K, Fox R, Binnian I, Glover V, Bright S. Inhaled budesonide in the treatment of early COVID-19 illness: a randomised controlled trial. medRxiv 2021.02.04.21251134; doi: https://doi.org/10.1101/2021.02.04.21251134
118. Sridhar S, Arnel J, Bonaparte MI, Bueso A, Chabanon AL, Chen A, Chicz RM, Diemert D, Essink BJ, Fu B, Grunenberg NA. Safety and immunogenicity of a SARS-CoV-2 recombinant protein vaccine with AS03 adjuvant in healthy adults: interim findings from a phase 2, randomised, dose-finding, multi-centre study. medRxiv 2021.10.08.21264302; doi: https://doi.org/10.1101/2021.10.08.21264302
119. Somersan-Karakaya S, Mylonakis E, Menon VP, Wells JC, Ali S, Sivapalasingam S, Sun Y, Bhore R, Mei J, Miller J, Cupelli L. REGEN-COV® for Treatment of Hospitalized Patients with Covid-19. medRxiv 2021.11.05.21265656; doi: https://doi.org/10.1101/2021.11.05.21265656
120. Fragoso-Saavedra S, Núñez I, Audelo-Cruz BM, Arias-Martínez S, Manzur-Sandoval D, Quintero-Villegas A, García-González HB, Carbajal-Morelos SL, de León-Rosales SP, Gotés-Palazuelos J, Caro-Vega Y. Pyridostigmine in adults with severe SARS-CoV-2 infection: the PISCO trial. medRxiv 2021.04.28.21255834; doi: https://doi.org/10.1101/2021.04.28.21255834
121. Heath PT, Galiza EP, Baxter DN, Boffito M, Browne D, Burns F, Chadwick DR, Clark R, Cosgrove C, Galloway J, Goodman AL, Heer A, Higham A, Iyengar S, Jamal A, Jeanes C, Kalra PA, Kyriakidou C, McAuley DF, Meyrick A, Minassian AM, Minton J, Moore P, Munsoor I, Nicholls H, Osanlou O, Packham J, Pretswell CH, San Francisco Ramos A, Saralaya D, Sheridan RP, Smith R, Soiza RL, Swift PA, Thomson EC, Turner J, Viljoen ME,

© 2023 Bai AD et al. *JAMA Network Open*.

Albert G, Cho I, Dubovsky F, Glenn G, Rivers J, Robertson A, Smith K, Toback S; 2019nCoV-302 Study Group. Efficacy of the NVX-CoV2373 Covid-19 Vaccine Against the B.1.1.7 Variant. medRxiv 2021.05.13.21256639; doi: https://doi.org/10.1101/2021.05.13.21256639
122. Panda PK, SEV COVID trial group. Safety and efficacy of antiviral therapy alone or in combination in COVID-19-a randomized controlled trial (SEV COVID Trial). medRxiv 2021.06.06.21258091; doi: https://doi.org/10.1101/2021.06.06.21258091
123. Madhi SA, Baillie VL, Cutland CL, Voysey M, Koen AL, Fairlie L, Padayachee SD, Dheda K, Barnabas SL, Bhorat QE, Briner C. Safety and efficacy of the ChAdOx1 nCoV-19 (AZD1222) Covid-19 vaccine against the B. 1.351 variant in South Africa. medRxiv 2021.02.10.21251247; doi: https://doi.org/10.1101/2021.02.10.21251247
124. Shenoy S, Munjal S, Al Youha S, Alghounaim M, Almazeedi S, Alshamali Y, Kaszynski RH, Al-Sabah S, Kuwait Clinical Trial Group. Favipiravir In Adults with Moderate to Severe COVID-19: A Phase 3 Multicentre, Randomized, Double-Blinded, Placebo-Controlled Trial. medRxiv 2021.11.08.21265884; doi: https://doi.org/10.1101/2021.11.08.21265884
125. Shoham S, Bloch EM, Casadevall A, Hanley D, Lau B, Gebo K, Cachay E, Kassaye SG, Paxton JH, Gerber J, Levine AC. Randomized controlled trial transfusing convalescent plasma as post-exposure prophylaxis against SARS-CoV-2 infection. medRxiv 2021.12.13.21267611; doi: https://doi.org/10.1101/2021.12.13.21267611
126. Méndez-Flores S, Priego-Ranero Á, Azamar-Llamas D, Olvera-Prado H, Rivas-Redondo KI, Ochoa-Hein E, Perez-Ortiz A, Rojas-Castañeda E, Urbina-Terán S, Septién-Stute L, Hernández-Gilsoul T. Effect of polymerized type I collagen in hyperinflammation of adult outpatients with symptomatic COVID-19: a double blind, randomised, placebo-controlled clinical trial. medRxiv 2021.05.12.21257133; doi: https://doi.org/10.1101/2021.05.12.21257133
127. Körper S, Weiss M, Zickler D, Wiesmann T, Zacharowski K, Corman VM, Grüner B, Ernst L, Spieth P, Lepper PM, Bentz M. High dose convalescent plasma in COVID-19: results from the randomized trial CAPSID. medRxiv 2021.05.10.21256192; doi: https://doi.org/10.1101/2021.05.10.21256192
128. Betka SJ, Kannape OA, Fasola J, Lance F, Cardin S, Schmid A, Similowski T, Soccal MP, Herbelin B, Adler D, Blanke O. Virtual reality exercise to help COVID patients with refractory breathlessness. medRxiv 2021.10.26.21265510; doi: https://doi.org/10.1101/2021.10.26.21265510
129. Thomas SJ, Moreira ED, Kitchin N, Absalon J, Gurtman A, Lockhart S, Perez JL, Marc GP, Polack FP, Zerbini C, Bailey R. Six Month safety and efficacy of the BNT162b2 Mrna Covid-19 vaccine. medRxiv 2021.07.28.21261159; doi: https://doi.org/10.1101/2021.07.28.21261159
130. Sivapalasingam S, Lederer D, Bhore R, Hajizadeh N, Criner G, Hosain R, Mahmood A, Giannelou A, Somersan-Karakaya S, O'Brien M, Boyapati A. A randomized placebo-controlled trial of sarilumab in hospitalized patients with Covid-19. medRxiv 2021.05.13.21256973; doi: https://doi.org/10.1101/2021.05.13.21256973
131. Patankar SB, Rangnekar H, Joshi K, Suryawanshi K, Soni P, Gorde A, Shah T, Patankar S, Jha D, Raje R. Efficacy and Safety of Polyherbal formulation as an add-on to the standard of care in mild to moderate COVID-19: A randomized, double-blind, placebo-controlled trial. medRxiv 2021.05.14.21256900; doi: https://doi.org/10.1101/2021.05.14.21256900
132. Naggie S, Milstone A, Castro M, Collins SP, Seetha L, Anderson DJ, Cahuayme-Zuniga L, Batey-Turner K, Cohen LW, Fraulo E, Friedland A. Hydroxychloroquine for pre-exposure prophylaxis of COVID-19 in health care workers: a randomized, multicenter, placebo-controlled trial (HERO-HCQ). medRxiv 2021.08.19.21262275; doi: https://doi.org/10.1101/2021.08.19.21262275
133. Bukhari SK, Asghar A, Perveen N, Hayat A, Mangat SA, Butt KR, Abdullah M, Fatima T, Mustafa A, Cheema T. Efficacy of ivermectin in COVID-19 patients with mild to moderate disease. medRxiv 2021.02.02.21250840; doi: https://doi.org/10.1101/2021.02.02.21250840

© 2023 Bai AD et al. *JAMA Network Open*.

134. Hsieh SM, Liu MC, Chen YH, Lee WS, Hwang SJ, Cheng SH, Ko WC, Hwang KP, Wang NC, Lee YL, Lin YL. Safety and immunogenicity of CpG 1018 and aluminium hydroxide-adjuvanted SARS-CoV-2 S-2P protein vaccine MVC-COV1901: a large-scale double-blind, randomised, placebo-controlled phase 2 trial. medRxiv 2021.08.05.21261532; doi: https://doi.org/10.1101/2021.08.05.21261532
135. Munch MW, Myatra SN, Vijayaraghavan BK, Saseedharan S, Benfield T, Wahlin RR, Rasmussen BS, Andreasen AS, Poulsen LM, Cioccari L, Khan MS. Dexamethasone 12 mg versus 6 mg for patients with COVID-19 and severe hypoxia: an international, randomized, blinded trial. medRxiv 2021.07.22.21260755; doi: https://doi.org/10.1101/2021.07.22.21260755
136. Dorward J, Yu LM, Hayward G, Saville BR, Gbinigie O, Van Hecke O, Ogburn E, Evans PH, Thomas NP, Patel MG, Richards D. Colchicine for COVID-19 in adults in the community (PRINCIPLE): a randomised, controlled, adaptive platform trial. medRxiv 2021.09.20.21263828; doi: https://doi.org/10.1101/2021.09.20.21263828
137. Yu LM, Bafadhel M, Dorward J, Hayward G, Saville BR, Gbinigie O, Van Hecke O, Ogburn E, Evans PH, Thomas NP, Patel MG. Inhaled budesonide for COVID-19 in people at higher risk of adverse outcomes in the community: interim analyses from the PRINCIPLE trial. medRxiv 2021.04.10.21254672; doi: https://doi.org/10.1101/2021.04.10.21254672
138. REMAP-CAP Investigators. Interleukin-6 Receptor Antagonists in Critically Ill Patients with Covid-19–Preliminary report. medRxiv 2021.01.07.21249390; doi: https://doi.org/10.1101/2021.01.07.21249390
139. Derde LP, REMAP-CAP Investigators. Effectiveness of tocilizumab, sarilumab, and anakinra for critically ill patients with COVID-19 the REMAP-CAP COVID-19 immune modulation therapy domain randomized clinical trial. medRxiv 2021.06.18.21259133; doi: https://doi.org/10.1101/2021.06.18.21259133
140. REMAP-CAP Investigators. Convalescent plasma in critically ill patients with Covid-19. medRxiv 2021.06.11.21258760; doi: https://doi.org/10.1101/2021.06.11.21258760
141. Weinreich DM, Sivapalasingam S, Norton T, Ali S, Gao H, Bhore R, Hooper AT, Hamilton JD, Musser BJ, Soo Y, Rofail D. REGEN-COV antibody cocktail in outpatients with Covid-19. medRxiv 2021.06.09.21257915; doi: https://doi.org/10.1101/2021.06.09.21257915
142. Nguyen TP, Do Q, Phan LT, Dinh DV, Khong H, Hoang LV, Nguyen TV, Pham HN, Chu MV, Nguyen TT, Le TM. Safety and Immunogenicity of Nanocovax, a SARS-CoV-2 Recombinant Spike Protein Vaccine. medRxiv 2021.07.22.21260942; doi: https://doi.org/10.1101/2021.07.22.21260942
143. Hinks TS, Cureton L, Knight R, Wang A, Cane JL, Barber VS, Black J, Dutton SJ, Melhorn J, Jabeen M, Moss P. A randomised clinical trial of azithromycin versus standard care in ambulatory COVID-19-the ATOMIC2 trial. medRxiv 2021.04.21.21255807; doi: https://doi.org/10.1101/2021.04.21.21255807
144. Reis G, dos Santos Moreira-Silva EA, Silva DC, Thabane L, Milagres AC, Ferreira TS, Dos Santos CV, de Souza Campos VH, Nogueira AM, de Almeida AP, Callegari ED. Effect of early treatment with fluvoxamine on risk of emergency care and hospitalisation among patients with COVID-19: the TOGETHER randomised, platform clinical trial. medRxiv 2021.08.19.21262323; doi: https://doi.org/10.1101/2021.08.19.21262323
145. Quinn TM, Gaughan EE, Bruce A, Antonelli J, O'Connor R, Li F, McNamara S, Koch O, MacIntosh C, Dockrell D, Walsh T. Randomised Controlled Trial of Intravenous Nafamostat Mesylate in COVID pneumonitis: Phase 1b/2a Experimental Study to Investigate Safety, Pharmacokinetics and Pharmacodynamics. medRxiv 2021.10.06.21264648; doi: https://doi.org/10.1101/2021.10.06.21264648

© 2023 Bai AD et al. *JAMA Network Open*.

146. Marconi VC, Ramanan AV, de Bono S, Kartman C, Krishnan V, Liao R, Piruzeli ML, Goldman JD, Alatorre-Alexander J, de Cassia Pellegrini R, Estrada V. Baricitinib plus standard of care for hospitalized adults with COVID-19. medRxiv 2021.04.30.21255934; doi: https://doi.org/10.1101/2021.04.30.21255934
147. Shinde V, Bhikha S, Hossain Z, Archary M, Bhorat Q, Fairlie L, Lalloo U, Masilela ML, Moodley D, Hanley S, Fouche L. Preliminary Efficacy of the NVX-CoV2373 Covid-19 Vaccine Against the B. 1.351 Variant. medRxiv 2021.02.25.21252477; doi: https://doi.org/10.1101/2021.02.25.21252477
148. Trieu V, Saund S, Rahate P, Barge V, Nalk S, Windlass H, Uckun F. Targeting TGF-b pathway with COVID-19 Drug Candidate ARTIVeda/PulmoHeal Accelerates Recovery from Mild-Moderate COVID-19. medRxiv 2021.01.24.21250418; doi: https://doi.org/10.1101/2021.01.24.21250418
149. Fischer WA, Eron JJ, Holman W, Cohen MS, Fang L, Szewczyk LJ, Sheahan TP, Baric RS, Mollan KR, Wolfe CR, Duke ER. Molnupiravir, an oral antiviral treatment for COVID-19. medRxiv 2021.06.17.21258639; doi: https://doi.org/10.1101/2021.06.17.21258639
150. Feng Y, Chen J, Yao T, Chang Y, Li X, Xing R, Li H, Xie R, Zhang X, Wei Z, Mu S. Safety and Immunogenicity of Inactivated SARS-CoV-2 Vaccine in High-Risk Occupational Population: a randomized, parallel, controlled clinical trial. medRxiv 2021.08.06.21261696; doi: https://doi.org/10.1101/2021.08.06.21261696
151. Temesgen Z, Burger CD, Baker J, Polk C, Libertin C, Kelley C, Marconi VC, Orenstein R, Durrant C, Chappell D, Ahmed O. LENZILUMAB EFFICACY AND SAFETY IN NEWLY HOSPITALIZED COVID-19 SUBJECTS: RESULTS FROM THE LIVE-AIR PHASE 3 RANDOMIZED DOUBLE-BLIND PLACEBO-CONTROLLED TRIAL. medRxiv 2021.05.01.21256470; doi: https://doi.org/10.1101/2021.05.01.21256470
152. Pan H, Wu Q, Zeng G, Yang J, Jiang D, Deng X, Chu K, Zheng W, Zhu F, Yu H, Yin W. Immunogenicity and safety of a third dose, and immune persistence of CoronaVac vaccine in healthy adults aged 18-59 years: interim results from a double-blind, randomized, placebo-controlled phase 2 clinical trial. MedRxiv. 2021.07.23.21261026; doi: https://doi.org/10.1101/2021.07.23.21261026

© 2023 Bai AD et al. *JAMA Network Open*.

eTable 2. Risk of Bias Assessment

	Domain 1: Randomization process	Domain 2: Deviations from intended interventions	Domain 3: Missing outcome data	Domain 4: Measurement of outcome	Domain 5: Selection of the reported result	Overall risk of bias
Abdulamir [1]	some concerns	some concerns	low risk	low risk	low risk	some concerns
Lima [2]	some concerns	low risk	high risk	low risk	some concerns	high risk
Hosseinzadeh [3]	low risk	some concerns	low risk	low risk	low risk	some concerns
Shiri [4]	some concerns	low risk	low risk	low risk	low risk	some concerns
Baxter [5]	low risk	some concerns	low risk	some concerns	low risk	some concerns
Bhatt [6]	low risk	some concerns	some concerns	low risk	low risk	some concerns
Gupta [7]	low risk	low risk	low risk	low risk	low risk	low risk
Gupta [8]	low risk	low risk	low risk	low risk	low risk	low risk
Gupta [9]	some concerns	some concerns	low risk	low risk	some concerns	some concerns
Chopra [10]	low risk	some concerns	high risk	low risk	low risk	high risk
Biber [11]	low risk	low risk	low risk	low risk	low risk	low risk
Fisher [12]	low risk	low risk	some concerns	low risk	low risk	some concerns
Gaborit [13]	low risk	low risk	low risk	low risk	low risk	low risk
Young [14][a]	low risk	some concerns	some concerns	low risk	low risk	some concerns
Clemency [15]	low risk	low risk	low risk	low risk	low risk	low risk
Araujo [16]	low risk	low risk	low risk	low risk	low risk	low risk
Tornero [17]	some concerns	some concerns	some concerns	some concerns	high risk	high risk
Hernandez-Cardenas [18]	low risk	low risk	low risk	low risk	low risk	low risk
Mok [19]	some concerns	some concerns	low risk	low risk	low risk	some concerns
Portal-Celhay [20]	some concerns	low risk	low risk	low risk	low risk	some concerns
Singh [21]	some concerns	low risk	low risk	low risk	low risk	some concerns
Sullivan [22]	low risk	low risk	low risk	low risk	low risk	low risk
Huang [23]	low risk	some concerns	low risk	low risk	low risk	some concerns
Weinreich [24]	low risk	low risk	low risk	low risk	low risk	low risk
Parikh [25]	some concerns	low risk	low risk	low risk	some concerns	some concerns
Ely [26]	low risk	low risk	low risk	low risk	low risk	low risk
Fedrizzi [27]	low risk	low risk	low risk	low risk	low risk	low risk
Balint [28]	low risk	some concerns	low risk	some concerns	low risk	some concerns
Kolev [29]	low risk	some concerns	low risk	low risk	low risk	some concerns
Breza [30][a]	low risk	low risk	low risk	low risk	low risk	low risk
Lattman [31]	some concerns	low risk	low risk	low risk	low risk	some concerns
Gaughan [32]	low risk	low risk	low risk	low risk	some concerns	some concerns
McCreary [33]	some concerns	some concerns	low risk	low risk	low risk	some concerns
Sobngwi [34]	low risk	low risk	low risk	high risk	low risk	high risk
Kyriazopoulou [35]	low risk	low risk	low risk	low risk	low risk	low risk
Mikhaylov [36]	some concerns	low risk	low risk	some concerns	low risk	some concerns
Goligher [37]	low risk	low risk	low risk	low risk	low risk	low risk
Syed [38]	some concerns	low risk	low risk	high risk	high risk	high risk
Cadegiani [39]	low risk	low risk	low risk	low risk	low risk	low risk
Cadegiani [40]	high risk	low risk	low risk	low risk	low risk	high risk
Ader [41]	low risk	low risk	some concerns	low risk	low risk	some concerns
Fralick [42]	low risk	low risk	some concerns	low risk	some concerns	some concerns
Hernandez-Bernal [43]	low risk	low risk	low risk	low risk	low risk	low risk

© 2023 Bai AD et al. *JAMA Network Open*.

Lescure [44]	low risk	low risk	low risk	low risk	low risk	low risk
Perkins [45]	low risk	low risk	low risk	low risk	high risk	high risk
Roozen [46]	low risk	some concerns	low risk	some concerns	low risk	some concerns
Tornling [47]	low risk	low risk	low risk	low risk	low risk	low risk
Resende [48]	some concerns	low risk	low risk	low risk	low risk	some concerns
Dai [49]	some concerns	low risk	low risk	low risk	low risk	some concerns
Gaitan-Duarte [50]	low risk	low risk	low risk	low risk	low risk	low risk
Arnardottir [51]	low risk	low risk	some concerns	low risk	low risk	some concerns
Pan [52]	low risk	low risk	low risk	low risk	low risk	low risk
Almanza-Reyes [53]	low risk	high risk	some concerns	some concerns	low risk	high risk
Faramarzi [54]	low risk	low risk	high risk	some concerns	low risk	high risk
Patel [55]	low risk	low risk	low risk	low risk	low risk	low risk
Tardif [56]	low risk	low risk	low risk	low risk	some concerns	some concerns
Rossignol [57]	low risk	low risk	low risk	low risk	low risk	low risk
Low [58]	some concerns	low risk	low risk	low risk	low risk	some concerns
Lundgren [59]	low risk	low risk	low risk	low risk	low risk	low risk
Liu [60]	low risk	high risk	low risk	low risk	low risk	high risk
Li [61]	low risk	low risk	low risk	low risk	low risk	low risk
Dupuis [62]	low risk	low risk	low risk	low risk	some concerns	some concerns
Haran [63]	low risk	low risk	low risk	high risk	low risk	high risk
Song [64]	some concerns	some concerns	low risk	low risk	low risk	some concerns
Rojas-Serrano [65]	low risk	low risk	some concerns	low risk	some concerns	some concerns
Beltran-Gonzalez [66]	some concerns	low risk	some concerns	low risk	low risk	some concerns
Gonzalez [67]	some concerns	high risk	some concerns	low risk	high risk	high risk
Figueroa [68]	low risk	low risk	low risk	low risk	low risk	low risk
Chew [69]	low risk	low risk	low risk	low risk	some concerns	some concerns
Winthrop [70]	low risk	low risk	high risk	low risk	high risk	high risk
Wanaratna [71]	low risk	low risk	some concerns	low risk	low risk	some concerns
Damle [72]	some concerns	some concerns	some concerns	low risk	some concerns	high risk
Huang [73]	low risk	low risk	low risk	low risk	low risk	low risk
Dunkle [74]	low risk	some concerns	low risk	low risk	low risk	some concerns
Maskin [75]	low risk	low risk	low risk	low risk	low risk	low risk
Mammen [76]	some concerns	low risk	low risk	low risk	low risk	some concerns
Silveira [77]	low risk	low risk	low risk	some concerns	low risk	some concerns
Toledo-Romani [78]	high risk	low risk	low risk	low risk	low risk	high risk
Toledo-Romani [79]	some concerns	low risk	low risk	low risk	low risk	some concerns
Tsilika [80]	some concerns	low risk	high risk	high risk	high risk	high risk
Holubar [81]	low risk	low risk	low risk	low risk	low risk	low risk
O'Donnell [82]	low risk	low risk	low risk	low risk	low risk	low risk
O'Brien [83]	low risk	low risk	low risk	low risk	some concerns	some concerns
O'Brien [84]	low risk	low risk	low risk	low risk	some concerns	some concerns
Puskarich [85]	low risk	low risk	some concerns	low risk	low risk	some concerns
Bonelli [86]	low risk	low risk	low risk	low risk	low risk	low risk
Sholzberg [87]	low risk	low risk	low risk	low risk	low risk	low risk
Li [88]	low risk	low risk	low risk	low risk	low risk	low risk
Elgohary [89]	low risk	low risk	low risk	high risk	low risk	high risk
Javaherian [90]	some concerns	low risk	low risk	low risk	low risk	some concerns
Formica [91]	low risk	low risk	low risk	low risk	some concerns	some concerns

© 2023 Bai AD et al. *JAMA Network Open*.

Morici [92]	low risk	low risk	low risk	high risk	high risk	high risk
Holubovska [93]	some concerns	low risk	some concerns	some concerns	some concerns	high risk
Babalola [94]	some concerns	low risk	low risk	low risk	some concerns	some concerns
Bhardwaj [95]	low risk	some concerns	low risk	low risk	low risk	some concerns
ATTACC investigators [96]	low risk	low risk	low risk	low risk	low risk	low risk
Goepfert [97]	low risk	low risk	low risk	low risk	low risk	low risk
Gutierrez-Castrellon [98]	low risk	low risk	low risk	low risk	low risk	low risk
RECOVERY Collaborative Group [99]	low risk	low risk	low risk	low risk	some concerns	some concerns
RECOVERY Collaborative Group [100]	low risk	some concerns	low risk	low risk	low risk	some concerns
RECOVERY Collaborative Group [101]	low risk	low risk	low risk	low risk	low risk	low risk
RECOVERY Collaborative Group [102]	low risk	low risk	low risk	low risk	low risk	low risk
RECOVERY Collaborative Group [103]	low risk	some concerns	low risk	low risk	low risk	some concerns
Gobeil [104]	low risk	low risk	low risk	low risk	low risk	low risk
Begin [105]	low risk	low risk	low risk	low risk	low risk	low risk
Mallory [106]	low risk	low risk	some concerns	low risk	low risk	some concerns
Ella [107]	low risk	low risk	low risk	low risk	low risk	low risk
Lazarus [108]	low risk	low risk	some concerns	low risk	low risk	some concerns
Amaravadi [109]	low risk	high risk	high risk	high risk	low risk	high risk
Ravikirti [110]	low risk	low risk	low risk	low risk	low risk	low risk
Ravichandran [111]	low risk	some concerns	some concerns	low risk	some concerns	some concerns
Elzein [112]	some concerns	low risk	low risk	low risk	low risk	some concerns
Sablerolles [113]	some concerns	low risk	low risk	low risk	low risk	some concerns
Chahla [114]	low risk	low risk	low risk	some concerns	low risk	some concerns
Chahla [115]	high risk	low risk	high risk	high risk	low risk	high risk
Izikson [116]	low risk	high risk	low risk	low risk	low risk	high risk
Ramakrishnan [117]	low risk	low risk	low risk	low risk	low risk	low risk
Sridhar [118]	low risk	low risk	low risk	low risk	low risk	low risk
Somersan-Karakaya [119]	some concerns	low risk	low risk	low risk	high risk	high risk
Fragoso-Saavedra [120]	low risk	low risk	low risk	low risk	some concerns	some concerns
Heath [121]	low risk	low risk	low risk	low risk	low risk	low risk
Panda [122]	low risk	high risk	some concerns	some concerns	low risk	high risk
Madhi [123]	some concerns	low risk	some concerns	low risk	low risk	some concerns
Shenoy [124]	some concerns	low risk	some concerns	low risk	some concerns	high risk
Shoham [125]	low risk	low risk	low risk	low risk	low risk	low risk
Mendez-Flores [126]	low risk	low risk	low risk	low risk	low risk	low risk
Korper [127]	low risk	high risk	low risk	low risk	low risk	high risk
Betka [128]	low risk	low risk	some concerns	low risk	low risk	some concerns
Thomas [129]	low risk	low risk	low risk	low risk	low risk	low risk

© 2023 Bai AD et al. *JAMA Network Open*.

Sivapalasingam [130]	some concerns	low risk	low risk	low risk	high risk	high risk
Patankar [131]	low risk	low risk	high risk	low risk	low risk	high risk
Naggie [132]	low risk	low risk	low risk	high risk	low risk	high risk
Bukhari [133]	some concerns	high risk	some concerns	low risk	high risk	high risk
Hsieh [134]	low risk	low risk	low risk	low risk	low risk	low risk
Munch [135]	low risk	low risk	low risk	low risk	low risk	low risk
Dorward [136]	low risk	low risk	low risk	some concerns	some concerns	some concerns
Yu [137]	low risk	low risk	low risk	some concerns	low risk	some concerns
REMAP-CAP investigators [138]	low risk	low risk	low risk	low risk	low risk	low risk
REMAP-CAP investigators [139]	low risk	low risk	low risk	low risk	low risk	low risk
REMAP-CAP investigator [140]	low risk	low risk	low risk	low risk	low risk	low risk
Weinreich [141]	low risk	low risk	low risk	low risk	some concerns	some concerns
Nguyen [142]	low risk	low risk	low risk	low risk	low risk	low risk
Hinks [143]	low risk	low risk	low risk	low risk	some concerns	some concerns
Reis [144]	low risk	low risk	low risk	low risk	low risk	low risk
Quinn [145]	low risk	high risk	low risk	low risk	some concerns	high risk
Marconi [146]	low risk	low risk	low risk	low risk	low risk	low risk
Shinde [147]	low risk	low risk	low risk	low risk	low risk	low risk
Trieu [148]	low risk	some concerns	some concerns	some concerns	some concerns	some concerns
Fischer [149]	some concerns	low risk	low risk	low risk	low risk	some concerns
Feng [150]	low risk	low risk	low risk	low risk	low risk	low risk
Temesgen [151]	low risk	low risk	low risk	low risk	low risk	low risk
Pan [152]	low risk	some concerns	low risk	low risk	low risk	some concerns

Reference numbers refer to the same references for Table 1.

[a]The revised Cochrane risk-of-bias tool for cluster-randomized trials (RoB 2 CRT) was used to assess risk of bias

© 2023 Bai AD et al. *JAMA Network Open*.

eTable 3. Preprints and Corresponding Published Journal Articles

Preprint	Journal article
Abdulamir AS, Gorial FI, Saadi SJ, Maulood MF, Hashim HA, Alnuaimi AS, Abdulrrazaq MK. Effectiveness and Safety of Niclosamaide as Add-on Therapy to the Standard of Care Measures in COVID-19 Management: Randomized controlled clinical trial. medRxiv 2021.06.10.21258709; doi: https://doi.org/10.1101/2021.06.10.21258709	Abdulamir AS, Gorial FI, Saadi SJ, Maulood MF, Hashim HA, Alnuaimi AS, Abdulrrazaq MK. A randomised controlled trial of effectiveness and safety of Niclosamide as add on therapy to the standard of care measures in COVID-19 management. Ann Med Surg (Lond). 2021;69:102779. doi: 10.1016/j.amsu.2021.102779.
Lima AA, Arruda EA, Pires-Neto RJ, Medeiros MS, Quirino-Filho J, Clementino MA, Gondim RN, Magalhaes LM, Cavalcante KF, Viana VA, Perdigao L. Clinical trial of efficacy and toxicity of disoproxil tenofovir fumarate and emtricitabine for mild to moderate SARS-CoV-2 infections. medRxiv 2021.09.28.21264242; doi: https://doi.org/10.1101/2021.09.28.21264242	Arruda EA, Pires-Neto RJ, Medeiros MS, Quirino-Filho J, Clementino M, Gondim RN, Magalhães LM, Cavalcante KF, Viana VA, Mello LP, Lima DG. Clinical Features, Pathobiology, Efficacy, and Toxicity of Tenofovir Disoproxil Fumarate and Emtricitabine for Mild to Moderate SARS-CoV-2 Infections. Eur J Respir Med. 2021;3(3):238-48.
Hosseinzadeh A, Emamian MH, Tavakkolian A, Kia V, Ebrahimi H, Sheibani H, Binesh E, Jafari R, Mirrezaie SM, Jafarisani M. Application of nasal spray containing dimethyl sulfoxide (DSMO) and ethanol during the COVID-19 pandemic may protect healthcare workers: A randomized controlled trials. medRxiv 2021.07.06.21259749; doi: https://doi.org/10.1101/2021.07.06.21259749.	
Shiri AH, Raiatdoost E, Afkhami H, Ravanshad R, Hosseini SE, Kalani N, Raoufi R. The herbal combination of Sugarcane, Black Myrobalan, and mastic as a supplementary treatment for COVID-19: a randomized clinical trial. medRxiv 2021.04.27.21256221; doi: https://doi.org/10.1101/2021.04.27.21256221	
Baxter AL, Schwartz KR, Johnson R, Rao A, Gibson RW, Cherian E, Kuchinski AM, Lyon M, Schwartz RB. Rapid initiation of nasal saline irrigation: hospitalizations in COVID-19 patients randomized to alkalinization or povidone-iodine compared to a national dataset. medRxiv 2021.08.16.21262044; doi: https://doi.org/10.1101/2021.08.16.21262044	Baxter AL, Schwartz KR, Johnson RW, Kuchinski AM, Swartout KM, Srinivasa Rao ASR, Gibson RW, Cherian E, Giller T, Boomer H, Lyon M, Schwartz R. Rapid initiation of nasal saline irrigation to reduce severity in high-risk COVID+ outpatients. Ear Nose Throat J. 2022 Aug 25:1455613221123737. doi: 10.1177/01455613221123737.
Bhatt AN, Shenoy S, Munjal S, Chinnadurai V, Agarwal A, Kumar AV, Shanavas A, Kanvar R, Chandna S. 2-Deoxy-D-Glucose as an Adjunct to Standard of Care in the Medical Management of COVID-19: A Proof-of-Concept & Dose-Ranging Randomised Clinical Trial. medRxiv 2021.10.08.21258621; doi: https://doi.org/10.1101/2021.10.08.21258621	Bhatt AN, Shenoy S, Munjal S, Chinnadurai V, Agarwal A, Vinoth Kumar A, Shanavas A, Kanwar R, Chandna S. 2-deoxy-D-glucose as an adjunct to standard of care in the medical management of COVID-19: a proof-of-concept and dose-ranging randomised phase II clinical trial. BMC Infect Dis. 2022 Aug 4;22(1):669. doi: 10.1186/s12879-022-07642-6.
Gupta A, Gonzalez-Rojas Y, Juarez E, Casal MC, Moya J, Falci DR, Sarkis E, Solis J, Zheng H, Scott N, Cathcart AL, Hebner CM, Sager J, Mogalian E, Tipple C, Peppercorn A, Alexander E, Pang PS, Free A, Brinson C, Aldinger M, Shapiro AE, for the COMET-ICE Investigators. Early Covid-19 Treatment With SARS-CoV-2 Neutralizing Antibody Sotrovimab medRxiv 2021.05.27.21257096; doi: https://doi.org/10.1101/2021.05.27.21257096	Gupta A, Gonzalez-Rojas Y, Juarez E, Crespo Casal M, Moya J, Falci DR, Sarkis E, Solis J, Zheng H, Scott N, Cathcart AL, Hebner CM, Sager J, Mogalian E, Tipple C, Peppercorn A, Alexander E, Pang PS, Free A, Brinson C, Aldinger M, Shapiro AE; COMET-ICE Investigators. Early Treatment for Covid-19 with SARS-CoV-2 Neutralizing Antibody Sotrovimab. N Engl J Med. 2021;385(21):1941-1950.
Gupta A, Gonzalez-Rojas Y, Juarez E, Casal MC, Moya J, Falci DR, Sarkis E, Solis J, Zheng H, Scott N, Cathcart AL. Effect of the Neutralizing SARS-CoV-2 Antibody Sotrovimab in Preventing Progression of COVID-19: A Randomized Clinical Trial. medRxiv 2021.11.03.21265533; doi: https://doi.org/10.1101/2021.11.03.21265533	Gupta A, Gonzalez-Rojas Y, Juarez E, Crespo Casal M, Moya J, Rodrigues Falci D, Sarkis E, Solis J, Zheng H, Scott N, Cathcart AL, Parra S, Sager JE, Austin D, Peppercorn A, Alexander E, Yeh WW, Brinson C, Aldinger M, Shapiro AE; COMET-ICE Investigators. Effect of Sotrovimab on Hospitalization or Death Among High-risk Patients With Mild to Moderate COVID-19: A Randomized Clinical Trial. JAMA. 2022;327(13):1236-1246.
Gupta A, Madan A, Yadav B, Singhal R, Mundada PS, Pandey YK, Agarwal R, Rana R, Tripathi A, Sharma BS, Rao BC. Chyawanprash for the prevention of COVID-19 infection among healthcare workers: A Randomized Controlled Trial. medRxiv 2021.02.17.21251899; doi: https://doi.org/10.1101/2021.02.17.21251899	Gupta A, Madan A, Yadav B, Mundada P, Singhal R, Tripathi A, Rao BC, Gupta B, Rana R, Sharma B, Pandey Y. A randomized controlled trial to evaluate the prophylactic efficacy of Chyawanprash in healthcare workers during the COVID-19 pandemic. Journal of Research in Ayurvedic Sciences. 2021; 5(1): 13-25.
Chopra A, Tillu G, Chaudhary K, Reddy G, Srivastava A, Lakdawala M, Gode D, Reddy H, Tamboli S, Saluja M, Sarmukkaddam S. Coadministration of AYUSH 64 as an adjunct to Standard of Care in mild and moderate COVID-19: A randomised, controlled, multicentric clinical trial. medRxiv 2021.06.12.21258345; doi: https://doi.org/10.1101/2021.06.12.21258345	
Biber A, Mandelboim M, Harmelin G, Lev D, Ram L, Shaham A, Nemet I, Kliker L, Erster O, Schwartz E. Favorable outcome on viral load and culture viability using Ivermectin in early treatment of non-hospitalized patients with mild COVID-19–A double-blind, randomized	Biber A, Harmelin G, Lev D, Ram L, Shaham A, Nemet I, Kliker L, Erster O, Mandelboim M, Schwartz E. The effect of ivermectin on the viral load and culture viability in early treatment of nonhospitalized patients with mild COVID-19 - a double-blind, randomized placebo-controlled trial. Int J Infect Dis. 2022 Sep;122:733-740. doi: 10.1016/j.ijid.2022.07.003.

© 2023 Bai AD et al. *JAMA Network Open*.

placebo-controlled trial.<br>medRxiv 2021.05.31.21258081; doi: https://doi.org/10.1101/2021.05.31.21258081	
Fisher BA, Veenith T, Slade D, Gaskell C, Rowland M, Whitehouse T, Scriven J, Parekh D, Balasubramaniam M, Cooke G, Morley N. Namilumab or infliximab compared to standard of care in hospitalised patients with COVID-19 (CATALYST): a phase 2 randomised adaptive trial. medRxiv 2021.06.02.21258204; doi: https://doi.org/10.1101/2021.06.02.21258204	Fisher BA, Veenith T, Slade D, Gaskell C, Rowland M, Whitehouse T, Scriven J, Parekh D, Balasubramaniam MS, Cooke G, Morley N, Gabriel Z, Wise MP, Porter J, McShane H, Ho LP, Newsome PN, Rowe A, Sharpe R, Thickett DR, Bion J, Gates S, Richards D, Kearns P; CATALYST investigators. Namilumab or infliximab compared with standard of care in hospitalised patients with COVID-19 (CATALYST): a randomised, multicentre, multi-arm, multistage, open-label, adaptive, phase 2, proof-of-concept trial. Lancet Respir Med. 2022;10(3):255-266.
Gaborit B, Dailly E, Vanhove B, Josien R, Lacombe K, Dubee V, Ferre V, Brouard S, Ader F, Vibet MA, Le Thuaut A. Pharmacokinetics and safety of XAV-19, a swine glyco-humanized polyclonal anti-SARS-CoV-2 antibody, for COVID-19-related moderate pneumonia: a randomized, double-blind, placebo-controlled, phase IIa study.<br>medRxiv 2021.04.15.21255549; doi: https://doi.org/10.1101/2021.04.15.21255549	Gaborit B, Dailly E, Vanhove B, Josien R, Lacombe K, Dubee V, Ferre V, Brouard S, Ader F, Vibet MA, Le Thuaut A, Danger R, Flet L, Omnes A, Berly L, Chiffoleau A, Jobert A, Duvaux O, Raffi F; POLYCOR Trial Group. Pharmacokinetics and Safety of XAV-19, a Swine Glyco-humanized Polyclonal Anti-SARS-CoV-2 Antibody, for COVID-19-Related Moderate Pneumonia: a Randomized, Double-Blind, Placebo-Controlled, Phase IIa Study. Antimicrob Agents Chemother. 2021;65(9):e0123721. doi: 10.1128/AAC.01237-21.
Young BC, Eyre DW, Kendrick S, White C, Smith S, Beveridge G, Nonnemacher T, Ichofu F, Hillier J, Diamond I, Rourke E. A cluster randomised trial of the impact of a policy of daily testing for contacts of COVID-19 cases on attendance and COVID-19 transmission in English secondary schools and colleges.<br>medRxiv 2021.07.23.21260992; doi: https://doi.org/10.1101/2021.07.23.21260992	Young BC, Eyre DW, Kendrick S, White C, Smith S, Beveridge G, Nonnenmacher T, Ichofu F, Hillier J, Oakley S, Diamond I, Rourke E, Dawe F, Day I, Davies L, Staite P, Lacey A, McCrae J, Jones F, Kelly J, Bankiewicz U, Tunkel S, Ovens R, Chapman D, Bhalla V, Marks P, Hicks N, Fowler T, Hopkins S, Yardley L, Peto TEA. Daily testing for contacts of individuals with SARS-CoV-2 infection and attendance and SARS-CoV-2 transmission in English secondary schools and colleges: an open-label, cluster-randomised trial. Lancet. 2021;398(10307):1217-1229. doi: 10.1016/S0140-6736(21)01908-5.
Clemency BM, Varughese R, Gonzalez-Rojas Y, Morse CG, Phipatanakul W, Koster DJ, Blaiss MS. A randomized controlled trial of inhaled ciclesonide for outpatient treatment of symptomatic COVID-19 infections.<br>medRxiv 2021.09.07.21261811; doi: https://doi.org/10.1101/2021.09.07.21261811	Clemency BM, Varughese R, Gonzalez-Rojas Y, Morse CG, Phipatanakul W, Koster DJ, Blaiss MS. Efficacy of Inhaled Ciclesonide for Outpatient Treatment of Adolescents and Adults With Symptomatic COVID-19: A Randomized Clinical Trial. JAMA Intern Med. 2022;182(1):42-49. doi: 10.1001/jamainternmed.2021.6759.
Araujo CS, Medeiros-Ribeiro AC, Saad CG, Bonfiglioli KR, Domiciano DS, Shimabuco AY, Silva MR, Yuki EF, Pasoto SG, Pedrosa T, Kupa LD. A randomized clinical trial of 2-week methotrexate discontinuation in rheumatoid arthritis patients vaccinated with inactivated SARS-CoV-2 vaccine.<br>medRxiv 2021.11.23.21266785; doi: https://doi.org/10.1101/2021.11.23.21266785	Araujo CSR, Medeiros-Ribeiro AC, Saad CGS, Bonfiglioli KR, Domiciano DS, Shimabuco AY, Silva MSR, Yuki EFN, Pasoto SG, Pedrosa T, Kupa LVK, Zou G, Pereira RMR, Silva CA, Aikawa NE, Bonfa E. Two-week methotrexate discontinuation in patients with rheumatoid arthritis vaccinated with inactivated SARS-CoV-2 vaccine: a randomised clinical trial. Ann Rheum Dis. 2022;81(6):889-897. doi: 10.1136/annrheumdis-2021-221916.
Tornero C, Pastor E, del Mar Garzando M, Orduña J, Forner MJ, Bocigas I, Cedeño DL, Vallejo R, Staats P, Liebler EJ. Non-invasive Vagus Nerve Stimulation for Respiratory Symptoms of COVID-19: Results From a Randomized Controlled Trial (SAVIOR I).<br>medRxiv 2021.09.24.21264045; doi: https://doi.org/10.1101/2021.09.24.21264045	Tornero C, Pastor E, Garzando MDM, Orduña J, Forner MJ, Bocigas I, Cedeño DL, Vallejo R, McClure CK, Czura CJ, Liebler EJ, Staats P. Non-invasive Vagus Nerve Stimulation for COVID-19: Results From a Randomized Controlled Trial (SAVIOR I). Front Neurol. 2022;13:820864. doi: 10.3389/fneur.2022.820864.
Hernandez-Cardenas C, Thirion-Romero I, Rivera-Martinez NE, Meza-Meneses P, Remigio-Luna A, Perez-Padilla R. Hyroxychloroquine for the treatment of severe respiratory infection by COVID-19: A randomized controlled trial.<br>medRxiv 2021.02.01.21250371; doi: https://doi.org/10.1101/2021.02.01.21250371	Hernandez-Cardenas C, Thirion-Romero I, Rodríguez-Llamazares S, Rivera-Martinez NE, Meza-Meneses P, Remigio-Luna A, Perez-Padilla R; Research Group on hydroxychloroquine for COVID-19. Hydroxychloroquine for the treatment of severe respiratory infection by COVID-19: A randomized controlled trial. PLoS One. 2021;16(9):e0257238. doi: 10.1371/journal.pone.0257238.
Mok CK, Cheng SM, Chen C, Yiu K, Chan TO, Lai KC, Ling KC, Ho LL, Peiris M, Hui DS. A RCT of a third dose CoronaVac or BNT162b2 vaccine in adults with two doses of CoronaVac.<br>medRxiv 2021.11.02.21265843; doi: https://doi.org/10.1101/2021.11.02.21265843	Mok CKP, Chen C, Yiu K, Chan TO, Lai KC, Ling KC, Sun Y, Hui DS, Cheng SMS, Peiris M. A Randomized Clinical Trial Using CoronaVac or BNT162b2 Vaccine as a Third Dose in Adults Vaccinated with Two Doses of CoronaVac. Am J Respir Crit Care Med. 2022;205(7):844-847. doi: 10.1164/rccm.202111-2655LE.
Portal-Celhay C, Forleo-Neto E, Eagan W, Musser BJ, Davis JD, Turner KC, Norton T, Hooper AT, Hamilton JD, Pan C, Mahmood A. Phase 2 dose-ranging study of the virologic efficacy and safety of the combination COVID-19 antibodies casirivimab and imdevimab in the outpatient setting. medRxiv 2021.11.09.21265912; doi: https://doi.org/10.1101/2021.11.09.21265912	Portal-Celhay C, Forleo-Neto E, Eagan W, Musser BJ, Davis JD, Turner KC, Norton T, Hooper AT, Hamilton JD, Pan C, Mahmood A, Baum A, Kyratsous CA, Kim Y, Parrino J, Kampman W, Roque-Guerrero L, Stoici R, Fatakia A, Soo Y, Geba GP, Kowal B, DiCioccio AT, Stahl N, Lipsich L, Braunstein N, Herman GA, Yancopoulos GD, Weinreich DM; COVID-19 Phase 2 Dose-Ranging Study Team. Virologic Efficacy of Casirivimab and Imdevimab COVID-19 Antibody Combination in Outpatients With SARS-CoV-2 Infection: A Phase 2 Dose-Ranging

	Randomized Clinical Trial. JAMA Netw Open. 2022 Aug 1;5(8):e2225411. doi: 10.1001/jamanetworkopen.2022.25411.
Singh D, Bogus M, Moskalenko V, Lord R, Moran EJ, Crater GD, Bourdet DL, Pfeifer ND, Woo J, Kaufman E, Lombardi DA. A phase 2 study of the inhaled pan-JAK inhibitor TD-0903 in severe COVID-19: Part 1. medRxiv 2021.03.09.21252944; doi: https://doi.org/10.1101/2021.03.09.21252944	Singh D, Bogus M, Moskalenko V, Lord R, Moran EJ, Crater GD, Bourdet DL, Pfeifer ND, Woo J, Kaufman E, Lombardi DA, Weng EY, Nguyen T, Woodcock A, Haumann B, Saggar R. A phase 2 multiple ascending dose study of the inhaled pan-JAK inhibitor nezulcitinib (TD-0903) in severe COVID-19. Eur Respir J. 2021;58(4):2100673.
Sullivan DJ, Gebo KA, Shoham S, Bloch EM, Lau B, Shenoy AG, Mosnaim GS, Gniadek TJ, Fukuta Y, Patel B, Heath SL. Randomized Controlled Trial of Early Outpatient COVID-19 Treatment with High-Titer Convalescent Plasma. medRxiv 2021.12.10.21267485; doi: https://doi.org/10.1101/2021.12.10.21267485	Sullivan DJ, Gebo KA, Shoham S, Bloch EM, Lau B, Shenoy AG, Mosnaim GS, Gniadek TJ, Fukuta Y, Patel B, Heath SL, Levine AC, Meisenberg BR, Spivak ES, Anjan S, Huaman MA, Blair JE, Currier JS, Paxton JH, Gerber JM, Petrini JR, Broderick PB, Rausch W, Cordisco ME, Hammel J, Greenblatt B, Cluzet VC, Cruser D, Oei K, Abinante M, Hammitt LL, Sutcliffe CG, Forthal DN, Zand MS, Cachay ER, Raval JS, Kassaye SG, Foster EC, Roth M, Marshall CE, Yarava A, Lane K, McBee NA, Gawad AL, Karlen N, Singh A, Ford DE, Jabs DA, Appel LJ, Shade DM, Ehrhardt S, Baksh SN, Laeyendecker O, Pekosz A, Klein SL, Casadevall A, Tobian AAR, Hanley DF. Early Outpatient Treatment for Covid-19 with Convalescent Plasma. N Engl J Med. 2022;386(18):1700-1711. doi: 10.1056/NEJMoa2119657.
Huang DT, McCreary EK, Bariola JR, Minnier TE, Wadas RJ, Shovel JA, Albin D, Marroquin OC, Kip KE, Collins K, Schmidhofer M. Effectiveness of casirivimab and imdevimab, and sotrovimab during Delta variant surge: a prospective cohort study and comparative effectiveness randomized trial. medRxiv 2021.12.23.21268244; doi: https://doi.org/10.1101/2021.12.23.21268244	Huang DT, McCreary EK, Bariola JR, Minnier TE, Wadas RJ, Shovel JA, Albin D, Marroquin OC, Kip KE, Collins K, Schmidhofer M, Wisniewski MK, Nace DA, Sullivan C, Axe M, Meyers R, Weissman A, Garrard W, Peck-Palmer OM, Wells A, Bart RD, Yang A, Berry LR, Berry S, Crawford AM, McGlothlin A, Khadem T, Linstrum K, Montgomery SK, Ricketts D, Kennedy JN, Pidro CJ, Nakayama A, Zapf RL, Kip PL, Haidar G, Snyder GM, McVerry BJ, Yealy DM, Angus DC, Seymour CW. Effectiveness of Casirivimab-Imdevimab and Sotrovimab During a SARS-CoV-2 Delta Variant Surge: A Cohort Study and Randomized Comparative Effectiveness Trial. JAMA Netw Open. 2022 Jul 1;5(7):e2220957. doi: 10.1001/jamanetworkopen.2022.20957.
Weinreich DM, Sivapalasingam S, Norton T, Ali S, Gao H, Bhore R, Xiao J, Hooper AT, Hamilton JD, Musser BJ, Rofail D. REGEN-COV antibody cocktail clinical outcomes study in Covid-19 outpatients. medRxiv 2021.05.19.21257469; doi: https://doi.org/10.1101/2021.05.19.21257469	Weinreich DM, Sivapalasingam S, Norton T, Ali S, Gao H, Bhore R, Xiao J, Hooper AT, Hamilton JD, Musser BJ, Rofail D, Hussein M, Im J, Atmodjo DY, Perry C, Pan C, Mahmood A, Hosain R, Davis JD, Turner KC, Baum A, Kyratsous CA, Kim Y, Cook A, Kampman W, Roque-Guerrero L, Acloque G, Aazami H, Cannon K, Simón-Campos JA, Bocchini JA, Kowal B, DiCioccio AT, Soo Y, Geba GP, Stahl N, Lipsich L, Braunstein N, Herman G, Yancopoulos GD; Trial Investigators. REGEN-COV Antibody Combination and Outcomes in Outpatients with Covid-19. N Engl J Med. 2021;385(23):e81.
Parikh D, Chaturvedi A, Shah N, Patel P, Patel R, Ray S. Safety and efficacy of COVID-19 hyperimmune globulin (HIG) solution in the treatment of active COVID-19 infection-Findings from a Prospective, Randomized, Controlled, Multi-Centric Trial. medRxiv 2021.07.26.21261119; doi: https://doi.org/10.1101/2021.07.26.21261119	Parikh D, Chaturvedi A, Shah N, Patel P, Patel R, Ray S. Safety and Efficacy of COVID-19 Hyperimmune Globulin (HIG) Solution in the Treatment of Active COVID-19 infection-Findings from a Prospective, Randomized, Controlled, Multi-Centric Trial. The Indian Practitioner. 2021;74(11):15.
Ely EW, Ramanan AV, Kartman CE, de Bono S, Liao R, Piruzeli ML, Goldman JD, Saraiva JF, Chakladar S, Marconi VC, Alatorre-Alexander J. Baricitinib plus Standard of Care for Hospitalised Adults with COVID-19 on Invasive Mechanical Ventilation or Extracorporeal Membrane Oxygenation: Results of a Randomised, Placebo-Controlled Trial. medRxiv 2021.10.11.21263897; doi: https://doi.org/10.1101/2021.10.11.21263897	Ely EW, Ramanan AV, Kartman CE, de Bono S, Liao R, Piruzeli MLB, Goldman JD, Saraiva JFK, Chakladar S, Marconi VC; COV-BARRIER Study Group. Efficacy and safety of baricitinib plus standard of care for the treatment of critically ill hospitalised adults with COVID-19 on invasive mechanical ventilation or extracorporeal membrane oxygenation: an exploratory, randomised, placebo-controlled trial. Lancet Respir Med. 2022;10(4):327-336. doi: 10.1016/S2213-2600(22)00006-6.
Fedrizzi EN, Girondi JB, Sakae TM, Steffens SM, de Souza Silvestrin AN, Claro GS, Iskenderian HA, Hillmann B, Gervasi L, Trapani A, de Amorim Rodrigues P. Efficacy of the measles-mumps-rubella (MMR) vaccine in the reducing the severity of covid-19: An interim analysis of a randomised controlled clinical trial. medRxiv 2021.09.14.21263598; doi: https://doi.org/10.1101/2021.09.14.21263598	Fedrizzi EN, Girondi JB, Sakae TM, Steffens SM, de Souza Silvestrin AN, Claro GS, Iskenderian HA, Hillmann B, Gervasi L, Trapani A, de Amorim Rodrigues P. (2022) Efficacy of the Measles-Mumps-Rubella (MMR) Vaccine in the Reducing the Severity of Covid-19: An Interim Analysis of a Randomised Controlled Clinical Trial. J Clin Trials. S14:009.
Balint EM, Gruener B, Haase S, Kaw-Geppert M, Thayer JF, Guendel HO, Jarczok MN. A randomized clinical trial to stimulate the cholinergic anti-inflammatory pathway in patients with moderate COVID-19-pneumonia using a slow-paced breathing technique. medRxiv 2021.12.03.21266946; doi: https://doi.org/10.1101/2021.12.03.21266946	

© 2023 Bai AD et al. *JAMA Network Open*.

Kolev E, Mircheva L, Edwards M, Johnston SL, Kalinov K, Stange R, Gancitano G, Berghe WV, Kreft S. Echinacea purpurea for the Long-term Prevention of Viral Respiratory Tract Infections during COVID-19 Pandemic: A Randomized, Open, Controlled, Exploratory Clinical Study. medRxiv 2021.12.10.21267582; doi: https://doi.org/10.1101/2021.12.10.21267582	Kolev E, Mircheva L, Edwards MR, Johnston SL, Kalinov K, Stange R, Gancitano G, Berghe WV, Kreft S. *Echinacea Purpurea* For the Long-Term Prevention of Viral Respiratory Tract Infections During Covid-19 Pandemic: A Randomized, Open, Controlled, Exploratory Clinical Study. Front Pharmacol. 2022;13:856410. doi: 10.3389/fphar.2022.856410.
Breza E, Stanford FC, Alsan M, Alsan B, Banerjee A, Chandrasekhar AG, Eichmeyer S, Glushko T, Goldsmith-Pinkham P, Holland K, Hoppe E. Doctors' and Nurses' Social Media Ads Reduced Holiday Travel and COVID-19 Infections: A Cluster Randomized Controlled Trial. National Bureau of Economic Research. medRxiv 2021.06.23.21259402; doi: https://doi.org/10.1101/2021.06.23.21259402	Breza E, Stanford FC, Alsan M, Alsan B, Banerjee A, Chandrasekhar AG, Eichmeyer S, Glushko T, Goldsmith-Pinkham P, Holland K, Hoppe E, Karnani M, Liegl S, Loisel T, Ogbu-Nwobodo L, Olken BA, Torres C, Vautrey PL, Warner ET, Wootton S, Duflo E. Effects of a large-scale social media advertising campaign on holiday travel and COVID-19 infections: a cluster randomized controlled trial. Nat Med. 2021;27(9):1622-1628. doi: 10.1038/s41591-021-01487-3.
Lattman E, Bhalerao P, ShashiBhushan BL, Nargundkar N, Lattmann P, Balaram PN. Randomized, Comparative, Clinical Trial to Evaluate Efficacy and Safety of PNB001 in Moderate COVID-19 Patients. medRxiv 2021.04.16.21255256; doi: https://doi.org/10.1101/2021.04.16.21255256.	Lattman E, Bhalerao P, ShashiBhushan BL, Nargundkar N, Lattmann P, Balaram PN. Randomized, Comparative, Clinical Trial to Evaluate Efficacy and Safety of PNB001 in Moderate COVID-19 Patients. Med J Clin Trials Case Stud 2021, 5(4): 000297. doi: 10.23880/mjccs-16000297.
Gaughan E, Sethi T, Quinn T, Hirani N, Mills A, Bruce AM, MacKinnon A, Aslanis V, Li F, O'Connor R, Parker RA. GB0139, an inhaled small molecule inhibitor of galectin-3, in COVID-19 pneumonitis: a randomised, controlled, open-label, phase 2a experimental medicine trial of the safety, pharmacokinetics, and potential therapeutic value. medRxiv 2021.12.21.21267983; doi: https://doi.org/10.1101/2021.12.21.21267983	Gaughan EE, Quinn TM, Mills A, Bruce AM, Antonelli J, MacKinnon A, Aslanis V, Li F, O'Connor R, Boz C, Mills R, Emanuel P, Burgess M, Rinaldi G, Valanciute A, Mills B, Scholefield E, Hardisty G, Gwyer Findlay E, Parker RA, Norrie J, Dear JW, Akram AR, Koch O, Templeton K, Dockrell DH, Walsh TS, Partridge S, Humphries D, Wang-Jairaj J, Slack RJ, Schambye H, Phung D, Gravelle L, Lindmark B, Shankar-Hari M, Hirani N, Sethi T, Dhaliwal K. An Inhaled Galectin-3 Inhibitor in COVID-19 Pneumonitis (DEFINE): A Phase Ib/IIa Randomised Controlled Trial. Am J Respir Crit Care Med. 2022 Aug 16. doi: 10.1164/rccm.202203-0477OC.
McCreary EK, Bariola JR, Minnier T, Wadas RJ, Shovel JA, Albin DL, Marroquin OC, Kip KE, Collins K, Schmidhofer M, Wisniewski MK. A learning health system randomized trial of monoclonal antibodies for COVID-19. medRxiv 2021.09.03.21262551; doi: https://doi.org/10.1101/2021.09.03.21262551	McCreary EK, Bariola JR, Minnier TE, Wadas RJ, Shovel JA, Albin D, Marroquin OC, Kip KE, Collins K, Schmidhofer M, Wisniewski MK, Nace DA, Sullivan C, Axe M, Meyers R, Weissman A, Garrard W, Peck-Palmer OM, Wells A, Bart RD, Yang A, Berry LR, Berry S, Crawford AM, McGlothlin A, Khadem T, Linstrum K, Montgomery SK, Ricketts D, Kennedy JN, Pidro CJ, Haidar G, Snyder GM, McVerry BJ, Yealy DM, Angus DC, Nakayama A, Zapf RL, Kip PL, Seymour CW, Huang DT. The comparative effectiveness of COVID-19 monoclonal antibodies: A learning health system randomized clinical trial. Contemp Clin Trials. 2022 Aug;119:106822. doi: 10.1016/j.cct.2022.106822.
Sobngwi E, Zemsi S, Guewo-Fokeng M, Katte JC, Kouanfack C, Mfeukeu-Kuate L, Zemsi A, Wasnyo Y, Assiga-Ntsama A, Ndi-Manga JA, Tambekou JS. Doxycycline is a safe alternative to Hydroxychloroquine+ Azithromycin to prevent clinical worsening and hospitalization in mild COVID-19 patients: An open label randomized clinical trial (DOXYCOV). medRxiv 2021.07.25.21260838; doi: https://doi.org/10.1101/2021.07.25.21260838	
Kyriazopoulou E, Poulakou G, Milionis H, Metallidis S, Adamis G, Tsiakos K, Fragkou A, Rapti A, Damoulari C, Fantoni M, Kalomenidis I. Early Anakinra Treatment for COVID-19 Guided by Urokinase Plasminogen Receptor. medRxiv 2021.05.16.21257283; doi: https://doi.org/10.1101/2021.05.16.21257283	Kyriazopoulou E, Poulakou G, Milionis H, Metallidis S, Adamis G, Tsiakos K, Fragkou A, Rapti A, Damoulari C, Fantoni M, Kalomenidis I, Chrysos G, Angheben A, Kainis I, Alexiou Z, Castelli F, Serino FS, Tsilika M, Bakakos P, Nicastri E, Tzavara V, Kostis E, Dagna L, Koufargyris P, Dimakou K, Savvanis S, Tzatzagou G, Chini M, Cavalli G, Bassetti M, Katrini K, Kotsis V, Tsoukalas G, Selmi C, Bliziotis I, Samarkos M, Doumas M, Ktena S, Masgala A, Papanikolaou I, Kosmidou M, Myrodia DM, Argyraki A, Cardellino CS, Koliakou K, Katsigianni EI, Rapti V, Giannitsioti E, Cingolani A, Micha S, Akinosoglou K, Liatsis-Douvitsas O, Symbardi S, Gatselis N, Mouktaroudi M, Ippolito G, Florou E, Kotsaki A, Netea MG, Eugen-Olsen J, Kyprianou M, Panagopoulos P, Dalekos GN, Giamarellos-Bourboulis EJ. Early treatment of COVID-19 with anakinra guided by soluble urokinase plasminogen receptor plasma levels: a double-blind, randomized controlled phase 3 trial. Nat Med. 2021;27(10):1752-1760. doi: 10.1038/s41591-021-01499-z.
Mikhaylov EN, Lyubimtseva TA, Vakhrushev AD, Stepanov D, Lebedev DS, Vasilieva EY, Konradi AO, Shlyakhto EV. Bromhexine hydrochloride prophylaxis of COVID-19 for medical	Mikhaylov EN, Lyubimtseva TA, Vakhrushev AD, Stepanov D, Lebedev DS, Vasilieva EY, Konradi AO, Shlyakhto EV. Bromhexine Hydrochloride Prophylaxis of COVID-19 for Medical

© 2023 Bai AD et al. *JAMA Network Open*.

personnel: a randomized open-label study. medRxiv 2021.03.03.21252855; doi: https://doi.org/10.1101/2021.03.03.21252855	Personnel: A Randomized Open-Label Study. Interdiscip Perspect Infect Dis. 2022 Jan 29;2022:4693121. doi: 10.1155/2022/4693121.
Goligher EC, Bradbury CA, McVerry BJ, Lawler PR, Berger JS, Gong MN, Carrier M, Reynolds HR, Kumar A, Turgeon AF, Kornblith LZ. Therapeutic anticoagulation in critically ill patients with Covid-19-preliminary report. medRxiv 2021.03.10.21252749; doi: https://doi.org/10.1101/2021.03.10.21252749	REMAP-CAP Investigators; ACTIV-4a Investigators; ATTACC Investigators, Goligher EC, Bradbury CA, McVerry BJ, Lawler PR, Berger JS, Gong MN, Carrier M, Reynolds HR, Kumar A, Turgeon AF, Kornblith LZ, Kahn SR, Marshall JC, Kim KS, Houston BL, Derde LPG, Cushman M, Tritschler T, Angus DC, Godoy LC, McQuilten Z, Kirwan BA, Farkouh ME, Brooks MM, Lewis RJ, Berry LR, Lorenzi E, Gordon AC, Ahuja T, Al-Beidh F, Annane D, Arabi YM, Aryal D, Baumann Kreuziger L, Beane A, Bhimani Z, Bihari S, Billett HH, Bond L, Bonten M, Brunkhorst F, Buxton M, Buzgau A, Castellucci LA, Chekuri S, Chen JT, Cheng AC, Chkhikvadze T, Coiffard B, Contreras A, Costantini TW, de Brouwer S, Detry MA, Duggal A, Džavík V, Effron MB, Eng HF, Escobedo J, Estcourt LJ, Everett BM, Fergusson DA, Fitzgerald M, Fowler RA, Froess JD, Fu Z, Galanaud JP, Galen BT, Gandotra S, Girard TD, Goodman AL, Goossens H, Green C, Greenstein YY, Gross PL, Haniffa R, Hegde SM, Hendrickson CM, Higgins AM, Hindenburg AA, Hope AA, Horowitz JM, Horvat CM, Huang DT, Hudock K, Hunt BJ, Husain M, Hyzy RC, Jacobson JR, Jayakumar D, Keller NM, Khan A, Kim Y, Kindzelski A, King AJ, Knudson MM, Kornblith AE, Kutcher ME, Laffan MA, Lamontagne F, Le Gal G, Leeper CM, Leifer ES, Lim G, Gallego Lima F, Linstrum K, Litton E, Lopez-Sendon J, Lother SA, Marten N, Saud Marinez A, Martinez M, Mateos Garcia E, Mavromichalis S, McAuley DF, McDonald EG, McGlothlin A, McGuinness SP, Middeldorp S, Montgomery SK, Mouncey PR, Murthy S, Nair GB, Nair R, Nichol AD, Nicolau JC, Nunez-Garcia B, Park JJ, Park PK, Parke RL, Parker JC, Parnia S, Paul JD, Pompilio M, Quigley JG, Rosenson RS, Rost NS, Rowan K, Santos FO, Santos M, Santos MO, Satterwhite L, Saunders CT, Schreiber J, Schutgens REG, Seymour CW, Siegal DM, Silva DG Jr, Singhal AB, Slutsky AS, Solvason D, Stanworth SJ, Turner AM, van Bentum-Puijk W, van de Veerdonk FL, van Diepen S, Vazquez-Grande G, Wahid L, Wareham V, Widmer RJ, Wilson JG, Yuriditsky E, Zhong Y, Berry SM, McArthur CJ, Neal MD, Hochman JS, Webb SA, Zarychanski R. Therapeutic Anticoagulation with Heparin in Critically Ill Patients with Covid-19. N Engl J Med. 2021;385(9):777-789. doi: 10.1056/NEJMoa2103417.
Syed F, Arif MA, Niazi R, Baqar JB, Hashmi UL, Batool S, Ashraf S, Arshad J, Musarrat S. Pre-Exposure Prophylaxis with Various Doses of Hydroxychloroquine among high-risk COVID 19 Healthcare Personnel: CHEER randomized controlled trial. medRxiv 2021.05.17.21257012; doi: https://doi.org/10.1101/2021.05.17.21257012	Syed F, Hassan M, Arif MA, Batool S, Niazi R, Laila UE, Ashraf S, Arshad J. Pre-exposure Prophylaxis With Various Doses of Hydroxychloroquine Among Healthcare Personnel With High-Risk Exposure to COVID-19: A Randomized Controlled Trial. Cureus. 2021;13(12):e20572. doi: 10.7759/cureus.20572.
Cadegiani FA, Fonseca DN, McCoy J, Zimerman RA, Mirza FN, Correia MN, Barros RN, Onety DC, Israel KC, Almeida BG, Guerreiro EO. Efficacy of proxalutamide in hospitalized COVID-19 patients: a randomized, double-blind, placebo-controlled, parallel-design clinical trial. medRxiv 2021.06.22.21259318; doi: https://doi.org/10.1101/2021.06.22.21259318	Cadegiani FA, Zimerman RA, Fonseca DN, Correia MN, Muller MP, Bet DL, Slaviero MR, Zardo I, Benites PR, Barros RN, Paulain RW, Onety DC, Israel KCP, Gustavo Wambier C, Goren A. Final Results of a Randomized, Placebo-Controlled, Two-Arm, Parallel Clinical Trial of Proxalutamide for Hospitalized COVID-19 Patients: A Multiregional, Joint Analysis of the Proxa-Rescue AndroCoV Trial. Cureus. 2021;13(12):e20691. doi: 10.7759/cureus.20691.
Cadegiani FA, Zimerman RA, do Nascimento Fonseca D, do Nascimento Correia M, McCoy J, Wambier CG, Goren A. Proxalutamide (GT0918) reduces the rate of hospitalization in mild-to-moderate COVID-19 female patients: a randomized double-blinded placebo-controlled two-arm parallel trial. medRxiv 2021.07.06.21260086; doi: https://doi.org/10.1101/2021.07.06.21260086	
Ader F, Peiffer-Smadja N, Poissy J, Bouscambert-Duchamp M, Belhadi D, Delmas C, Saillard J, Dechanet A, Mercier N, Dupont A, Alfaiate T. Antiviral drugs in hospitalized patients with COVID-19-the DisCoVeRy trial. medRxiv 2021.01.08.20248149; doi: https://doi.org/10.1101/2021.01.08.20248149	Ader F, Peiffer-Smadja N, Poissy J, Bouscambert-Duchamp M, Belhadi D, Diallo A, Delmas C, Saillard J, Dechanet A, Mercier N, Dupont A, Alfaiate T, Lescure FX, Raffi F, Goehringer F, Kimmoun A, Jaureguiberry S, Reignier J, Nseir S, Danion F, Clere-Jehl R, Bouiller K, Navellou JC, Tolsma V, Cabié A, Dubost C, Courjon J, Leroy S, Mootien J, Gaci R, Mourvillier B, Faure E, Pourcher V, Gallien S, Launay O, Lacombe K, Lanoix JP, Makinson A, Martin-Blondel G, Bouadma L, Botelho-Nevers E, Gagneux-Brunon A, Epaulard O, Piroth L, Wallet F, Richard JC, Reuter J, Staub T, Lina B, Noret M, Andrejak C, Lê MP, Peytavin G, Hites M, Costagliola D, Yazdanpanah Y, Burdet C, Mentré F; DisCoVeRy study group. An open-label randomized controlled trial of the effect of lopinavir/ritonavir, lopinavir/ritonavir plus IFN-β-1a and

© 2023 Bai AD et al. *JAMA Network Open*.

	hydroxychloroquine in hospitalized patients with COVID-19. Clin Microbiol Infect. 2021;27(12):1826-1837. doi: 10.1016/j.cmi.2021.05.020.
Fralick M, Colacci M, Munshi L, Venus K, Fidler L, Hussein H, Britto K, Fowler R, Da Costa B, Dhalla I, Dunbar-Yaffe R. Prone positioning of patients with moderate hypoxia due to COVID-19: A multicenter pragmatic randomized trial [COVID-PRONE]. medRxiv 2021.11.05.21264590; doi: https://doi.org/10.1101/2021.11.05.21264590	Fralick M, Colacci M, Munshi L, Venus K, Fidler L, Hussein H, Britto K, Fowler R, da Costa BR, Dhalla I, Dunbar-Yaffe R, Branfield Day L, MacMillan TE, Zipursky J, Carpenter T, Tang T, Cooke A, Hensel R, Bregger M, Gordon A, Worndl E, Go S, Mandelzweig K, Castellucci LA, Tamming D, Razak F, Verma AA; COVID Prone Study Investigators. Prone positioning of patients with moderate hypoxaemia due to covid-19: multicentre pragmatic randomised trial (COVID-PRONE). BMJ. 2022 Mar 23;376:e068585. doi: 10.1136/bmj-2021-068585.
Hernandez-Bernal, F., del Carmen Ricardo-Cobas, M., Martin-Bauta, Y., Navarro-Rodriguez, Z., Piñera-Martínez, M., Quintana-Guerra, J., Urrutia-Pérez, K., Urrutia-Perez, K., Chavez-Chong, C.O., Azor-Hernandez, J.L. and Rodriguez-Reinoso, J.L., 2021. Safety, tolerability, and immunogenicity of a SARS-CoV-2 recombinant spike protein vaccine: a randomised, double-blind, placebo-controlled, phase 1-2 clinical trial (ABDALA Study). medRxiv 2021.11.30.21267047; doi: https://doi.org/10.1101/2021.11.30.21267047	Hernández-Bernal F, Ricardo-Cobas MC, Martín-Bauta Y, Navarro-Rodríguez Z, Piñera-Martínez M, Quintana-Guerra J, Urrutia-Pérez K, Urrutia-Pérez K, Chávez-Chong CO, Azor-Hernández JL, Rodríguez-Reinoso JL, Lobaina-Lambert L, Colina-Ávila E, Bizet-Almeida J, Rodríguez-Nuviola J, Del Valle-Piñera S, Ramírez-Domínguez M, Tablada-Ferreiro E, Alonso-Valdés M, Lemos-Pérez G, Guillén-Nieto GE, Palenzuela-Díaz A, Noa-Romero E, Limonta-Fernández M, Fernández-Ávila JM, Ali-Mros NA, Del Toro-Lahera L, Remedios-Reyes R, Ayala-Ávila M, Muzio-González VL. Safety, tolerability, and immunogenicity of a SARS-CoV-2 recombinant spike RBD protein vaccine: A randomised, double-blind, placebo-controlled, phase 1-2 clinical trial (ABDALA Study). EClinicalMedicine. 2022 Apr;46:101383. doi: 10.1016/j.eclinm.2022.101383.
Lescure FX, Honda H, Fowler RA, Lazar JS, Shi G, Wung P, Patel N, Hagino O. Sarilumab treatment of hospitalised patients with severe or critical COVID-19: a multinational, randomised, adaptive, phase 3, double-blind, placebo-controlled trial. medRxiv 2021.02.01.21250769; doi: https://doi.org/10.1101/2021.02.01.21250769	Lescure FX, Honda H, Fowler RA, Lazar JS, Shi G, Wung P, Patel N, Hagino O; Sarilumab COVID-19 Global Study Group. Sarilumab in patients admitted to hospital with severe or critical COVID-19: a randomised, double-blind, placebo-controlled, phase 3 trial. Lancet Respir Med. 2021 May;9(5):522-532. doi: 10.1016/S2213-2600(21)00099-0.
Perkins GD, Ji C, Connolly BA, Couper K, Lall R, Baillie JK, Bradley JM, Dark P, Dave C, De Soyza A, Dennis AV. An adaptive randomized controlled trial of non-invasive respiratory strategies in acute respiratory failure patients with COVID-19. medRxiv 2021.08.02.21261379; doi: https://doi.org/10.1101/2021.08.02.21261379	Perkins GD, Ji C, Connolly BA, Couper K, Lall R, Baillie JK, Bradley JM, Dark P, Dave C, De Soyza A, Dennis AV, Devrell A, Fairbairn S, Ghani H, Gorman EA, Green CA, Hart N, Hee SW, Kimbley Z, Madathil S, McGowan N, Messer B, Naisbitt J, Norman C, Parekh D, Parkin EM, Patel J, Regan SE, Ross C, Rostron AJ, Saim M, Simonds AK, Skilton E, Stallard N, Steiner M, Vancheeswaran R, Yeung J, McAuley DF; RECOVERY-RS Collaborators. Effect of Noninvasive Respiratory Strategies on Intubation or Mortality Among Patients With Acute Hypoxemic Respiratory Failure and COVID-19: The RECOVERY-RS Randomized Clinical Trial. JAMA. 2022;327(6):546-558. doi: 10.1001/jama.2022.0028.
Roozen GV, Prins ML, van Binnendijk RS, den Hartog G, Kuiper VP, Prins C, Janse JJ, Kruithof AC, Feltkamp MC, Kuijer M, Roosendaal FR. Tolerability, safety and immunogenicity of intradermal delivery of a fractional dose mRNA-1273 SARS-CoV-2 vaccine in healthy adults as a dose sparing strategy. medRxiv 2021.07.27.21261116; doi: https://doi.org/10.1101/2021.07.27.21261116	
Tornling G, Batta R, Porter J, Bengtsson T, Parmar K, Kashiva R, Hallberg A, Cohrt AK, Westergaard K, Dalsgaard CJ, Raud J. The angiotensin type 2 receptor agonist C21 restores respiratory function in COVID19-a double-blind, randomized, placebo-controlled Phase 2 trial. medRxiv. 2021 Jan 1. medRxiv 2021.01.26.21250511; doi: https://doi.org/10.1101/2021.01.26.21250511	Tornling G, Batta R, Porter JC, Williams B, Bengtsson T, Parmar K, Kashiva R, Hallberg A, Cohrt AK, Westergaard K, Dalsgaard CJ, Raud J. Seven days treatment with the angiotensin II type 2 receptor agonist C21 in hospitalized COVID-19 patients; a placebo-controlled randomised multi-centre double-blind phase 2 trial. EClinicalMedicine. 2021;41:101152. doi: 10.1016/j.eclinm.2021.101152.
Resende GG, da Cruz Lage R, Lobê SQ, Medeiros AF, e Silva AD, Sá AT, de Assis Oliveira AJ, Sousa D, Guimarães HC, Gomes IC, Souza RP. Blockade of Interleukin Seventeen (IL-17A) with Secukinumab in Hospitalized COVID-19 patients–the BISHOP study. medRxiv 2021.07.21.21260963; doi: https://doi.org/10.1101/2021.07.21.21260963	Resende GG, da Cruz Lage R, Lobê SQ, Medeiros AF, Costa E Silva AD, Nogueira Sá AT, Oliveira AJA, Sousa D, Guimarães HC, Gomes IC, Souza RP, Aguiar RS, Tunala R, Forestiero F, Bueno Filho JSS, Teixeira MM. Blockade of interleukin seventeen (IL-17A) with secukinumab in hospitalized COVID-19 patients - the BISHOP study. Infect Dis (Lond). 2022:1-9. doi: 10.1080/23744235.2022.2066171.
Dai H, Saccardo S, Han MA, Roh L, Raja N, Vangala S, Modi H, Pandya S, Croymans DM. Behavioral nudges increase COVID-19 vaccinations: Two randomized controlled trials. medRxiv 2021.04.12.21254876; doi: https://doi.org/10.1101/2021.04.12.21254876	Dai H, Saccardo S, Han MA, Roh L, Raja N, Vangala S, Modi H, Pandya S, Sloyan M, Croymans DM. Behavioural nudges increase COVID-19 vaccinations. Nature. 2021;597(7876):404-409. doi: 10.1038/s41586-021-03843-2.
Gaitán-Duarte HG, Álvarez-Moreno C, Rincón-Rodríguez CJ, Yomayusa-González N, Cortés JA, Villar JC, Bravo-Ojeda JS, García-Peña Á, Adarme-Jaimes W, Rodríguez-Romero VA,	Gaitán-Duarte HG, Álvarez-Moreno C, Rincón-Rodríguez CJ, Yomayusa-González N, Cortés JA, Villar JC, Bravo-Ojeda JS, García-Peña A, Adarme-Jaimes W, Rodríguez-Romero VA,

© 2023 Bai AD et al. *JAMA Network Open*.

Villate-Soto SL. Effectiveness of Rosuvastatin plus Colchicine, Emtricitabine/Tenofovir and a combination of them in Hospitalized Patients with SARS Covid-19. medRxiv 2021.07.06.21260085; doi: https://doi.org/10.1101/2021.07.06.21260085	Villate-Soto SL, Buitrago G, Chacón-Sarmiento J, Macias-Quintero M, Vaca CP, Gómez-Restrepo C, Rodríguez-Malagón N. Effectiveness of rosuvastatin plus colchicine, emtricitabine/tenofovir and combinations thereof in hospitalized patients with COVID-19: a pragmatic, open-label randomized trial. EClinicalMedicine. 2022;43:101242. doi: 10.1016/j.eclinm.2021.101242.
Arnardottir H, Pawelzik SC, Sarajlic P, Quaranta A, Kolmert J, Religa D, Wheelock CE, Back M. Immunomodulation by intravenous omega-3 fatty acid treatment in older subjects hospitalized for COVID-19: a single-blind randomized controlled trial. medRxiv 2021.12.27.21268264; doi: https://doi.org/10.1101/2021.12.27.21268264	Arnardottir H, Pawelzik SC, Sarajlic P, Quaranta A, Kolmert J, Religa D, Wheelock CE, Bäck M. Immunomodulation by intravenous omega-3 fatty acid treatment in older subjects hospitalized for COVID-19: A single-blind randomized controlled trial. Clin Transl Med. 2022 Sep;12(9):e895. doi: 10.1002/ctm2.895.
Zhang Y, Zeng G, Pan H, Li C, Kan B, Hu Y, Mao H, Xin Q, Chu K, Han W, Chen Z. Immunogenicity and safety of a SARS-CoV-2 inactivated vaccine in healthy adults aged 18-59 years: report of the randomized, double-blind, and placebo-controlled phase 2 clinical trial. medRxiv 2020.07.31.20161216; doi: https://doi.org/10.1101/2020.07.31.20161216	Pan HX, Liu JK, Huang BY, Li GF, Chang XY, Liu YF, Wang WL, Chu K, Hu JL, Li JX, Zhu DD, Wu JL, Xu XY, Zhang L, Wang M, Tan WJ, Huang WJ, Zhu FC. Immunogenicity and safety of a severe acute respiratory syndrome coronavirus 2 inactivated vaccine in healthy adults: randomized, double-blind, and placebo-controlled phase 1 and phase 2 clinical trials. Chin Med J (Engl). 2021;134(11):1289-1298. doi: 10.1097/CM9.0000000000001573.
Almanza-Reyes H, Moreno S, Plascencia-López I, Alvarado-Vera M, Patrón-Romero L, Borrego B, Reyes-Escamilla A, Valencia-Manzo D, Brun A, Pestryakov A, Bogdanchikova N. Evaluation of silver nanoparticles for the prevention of SARS-CoV-2 infection in health workers: in vitro and in vivo. medRxiv 2021.05.20.21256197; doi: https://doi.org/10.1101/2021.05.20.21256197	Almanza-Reyes H, Moreno S, Plascencia-López I, Alvarado-Vera M, Patrón-Romero L, Borrego B, Reyes-Escamilla A, Valencia-Manzo D, Brun A, Pestryakov A, Bogdanchikova N. Evaluation of silver nanoparticles for the prevention of SARS-CoV-2 infection in health workers: In vitro and in vivo. PLoS One. 2021;16(8):e0256401. doi: 10.1371/journal.pone.0256401.
Faramarzi H, Sahebkar A, Hosseinpour A, Khaloo V, Chamanpara P, Heydari MR, Najafi S, Khankahdany FF, Movahedpour A. Efficacy and safety of a novel antiviral preparation in ICU-admitted patients with COVID-19: a phase III randomized controlled trial. medRxiv 2021.11.20.21266229; doi: https://doi.org/10.1101/2021.11.20.21266229	
Patel J, Beishuizen A, Ruiz XB, Boughanmi H, Cahn A, Criner GJ, Davy K, de-Miguel-Díez J, Fernandes S, François B, Gupta A. A randomized trial of otilimab in severe COVID-19 pneumonia (OSCAR). medRxiv 2021.04.14.21255475; doi: https://doi.org/10.1101/2021.04.14.21255475	
Tardif JC, Bouabdallaoui N, L'Allier PL, Gaudet D, Shah B, Pillinger MH, Lopez-Sendon J, da Luz P, Verret L, Audet S, Dupuis J. Efficacy of colchicine in non-hospitalized patients with COVID-19. medRxiv 2021.01.26.21250494; doi: https://doi.org/10.1101/2021.01.26.21250494	Tardif JC, Bouabdallaoui N, L'Allier PL, Gaudet D, Shah B, Pillinger MH, Lopez-Sendon J, da Luz P, Verret L, Audet S, Dupuis J, Denault A, Pelletier M, Tessier PA, Samson S, Fortin D, Tardif JD, Busseuil D, Goulet E, Lacoste C, Dubois A, Joshi AY, Waters DD, Hsue P, Lepor NE, Lesage F, Sainturet N, Roy-Clavel E, Bassevitch Z, Orfanos A, Stamatescu G, Grégoire JC, Busque L, Lavallée C, Hétu PO, Paquette JS, Deftereos SG, Levesque S, Cossette M, Nozza A, Chabot-Blanchet M, Dubé MP, Guertin MC, Boivin G; COLCORONA Investigators. Colchicine for community-treated patients with COVID-19 (COLCORONA): a phase 3, randomised, double-blinded, adaptive, placebo-controlled, multicentre trial. Lancet Respir Med. 2021;9(8):924-932. doi: 10.1016/S2213-2600(21)00222-8.
Rossignol JF, Bardin M, Fulgencio J, Mogelnicki D, Brechot C. Early treatment with nitazoxanide prevents worsening of mild and moderate COVID-19 and subsequent hospitalization. Vanguard Study, Early Treatment With Nitazoxanide Prevents Worsening of Mild and Moderate COVID-19 and Subsequent Hospitalization. medRxiv 2021.04.19.21255441; doi: https://doi.org/10.1101/2021.04.19.21255441	Rossignol JF, Bardin MC, Fulgencio J, Mogelnicki D, Bréchot C. A randomized double-blind placebo-controlled clinical trial of nitazoxanide for treatment of mild or moderate COVID-19. EClinicalMedicine. 2022;45:101310. doi: 10.1016/j.eclinm.2022.101310.
Low JG, de Alwis R, Chen S, Kalimuddin S, Leong YS, Mah T, Yuen N, Tan HC, Zhang SL, Siim J, Chan Y. A phase 1/2 randomized, double-blinded, placebo controlled ascending dose trial to assess the safety, tolerability and immunogenicity of ARCT-021 in healthy adults. medRxiv 2021.07.01.21259831; doi: https://doi.org/10.1101/2021.07.01.21259831	
Lundgren JD, ACTIV-3/TICO Bamlanivimab Study Group. Clinical and virological response to a neutralizing monoclonal antibody for hospitalized patients with COVID-19. medRxiv 2021.07.19.21260559; doi: https://doi.org/10.1101/2021.07.19.21260559	ACTIV-3/TICO Bamlanivimab Study Group, Lundgren JD, Grund B, Barkauskas CE, Holland TL, Gottlieb RL, Sandkovsky U, Brown SM, Knowlton KU, Self WH, Files DC, Jain MK, Benfield T, Bowdish ME, Leshnower BG, Baker JV, Jensen JU, Gardner EM, Ginde AA, Harris ES, Johansen IS, Markowitz N, Matthay MA, Østergaard L, Chang CC, Goodman AL, Chang W, Dewar RL, Gerry NP, Higgs ES, Highbarger H, Murray DD, Murray TA, Natarajan V,

© 2023 Bai AD et al. *JAMA Network Open*.

	Paredes R, Parmar MKB, Phillips AN, Reilly C, Rupert AW, Sharma S, Shaw-Saliba K, Sherman BT, Teitelbaum M, Wentworth D, Cao H, Klekotka P, Babiker AG, Davey VJ, Gelijns AC, Kan VL, Polizzotto MN, Thompson BT, Lane HC, Neaton JD. Responses to a Neutralizing Monoclonal Antibody for Hospitalized Patients With COVID-19 According to Baseline Antibody and Antigen Levels : A Randomized Controlled Trial. Ann Intern Med. 2022;175(2):234-243. doi: 10.7326/M21-3507.
Liu J, Yang W, Liu Y, Lv C, Ruan L, Zhao C, Huo R, Shen X, Miao Q, Lv W, Li H. Chinese medicine (Q-14) in the Treatment of Patients with Coronavirus Disease 2019 (COVID-19): A Single-center, Open label, Randomised Controlled Trial. medRxiv 2021.01.25.21249417; doi: https://doi.org/10.1101/2021.01.25.21249417	Liu J, Yang W, Liu Y, Lu C, Ruan L, Zhao C, Huo R, Shen X, Miao Q, Lv W, Li H, Shi H, Hu L, Yang Z, Zhang L, Wang B, Dong G, Xian Y, Li B, Zhou Z, Xu C, Chen Y, Bian Y, Guo J, Yang J, Wang J, Qi W, Chen S, Chen Y, Yan B, Wang W, Li J, Xie X, Xu M, Jiang J, Wang G, Cong X, Zhu H, Shi J, Leng L, Li D, Guo L, Huang L. Combination of Hua Shi Bai Du granule (Q-14) and standard care in the treatment of patients with coronavirus disease 2019 (COVID-19): A single-center, open-label, randomized controlled trial. Phytomedicine. 2021;91:153671. doi: 10.1016/j.phymed.2021.153671.
Li J, Hou L, Guo X, Jin P, Wu S, Zhu J, Pan H, Wang X, Song Z, Wan J, Cui L. Heterologous prime-boost immunization with CoronaVac and Convidecia. medRxiv 2021.09.03.21263062; doi: https://doi.org/10.1101/2021.09.03.21263062	Li J, Hou L, Guo X, Jin P, Wu S, Zhu J, Pan H, Wang X, Song Z, Wan J, Cui L, Li J, Chen Y, Wang X, Jin L, Liu J, Shi F, Xu X, Zhu T, Chen W, Zhu F. Heterologous AD5-nCOV plus CoronaVac versus homologous CoronaVac vaccination: a randomized phase 4 trial. Nat Med. 2022 Feb;28(2):401-409. doi: 10.1038/s41591-021-01677-z.
Dupuis J, Laurin P, Tardif JC, Hausermann L, Rosa C, Guertin MC, Thibaudeau K, Gagnon L, Cesari F, Robitaille M, Moran JE. Fourteen-days Evolution of COVID-19 Symptoms During the Third Wave in Non-vaccinated Subjects and Effects of Hesperidin Therapy: A randomized, double-blinded, placebo-controlled study. medRxiv 2021.10.04.21264483; doi: https://doi.org/10.1101/2021.10.04.21264483	
Haran JP, Zheng Y, Knobil K, Alonzo-Palma N, Lawrence J, Wingertzahn M. Targeting the Microbiome With KB109 in Outpatients with Mild to Moderate COVID-19 Reduced Medically Attended Acute Care Visits and Improved Symptom Duration in Patients With Comorbidities. medRxiv 2021.03.26.21254422; doi: https://doi.org/10.1101/2021.03.26.21254422	
Song JY, Kim YS, Eom JS, Kim JY, Lee JS, Lee J, Choi WS, Heo JY, Sohn JW, Lee KD, Cho D. Oral antiviral clevudine compared with placebo in Korean COVID-19 patients with moderate severity. medRxiv 2021.12.09.21267566; doi: https://doi.org/10.1101/2021.12.09.21267566	
Rojas-Serrano J, Ireri AM, Thirion-Romero JV, Alejandra FM, Ramírez-Venegas KM, Pérez-Padilla R. Hydroxychloroquine For Prophylaxis Of COVID-19 In Health Workers: A Randomized Clinical Trial. medRxiv 2021.05.14.21257059; doi: https://doi.org/10.1101/2021.05.14.21257059	Rojas-Serrano J, Portillo-Vásquez AM, Thirion-Romero I, Vázquez-Pérez J, Mejía-Nepomuceno F, Ramírez-Venegas A, Pérez-Kawabe KM, Pérez-Padilla R. Hydroxychloroquine for prophylaxis of COVID-19 in health workers: A randomized clinical trial. PLoS One. 2022;17(2):e0261980. doi: 10.1371/journal.pone.0261980.
Beltran-Gonzalez JL, Gonzalez-Gamez M, Mendoza-Enciso EA, Esparza-Maldonado RJ, Hernanez-Palacios D, Duenas-Campos S, Ovalle-Robles I, Macias-Guzman MJ, Diaz AL, Pena CM, Martinez-Medina L. Efficacy and safety of ivermectin and hydroxychloroquine in patients with severe COVID-19. A randomized controlled trial. medRxiv 2021.02.18.21252037; doi: https://doi.org/10.1101/2021.02.18.21252037.	Beltran Gonzalez JL, González Gámez M, Mendoza Enciso EA, Esparza Maldonado RJ, Hernández Palacios D, Dueñas Campos S, Robles IO, Macías Guzmán MJ, García Díaz AL, Gutiérrez Peña CM, Martinez Medina L, Monroy Colin VA, Arreola Guerra JM. Efficacy and Safety of Ivermectin and Hydroxychloroquine in Patients with Severe COVID-19: A Randomized Controlled Trial. Infect Dis Rep. 2022;14(2):160-168. doi: 10.3390/idr14020020.
Gonzalez JL, Gamez MG, Enciso EA, Maldonado RJ, Palacios DH, Campos SD, Robles IO, Guzman MJ, Diaz AL, Pena CM, Escalera AL. Efficacy and safety of convalescent plasma and intravenous immunoglobulin in critically ill COVID-19 patients. A controlled clinical trial. medRxiv 2021.03.28.21254507; doi: https://doi.org/10.1101/2021.03.28.21254507	
Figueroa, J.M., Lombardo, M., Dogliotti, A., Flynn, L., Giugliano, R.P., Simonelli, G., Valentini, R., Ramos, A., Romano, P., Marcote, M. and Michelini, A., 2021. Efficacy of a nasal spray containing Iota-Carrageenan in the prophylaxis of COVID-19 in hospital personnel dedicated to patients care with COVID-19 disease. A pragmatic multicenter, randomized, double-blind, placebo-controlled trial (CARR-COV-02). medRxiv 2021.04.13.21255409; doi: https://doi.org/10.1101/2021.04.13.21255409.	Figueroa JM, Lombardo ME, Dogliotti A, Flynn LP, Giugliano R, Simonelli G, Valentini R, Ramos A, Romano P, Marcote M, Michelini A, Salvado A, Sykora E, Kniz C, Kobelinsky M, Salzberg DM, Jerusalinsky D, Uchitel O. Efficacy of a Nasal Spray Containing Iota-Carrageenan in the Postexposure Prophylaxis of COVID-19 in Hospital Personnel Dedicated to Patients Care with COVID-19 Disease. Int J Gen Med. 2021;14:6277-6286. doi: 10.2147/IJGM.S328486. PMID: 34629893; PMCID: PMC8493111.
Chew KW, Moser C, Daar ES, Wohl DA, Li JZ, Coombs R, Ritz J, Giganti M, Javan AC, Li Y, Malvestutto C, Klekotka P, Price K, Nirula A, Fischer W, Bala V, Ribeiro RM, Perelson AS,	

© 2023 Bai AD et al. *JAMA Network Open*.

Fletcher CV, Eron JJ, Currier JS, Hughes MD, Smith DM. Bamlanivimab reduces nasopharyngeal SARS-CoV-2 RNA levels but not symptom duration in non-hospitalized adults with COVID-19. medRxiv. 2021.12.17.21268009. doi: 10.1101/2021.12.17.21268009.	
Winthrop KL, Skolnick AW, Rafiq AM, Beegle SH, Suszanski J, Koehne G, Barnett-Griness O, Bibliowicz A, Fathi R, Anderson P, Raday G. Opaganib in COVID-19 pneumonia: Results of a randomized, placebo-controlled Phase 2a trial. medRxiv 2021.08.23.21262464; doi: https://doi.org/10.1101/2021.08.23.21262464	Winthrop KL, Skolnick AW, Rafiq AM, Beegle SH, Suszanski J, Koehne G, Barnett-Griness O, Bibliowicz A, Fathi R, Anderson P, Raday G, Eagle G, Ben-Yair VK, Minkowitz HS, Levitt ML, Gordon MS. Opaganib in Coronavirus Disease 2019 Pneumonia: Results of a Randomized, Placebo-Controlled Phase 2a Trial. Open Forum Infect Dis. 2022 May 11;9(7):ofac232. doi: 10.1093/ofid/ofac232.
Wanaratna K, Leethong P, Inchai N, Chueawiang W, Sriraksa P, Tabmee A, Sirinavin S. Efficacy and safety of Andrographis paniculata extract in patients with mild COVID-19: A randomized controlled trial. medRxiv 2021.07.08.21259912; doi: https://doi.org/10.1101/2021.07.08.21259912	Wanaratna K, Leethong P, Inchai N, Chueawiang W, Sriraksa P, Tabmee A, Sirinavin S. Efficacy and Safety of Andrographis Paniculata Extract in Patients with Mild COVID-19: A Randomized Controlled Trial. Archives of Internal Medicine Research 5 (2022): 423-427.
Damle L, Damle H, Bharath BR. Plant Formulation ATRICOV 452 in Improving the Level of COVID-19 Specific Inflammatory Markers in Patients. medRxiv 2021.10.07.21264491; doi: https://doi.org/10.1101/2021.10.07.21264491	Damle L, Damle H, Br B. Plant formulation ATRICOV 452 in improving the level of COVID-19 specific inflammatory markers in patients. Contemp Clin Trials Commun. 2022 Jul 4;28:100961. doi: 10.1016/j.conctc.2022.100961.
Huang L, Li Q, Sayed SZ, Mohammad N, Chen B, Iftikhar MA, Xie L, Hu J, Chen H. Ultra–short-wave diathermy shortens the course of moderate and severe COVID-19: a randomized trial. medRxiv 2021.01.28.21250163; doi: https://doi.org/10.1101/2021.01.28.21250163	
Dunkle LM, Kotloff KL, Gay CL, Anez G, Adelglass JM, Hernandez AQ, Harper WL, Duncanson DM, McArthur MA, Florescu DF, McClelland S. Efficacy and Safety of NVX-CoV2373 in Adults in the United States and Mexico. medRxiv 2021.10.05.21264567; doi: https://doi.org/10.1101/2021.10.05.21264567	Dunkle LM, Kotloff KL, Gay CL, Áñez G, Adelglass JM, Barrat Hernández AQ, Harper WL, Duncanson DM, McArthur MA, Florescu DF, McClelland RS, Garcia-Fragoso V, Riesenberg RA, Musante DB, Fried DL, Safirstein BE, McKenzie M, Jeanfreau RJ, Kingsley JK, Henderson JA, Lane DC, Ruíz-Palacios GM, Corey L, Neuzil KM, Coombs RW, Greninger AL, Hutter J, Ake JA, Smith K, Woo W, Cho I, Glenn GM, Dubovsky F; 2019nCoV-301 Study Group. Efficacy and Safety of NVX-CoV2373 in Adults in the United States and Mexico. N Engl J Med. 2022;386(6):531-543. doi: 10.1056/NEJMoa2116185.
Maskin LP, Bonelli I, Olarte GL, Palizas F, Velo AE, Lurbet MF, Lovazzano P, Kotsias S, Attie S, Saubidet IL, Baredes ND. High-Versus Low-Dose Dexamethasone for the Treatment of COVID-19-related Acute Respiratory Distress Syndrome: A Multicenter and Randomized Open-label Clinical Trial. medRxiv 2021.09.15.21263597; doi: https://doi.org/10.1101/2021.09.15.21263597	Maskin LP, Bonelli I, Olarte GL, Palizas F Jr, Velo AE, Lurbet MF, Lovazzano P, Kotsias S, Attie S, Lopez Saubidet I, Baredes ND, Setten M, Rodriguez PO. High- Versus Low-Dose Dexamethasone for the Treatment of COVID-19-Related Acute Respiratory Distress Syndrome: A Multicenter, Randomized Open-Label Clinical Trial. J Intensive Care Med. 2022;37(4):491-499. doi: 10.1177/08850666211066799.
Mammen MP, Tebas P, Agnes J, Giffear M, Kraynyak KA, Blackwood E, Amante D, Reuschel EL, Purwar M, Christensen-Quick A, Liu N. Safety and immunogenicity of INO-4800 DNA vaccine against SARS-CoV-2: a preliminary report of a randomized, blinded, placebo-controlled, Phase 2 clinical trial in adults at high risk of viral exposure. medRxiv 2021.05.07.21256652; doi: https://doi.org/10.1101/2021.05.07.21256652	
Silveira MA, De Jong D, dos Santos Galvão EB, Ribeiro JC, Silva TC, Berretta AA, Amorim TC, San Martin RL, da Conceição LF, Gomes MM, Teixeira MB. Efficacy of propolis as an adjunct treatment for hospitalized COVID-19 patients: a randomized, controlled clinical trial. medRxiv 2021.01.08.20248932; doi: https://doi.org/10.1101/2021.01.08.20248932.	Silveira MAD, De Jong D, Berretta AA, Galvão EBDS, Ribeiro JC, Cerqueira-Silva T, Amorim TC, Conceição LFMRD, Gomes MMD, Teixeira MB, Souza SP, Santos MHCAD, San Martin RLA, Silva MO, Lírio M, Moreno L, Sampaio JCM, Mendonça R, Ultchak SS, Amorim FS, Ramos JGR, Batista PBP, Guarda SNFD, Mendes AVA, Passos RDH; BeeCovid Team. Efficacy of Brazilian green propolis (EPP-AF®) as an adjunct treatment for hospitalized COVID-19 patients: A randomized, controlled clinical trial. Biomed Pharmacother. 2021;138:111526. doi: 10.1016/j.biopha.2021.111526.
Toledo-Romani ME, Sanchez LV, Gonzalez MR, Noda LR, Silva CV, Moreno BP, Ramirez BS, Nicado RP, Mugica RG, Garcia TH, Baez GB. Safety and Immunogenicity of anti-SARS CoV-2 vaccine SOBERANA 02 in homologous or heterologous scheme. medRxiv 2021.11.14.21266309; doi: https://doi.org/10.1101/2021.11.14.21266309	Eugenia-Toledo-Romaní M, Verdecia-Sánchez L, Rodríguez-González M, Rodríguez-Noda L, Valenzuela-Silva C, Paredes-Moreno B, Sánchez-Ramírez B, Pérez-Nicado R, González-Mugica R, Hernández-García T, Bergado-Baez G, Pi-Estopiñán F, Cruz-Sui O, Fraga-Quintero A, García-Montero M, Palenzuela-Díaz A, Baró-Román G, Mendoza-Hernández I, Fernandez-Castillo S, Climent-Ruiz Y, Santana-Mederos D, Ramírez Gonzalez U, García-Vega Y, Pérez-Massón B, Guang-Wu-Chen, Boggiano-Ayo T, Ojito-Magaz E, Rivera DG, Valdés-Balbín Y, García-Rivera D, Vérez-Bencomo V; SOBERANA Research Group, Gómez-Maceo Y, Reyes-Matienzo R, Manuel Coviella-Artime J, Morffi-Cinta I, Martínez-Pérez M, Castillo-Quintana I,

© 2023 Bai AD et al. *JAMA Network Open*.

	Garcés-Hechavarría A, Valera-Fernández R, Martínez-Bedoya D, Garrido-Arteaga R, Cardoso-SanJorge F, Quintero Moreno L, Ontivero-Pino I, Teresa Pérez-Guevara M, Morales-García M, Noa-Romero E, Orosa-Vázquez I, Díaz-Hernández M, Rojas G, Tundidor Y, García-López E, Muñoz-Morejon Y, Galano-Frutos E, Rodríguez-Alvarez J, Arteaga A, Medina Nápoles M, Espi Ávila J, Fontanies Fernández M. Safety and immunogenicity of anti-SARS CoV-2 vaccine SOBERANA 02 in homologous or heterologous scheme: Open label phase I and phase IIa clinical trials. Vaccine. 2022 Jul 29;40(31):4220-4230. doi: 10.1016/j.vaccine.2022.05.082.
Toledo-Romani ME, Garcia-Carmenate M, Silva CV, Baldoquin-Rodriguez W, Perez MM, Gonzalez MC, Moreno BP, Hernandez IM, Romero RG, Tabio OS, Villares PV. Efficacy and Safety of SOBERANA 02, a COVID-19 conjugate vaccine in heterologous three doses combination. medRxiv 2021.10.31.21265703; doi: https://doi.org/10.1101/2021.10.31.21265703	
Tsilika M, Taks E, Dolianitis K, Kotsaki A, Leventogiannis K, Damoulari C, Kostoula M, Paneta M, Adamis G, Papanikolaou IC, Stamatelopoulos K. Activate-2: a double-blind randomized trial of BCG vaccination against COVID19 in individuals at risk. medRxiv 2021.05.20.21257520; doi: https://doi.org/10.1101/2021.05.20.21257520	Tsilika M, Taks E, Dolianitis K, Kotsaki A, Leventogiannis K, Damoulari C, Kostoula M, Paneta M, Adamis G, Papanikolaou I, Stamatelopoulos K, Bolanou A, Katsaros K, Delavinia C, Perdios I, Pandi A, Tsiakos K, Proios N, Kalogianni E, Delis I, Skliros E, Akinosoglou K, Perdikouli A, Poulakou G, Milionis H, Athanassopoulou E, Kalpaki E, Efstratiou L, Perraki V, Papadopoulos A, Netea MG, Giamarellos-Bourboulis EJ. ACTIVATE-2: A Double-Blind Randomized Trial of BCG Vaccination Against COVID-19 in Individuals at Risk. Front Immunol. 2022 Jul 5;13:873067. doi: 10.3389/fimmu.2022.873067.
Holubar M, Subramanian AK, Purington N, Hedlin H, Bunning B, Walter K, Bonilla H, Boumis A, Chen M, Clinton K, Dewhurst L. Favipiravir for treatment of outpatients with asymptomatic or uncomplicated COVID-19: a double-blind randomized, placebo-controlled, phase 2 trial. medRxiv 2021.11.22.21266690; doi: https://doi.org/10.1101/2021.11.22.21266690	Holubar M, Subramanian A, Purington N, Hedlin H, Bunning B, Walter KS, Bonilla H, Boumis A, Chen M, Clinton K, Dewhurst L, Epstein C, Jagannathan P, Kaszynski RH, Panu L, Parsonnet J, Ponder EL, Quintero O, Sefton E, Singh U, Soberanis L, Truong H, Andrews JR, Desai M, Khosla C, Maldonado Y. Favipiravir for treatment of outpatients with asymptomatic or uncomplicated COVID-19: a double-blind randomized, placebo-controlled, phase 2 trial. Clin Infect Dis. 2022:ciac312. doi: 10.1093/cid/ciac312.
O’Donnell MR, Grinsztejn B, Cummings MJ, Justman J, Lamb MR, Eckhardt CM, Philip NM, Cheung YK, Gupta V, João E, Pilotto JH. A randomized, double-blind, controlled trial of convalescent plasma in adults with severe COVID-19. medRxiv 2021.03.12.21253373; doi: https://doi.org/10.1101/2021.03.12.21253373	O'Donnell MR, Grinsztejn B, Cummings MJ, Justman JE, Lamb MR, Eckhardt CM, Philip NM, Cheung YK, Gupta V, João E, Pilotto JH, Diniz MP, Cardoso SW, Abrams D, Rajagopalan KN, Borden SE, Wolf A, Sidi LC, Vizzoni A, Veloso VG, Bitan ZC, Scotto DE, Meyer BJ, Jacobson SD, Kantor A, Mishra N, Chauhan LV, Stone EF, Dei Zotti F, La Carpia F, Hudson KE, Ferrara SA, Schwartz J, Stotler BA, Lin WW, Wontakal SN, Shaz B, Briese T, Hod EA, Spitalnik SL, Eisenberger A, Lipkin WI. A randomized double-blind controlled trial of convalescent plasma in adults with severe COVID-19. J Clin Invest. 2021;131(13):e150646. doi: 10.1172/JCI150646.
O'Brien MP, Forleo-Neto E, Musser BJ, Isa F, Chan KC, Sarkar N, Bar KJ, Barnabas RV, Barouch DH, Cohen MS, Hurt CB. Subcutaneous REGEN-COV Antibody Combination for Covid-19 Prevention. medRxiv 2021.06.14.21258567; doi: https://doi.org/10.1101/2021.06.14.21258567	O'Brien MP, Forleo-Neto E, Musser BJ, Isa F, Chan KC, Sarkar N, Bar KJ, Barnabas RV, Barouch DH, Cohen MS, Hurt CB, Burwen DR, Marovich MA, Hou P, Heirman I, Davis JD, Turner KC, Ramesh D, Mahmood A, Hooper AT, Hamilton JD, Kim Y, Purcell LA, Baum A, Kyratsous CA, Krainson J, Perez-Perez R, Mohseni R, Kowal B, DiCioccio AT, Stahl N, Lipsich L, Braunstein N, Herman G, Yancopoulos GD, Weinreich DM; Covid-19 Phase 3 Prevention Trial Team. Subcutaneous REGEN-COV Antibody Combination to Prevent Covid-19. N Engl J Med. 2021;385(13):1184-1195. doi: 10.1056/NEJMoa2109682.
O’Brien MP, Forleo-Neto E, Sarkar N, Isa F, Hou P, Chan KC, Musser BJ, Bar KJ, Barnabas RV, Barouch DH, Cohen MS. Subcutaneous REGEN-COV antibody combination in early SARS-CoV-2 infection. medRxiv 2021.06.14.21258569; doi: https://doi.org/10.1101/2021.06.14.21258569	O'Brien MP, Forleo-Neto E, Sarkar N, Isa F, Hou P, Chan KC, Musser BJ, Bar KJ, Barnabas RV, Barouch DH, Cohen MS, Hurt CB, Burwen DR, Marovich MA, Brown ER, Heirman I, Davis JD, Turner KC, Ramesh D, Mahmood A, Hooper AT, Hamilton JD, Kim Y, Purcell LA, Baum A, Kyratsous CA, Krainson J, Perez-Perez R, Mohseni R, Kowal B, DiCioccio AT, Geba GP, Stahl N, Lipsich L, Braunstein N, Herman G, Yancopoulos GD, Weinreich DM; COVID-19 Phase 3 Prevention Trial Team. Effect of Subcutaneous Casirivimab and Imdevimab Antibody Combination vs Placebo on Development of Symptomatic COVID-19 in Early Asymptomatic SARS-CoV-2 Infection: A Randomized Clinical Trial. JAMA. 2022;327(5):432-441. doi: 10.1001/jama.2021.24939.
Puskarich MA, Ingraham NE, Merck LH, Driver BE, Wacker DA, Black LP, Jones AE, Fletcher CV, South AM, Nelson AC, Murray TA. Effect of losartan on hospitalized patients with	Puskarich MA, Ingraham NE, Merck LH, Driver BE, Wacker DA, Black LP, Jones AE, Fletcher CV, South AM, Murray TA, Lewandowski C, Farhat J, Benoit JL, Biros MH, Cherabuddi K, Chipman JG, Schacker TW, Guirgis FW, Voelker HT, Koopmeiners JS, Tignanelli CJ;

© 2023 Bai AD et al. *JAMA Network Open*.

COVID-19-induced lung injury: A randomized clinical trial. medRxiv 2021.08.25.21262623; doi: https://doi.org/10.1101/2021.08.25.21262623.	Angiotensin Receptor Blocker Based Lung Protective Strategies for Inpatients With COVID-19 (ALPS-IP) Investigators. Efficacy of Losartan in Hospitalized Patients With COVID-19-Induced Lung Injury: A Randomized Clinical Trial. JAMA Netw Open. 2022;5(3):e222735. doi: 10.1001/jamanetworkopen.2022.2735.
Bonelli M, Mrak D, Tobudic S, Sieghart D, Koblischke M, Mandl P, Kornek B, Simader E, Radner H, Perkmann T, Haslacher H. Additional heterologous versus homologous booster vaccination in immunosuppressed patients without SARS-CoV-2 antibody seroconversion after primary mRNA vaccination: a randomized controlled trial. medRxiv 2021.09.05.21263125; doi: https://doi.org/10.1101/2021.09.05.21263125	Bonelli M, Mrak D, Tobudic S, Sieghart D, Koblischke M, Mandl P, Kornek B, Simader E, Radner H, Perkmann T, Haslacher H, Mayer M, Hofer P, Redlich K, Husar-Memmer E, Fritsch-Stork R, Thalhammer R, Stiasny K, Winkler S, Smolen JS, Aberle JH, Zeitlinger M, Heinz LX, Aletaha D. Additional heterologous versus homologous booster vaccination in immunosuppressed patients without SARS-CoV-2 antibody seroconversion after primary mRNA vaccination: a randomised controlled trial. Ann Rheum Dis. 2022;81(5):687-694. doi: 10.1136/annrheumdis-2021-221558.
Sholzberg M, Tang GH, Rahhal H, AlHamzah M, Kreuziger LB, Áinle FN, Alomran F, Alayed K, AlSheef M, AlSumait F, Pompilio CE. Heparin for moderately ill patients with Covid-19. medRxiv 2021.07.08.21259351; doi: https://doi.org/10.1101/2021.07.08.21259351	Sholzberg M, Tang GH, Rahhal H, AlHamzah M, Kreuziger LB, Áinle FN, Alomran F, Alayed K, Alsheef M, AlSumait F, Pompilio CE, Sperlich C, Tangri S, Tang T, Jaksa P, Suryanarayan D, Almarshoodi M, Castellucci LA, James PD, Lillicrap D, Carrier M, Beckett A, Colovos C, Jayakar J, Arsenault MP, Wu C, Doyon K, Andreou ER, Dounaevskaia V, Tseng EK, Lim G, Fralick M, Middeldorp S, Lee AYY, Zuo F, da Costa BR, Thorpe KE, Negri EM, Cushman M, Jüni P; RAPID trial investigators. Effectiveness of therapeutic heparin versus prophylactic heparin on death, mechanical ventilation, or intensive care unit admission in moderately ill patients with covid-19 admitted to hospital: RAPID randomised clinical trial. BMJ. 2021;375:n2400. doi: 10.1136/bmj.n2400.
Li M, Yang J, Wang L, Wu Q, Wu Z, Zheng W, Wang L, Lu W, Deng X, Peng C, Han B. A booster dose is immunogenic and will be needed for older adults who have completed two doses vaccination with CoronaVac: a randomised, double-blind, placebo-controlled, phase 1/2 clinical trial. medRxiv 2021.08.03.21261544; doi: https://doi.org/10.1101/2021.08.03.21261544	Zeng G, Wu Q, Pan H, Li M, Yang J, Wang L, Wu Z, Jiang D, Deng X, Chu K, Zheng W, Wang L, Lu W, Han B, Zhao Y, Zhu F, Yu H, Yin W. Immunogenicity and safety of a third dose of CoronaVac, and immune persistence of a two-dose schedule, in healthy adults: interim results from two single-centre, double-blind, randomised, placebo-controlled phase 2 clinical trials. Lancet Infect Dis. 2022;22(4):483-495. doi: 10.1016/S1473-3099(21)00681-2.
Elgohary MA, Hasan EM, Ibrahim AA, Abdelsalam MF, Abdel-Rahman RZ, Zaki AI, Elaatar MB, Elnagar MT, Emam ME, Hamada MM, Abdel-Hamid TM. Efficacy of Sofosbuvir plus Ledipasvir in Egyptian patients with COVID-19 compared to standard treatment: a randomized controlled trial. medRxiv 2021.05.19.21257429; doi: https://doi.org/10.1101/2021.05.19.21257429	Elgohary MA, Hasan EM, Ibrahim AA, Abdelsalam MFA, Abdel-Rahman RZ, Zaki AI, Elaatar MB, Elnagar MT, Emam ME, Hamada MM, Abdel-Hamid TM, Abdel-Hafez AS, Seadawy MG, Fatoh AR, Elsaied MA, Sakr MA, Elkady AO, Shehata MM, Nawar OM, Selem MA, Abd-Aal MS, Lotfy HH, Elnagdy TR, Helmy S, Mubark MA. Efficacy of Sofosbuvir plus Ledipasvir in Egyptian patients with COVID-19 compared to standard treatment: a randomized controlled trial. J Med Life. 202215(3):350-358. doi: 10.25122/jml-2021-0175.
Javaherian M, Shadmehr A, Keshtkar A, Beigmohammadi MT, Dabbaghipour N, Syed A, Moghaddam BA. Safety and efficacy of Pulmonary physiotherapy in hospitalized patients with severe COVID-19 pneumonia (PPTCOVID): A prospective, randomised, single-blind, controlled trial. medRxiv 2021.04.24.21255892; doi: https://doi.org/10.1101/2021.04.24.21255892.	
Formica N, Mallory R, Albert G, Robinson M, Plested J, Cho I, Robertson A, Dubovsky F, Glenn GM. Evaluation of a SARS-CoV-2 vaccine NVX-CoV2373 in younger and older adults. medRxiv 2021.02.26.21252482; doi: https://doi.org/10.1101/2021.02.26.21252482	Formica N, Mallory R, Albert G, Robinson M, Plested JS, Cho I, Robertson A, Dubovsky F, Glenn GM; 2019nCoV-101 Study Group. Different dose regimens of a SARS-CoV-2 recombinant spike protein vaccine (NVX-CoV2373) in younger and older adults: A phase 2 randomized placebo-controlled trial. PLoS Med. 2021;18(10):e1003769. doi: 10.1371/journal.pmed.1003769.
Morici N, Podda GM, Birocchi S, Bonacchini L, Merli M, Trezzi M, Massaini G, Agostinis M, Carioti G, Serino FS, Gazzaniga G. Enoxaparin for thromboprophylaxis in hospitalized COVID-19 patients: comparison of 40 mg od vs 40 mg bid The X-COVID19 Randomized Clinical Trial. medRxiv 2021.11.17.21266488; doi: https://doi.org/10.1101/2021.11.17.21266488	Morici N, Podda G, Birocchi S, Bonacchini L, Merli M, Trezzi M, Massaini G, Agostinis M, Carioti G, Saverio Serino F, Gazzaniga G, Barberis D, Antolini L, Grazia Valsecchi M, Cattaneo M. Enoxaparin for thromboprophylaxis in hospitalized COVID-19 patients: The X-COVID-19 Randomized Trial. Eur J Clin Invest. 2022;52(5):e13735. doi: 10.1111/eci.13735.
Holubovska O, Bojkova D, Elli S, Bechtel M, Boltz D, Muzzio M, Peng X, Sala F, Cosentino C, Mironenko A, Milde J. Enisamium is an inhibitor of the SARS-CoV-2 RNA polymerase and shows improvement of recovery in COVID-19 patients in an interim analysis of a clinical trial. medRxiv 2021.01.05.21249237; doi: https://doi.org/10.1101/2021.01.05.21249237	

© 2023 Bai AD et al. *JAMA Network Open*.

Babalola OE, Bode CO, Ajayi AA, Alakaloko FM, Akase IE, Otrofanowei E, Salu OB, Adeyemo WL, Ademuyiwa AO, Omilabu S. Ivermectin shows clinical benefits in mild to moderate COVID19: A randomised controlled double blind dose response study in Lagos. medRxiv 2021.01.05.21249131; doi: https://doi.org/10.1101/2021.01.05.21249131	Babalola OE, Bode CO, Ajayi AA, Alakaloko FM, Akase IE, Otrofanowei E, Salu OB, Adeyemo WL, Ademuyiwa AO, Omilabu S. Ivermectin shows clinical benefits in mild to moderate COVID19: a randomized controlled double-blind, dose-response study in Lagos. QJM. 2022;114(11):780-788. doi: 10.1093/qjmed/hcab035.
Bhardwaj P, Godatwar PK, Charan J, Sharma S, Shafi S, Chauhan N, Vyas P, Dutt N, Midha N, Jalandra R, Sharma M. Efficacy and Safety of Ayurveda Intervention AYUSH 64 as add-on therapy for patients with COVID 19 infections: An open labelled, Parallel Group, Randomized controlled clinical trial. medRxiv 2021.08.10.21261836; doi: https://doi.org/10.1101/2021.08.10.21261836	
The ATTACC, ACTIV-4a, and REMAP-CAP Investigators, Patrick R. Lawler, Ewan C. Goligher, Jeffrey S. Berger, Matthew D. Neal, Bryan J. McVerry, Jose C. Nicolau, Michelle N. Gong, Marc Carrier, Robert S. Rosenson, Harmony R. Reynolds, Alexis F. Turgeon, Jorge Escobedo, David T. Huang, Charlotte Ann Bradbury, Brett L. Houston, Lucy Z. Kornblith, Anand Kumar, Susan R. Kahn, Mary Cushman, Zoe McQuilten, Arthur S. Slutsky, Keri S. Kim, Anthony C. Gordon, Bridget-Anne Kirwan, Maria M. Brooks, Alisa M. Higgins, Roger J. Lewis, Elizabeth Lorenzi, Scott M. Berry, Lindsay R. Berry, Derek C. Angus, Colin J. McArthur, Steven A. Webb, Michael E. Farkouh, Judith S. Hochman, Ryan Zarychanski. Therapeutic Anticoagulation in Non-Critically Ill Patients with Covid-19 medRxiv 2021.05.13.21256846; doi: https://doi.org/10.1101/2021.05.13.21256846.	ATTACC Investigators; ACTIV-4a Investigators; REMAP-CAP Investigators, Lawler PR, Goligher EC, Berger JS, Neal MD, McVerry BJ, Nicolau JC, Gong MN, Carrier M, Rosenson RS, Reynolds HR, Turgeon AF, Escobedo J, Huang DT, Bradbury CA, Houston BL, Kornblith LZ, Kumar A, Kahn SR, Cushman M, McQuilten Z, Slutsky AS, Kim KS, Gordon AC, Kirwan BA, Brooks MM, Higgins AM, Lewis RJ, Lorenzi E, Berry SM, Berry LR, Aday AW, Al-Beidh F, Annane D, Arabi YM, Aryal D, Baumann Kreuziger L, Beane A, Bhimani Z, Bihari S, Billett HH, Bond L, Bonten M, Brunkhorst F, Buxton M, Buzgau A, Castellucci LA, Chekuri S, Chen JT, Cheng AC, Chkhikvadze T, Coiffard B, Costantini TW, de Brouwer S, Derde LPG, Detry MA, Duggal A, Džavík V, Effron MB, Estcourt LJ, Everett BM, Fergusson DA, Fitzgerald M, Fowler RA, Galanaud JP, Galen BT, Gandotra S, García-Madrona S, Girard TD, Godoy LC, Goodman AL, Goossens H, Green C, Greenstein YY, Gross PL, Hamburg NM, Haniffa R, Hanna G, Hanna N, Hegde SM, Hendrickson CM, Hite RD, Hindenburg AA, Hope AA, Horowitz JM, Horvat CM, Hudock K, Hunt BJ, Husain M, Hyzy RC, Iyer VN, Jacobson JR, Jayakumar D, Keller NM, Khan A, Kim Y, Kindzelski AL, King AJ, Knudson MM, Kornblith AE, Krishnan V, Kutcher ME, Laffan MA, Lamontagne F, Le Gal G, Leeper CM, Leifer ES, Lim G, Lima FG, Linstrum K, Litton E, Lopez-Sendon J, Lopez-Sendon Moreno JL, Lother SA, Malhotra S, Marcos M, Saud Marinez A, Marshall JC, Marten N, Matthay MA, McAuley DF, McDonald EG, McGlothlin A, McGuinness SP, Middeldorp S, Montgomery SK, Moore SC, Morillo Guerrero R, Mouncey PR, Murthy S, Nair GB, Nair R, Nichol AD, Nunez-Garcia B, Pandey A, Park PK, Parke RL, Parker JC, Parnia S, Paul JD, Pérez González YS, Pompilio M, Prekker ME, Quigley JG, Rost NS, Rowan K, Santos FO, Santos M, Olombrada Santos M, Satterwhite L, Saunders CT, Schutgens REG, Seymour CW, Siegal DM, Silva DG Jr, Shankar-Hari M, Sheehan JP, Singhal AB, Solvason D, Stanworth SJ, Tritschler T, Turner AM, van Bentum-Puijk W, van de Veerdonk FL, van Diepen S, Vazquez-Grande G, Wahid L, Wareham V, Wells BJ, Widmer RJ, Wilson JG, Yuriditsky E, Zampieri FG, Angus DC, McArthur CJ, Webb SA, Farkouh ME, Hochman JS, Zarychanski R. Therapeutic Anticoagulation with Heparin in Noncritically Ill Patients with Covid-19. N Engl J Med. 2021;385(9):790-802. doi: 10.1056/NEJMoa2105911.
Goepfert PA, Fu B, Chabanon AL, Bonaparte MI, Davis MG, Essink BJ, Frank I, Haney O, Janosczyk H, Keefer MC, Koutsoukos M. Safety and immunogenicity of SARS-CoV-2 recombinant protein vaccine formulations in healthy adults: a randomised, placebo-controlled, dose-ranging study. medRxiv 2021.01.19.20248611; doi: https://doi.org/10.1101/2021.01.19.20248611	Goepfert PA, Fu B, Chabanon AL, Bonaparte MI, Davis MG, Essink BJ, Frank I, Haney O, Janosczyk H, Keefer MC, Koutsoukos M, Kimmel MA, Masotti R, Savarino SJ, Schuerman L, Schwartz H, Sher LD, Smith J, Tavares-Da-Silva F, Gurunathan S, DiazGranados CA, de Bruyn G. Safety and immunogenicity of SARS-CoV-2 recombinant protein vaccine formulations in healthy adults: interim results of a randomised, placebo-controlled, phase 1-2, dose-ranging study. Lancet Infect Dis. 2021;21(9):1257-1270. doi: 10.1016/S1473-3099(21)00147-X.
Gutierrez-Castrellon P, Gandara-Martí T, y Abreu AT, Nieto-Rufino CD, Lopez-Orduna E, Jiménez-Escobar I, Jiménez-Gutiérrez C, Lopez-Vazquez G, Espadaler-Mazo J. Efficacy and safety of novel probiotic formulation in adult Covid19 outpatients: a randomized, placebo-controlled clinical trial. medRxiv 2021.05.20.21256954; doi: https://doi.org/10.1101/2021.05.20.21256954	Gutiérrez-Castrellón P, Gandara-Martí T, Abreu Y Abreu AT, Nieto-Rufino CD, López-Orduña E, Jiménez-Escobar I, Jiménez-Gutiérrez C, López-Velazquez G, Espadaler-Mazo J. Probiotic improves symptomatic and viral clearance in Covid19 outpatients: a randomized, quadruple-blinded, placebo-controlled trial. Gut Microbes. 2022;14(1):2018899. doi: 10.1080/19490976.2021.2018899.

© 2023 Bai AD et al. *JAMA Network Open*.

RECOVERY Collaborative Group. Aspirin in patients admitted to hospital with COVID-19 (RECOVERY): a randomised, controlled, open-label, platform trial. medRxiv 2021.06.08.21258132; doi: https://doi.org/10.1101/2021.06.08.21258132	RECOVERY Collaborative Group. Aspirin in patients admitted to hospital with COVID-19 (RECOVERY): a randomised, controlled, open-label, platform trial. Lancet. 2022 Jan 8;399(10320):143-151. doi: 10.1016/S0140-6736(21)01825-0.
RECOVERY Collaborative Group. Convalescent plasma in patients admitted to hospital with COVID-19 (RECOVERY): a randomised, controlled, open-label, platform trial. medRxiv 2021.03.09.21252736; doi: https://doi.org/10.1101/2021.03.09.21252736	RECOVERY Collaborative Group. Convalescent plasma in patients admitted to hospital with COVID-19 (RECOVERY): a randomised controlled, open-label, platform trial. Lancet. 2021;397(10289):2049-2059. doi: 10.1016/S0140-6736(21)00897-7.
RECOVERY Collaborative Group. Casirivimab and imdevimab in patients admitted to hospital with COVID-19 (RECOVERY): a randomised, controlled, open-label, platform trial. medRxiv 2021.06.15.21258542; doi: https://doi.org/10.1101/2021.06.15.21258542	RECOVERY Collaborative Group. Casirivimab and imdevimab in patients admitted to hospital with COVID-19 (RECOVERY): a randomised, controlled, open-label, platform trial. Lancet. 2022;399(10325):665-676. doi: 10.1016/S0140-6736(22)00163-5.
RECOVERY Collaborative Group. Colchicine in patients admitted to hospital with COVID-19 (RECOVERY): a randomised, controlled, open-label, platform trial. medRxiv 2021.05.18.21257267; doi: https://doi.org/10.1101/2021.05.18.21257267	RECOVERY Collaborative Group. Colchicine in patients admitted to hospital with COVID-19 (RECOVERY): a randomised, controlled, open-label, platform trial. Lancet Respir Med. 2021;9(12):1419-1426. doi: 10.1016/S2213-2600(21)00435-5.
RECOVERY Collaborative Group. Tocilizumab in patients admitted to hospital with COVID-19 (RECOVERY): preliminary results of a randomised, controlled, open-label, platform trial. medRxiv 2021.02.11.21249258; doi: https://doi.org/10.1101/2021.02.11.21249258	RECOVERY Collaborative Group. Tocilizumab in patients admitted to hospital with COVID-19 (RECOVERY): a randomised, controlled, open-label, platform trial. Lancet. 2021;397(10285):1637-1645. doi: 10.1016/S0140-6736(21)00676-0.
Gobeil P, Pillet S, Séguin A, Boulay I, Mahmood A, Vinh DC, Charland N, Boutet P, Roman FP, Van Der Most R, Perez MD. Interim report of a phase 2 randomized trial of a plant-produced virus-like particle vaccine for Covid-19 in healthy adults aged 18-64 and older adults aged 65 and older. medRxiv 2021.05.14.21257248; doi: https://doi.org/10.1101/2021.05.14.21257248	
Bégin P, Callum J, Jamula E, Cook R, Heddle NM, Tinmouth A, Zeller MP, Beaudoin-Bussières G, Amorim L, Bazin R, Loftsgard KC. Convalescent plasma for hospitalized patients with COVID-19 and the effect of plasma antibodies: a randomized controlled, open-label trial. medRxiv 2021.06.29.21259427; doi: https://doi.org/10.1101/2021.06.29.21259427	Bégin P, Callum J, Jamula E, Cook R, Heddle NM, Tinmouth A, Zeller MP, Beaudoin-Bussières G, Amorim L, Bazin R, Loftsgard KC, Carl R, Chassé M, Cushing MM, Daneman N, Devine DV, Dumaresq J, Fergusson DA, Gabe C, Glesby MJ, Li N, Liu Y, McGeer A, Robitaille N, Sachais BS, Scales DC, Schwartz L, Shehata N, Turgeon AF, Wood H, Zarychanski R, Finzi A; CONCOR-1 Study Group, Arnold DM. Convalescent plasma for hospitalized patients with COVID-19: an open-label, randomized controlled trial. Nat Med. 2021;27(11):2012-2024. doi: 10.1038/s41591-021-01488-2.
Mallory R, Formica N, Pfeiffer S, Wilkinson B, Marcheschi A, Albert G, McFall H, Robinson M, Plested J, Zhu M, Cloney-Clark S. Immunogenicity and Safety Following a Homologous Booster Dose of a SARS-CoV-2 recombinant spike protein vaccine (NVX-CoV2373): A Phase 2 Randomized Placebo-Controlled Trial. medRxiv 2021.12.23.21267374; doi: https://doi.org/10.1101/2021.12.23.21267374	Mallory RM, Formica N, Pfeiffer S, Wilkinson B, Marcheschi A, Albert G, McFall H, Robinson M, Plested JS, Zhu M, Cloney-Clark S, Zhou B, Chau G, Robertson A, Maciejewski S, Hammond HL, Baracco L, Logue J, Frieman MB, Smith G, Patel N, Glenn GM; Novavax 2019nCoV101 Study Group. Safety and immunogenicity following a homologous booster dose of a SARS-CoV-2 recombinant spike protein vaccine (NVX-CoV2373): a secondary analysis of a randomised, placebo-controlled, phase 2 trial. Lancet Infect Dis. 2022 Aug 10:S1473-3099(22)00420-0. doi: 10.1016/S1473-3099(22)00420-0.
Ella R, Reddy S, Blackwelder W, Potdar V, Yadav P, Sarangi V, Aileni VK, Kanungo S, Rai S, Reddy P, Verma S. Efficacy, safety, and lot to lot immunogenicity of an inactivated SARS-CoV-2 vaccine (BBV152): a double-blind, randomised, controlled phase 3 trial. medRxiv 2021.06.30.21259439; doi: https://doi.org/10.1101/2021.06.30.21259439	Ella R, Reddy S, Blackwelder W, Potdar V, Yadav P, Sarangi V, Aileni VK, Kanungo S, Rai S, Reddy P, Verma S, Singh C, Redkar S, Mohapatra S, Pandey A, Ranganadin P, Gumashta R, Multani M, Mohammad S, Bhatt P, Kumari L, Sapkal G, Gupta N, Abraham P, Panda S, Prasad S, Bhargava B, Ella K, Vadrevu KM; COVAXIN Study Group. Efficacy, safety, and lot-to-lot immunogenicity of an inactivated SARS-CoV-2 vaccine (BBV152): interim results of a randomised, double-blind, controlled, phase 3 trial. Lancet. 2021;398(10317):2173-2184.
Lazarus R, Taucher C, Duncan C, Faust S, Green CA, Finn A. Immunogenicity and safety of inactivated whole virion Coronavirus vaccine with CpG (VLA2001) in healthy adults aged 18 to 55: a randomised phase 1/2 clinical trial. medRxiv 2021.08.13.21262021; doi: https://doi.org/10.1101/2021.08.13.21262021	Lazarus R, Taucher C, Brown C, Čorbic Ramljak I, Danon L, Dubischar K, Duncan CJA, Eder-Lingelbach S, Faust SN, Green C, Gokani K, Hochreiter R, Wright JK, Kwon D, Middleditch A, Munro APS, Naker K, Penciu F, Price D, Querton B, Riaz T, Ross-Russell A, Sanchez-Gonzalez A, Wardle H, Warren S, Finn A; Valneva Phase 1 Trial Group. Safety and immunogenicity of the inactivated whole-virus adjuvanted COVID-19 vaccine VLA2001: A randomized, dose escalation, double-blind phase 1/2 clinical trial in healthy adults. J Infect. 2022 Sep;85(3):306-317. doi: 10.1016/j.jinf.2022.06.009.
Amaravadi RK, Giles L, Carberry M, Hyman MC, Frank I, Nasta SD, Walsh J, Wileyto EP, Gimotty P, Milone M, Teng EM. Hydroxychloroquine for SARS-CoV-2 positive patients	

© 2023 Bai AD et al. *JAMA Network Open*.

quarantined at home: The first interim analysis of a remotely conducted randomized clinical trial. medRxiv 2021.02.22.21252228; doi: https://doi.org/10.1101/2021.02.22.21252228	
Ravakirti, Roy R, Pattadar C, Raj R, Agarwal N, Biswas B, Majhi PK, Rai DK, Kumar A, Sarfaraz A. Ivermectin as a potential treatment for mild to moderate COVID-19–a double blind randomized placebo-controlled trial. medRxiv 2021.01.05.21249310; doi: https://doi.org/10.1101/2021.01.05.21249310	Ravikirti, Roy R, Pattadar C, Raj R, Agarwal N, Biswas B, Manjhi PK, Rai DK, Shyama, Kumar A, Sarfaraz A. Evaluation of Ivermectin as a Potential Treatment for Mild to Moderate COVID-19: A Double-Blind Randomized Placebo Controlled Trial in Eastern India. J Pharm Pharm Sci. 2021;24:343-350. doi: 10.18433/jpps32105.
Ravichandran R, Mohan SK, Sukumaran SK, Kamaraj D, Daivasuga SS, Ravi SO, Vijayaraghavalu S, Kumar RK. Use of Indomethacin for mild and moderate Covid-19 patients A Randomized Control Trial. medRxiv 2021.07.24.21261007; doi: https://doi.org/10.1101/2021.07.24.21261007	Ravichandran R, Mohan SK, Sukumaran SK, Kamaraj D, Daivasuga SS, Ravi SOAS, Vijayaraghavalu S, Kumar RK. An open label randomized clinical trial of Indomethacin for mild and moderate hospitalised Covid-19 patients. Sci Rep. 2022;12(1):6413. doi: 10.1038/s41598-022-10370-1.
ElZein R, Fakhreddine S, Abi Hanna P, Feghali R, Hamad H, Ayoub F. In vivo evaluation of the virucidal efficacy of Chlorhexidine and Povidone-iodine mouthwashes against salivary SARS-CoV-2. medRxiv 2021.03.07.21252302; doi: https://doi.org/10.1101/2021.03.07.21252302	Elzein R, Abdel-Sater F, Fakhreddine S, Hanna PA, Feghali R, Hamad H, Ayoub F. In vivo evaluation of the virucidal efficacy of chlorhexidine and povidone-iodine mouthwashes against salivary SARS-CoV-2. A randomized-controlled clinical trial. J Evid Based Dent Pract. 2021;21(3):101584. doi: 10.1016/j.jebdp.2021.101584.
Sablerolles R, Rietdijk W, Goorhuis B, Postma D, Visser L, Geers D, Schmitz K, Garrido HG, Koopmans M, Dalm V, Kootstra NA. Immunogenicity and reactogenicity of booster vaccinations after Ad26. COV2. S priming. medRxiv 2021.10.18.21264979; doi: https://doi.org/10.1101/2021.10.18.21264979	Sablerolles RSG, Rietdijk WJR, Goorhuis A, Postma DF, Visser LG, Geers D, Schmitz KS, Garcia Garrido HM, Koopmans MPG, Dalm VASH, Kootstra NA, Huckriede ALW, Lafeber M, van Baarle D, GeurtsvanKessel CH, de Vries RD, van der Kuy PHM; SWITCH Research Group. Immunogenicity and Reactogenicity of Vaccine Boosters after Ad26.COV2.S Priming. N Engl J Med. 2022;386(10):951-963. doi: 10.1056/NEJMoa2116747.
Chahla RE, Ruiz LM, Ortega ES, Morales MF, Barreiro F, George A, Mansilla C, D'Amato SP, Barrenechea G, Goroso GD. A randomized trial-intensive treatment based in ivermectin and iota-carrageenan as pre-exposure prophylaxis for COVID-19 in healthcare agents. medRxiv 2021.03.26.21254398; doi: https://doi.org/10.1101/2021.03.26.21254398	Chahla RE, Medina Ruiz L, Ortega ES, Morales Rn MF, Barreiro F, George A, Mancilla Rn C, D' Amato Rn S, Barrenechea G, Goroso DG, Peral de Bruno M. Intensive Treatment With Ivermectin and Iota-Carrageenan as Pre-exposure Prophylaxis for COVID-19 in Health Care Workers From Tucuman, Argentina. Am J Ther. 2021;28(5):e601-e604. doi: 10.1097/MJT.0000000000001433.
Chahla RE, Ruiz LM, Mena T, Brepe Y, Terranova P, Ortega ES, Barrenechea GG, Goroso DG. Ivermectin reproposing for COVID-19 treatment outpatients in mild stage in primary health care centers. medRxiv 2021.03.29.21254554; doi: https://doi.org/10.1101/2021.03.29.21254554	
Izikson R, Brune D, Bolduc JS, Bourron P, Fournier M, Moore TM, Pandey A, Perez L, Sater N, Shrestha A, Wague S. Safety and immunogenicity of a high-dose quadrivalent influenza vaccine administered concomitantly with a third dose of the mRNA-1273 SARS-CoV-2 vaccine in adults ≥ 65 years of age: a Phase II, open-label study. medRxiv 2021.10.29.21265248; doi: https://doi.org/10.1101/2021.10.29.21265248	Izikson R, Brune D, Bolduc JS, Bourron P, Fournier M, Moore TM, Pandey A, Perez L, Sater N, Shrestha A, Wague S, Samson SI. Safety and immunogenicity of a high-dose quadrivalent influenza vaccine administered concomitantly with a third dose of the mRNA-1273 SARS-CoV-2 vaccine in adults aged ≥65 years: a phase 2, randomised, open-label study. Lancet Respir Med. 2022;10(4):392-402. doi: 10.1016/S2213-2600(21)00557-9.
Ramakrishnan S, Nicolau Jr DV, Langford B, Mahdi M, Jeffers H, Mwasuku C, Krassowska K, Fox R, Binnian I, Glover V, Bright S. Inhaled budesonide in the treatment of early COVID-19 illness: a randomised controlled trial. medRxiv 2021.02.04.21251134; doi: https://doi.org/10.1101/2021.02.04.21251134	Ramakrishnan S, Nicolau Jr DV, Langford B, Mahdi M, Jeffers H, Mwasuku C, Krassowska K, Fox R, Binnian I, Glover V, Bright S. Inhaled budesonide in the treatment of early COVID-19 (STOIC): a phase 2, open-label, randomised controlled trial. The Lancet Respiratory Medicine. 2021;9(7):763-72. doi: 10.1016/S2213-2600(21)00160-0.
Sridhar S, Arnel J, Bonaparte MI, Bueso A, Chabanon AL, Chen A, Chicz RM, Diemert D, Essink BJ, Fu B, Grunenberg NA. Safety and immunogenicity of a SARS-CoV-2 recombinant protein vaccine with AS03 adjuvant in healthy adults: interim findings from a phase 2, randomised, dose-finding, multi-centre study. medRxiv 2021.10.08.21264302; doi: https://doi.org/10.1101/2021.10.08.21264302	Sridhar S, Joaquin A, Bonaparte MI, Bueso A, Chabanon AL, Chen A, Chicz RM, Diemert D, Essink BJ, Fu B, Grunenberg NA, Janosczyk H, Keefer MC, Rivera M DM, Meng Y, Michael NL, Munsiff SS, Ogbuagu O, Raabe VN, Severance R, Rivas E, Romanyak N, Rouphael NG, Schuerman L, Sher LD, Walsh SR, White J, von Barbier D, de Bruyn G, Canter R, Grillet MH, Keshtkar-Jahromi M, Koutsoukos M, Lopez D, Masotti R, Mendoza S, Moreau C, Ceregido MA, Ramirez S, Said A, Tavares-Da-Silva F, Shi J, Tong T, Treanor J, Diazgranados CA, Savarino S. Safety and immunogenicity of an AS03-adjuvanted SARS-CoV-2 recombinant protein vaccine (CoV2 preS dTM) in healthy adults: interim findings from a phase 2, randomised, dose-finding, multicentre study. Lancet Infect Dis. 2022;22(5):636-648. doi: 10.1016/S1473-3099(21)00764-7.
Somersan-Karakaya S, Mylonakis E, Menon VP, Wells JC, Ali S, Sivapalasingam S, Sun Y, Bhore R, Mei J, Miller J, Cupelli L. REGEN-COV® for Treatment of Hospitalized Patients with Covid-19. medRxiv 2021.11.05.21265656; doi: https://doi.org/10.1101/2021.11.05.21265656	Somersan-Karakaya S, Mylonakis E, Menon VP, Wells JC, Ali S, Sivapalasingam S, Sun Y, Bhore R, Mei J, Miller J, Cupelli L, Forleo-Neto E, Hooper AT, Hamilton JD, Pan C, Pham V, Zhao Y, Hosain R, Mahmood A, Davis JD, Turner KC, Kim Y, Cook A, Kowal B, Soo Y, DiCioccio AT, Geba GP, Stahl N, Lipsich L, Braunstein N, Herman GA, Yancopoulos GD,

	Weinreich DM; COVID-19 Phase 2/3 Hospitalized Trial Team. Casirivimab and Imdevimab for the Treatment of Hospitalized Patients With COVID-19. J Infect Dis. 2022 Jul 27:jiac320. doi: 10.1093/infdis/jiac320.
Fragoso-Saavedra S, Núñez I, Audelo-Cruz BM, Arias-Martínez S, Manzur-Sandoval D, Quintero-Villegas A, García-González HB, Carbajal-Morelos SL, de León-Rosales SP, Gotés-Palazuelos J, Caro-Vega Y. Pyridostigmine in adults with severe SARS-CoV-2 infection: the PISCO trial. medRxiv 2021.04.28.21255834; doi: https://doi.org/10.1101/2021.04.28.21255834	
Heath PT, Galiza EP, Baxter DN, Boffito M, Browne D, Burns F, Chadwick DR, Clark R, Cosgrove C, Galloway J, Goodman AL, Heer A, Higham A, Iyengar S, Jamal A, Jeanes C, Kalra PA, Kyriakidou C, McAuley DF, Meyrick A, Minassian AM, Minton J, Moore P, Munsoor I, Nicholls H, Osanlou O, Packham J, Pretswell CH, San Francisco Ramos A, Saralaya D, Sheridan RP, Smith R, Soiza RL, Swift PA, Thomson EC, Turner J, Viljoen ME, Albert G, Cho I, Dubovsky F, Glenn G, Rivers J, Robertson A, Smith K, Toback S; 2019nCoV-302 Study Group. Efficacy of the NVX-CoV2373 Covid-19 Vaccine Against the B.1.1.7 Variant. medRxiv 2021.05.13.21256639; doi: https://doi.org/10.1101/2021.05.13.21256639	Heath PT, Galiza EP, Baxter DN, Boffito M, Browne D, Burns F, Chadwick DR, Clark R, Cosgrove C, Galloway J, Goodman AL, Heer A, Higham A, Iyengar S, Jamal A, Jeanes C, Kalra PA, Kyriakidou C, McAuley DF, Meyrick A, Minassian AM, Minton J, Moore P, Munsoor I, Nicholls H, Osanlou O, Packham J, Pretswell CH, San Francisco Ramos A, Saralaya D, Sheridan RP, Smith R, Soiza RL, Swift PA, Thomson EC, Turner J, Viljoen ME, Albert G, Cho I, Dubovsky F, Glenn G, Rivers J, Robertson A, Smith K, Toback S; 2019nCoV-302 Study Group. Safety and Efficacy of NVX-CoV2373 Covid-19 Vaccine. N Engl J Med. 2021;385(13):1172-1183. doi: 10.1056/NEJMoa2107659.
Panda PK, SEV COVID trial group. Safety and efficacy of antiviral therapy alone or in combination in COVID-19-a randomized controlled trial (SEV COVID Trial). medRxiv 2021.06.06.21258091; doi: https://doi.org/10.1101/2021.06.06.21258091	
Madhi SA, Baillie VL, Cutland CL, Voysey M, Koen AL, Fairlie L, Padayachee SD, Dheda K, Barnabas SL, Bhorat QE, Briner C. Safety and efficacy of the ChAdOx1 nCoV-19 (AZD1222) Covid-19 vaccine against the B. 1.351 variant in South Africa. medRxiv 2021.02.10.21251247; doi: https://doi.org/10.1101/2021.02.10.21251247	Madhi SA, Baillie V, Cutland CL, Voysey M, Koen AL, Fairlie L, Padayachee SD, Dheda K, Barnabas SL, Bhorat QE, Briner C, Kwatra G, Ahmed K, Aley P, Bhikha S, Bhiman JN, Bhorat AE, du Plessis J, Esmail A, Groenewald M, Horne E, Hwa SH, Jose A, Lambe T, Laubscher M, Malahleha M, Masenya M, Masilela M, McKenzie S, Molapo K, Moultrie A, Oelofse S, Patel F, Pillay S, Rhead S, Rodel H, Rossouw L, Taoushanis C, Tegally H, Thombrayil A, van Eck S, Wibmer CK, Durham NM, Kelly EJ, Villafana TL, Gilbert S, Pollard AJ, de Oliveira T, Moore PL, Sigal A, Izu A; NGS-SA Group; Wits-VIDA COVID Group. Efficacy of the ChAdOx1 nCoV-19 Covid-19 Vaccine against the B.1.351 Variant. N Engl J Med. 2021;384(20):1885-1898. doi: 10.1056/NEJMoa2102214.
Shenoy S, Munjal S, Al Youha S, Alghounaim M, Almazeedi S, Alshamali Y, Kaszynski RH, Al-Sabah S, Kuwait Clinical Trial Group. Favipiravir In Adults with Moderate to Severe COVID-19: A Phase 3 Multicentre, Randomized, Double-Blinded, Placebo-Controlled Trial. medRxiv 2021.11.08.21265884; doi: https://doi.org/10.1101/2021.11.08.21265884	
Shoham S, Bloch EM, Casadevall A, Hanley D, Lau B, Gebo K, Cachay E, Kassaye SG, Paxton JH, Gerber J, Levine AC. Randomized controlled trial transfusing convalescent plasma as post-exposure prophylaxis against SARS-CoV-2 infection. medRxiv 2021.12.13.21267611; doi: https://doi.org/10.1101/2021.12.13.21267611	Shoham S, Bloch EM, Casadevall A, Hanley D, Lau B, Gebo K, Cachay E, Kassaye SG, Paxton JH, Gerber J, Levine AC, Naeim A, Currier J, Patel B, Allen ES, Anjan S, Appel L, Baksh S, Blair PW, Bowen A, Broderick P, Caputo CA, Cluzet V, Elena MC, Cruser D, Ehrhardt S, Forthal D, Fukuta Y, Gawad AL, Gniadek T, Hammel J, Huaman MA, Jabs DA, Jedlicka A, Karlen N, Klein S, Laeyendecker O, Karen L, McBee N, Meisenberg B, Merlo C, Mosnaim G, Park HS, Pekosz A, Petrini J, Rausch W, Shade DM, Shapiro JR, Singleton RJ, Sutcliffe C, Thomas DL, Yarava A, Zand M, Zenilman JM, Tobian AAR, Sullivan DJ. Transfusing convalescent plasma as post-exposure prophylaxis against SARS-CoV-2 infection: a double-blinded, phase 2 randomized, controlled trial. Clin Infect Dis. 2022 May 17:ciac372. doi: 10.1093/cid/ciac372.
Méndez-Flores S, Priego-Ranero Á, Azamar-Llamas D, Olvera-Prado H, Rivas-Redondo KI, Ochoa-Hein E, Perez-Ortiz A, Rojas-Castañeda E, Urbina-Terán S, Septién-Stute L, Hernández-Gilsoul T. Effect of polymerized type I collagen in hyperinflammation of adult outpatients with symptomatic COVID-19: a double blind, randomised, placebo-controlled clinical trial. medRxiv 2021.05.12.21257133; doi: https://doi.org/10.1101/2021.05.12.21257133	Méndez-Flores S, Priego-Ranero Á, Azamar-Llamas D, Olvera-Prado H, Rivas-Redonda KI, Ochoa-Hein E, Perez-Ortiz A, Rendón-Macías ME, Rojas-Castañeda E, Urbina-Terán S, Septién-Stute L, Hernández-Gilsoul T, Aguilar-Morgan AA, Fernández-Camargo DA, Olivares-Martínez E, Hernández-Ramírez DF, Torres-Villalobos G, Furuzawa-Carballeda J. Effect of polymerised type I collagen on hyperinflammation of adult outpatients with symptomatic COVID-19. Clin Transl Med. 2022;12(3):e763. doi: 10.1002/ctm2.763.
Körper S, Weiss M, Zickler D, Wiesmann T, Zacharowski K, Corman VM, Grüner B, Ernst L, Spieth P, Lepper PM, Bentz M. High dose convalescent plasma in COVID-19: results from the	Körper S, Weiss M, Zickler D, Wiesmann T, Zacharowski K, Corman VM, Grüner B, Ernst L, Spieth P, Lepper PM, Bentz M, Zinn S, Paul G, Kalbhenn J, Dollinger MM, Rosenberger P, Kirschning T, Thiele T, Appl T, Mayer B, Schmidt M, Drosten C, Wulf H, Kruse JM, Jungwirth

© 2023 Bai AD et al. *JAMA Network Open*.

randomized trial CAPSID. medRxiv 2021.05.10.21256192; doi: https://doi.org/10.1101/2021.05.10.21256192	B, Seifried E, Schrezenmeier H; CAPSID Clinical Trial Group. Results of the CAPSID randomized trial for high-dose convalescent plasma in patients with severe COVID-19. J Clin Invest. 2021;131(20):e152264. doi: 10.1172/JCI152264.
Betka SJ, Kannape OA, Fasola J, Lance F, Cardin S, Schmid A, Similowski T, Soccal MP, Herbelin B, Adler D, Blanke O. Virtual reality exercise to help COVID patients with refractory breathlessness. medRxiv 2021.10.26.21265510; doi: https://doi.org/10.1101/2021.10.26.21265510	
Thomas SJ, Moreira ED, Kitchin N, Absalon J, Gurtman A, Lockhart S, Perez JL, Marc GP, Polack FP, Zerbini C, Bailey R. Six Month safety and efficacy of the BNT162b2 Mrna Covid-19 vaccine. medRxiv 2021.07.28.21261159; doi: https://doi.org/10.1101/2021.07.28.21261159	Thomas SJ, Moreira ED Jr, Kitchin N, Absalon J, Gurtman A, Lockhart S, Perez JL, Pérez Marc G, Polack FP, Zerbini C, Bailey R, Swanson KA, Xu X, Roychoudhury S, Koury K, Bouguermouh S, Kalina WV, Cooper D, Frenck RW Jr, Hammitt LL, Türeci Ö, Nell H, Schaefer A, Ünal S, Yang Q, Liberator P, Tresnan DB, Mather S, Dormitzer PR, Şahin U, Gruber WC, Jansen KU; C4591001 Clinical Trial Group. Safety and Efficacy of the BNT162b2 mRNA Covid-19 Vaccine through 6 Months. N Engl J Med. 2021;385(19):1761-1773. doi: 10.1056/NEJMoa2110345.
Sivapalasingam S, Lederer D, Bhore R, Hajizadeh N, Criner G, Hosain R, Mahmood A, Giannelou A, Somersan-Karakaya S, O'Brien M, Boyapati A. A randomized placebo-controlled trial of sarilumab in hospitalized patients with Covid-19. medRxiv 2021.05.13.21256973; doi: https://doi.org/10.1101/2021.05.13.21256973	Sivapalasingam S, Lederer DJ, Bhore R, Hajizadeh N, Criner G, Hosain R, Mahmood A, Giannelou A, Somersan-Karakaya S, O'Brien MP, Boyapati A, Parrino J, Musser BJ, Labriola-Tompkins E, Ramesh D, Purcell LA, Gulabani D, Kampman W, Waldron A, Gong MN, Saggar S, Sperber SJ, Menon V, Stein DK, Sobieszczyk ME, Park W, Aberg JA, Brown SM, Kosmicki JA, Horowitz JE, Ferreira MA, Baras A, Kowal B, DiCioccio AT, Akinlade B, Nivens MC, Braunstein N, Herman GA, Yancopoulos GD, Weinreich DM; Sarilumab-COVID-19 Study Team. Efficacy and Safety of Sarilumab in Hospitalized Patients With COVID-19: A Randomized Clinical Trial. Clin Infect Dis. 2022:ciac153. doi: 10.1093/cid/ciac153.
Patankar SB, Rangnekar H, Joshi K, Suryawanshi K, Soni P, Gorde A, Shah T, Patankar S, Jha D, Raje R. Efficacy and Safety of Polyherbal formulation as an add-on to the standard of care in mild to moderate COVID-19: A randomized, double-blind, placebo-controlled trial. medRxiv 2021.05.14.21256900; doi: https://doi.org/10.1101/2021.05.14.21256900	
Naggie S, Milstone A, Castro M, Collins SP, Seetha L, Anderson DJ, Cahuayme-Zuniga L, Batey-Turner K, Cohen LW, Fraulo E, Friedland A. Hydroxychloroquine for pre-exposure prophylaxis of COVID-19 in health care workers: a randomized, multicenter, placebo-controlled trial (HERO-HCQ). medRxiv 2021.08.19.21262275; doi: https://doi.org/10.1101/2021.08.19.21262275	
Bukhari SK, Asghar A, Perveen N, Hayat A, Mangat SA, Butt KR, Abdullah M, Fatima T, Mustafa A, Cheema T. Efficacy of ivermectin in COVID-19 patients with mild to moderate disease. medRxiv 2021.02.02.21250840; doi: https://doi.org/10.1101/2021.02.02.21250840	
Hsieh SM, Liu MC, Chen YH, Lee WS, Hwang SJ, Cheng SH, Ko WC, Hwang KP, Wang NC, Lee YL, Lin YL. Safety and immunogenicity of CpG 1018 and aluminium hydroxide-adjuvanted SARS-CoV-2 S-2P protein vaccine MVC-COV1901: a large-scale double-blind, randomised, placebo-controlled phase 2 trial. medRxiv 2021.08.05.21261532; doi: https://doi.org/10.1101/2021.08.05.21261532	Hsieh SM, Liu MC, Chen YH, Lee WS, Hwang SJ, Cheng SH, Ko WC, Hwang KP, Wang NC, Lee YL, Lin YL, Shih SR, Huang CG, Liao CC, Liang JJ, Chang CS, Chen C, Lien CE, Tai IC, Lin TY. Safety and immunogenicity of CpG 1018 and aluminium hydroxide-adjuvanted SARS-CoV-2 S-2P protein vaccine MVC-COV1901: interim results of a large-scale, double-blind, randomised, placebo-controlled phase 2 trial in Taiwan. Lancet Respir Med. 2021;9(12):1396-1406. doi: 10.1016/S2213-2600(21)00402-1.
Munch MW, Myatra SN, Vijayaraghavan BK, Saseedharan S, Benfield T, Wahlin RR, Rasmussen BS, Andreasen AS, Poulsen LM, Cioccari L, Khan MS. Dexamethasone 12 mg versus 6 mg for patients with COVID-19 and severe hypoxia: an international, randomized, blinded trial. medRxiv 2021.07.22.21260755; doi: https://doi.org/10.1101/2021.07.22.21260755	COVID STEROID 2 Trial Group, Munch MW, Myatra SN, Vijayaraghavan BKT, Saseedharan S, Benfield T, Wahlin RR, Rasmussen BS, Andreasen AS, Poulsen LM, Cioccari L, Khan MS, Kapadia F, Divatia JV, Brøchner AC, Bestle MH, Helleberg M, Michelsen J, Padmanaban A, Bose N, Møller A, Borawake K, Kristiansen KT, Shukla U, Chew MS, Dixit S, Ulrik CS, Amin PR, Chawla R, Wamberg CA, Shah MS, Darfelt IS, Jørgensen VL, Smitt M, Granholm A, Kjær MN, Møller MH, Meyhoff TS, Vesterlund GK, Hammond NE, Micallef S, Bassi A, John O, Jha A, Cronhjort M, Jakob SM, Gluud C, Lange T, Kadam V, Marcussen KV, Hollenberg J, Hedman A, Nielsen H, Schjørring OL, Jensen MQ, Leistner JW, Jonassen TB, Kristensen CM, Clapp EC, Hjortsø CJS, Jensen TS, Halstad LS, Bak ERB, Zaabalawi R, Metcalf-Clausen M, Abdi S, Hatley EV, Aksnes TS, Gleipner-Andersen E, Alarcón AF, Yamin G, Heymowski A,

© 2023 Bai AD et al. *JAMA Network Open*.

	Berggren A, La Cour K, Weihe S, Pind AH, Engstrøm J, Jha V, Venkatesh B, Perner A. Effect of 12 mg vs 6 mg of Dexamethasone on the Number of Days Alive Without Life Support in Adults With COVID-19 and Severe Hypoxemia: The COVID STEROID 2 Randomized Trial. JAMA. 2021;326(18):1807-1817. doi: 10.1001/jama.2021.18295.
Dorward J, Yu LM, Hayward G, Saville BR, Gbinigie O, Van Hecke O, Ogburn E, Evans PH, Thomas NP, Patel MG, Richards D. Colchicine for COVID-19 in adults in the community (PRINCIPLE): a randomised, controlled, adaptive platform trial. medRxiv 2021.09.20.21263828; doi: https://doi.org/10.1101/2021.09.20.21263828	Dorward J, Yu LM, Hayward G, Saville BR, Gbinigie O, Van Hecke O, Ogburn E, Evans PH, Thomas NP, Patel MG, Richards D, Berry N, Detry MA, Saunders C, Fitzgerald M, Harris V, Shanyinde M, de Lusignan S, Andersson MI, Butler CC, Hobbs FR; PRINCIPLE Trial Collaborative Group. Colchicine for COVID-19 in the community (PRINCIPLE): a randomised, controlled, adaptive platform trial. Br J Gen Pract. 2022:BJGP.2022.0083. doi: 10.3399/BJGP.2022.0083.
Yu LM, Bafadhel M, Dorward J, Hayward G, Saville BR, Gbinigie O, Van Hecke O, Ogburn E, Evans PH, Thomas NP, Patel MG. Inhaled budesonide for COVID-19 in people at higher risk of adverse outcomes in the community: interim analyses from the PRINCIPLE trial. medRxiv 2021.04.10.21254672; doi: https://doi.org/10.1101/2021.04.10.21254672	Yu LM, Bafadhel M, Dorward J, Hayward G, Saville BR, Gbinigie O, Van Hecke O, Ogburn E, Evans PH, Thomas NPB, Patel MG, Richards D, Berry N, Detry MA, Saunders C, Fitzgerald M, Harris V, Shanyinde M, de Lusignan S, Andersson MI, Barnes PJ, Russell REK, Nicolau DV Jr, Ramakrishnan S, Hobbs FDR, Butler CC; PRINCIPLE Trial Collaborative Group. Inhaled budesonide for COVID-19 in people at high risk of complications in the community in the UK (PRINCIPLE): a randomised, controlled, open-label, adaptive platform trial. Lancet. 2021;398(10303):843-855. doi: 10.1016/S0140-6736(21)01744-X.
REMAP-CAP Investigators. Interleukin-6 Receptor Antagonists in Critically Ill Patients with Covid-19–Preliminary report. medRxiv 2021.01.07.21249390; doi: https://doi.org/10.1101/2021.01.07.21249390	REMAP-CAP Investigators, Gordon AC, Mouncey PR, Al-Beidh F, Rowan KM, Nichol AD, Arabi YM, Annane D, Beane A, van Bentum-Puijk W, Berry LR, Bhimani Z, Bonten MJM, Bradbury CA, Brunkhorst FM, Buzgau A, Cheng AC, Detry MA, Duffy EJ, Estcourt LJ, Fitzgerald M, Goossens H, Haniffa R, Higgins AM, Hills TE, Horvat CM, Lamontagne F, Lawler PR, Leavis HL, Linstrum KM, Litton E, Lorenzi E, Marshall JC, Mayr FB, McAuley DF, McGlothlin A, McGuinness SP, McVerry BJ, Montgomery SK, Morpeth SC, Murthy S, Orr K, Parke RL, Parker JC, Patanwala AE, Pettilä V, Rademaker E, Santos MS, Saunders CT, Seymour CW, Shankar-Hari M, Sligl WI, Turgeon AF, Turner AM, van de Veerdonk FL, Zarychanski R, Green C, Lewis RJ, Angus DC, McArthur CJ, Berry S, Webb SA, Derde LPG. Interleukin-6 Receptor Antagonists in Critically Ill Patients with Covid-19. N Engl J Med. 2021;384(16):1491-1502. doi: 10.1056/NEJMoa2100433.
Derde LP, REMAP-CAP Investigators. Effectiveness of tocilizumab, sarilumab, and anakinra for critically ill patients with COVID-19 the REMAP-CAP COVID-19 immune modulation therapy domain randomized clinical trial. medRxiv 2021.06.18.21259133; doi: https://doi.org/10.1101/2021.06.18.21259133	
REMAP-CAP Investigators. Convalescent plasma in critically ill patients with Covid-19. medRxiv 2021.06.11.21258760; doi: https://doi.org/10.1101/2021.06.11.21258760	Writing Committee for the REMAP-CAP Investigators, Estcourt LJ, Turgeon AF, McQuilten ZK, McVerry BJ, Al-Beidh F, Annane D, Arabi YM, Arnold DM, Beane A, Bégin P, van Bentum-Puijk W, Berry LR, Bhimani Z, Birchall JE, Bonten MJM, Bradbury CA, Brunkhorst FM, Buxton M, Callum JL, Chassé M, Cheng AC, Cove ME, Daly J, Derde L, Detry MA, De Jong M, Evans A, Fergusson DA, Fish M, Fitzgerald M, Foley C, Goossens H, Gordon AC, Gosbell IB, Green C, Haniffa R, Harvala H, Higgins AM, Hills TE, Hoad VC, Horvat C, Huang DT, Hudson CL, Ichihara N, Laing E, Lamikanra AA, Lamontagne F, Lawler PR, Linstrum K, Litton E, Lorenzi E, MacLennan S, Marshall J, McAuley DF, McDyer JF, McGlothlin A, McGuinness S, Miflin G, Montgomery S, Mouncey PR, Murthy S, Nichol A, Parke R, Parker JC, Priddee N, Purcell DFJ, Reyes LF, Richardson P, Robitaille N, Rowan KM, Rynne J, Saito H, Santos M, Saunders CT, Serpa Neto A, Seymour CW, Silversides JA, Tinmouth AA, Triulzi DJ, Turner AM, van de Veerdonk F, Walsh TS, Wood EM, Berry S, Lewis RJ, Menon DK, McArthur C, Zarychanski R, Angus DC, Webb SA, Roberts DJ, Shankar-Hari M. Effect of Convalescent Plasma on Organ Support-Free Days in Critically Ill Patients With COVID-19: A Randomized Clinical Trial. JAMA. 2021;326(17):1690-1702. doi: 10.1001/jama.2021.18178.
Weinreich DM, Sivapalasingam S, Norton T, Ali S, Gao H, Bhore R, Hooper AT, Hamilton JD, Musser BJ, Soo Y, Rofail D. REGEN-COV antibody cocktail in outpatients with Covid-19. medRxiv 2021.06.09.21257915; doi: https://doi.org/10.1101/2021.06.09.21257915	

© 2023 Bai AD et al. *JAMA Network Open*.

Nguyen TP, Do Q, Phan LT, Dinh DV, Khong H, Hoang LV, Nguyen TV, Pham HN, Chu MV, Nguyen TT, Le TM. Safety and Immunogenicity of Nanocovax, a SARS-CoV-2 Recombinant Spike Protein Vaccine. medRxiv 2021.07.22.21260942; doi: https://doi.org/10.1101/2021.07.22.21260942	Nguyen TP, Do Q, Phan LT, Dinh DV, Khong H, Hoang LV, Nguyen TV, Pham HN, Chu MV, Nguyen TT, Pham QD, Le TM, Trang TNT, Dinh TT, Vo TV, Vu TT, Nguyen QBP, Phan VT, Nguyen LV, Nguyen GT, Tran PM, Nghiem TD, Tran TV, Nguyen TG, Tran TQ, Nguyen LT, Do AT, Nguyen DD, Ho SA, Nguyen VT, Pham DT, Tran HB, Vu ST, Hoang SX, Do TM, Nguyen XT, Le GQ, Tran T, Cao TM, Dao HM, Nguyen TTT, Doan UY, Le VTT, Tran LP, Nguyen NM, Nguyen NT, Pham HTT, Nguyen QH, Nguyen HT, Nguyen HLK, Tran VT, Tran MTN, Nguyen TTT, Ha PT, Huynh HT, Nguyen KD, Thuan UT, Doan CC, Do SM. Safety and immunogenicity of Nanocovax, a SARS-CoV-2 recombinant spike protein vaccine: Interim results of a double-blind, randomised controlled phase 1 and 2 trial. Lancet Reg Health West Pac. 2022 May 16;24:100474. doi: 10.1016/j.lanwpc.2022.100474.
Hinks TS, Cureton L, Knight R, Wang A, Cane JL, Barber VS, Black J, Dutton SJ, Melhorn J, Jabeen M, Moss P. A randomised clinical trial of azithromycin versus standard care in ambulatory COVID-19-the ATOMIC2 trial. medRxiv 2021.04.21.21255807; doi: https://doi.org/10.1101/2021.04.21.21255807	Hinks TSC, Cureton L, Knight R, Wang A, Cane JL, Barber VS, Black J, Dutton SJ, Melhorn J, Jabeen M, Moss P, Garlapati R, Baron T, Johnson G, Cantle F, Clarke D, Elkhodair S, Underwood J, Lasserson D, Pavord ID, Morgan S, Richards D. Azithromycin versus standard care in patients with mild-to-moderate COVID-19 (ATOMIC2): an open-label, randomised trial. Lancet Respir Med. 2021;9(10):1130-1140. doi: 10.1016/S2213-2600(21)00263-0.
Reis G, dos Santos Moreira-Silva EA, Silva DC, Thabane L, Milagres AC, Ferreira TS, Dos Santos CV, de Souza Campos VH, Nogueira AM, de Almeida AP, Callegari ED. Effect of early treatment with fluvoxamine on risk of emergency care and hospitalisation among patients with COVID-19: the TOGETHER randomised, platform clinical trial. medRxiv 2021.08.19.21262323; doi: https://doi.org/10.1101/2021.08.19.21262323	Reis G, dos Santos Moreira-Silva EA, Silva DC, Thabane L, Milagres AC, Ferreira TS, Dos Santos CV, de Souza Campos VH, Nogueira AM, de Almeida AP, Callegari ED. Effect of early treatment with fluvoxamine on risk of emergency care and hospitalisation among patients with COVID-19: the TOGETHER randomised, platform clinical trial. The Lancet Global Health. 2022;10(1):e42-51.
Quinn TM, Gaughan EE, Bruce A, Antonelli J, O'Connor R, Li F, McNamara S, Koch O, MacIntosh C, Dockrell D, Walsh T. Randomised Controlled Trial of Intravenous Nafamostat Mesylate in COVID pneumonitis: Phase 1b/2a Experimental Study to Investigate Safety, Pharmacokinetics and Pharmacodynamics. medRxiv 2021.10.06.21264648; doi: https://doi.org/10.1101/2021.10.06.21264648	Quinn TM, Gaughan EE, Bruce A, Antonelli J, O'Connor R, Li F, McNamara S, Koch O, MacKintosh C, Dockrell D, Walsh T, Blyth KG, Church C, Schwarze J, Boz C, Valanciute A, Burgess M, Emanuel P, Mills B, Rinaldi G, Hardisty G, Mills R, Findlay EG, Jabbal S, Duncan A, Plant S, Marshall ADL, Young I, Russell K, Scholefield E, Nimmo AF, Nazarov IB, Churchill GC, McCullagh JSO, Ebrahimi KH, Ferrett C, Templeton K, Rannard S, Owen A, Moore A, Finlayson K, Shankar-Hari M, Norrie J, Parker RA, Akram AR, Anthony DC, Dear JW, Hirani N, Dhaliwal K. Randomised controlled trial of intravenous nafamostat mesylate in COVID pneumonitis: Phase 1b/2a experimental study to investigate safety, Pharmacokinetics and Pharmacodynamics. EBioMedicine. 2022;76:103856. doi: 10.1016/j.ebiom.2022.103856.
Marconi VC, Ramanan AV, de Bono S, Kartman C, Krishnan V, Liao R, Piruzeli ML, Goldman JD, Alatorre-Alexander J, de Cassia Pellegrini R, Estrada V. Baricitinib plus standard of care for hospitalized adults with COVID-19. medRxiv 2021.04.30.21255934; doi: https://doi.org/10.1101/2021.04.30.21255934	Marconi VC, Ramanan AV, de Bono S, Kartman CE, Krishnan V, Liao R, Piruzeli MLB, Goldman JD, Alatorre-Alexander J, de Cassia Pellegrini R, Estrada V, Som M, Cardoso A, Chakladar S, Crowe B, Reis P, Zhang X, Adams DH, Ely EW; COV-BARRIER Study Group. Efficacy and safety of baricitinib for the treatment of hospitalised adults with COVID-19 (COV-BARRIER): a randomised, double-blind, parallel-group, placebo-controlled phase 3 trial. Lancet Respir Med. 2021;9(12):1407-1418. doi: 10.1016/S2213-2600(21)00331-3.
Shinde V, Bhikha S, Hossain Z, Archary M, Bhorat Q, Fairlie L, Lalloo U, Masilela ML, Moodley D, Hanley S, Fouche L. Preliminary Efficacy of the NVX-CoV2373 Covid-19 Vaccine Against the B. 1.351 Variant. medRxiv 2021.02.25.21252477; doi: https://doi.org/10.1101/2021.02.25.21252477	Shinde V, Bhikha S, Hoosain Z, Archary M, Bhorat Q, Fairlie L, Lalloo U, Masilela MSL, Moodley D, Hanley S, Fouche L, Louw C, Tameris M, Singh N, Goga A, Dheda K, Grobbelaar C, Kruger G, Carrim-Ganey N, Baillie V, de Oliveira T, Lombard Koen A, Lombaard JJ, Mngqibisa R, Bhorat AE, Benadé G, Lalloo N, Pitsi A, Vollgraaff PL, Luabeya A, Esmail A, Petrick FG, Oommen-Jose A, Foulkes S, Ahmed K, Thombrayil A, Fries L, Cloney-Clark S, Zhu M, Bennett C, Albert G, Faust E, Plested JS, Robertson A, Neal S, Cho I, Glenn GM, Dubovsky F, Madhi SA; 2019nCoV-501 Study Group. Efficacy of NVX-CoV2373 Covid-19 Vaccine against the B.1.351 Variant. N Engl J Med. 2021;384(20):1899-1909. doi: 10.1056/NEJMoa2103055.
Trieu V, Saund S, Rahate P, Barge V, Nalk S, Windlass H, Uckun F. Targeting TGF-b pathway with COVID-19 Drug Candidate ARTIVeda/PulmoHeal Accelerates Recovery from Mild-Moderate COVID-19. medRxiv 2021.01.24.21250418; doi: https://doi.org/10.1101/2021.01.24.21250418	Trieu V, Saund S, Rahate P, Barge V, Nalk S, Windlass H, Uckun F. Targeting TGF-β pathway with COVID-19 Drug Candidate ARTIVeda/PulmoHeal Accelerates Recovery from Mild-Moderate COVID-19. Clin Invest (Lond). 2021;11(1):10-18.

© 2023 Bai AD et al. *JAMA Network Open*.

Fischer WA, Eron JJ, Holman W, Cohen MS, Fang L, Szewczyk LJ, Sheahan TP, Baric RS, Mollan KR, Wolfe CR, Duke ER. Molnupiravir, an oral antiviral treatment for COVID-19. medRxiv 2021.06.17.21258639; doi: https://doi.org/10.1101/2021.06.17.21258639	Fischer WA 2nd, Eron JJ Jr, Holman W, Cohen MS, Fang L, Szewczyk LJ, Sheahan TP, Baric R, Mollan KR, Wolfe CR, Duke ER, Azizad MM, Borroto-Esoda K, Wohl DA, Coombs RW, James Loftis A, Alabanza P, Lipansky F, Painter WP. A phase 2a clinical trial of molnupiravir in patients with COVID-19 shows accelerated SARS-CoV-2 RNA clearance and elimination of infectious virus. Sci Transl Med. 2022;14(628):eabl7430. doi: 10.1126/scitranslmed.abl7430.
Feng Y, Chen J, Yao T, Chang Y, Li X, Xing R, Li H, Xie R, Zhang X, Wei Z, Mu S. Safety and Immunogenicity of Inactivated SARS-CoV-2 Vaccine in High-Risk Occupational Population: a randomized, parallel, controlled clinical trial. medRxiv 2021.08.06.21261696; doi: https://doi.org/10.1101/2021.08.06.21261696	Feng Y, Chen J, Yao T, Chang Y, Li X, Xing R, Li H, Xie R, Zhang X, Wei Z, Mu S, Liu L, Feng L, Wang S. Safety and immunogenicity of inactivated SARS-CoV-2 vaccine in high-risk occupational population: a randomized, parallel, controlled clinical trial. Infect Dis Poverty. 2021;10(1):138. doi: 10.1186/s40249-021-00924-2.
Temesgen Z, Burger CD, Baker J, Polk C, Libertin C, Kelley C, Marconi VC, Orenstein R, Durrant C, Chappell D, Ahmed O. LENZILUMAB EFFICACY AND SAFETY IN NEWLY HOSPITALIZED COVID-19 SUBJECTS: RESULTS FROM THE LIVE-AIR PHASE 3 RANDOMIZED DOUBLE-BLIND PLACEBO-CONTROLLED TRIAL. medRxiv 2021.05.01.21256470; doi: https://doi.org/10.1101/2021.05.01.21256470	Temesgen Z, Burger CD, Baker J, Polk C, Libertin CR, Kelley CF, Marconi VC, Orenstein R, Catterson VM, Aronstein WS, Durrant C, Chappell D, Ahmed O, Chappell G, Badley AD; LIVE-AIR Study Group. Lenzilumab in hospitalised patients with COVID-19 pneumonia (LIVE-AIR): a phase 3, randomised, placebo-controlled trial. Lancet Respir Med. 2022;10(3):237-246. doi: 10.1016/S2213-2600(21)00494-X.
Pan H, Wu Q, Zeng G, Yang J, Jiang D, Deng X, Chu K, Zheng W, Zhu F, Yu H, Yin W. Immunogenicity and safety of a third dose, and immune persistence of CoronaVac vaccine in healthy adults aged 18-59 years: interim results from a double-blind, randomized, placebo-controlled phase 2 clinical trial. MedRxiv. 2021.07.23.21261026; doi: https://doi.org/10.1101/2021.07.23.21261026	Zeng G, Wu Q, Pan H, Li M, Yang J, Wang L, Wu Z, Jiang D, Deng X, Chu K, Zheng W, Wang L, Lu W, Han B, Zhao Y, Zhu F, Yu H, Yin W. Immunogenicity and safety of a third dose of CoronaVac, and immune persistence of a two-dose schedule, in healthy adults: interim results from two single-centre, double-blind, randomised, placebo-controlled phase 2 clinical trials. Lancet Infect Dis. 2022;22(4):483-495. doi: 10.1016/S1473-3099(21)00681-2.

© 2023 Bai AD et al. *JAMA Network Open*.

eTable 4. Differences Between Preprints and Corresponding Journal Articles

	Differences in published journal article when compared to preprint				
Journal article	Outcomes (Primary and secondary)	Analyses	Sub-groups	Result	Conclusion
Abdulamir [1]	Same as preprint	Added Cox proportional hazard model	Same as preprint	Added Niclosamide had hazard ratio of 1.6 (p=0.007)	Niclosamide increased chance of cure and shortened hospital stay
Arruda [2]	Same as preprint	Same as preprint	Same as preprint	Same as preprint	Same as preprint
Baxter [3]	Symptom severity added as secondary outcome.	Changed analysis to binomial test with Clopper–Pearson confidence intervals.	Same as preprint	Added relative risk for nasal irrigation participants of .119 (95% CI: .0169–.833, P = .032).	Same as preprint
Bhatt [4]	Same as preprint	Same as preprint	Same as preprint	Same as preprint	Same as preprint
Gupta [5]	Omitted viral load and patient reported outcomes as secondary outcomes.	Same as preprint	Same as preprint	Same as preprint	Same as preprint
Gupta [6]	Same as preprint	Added absolute risk reduction	Same as preprint	Added absolute risk reduction of –4.53% (95% CI, –6.70% to –2.37%)	Same as preprint
Gupta [7]	Same as preprint	Same as preprint	Same as preprint	Same as preprint	Same as preprint
Biber [8]	Same as preprint	Same as preprint	Same as preprint	Adjusted odds ratio changed to at day 6 was 2.28 (95% CI: 0.87- 5.95, *P* = 0.09) at day 6 and 3.70 (95% CI: 1.19-11.49, *P* = 0.02) at day 8.	Same as preprint
Fisher [9]	Added following secondary outcomes: length of hospital stay, proportion of patients discharged at day 28, destination of discharge, body temperature, respiratory rate, lymphocyte count, neutrophil count, ferritin, d-dimers, lactate dehydrogenase.	Same as preprint	Same as preprint	Same as preprint	Same as preprint
Gaborit [10]	Same as preprint	Same as preprint	Same as preprint	Same as preprint	Same as preprint
Young [11]	Added following secondary outcomes: symptomatic and asymptomatic SARS-CoV-2 infections outside of first order contacts.	Same as preprint	Omitted subgroup analysis by student and staff	Same as preprint	Same as preprint
Clemency [12]	Omitted following secondary outcomes: oxygen saturation levels, COVID-19 viral load, and safety assessments.	Same as preprint	Added sub-group analysis of symptom severity.	Same as preprint	Same as preprint
Araujo [13]	Same as preprint	Same as preprint	Same as preprint	Same as preprint	Same as preprint
Tornero [14]	Same as preprint	Same as preprint	Same as preprint	Bar graph for change in CRP (Figure 4) is different in terms of absolute change and p-value.	Same as preprint
Hernandez-Cardenas [15]	Omitted secondary outcome of proportion of patients requiring	Same as preprint	Same as preprint	Same as preprint	Same as preprint

© 2023 Bai AD et al. *JAMA Network Open*.

	ventilatory support after admission.				
Mok [16]	Same as preprint	Same as preprint	Same as preprint	Same as preprint	Same as preprint
Portal-Celhay [17]	Same as preprint	Same as preprint	Same as preprint	Same as preprint	Same as preprint
Singh [18]	Same as preprint	Same as preprint	Same as preprint	Same as preprint	Same as preprint
Sullivan [19]	Omitted following secondary outcomes: ICU admission, invasive mechanical ventilation, death in hospital.	Added risk difference.	Added following sub0group analyses (Figure 3): BMI, age, duration of symptoms, CRP, lymphocyte count, co-existing conditions.	Added absolute risk reduction of 3.4% 95% CI 1.0 to 5.8; P = 0.005.	Same as preprint
Huang [20]	Same as preprint	Same as preprint	Same as preprint	Same as preprint	Same as preprint
Weinreich [21]	Same as preprint	Same as preprint	Same as preprint	Same as preprint	Same as preprint
Parikh [22]	Same as preprint	Same as preprint	Same as preprint	Same as preprint	Same as preprint
Ely [23]	Same as preprint	Same as preprint	Omitted subgroup analysis of baseline remdesivir in supplementary materials.	Same as preprint	Same as preprint
Fedrizzi [24]	Same as preprint	Same as preprint	Same as preprint	Same as preprint	Same as preprint
Kolev [25]	Same as preprint	Same as preprint	Same as preprint	Same as preprint	Same as preprint
Breza [26]	Primary outcome changed to Aggregate holiday travel measured using mobile phone location data, **fraction who left home** and COVID-19 infection at zip-code level.	Same as preprint	Same as preprint	Added fraction of people who left home on the holiday was not significantly affected, adjusted difference 0.030, 95% CI -0.361 to 0.420, p=0.881.	Same as preprint
Lattman [27]	Primary outcome changed to Mean change in 8 point WHO ordinal scale by day **15** and 28-day mortality.<br>Omitted secondary outcome of change in inflammation markers IL6 and CRP from baseline to Day 14,	Same as preprint	Same as preprint	Same as preprint	Same as preprint
Gaughan [28]	Same as preprint	Same as preprint	Same as preprint	Same as preprint	Same as preprint
McCreary [29]	Same as preprint	Same as preprint	Same as preprint	Same as preprint	Same as preprint
Kyriazopoulou [30]	Same as preprint	Same as preprint	Same as preprint	Same as preprint	Same as preprint
Mikhaylov [31]	Primary outcome changed to time frame of 8 weeks	Same as preprint	Same as preprint	Same as preprint	Same as preprint
REMAP-CAP, ACTIV-4a, and ATTACC Investigators [32]	Added secondary outcome of death alone.	Same as preprint	Same as preprint	Sample size changed to 536 in therapeutic anticoagulation arm and 567 in thromboprophylaxis arm. The result was changed to median value for organ support–free days was 1 (interquartile range, –1 to 16); among the patients assigned to usual-care pharmacologic. Odds ratio was 0.83 (95% credible interval, 0.67 to 1.03), yielding a posterior probability of futility	Same as preprint

© 2023 Bai AD et al. *JAMA Network Open*.

				of 99.9% and a posterior probability of inferiority of 95.0% thromboprophylaxis.	
Syed [33]	Combined secondary outcome of drug discontinuation due to side effects and other reasons into a single secondary outcome.	Same as preprint	Same as preprint	Same as preprint	Same as preprint
Cadegiani [34]	Same as preprint	Same as preprint	Subgroup analysis of cities was changed to north versus south.	Sample size changed to 424 in proxalutamide arm and 355 in placebo arm as added 3 sites. Results changed to recovery occurring in 343 (81.1%) in proxalutamide arm and 130 (36.6%) in placebo arm with rate ratio of 2.21 (95% CI 1.92 to 2.56).	Added that Proxalutamide shortened hospital stay in hospitalized COVID-19 patients.
Ader [35]	Same as preprint	Same as preprint	Same as preprint	Same as preprint	Same as preprint
Fralick [36]	Omitted secondary outcome of time to recovery defined as being on room air for at least 24 hours in the journal article, but this was never reported in the preprint.	Same as preprint	Same as preprint	Same as preprint	Same as preprint
Hernandez-Bernal [37]	Same as preprint	Calculated the difference in proportion of seroconversion for RBD-IgG at day 56 compared to control.	Same as preprint	Added differences in the proportion of participants with seroconversion compared to placebo, which were 73.1% (95% CI 66.8-79.5) and 84.6% (79.4-89.7) in the 25 mg and 50 mg groups, respectively. The seroconversion rate in the 50 mg group was significantly higher than in the 25 mg group (p=0.0012).	Same as preprint
Lescure [38]	Same as preprint	Same as preprint	Added 15 additional subgroup analyses (Figure S2).	Same as preprint	Same as preprint
Perkins [39]	Same as preprint	Added risk difference for analysis.	Added 6 sub-group analyses (Figure 2 and 3).	Sample size changed to 418 in high flow nasal oxygenation group. Thus, Composite outcome occurred in 44.3% (184/415) of the participants in the HFNO group vs 45.1%(166/368) of the participants in the conventional oxygen therapy group (absolute difference, −1% [95%CI, −8% to 6%], P = .83).	Same as preprint
Tornling [40]	Primary outcome was changed to CRP from baseline to end of treatment **where mean of last 2 assessments were used**.	Added analysis of ratio of adjusted treatment means by intention to treat principle.	Same as preprint	Results added ratio of adjusted treatment means 0.85; 90% CI 0.57, 1.26)	Same as preprint

© 2023 Bai AD et al. *JAMA Network Open*.

Resended [41]	Same as preprint	Analysis changed from Mann Whitney U test to Student's t-test.	Same as preprint	Same as preprint	Same as preprint
Dai [42]	Same as preprint	Same as preprint	Same as preprint	Sample size changed for each group for the first and second trial, leading to small changes in the point estimates.	Same as preprint
Gaitan-Duarte [43]	Same as preprint	Analysis changed to Cox proportional hazards model.	Same as preprint	Added hazard ratio for 28-day mortality, which was 0.53 95% CI 0.29 to 0.96 for FTC/TDF + COLCH + ROSUV group when compared to SOC group. The HR was 0.68 95% CI 0.39 to 1.20 for FTC/TDF group and 0.78 95% CI 0.44 to 1.36 for COLCH+ROSUV group.	Conclusion changed to positive result that the FTC/TDF+COLCH+ROSUV combination is associated with a reduced risk of dying within the first 28 days.
Arnardottir [44]	Same as preprint	Analysis changed to Wilcoxon rank sum test.	Same as preprint	P-value not specified in figures, but presumed to be different given different statistical method was used.	Same as preprint
Pan [45]	Same as preprint	Same as preprint	Same as preprint	Same as preprint	Same as preprint
Almanza-Reyes [46]	Same as preprint	Same as preprint	Same as preprint	Same as preprint	Same as preprint
Tardif [47]	Same as preprint	Same as preprint	Added subgroup analysis of race.	Same as preprint	Conclusion changed to acknowledge that it is a negative trial and that effect of colchicine on COVID-19 related clinical events was not statistically significant.
Rossignol [48]	Omitted following secondary outcomes: proportion of subjects positive for SARS-CoV-2 by days 4 and 10, change from baseline in quantitative SARS-CoV-2 RNA by days 4 and 10, proportion of subjects hospitalized due to COVID-19 or COVID-19 complications, and proportion of subjects with mortality due to COVID-19 or COVID-19 complications.	Same as preprint	Same as preprint	Same as preprint	Conclusion changed to acknowledge that it is a negative trial and that there was no difference in primary outcome between nitazoxanide and placebo.
ACTIV-3/TICO Bamlanivimab Study Group [49]	Same as preprint	Analysis changed to use the Aalen-Johansen method to estimate the cumulative incidence.	Omitted following subgroup analyses: antigen and viral RNA, antigen and viral RNA by nAb sub-groups.	The relative risk reduction between bamlanivimab and placebo for when nasal wab viral RNA was above the median entry level was changed to 1.89 CI 1.23 to 2.91.	Same as preprint
Liu [50]	Same as preprint	Same as preprint	Same as preprint	Same as preprint	Same as preprint
Li [51]	Same as preprint	Analysis added non-inferiority definition of	Same as preprint	Added p-value of GMT, which was P<0.0001 for superiority	Same as preprint

© 2023 Bai AD et al. *JAMA Network Open*.

		when thee lower 95% CI of GMT ratio exceeded 0.67.		when compared to homologous group.	
Rojas-Serrano [52]	Same as preprint	Same as preprint	Same as preprint	P-value for log rank test changed to 0.07.	Same as preprint
Beltran-Gonzalez [53]	Primary outcome changed to include proportion of respiratory deterioration, and death.	Same as preprint	Same as preprint	Added proportion of respiratory deterioration and death, which occurred in 6 (18.1%) in hydroxychloroquine, 8 (22.2%) in ivermectin and 9 (24.3%) in placebo (p=0.83).	Same as preprint
Figueroa [54]	Omitted secondary outcome of days off work.	Added absolute risk reduction.	Same as preprint	Relative risk reduction was changed to 79.8% 95% CI 5.3% to 95.4% P=0.03. Absolute risk reduction was Absolute risk reduction: 4% 95% CI 0.6% to 7.4%.	Same as preprint
Winthrop [55]	Same as preprint	Same as preprint	Same as preprint	Same as preprint	Same as preprint
Wanaratna [56]	Primary outcome changed to complete clinical recovery by day 5. The primary outcome in the preprint of pneumonia was changed to a secondary outcome.	Analysis changed to Chi-square or Fisher's exact test.	Same as preprint	Based on new primary outcome, no one recovered completely by day 5 in the trial.	Authors changed conclusion to no efficacy based on no complete recovery by day 5 in either group.
Damle [57]	Added following for secondary outcomes: escalation of care, development of fever.	Not described.	Same as preprint	Same as preprint	Same as preprint
Dunkle [58]	Same as preprint	Same as preprint	Same as preprint	Same as preprint	Same as preprint
Maskin [59]	Same as preprint	Same as preprint	Same as preprint	Same as preprint	Same as preprint
Silveira [60]	Same as preprint	Same as preprint	Same as preprint	Same as preprint	Same as preprint
Eugenia-Toledo-Romaní [61]	Same as preprint	Same as preprint	Same as preprint	Same as preprint	Same as preprint
Tsilika [62]	Same as preprint	Same as preprint	Same as preprint	Same as preprint	Same as preprint
Holubar [63]	Same as preprint	Same as preprint	Same as preprint	Same as preprint	Same as preprint
O'Donnell [64]	Same as preprint	Same as preprint	Same as preprint	Same as preprint	Same as preprint
O'Brien [65]	Same as preprint	Same as preprint	Added sub-group analyses for age of at least 50 years, at least 65 years, patients at high risk for progression to severe disease.	Same as preprint	Same as preprint
O'Brien [66]	Added following secondary outcomes: only symptomatic infections that began 3 days or longer after treatment; symptomatic infections in participants with risk factors for progression to severe COVID-19; and the number of participants with detectable virus at each week during the efficacy assessment period.	Analysis was described as logistic regression model, which was not described in the preprint.	Added following subgroup analyses (Table e3): age, race & ethnicity, gender, BMI, region, household size, risk factor, healthcare worker, high viral load.	Added absolute risk difference, which was -13.3% (95% CI -26.3% to -0.3%) for REGEV-COV group compared to placebo.	Same as preprint

© 2023 Bai AD et al. *JAMA Network Open*.

Puskarich [67]	Same as preprint	Added secondary analyses that excluded early death.	Same as preprint	Excluding early death yielded estimated effect of −27.1 (95%CI, −57.8 to 3.7; P = .08), while a complete case analysis yielded −23.6 (95% CI −55.6 to 8.5; P = .15).	Same as preprint
Bonelli [68]	Same as preprint	Same as preprint	Same as preprint	Same as preprint	Same as preprint
Sholzberg [69]	Same as preprint	Same as preprint	Same as preprint	Same as preprint	Same as preprint
Zeng [70]	Added primary outcome of geometric mean increases.	Added analysis between group by t-test with log-transformation and Bonferroni correction done as a post-hoc test if variance was significant. Added generalised linear mixed models to compare antibody concentrations.	Omitted age subgroup analyses.	New results showed that immune responses induced by 6 µg doses were better than those induced by 3 µg doses, and a third dose significantly raised antibody levels compare with 28 days after dose 2.	Conclusion emphasized third dose had a remarkable immune response as booster.
Elgohary [71]	Same as preprint	Same as preprint	Same as preprint	Same as preprint	Same as preprint
Formica [72]	Same as preprint	Same as preprint	Same as preprint	Same as preprint	Same as preprint
Morici [73]	Same as preprint	Same as preprint	Same as preprint	Same as preprint	Same as preprint
Babalola [74]	Same as preprint	Same as preprint	Same as preprint	Same as preprint	Same as preprint
ATTACC, ACTIV-4a, REMAP-CAP Investigators [75]	Same as preprint	Omitted sensitivity analysis permitting dynamic borrowing of information on treatment effect from severe participants (see Table S4)	Same as preprint	Change of results in terms of probability that therapeutic anticoagulation increased organ support-free days was 98.6% (adjusted odds ratio 1.27, 95% credible interval 1.03 to 1.58). The adjusted absolute increase in survival to hospital discharge without organ support with therapeutic-dose anticoagulation was 4.0% (95% credible interval 0.5 to 7.2).	Same as preprint
Goepfert [76]	Same as preprint	Same as preprint	Same as preprint	Same as preprint	Same as preprint
Gutierrez-Castrellon [77]	Same as preprint	Omitted analysis for absolute risk reduction and odds ratio. Reported relative risk.	Same as preprint	The relative risk for complete remission was 1.89 (95% CI 1.40 to 2.55 P<0.001) for probiotic group compared to placebo.	Same as preprint
RECOVERY Collaborative Group [78]	Same as preprint	Same as preprint	Same as preprint	Same as preprint	Same as preprint
RECOVERY Collaborative Group [79]	Same as preprint	Same as preprint	Same as preprint	Mortality was changed to **1399**/5795 (24%) in convalescent plasma group and 1408/5763 (24%) in usual care group died (RR 1.0 95% CI 0.93 to 1.07 **P=0.93**)	Same as preprint

© 2023 Bai AD et al. *JAMA Network Open*.

RECOVERY Collaborative Group [80]	Same as preprint	Same as preprint	Same as preprint	Mortality was changed to **943**/4839 (20%) treated with REGEN-COV and **1029**/4946 (21%) treated with usual care die (RR 0.94 95% CI 0.86 to **1.02** **P=0.14**). In seronegative patients, 396/1633 (24%) treated with REGEN-COV and **452**/1520 (30%) treated with usual care died (**RR 0.79 95% CI 0.69 to 0.91** **P=0.009**).	Same as preprint
RECOVERY Collaborative Group [81]	Same as preprint	Same as preprint	Same as preprint	Same as preprint	Same as preprint
RECOVERY Collaborative Group [82]	Same as preprint	Same as preprint	Same as preprint	Mortality was changed to **621/2022 (31%)** in tocilizumab group and **729/2094 (33%)** in usual care group die (**RR 0.85 95% CI 0.76 to 0.94 P=0.0028)**.	Same as preprint
Begin [83]	Same as preprint	Same as preprint	Same as preprint	Same as preprint	Same as preprint
Mallory [84]	Same as preprint	Same as preprint	Same as preprint	Same as preprint	Same as preprint
Ella [85]	Same as preprint	Same as preprint	Same as preprint	Same as preprint	Same as preprint
Lazarus [86]	Secondary outcome of adverse events extended to 106 days.	Same as preprint	Same as preprint	Same as preprint	Same as preprint
Ravikirti [87]	Same as preprint	Analysis changed from Fisher's exact test to Chi-square test.	Same as preprint	Same as preprint	Same as preprint
Ravichandran [88]	Same as preprint	Analysis changed to Chi-square under intent-to-treat principle.	Same as preprint	Hypoxia occurred in 0/102 (0%) in the indomethacin group and 20/108 (19%) in the standard of care group **(P<0.01).**	Same as preprint
Elzein [89]	Same as preprint	Changed analyses to Kruskal-Wallis test followed by Dunn's multiple comparison post-hoc test plus paired T-test.	Same as preprint	Same as preprint	Same as preprint
Sablerolles [90]	Same as preprint	Analysis changed to Mann-Whitney U and quantile regression.	Same as preprint	Results changed to the following: Antibody level was higher in homologous Ad26.COV2.S booster than 1 short regimen (beta coefficient, 0.64; 98.3% CI, 0.41 to 0.81; P<0.001). Antibody level was higher for BNT162b2 booster regimen (beta coefficient, 0.73; 98.3% CI, 0.57 to 0.90; P<0.001) and	Same as preprint

© 2023 Bai AD et al. *JAMA Network Open*.

				mRNA-1273 booster regimen (beta coefficient, 0.94; 98.3% CI, 0.85 to 1.12; P<0.001). mRNA-1273 booster increased binding antibodies to higher levels than the BNT162b2 booster (beta coefficient, 0.21; 98.3% CI, 0.13 to 0.37).	
Chahla [91]	Omitted secondary outcome of symptoms.	Same as preprint	Same as preprint	Same as preprint	Same as preprint
Izikson [92]	Same as preprint	Same as preprint	Same as preprint	Same as preprint	Same as preprint
Ramakrishnan [93]	Same as preprint	Same as preprint	Same as preprint	Same as preprint	Same as preprint
Sridhar [94]	Same as preprint	Same as preprint	Added following subgroup analyses: age group, high risk medical conditions and baseline SARS-CoV-2 naïve status.	Same as preprint	Same as preprint
Somersan-Karakaya [95]	Same as preprint	Same as preprint	Same as preprint	Same as preprint	Same as preprint
Heath [96]	Same as preprint	Analysis changed to be under per-protocol and intent-to-treat principle.	Same as preprint	Added results for intent-to-treat population, where COVID occurred in 141/7570 in placebo group and 42/7569 in vaccine group that had COVID-19 with vaccine efficacy of 70.4% (95% CI 58.3% to 79.1%).	Same as preprint
Madhi [97]	Same as preprint	Same as preprint	Same as preprint	Same as preprint	Same as preprint
Shoham [98]	Same as preprint	Same as preprint	Same as preprint	Same as preprint	Same as preprint
Mendez-Flores [99]	Same as preprint	Same as preprint	Same as preprint	Same as preprint	Same as preprint
Korper [100]	Same as preprint	Same as preprint	Same as preprint	Same as preprint	Same as preprint
Thomas [101]	Same as preprint	Same as preprint	Same as preprint	Same as preprint	Same as preprint
Sivapalasingam [102]	Same as preprint	Same as preprint	Same as preprint	Same as preprint	Same as preprint
Hsieh [103]	Added following secondary outcomes: lot-to-lot consistency of MVC-COV1901 in patients aged 20 years to younger than 65 years, and the immunogenicity of MVC-COV1901 compared with placebo, in terms of antigen-specific IgG titres and neutralising antibody titres.	Same as preprint	Same as preprint	Same as preprint	Same as preprint
COVID STEROID 2 Trial Group [104]	Added following secondary outcomes: number of days alive without life support at 90 days, the number of days alive out of the hospital at 90 days, and mortality at 90 days.	Same as preprint	Same as preprint	Same as preprint	Same as preprint
Dorward [105]	Same as preprint	Same as preprint	Same as preprint	Same as preprint	Same as preprint

© 2023 Bai AD et al. *JAMA Network Open*.

Yu [106]	Added secondary outcome of reports of new household infections.	Same as preprint	Added following sub-group analyses: COPD, asthma or lung disease, and vaccination	Sample size increased to 833 in budesonide group and 1126 in usual care group. The hazard ratio was changed to 1.21 (95% credible interval 1.08 to 1.36) with median benefit of 2.94 (95% credible interval 1.19 to 5.11 days), probability of superiority of 0.999. Hospitalization/deaths occurred in 72/787 (9%) in the budesonide group and 116/1069 (11%) in usual care with estimated percentage benefit of 2.0% (95% credible level -0.2% to 4.5%) and probability of superiority of 0.963.	Same as preprint
REMAP-CAP investigators [107]	Same as preprint	Same as preprint	Added sub-group analysis of mechanical ventilation or not at baseline.	Same as preprint	Same as preprint
REMAP-CAP investigators [108]	Added following secondary outcomes: respiratory support–free days; cardiovascular support–free days; venous thromboembolic events at 90 days; and serious adverse events.	Same as preprint	Same as preprint	Median adjusted odds ratio was changed to 0.97 **(95% CrI 0.82 to 1.14)** with a posterior probability of futility was **99.5%**.	Same as preprint
Nguyen [109]	Same as preprint	Same as preprint	Same as preprint	Same as preprint	Same as preprint
Hinks [110]	Same as preprint	Added risk difference calculation.	Added subgroup analysis for hypertension, diabetes, age and sex in S4.5. Note that the preprint supplementary materials was damaged and could not be opened.	Same as preprint<br>The fully adjusted risk difference in the ITT population was -1.2% (-8.2% to 5.7%).	Same as preprint
Reis [111]	Same as preprint	Same as preprint	Added following subgroup analyses: chronic kidney disease and smoking status. Omitted following subgroup analyses: lung disease and diabetes mellitus.	Sample size increased to 741 in fluvoxamine group and 756 in placebo group. RR was changed to **0.68 (95% CrI 0.52 to 0.88)** with probability of superiority of **99.8%.**	Same as preprint
Quinn [112]	Same as preprint	Same as preprint	Same as preprint	Adverse event was changed to 86% in nafamostat group. Posterior mean odds ratio was changed to 5.17 (95% CrI 1.10 to 26.05).	Same as preprint
Marconi [113]	Same as preprint	Same as preprint	Same as preprint	Same as preprint	Same as preprint
Shinde [114]	Same as preprint	Same as preprint	Same as preprint	Same as preprint	Same as preprint
Trieu [115]	Same as preprint	Same as preprint	Same as preprint	Same as preprint	Same as preprint
Fischer [116]	Same as preprint	Same as preprint	Same as preprint	Same as preprint	Same as preprint

© 2023 Bai AD et al. *JAMA Network Open*.

Feng [117]	Removed GMT of neutralizing antibody to live SARS-CoV-2, 28 days after the whole course of vaccination from secondary outcome.	Same as preprint	Same as preprint	Same as preprint	Same as preprint
Temesgen [118]	Added following secondary outcomes: ventilator-free days; duration of ICU stay as well as 15 other secondary outcomes.	Added sensitivity analysis that included covariate from comorbidities and baseline CRP.	Omitted following subgroup analyses: CRP<150mg/L plus age <85, hospitalization prior to randomization.	Likelihood of ventilator free survival hazard ratio for lenzilumab was changed to 1.54 (95% CI 1.02 to **2.32**; **P=0.040**).	Same as preprint
Zeng [119]	Added primary outcome of geometric mean increases. Omitted any adverse events from secondary outcome.	Added analysis between group by t-test with log-transformation and Bonferroni correction done as a post-hoc test if variance was significant. Added generalised linear mixed models to compare antibody concentrations.	Same as preprint	Safety population changed from total of 600 to 540. New results showed that immune responses induced by 6 μg doses were better than those induced by 3 μg doses, and a third dose significantly raised antibody levels compare with 28 days after dose 2.	Conclusion emphasized third dose had a remarkable immune response as booster.

© 2023 Bai AD et al. *JAMA Network Open*.

eReferences 2

1. Abdulamir AS, Gorial FI, Saadi SJ, Maulood MF, Hashim HA, Alnuaimi AS, Abdulrrazaq MK. A randomised controlled trial of effectiveness and safety of Niclosamide as add on therapy to the standard of care measures in COVID-19 management. Ann Med Surg (Lond). 2021 Sep;69:102779. doi: 10.1016/j.amsu.2021.102779.
2. Arruda EA, Pires-Neto RJ, Medeiros MS, Quirino-Filho J, Clementino M, Gondim RN, Magalhães LM, Cavalcante KF, Viana VA, Mello LP, Lima DG. Clinical Features, Pathobiology, Efficacy, and Toxicity of Tenofovir Disoproxil Fumarate and Emtricitabine for Mild to Moderate SARS-CoV-2 Infections. Eur J Respir Med. 2021;3(3):238-48.
3. Baxter AL, Schwartz KR, Johnson RW, Kuchinski AM, Swartout KM, Srinivasa Rao ASR, Gibson RW, Cherian E, Giller T, Boomer H, Lyon M, Schwartz R. Rapid initiation of nasal saline irrigation to reduce severity in high-risk COVID+ outpatients. Ear Nose Throat J. 2022 Aug 25:1455613221123737. doi: 10.1177/01455613221123737.
4. Bhatt AN, Shenoy S, Munjal S, Chinnadurai V, Agarwal A, Vinoth Kumar A, Shanavas A, Kanwar R, Chandna S. 2-deoxy-D-glucose as an adjunct to standard of care in the medical management of COVID-19: a proof-of-concept and dose-ranging randomised phase II clinical trial. BMC Infect Dis. 2022 Aug 4;22(1):669. doi: 10.1186/s12879-022-07642-6.
5. Gupta A, Gonzalez-Rojas Y, Juarez E, Crespo Casal M, Moya J, Falci DR, Sarkis E, Solis J, Zheng H, Scott N, Cathcart AL, Hebner CM, Sager J, Mogalian E, Tipple C, Peppercorn A, Alexander E, Pang PS, Free A, Brinson C, Aldinger M, Shapiro AE; COMET-ICE Investigators. Early Treatment for Covid-19 with SARS-CoV-2 Neutralizing Antibody Sotrovimab. N Engl J Med. 2021 Nov 18;385(21):1941-1950.
6. Gupta A, Gonzalez-Rojas Y, Juarez E, Crespo Casal M, Moya J, Rodrigues Falci D, Sarkis E, Solis J, Zheng H, Scott N, Cathcart AL, Parra S, Sager JE, Austin D, Peppercorn A, Alexander E, Yeh WW, Brinson C, Aldinger M, Shapiro AE; COMET-ICE Investigators. Effect of Sotrovimab on Hospitalization or Death Among High-risk Patients With Mild to Moderate COVID-19: A Randomized Clinical Trial. JAMA. 2022 Apr 5;327(13):1236-1246.
7. Gupta A, Madan A, Yadav B, Mundada P, Singhal R, Tripathi A, Rao BC, Gupta B, Rana R, Sharma B, Pandey Y. A randomized controlled trial to evaluate the prophylactic efficacy of Chyawanprash in healthcare workers during the COVID-19 pandemic. Journal of Research in Ayurvedic Sciences. 2021; 5(1): 13-25.
8. Biber A, Harmelin G, Lev D, Ram L, Shaham A, Nemet I, Kliker L, Erster O, Mandelboim M, Schwartz E. The effect of ivermectin on the viral load and culture viability in early treatment of nonhospitalized patients with mild COVID-19 - a double-blind, randomized placebo-controlled trial. Int J Infect Dis. 2022 Sep;122:733-740. doi: 10.1016/j.ijid.2022.07.003.
9. Fisher BA, Veenith T, Slade D, Gaskell C, Rowland M, Whitehouse T, Scriven J, Parekh D, Balasubramaniam MS, Cooke G, Morley N, Gabriel Z, Wise MP, Porter J, McShane H, Ho LP, Newsome PN, Rowe A, Sharpe R, Thickett DR, Bion J, Gates S, Richards D, Kearns P; CATALYST investigators. Namilumab or infliximab compared with standard of care in hospitalised patients with COVID-19 (CATALYST): a randomised, multicentre, multi-arm, multistage, open-label, adaptive, phase 2, proof-of-concept trial. Lancet Respir Med. 2022 Mar;10(3):255-266.
10. Gaborit B, Dailly E, Vanhove B, Josien R, Lacombe K, Dubee V, Ferre V, Brouard S, Ader F, Vibet MA, Le Thuaut A, Danger R, Flet L, Omnes A, Berly L, Chiffoleau A, Jobert A, Duvaux O, Raffi F; POLYCOR Trial Group. Pharmacokinetics and Safety of XAV-19, a Swine Glyco-humanized Polyclonal Anti-SARS-CoV-2 Antibody, for COVID-19-Related Moderate Pneumonia: a Randomized, Double-Blind, Placebo-Controlled, Phase IIa Study. Antimicrob Agents Chemother. 2021 Aug 17;65(9):e0123721. doi: 10.1128/AAC.01237-21.
11. Young BC, Eyre DW, Kendrick S, White C, Smith S, Beveridge G, Nonnenmacher T, Ichofu F, Hillier J, Oakley S, Diamond I, Rourke E, Dawe F, Day I, Davies L, Staite P, Lacey A, McCrae J, Jones F, Kelly J, Bankiewicz U, Tunkel S, Ovens R, Chapman D, Bhalla V, Marks P, Hicks N, Fowler T,

© 2023 Bai AD et al. *JAMA Network Open*.

Hopkins S, Yardley L, Peto TEA. Daily testing for contacts of individuals with SARS-CoV-2 infection and attendance and SARS-CoV-2 transmission in English secondary schools and colleges: an open-label, cluster-randomised trial. Lancet. 2021 Oct 2;398(10307):1217-1229. doi: 10.1016/S0140-6736(21)01908-5.

12. Clemency BM, Varughese R, Gonzalez-Rojas Y, Morse CG, Phipatanakul W, Koster DJ, Blaiss MS. Efficacy of Inhaled Ciclesonide for Outpatient Treatment of Adolescents and Adults With Symptomatic COVID-19: A Randomized Clinical Trial. JAMA Intern Med. 2022 Jan 1;182(1):42-49. doi: 10.1001/jamainternmed.2021.6759.
13. Araujo CSR, Medeiros-Ribeiro AC, Saad CGS, Bonfiglioli KR, Domiciano DS, Shimabuco AY, Silva MSR, Yuki EFN, Pasoto SG, Pedrosa T, Kupa LVK, Zou G, Pereira RMR, Silva CA, Aikawa NE, Bonfa E. Two-week methotrexate discontinuation in patients with rheumatoid arthritis vaccinated with inactivated SARS-CoV-2 vaccine: a randomised clinical trial. Ann Rheum Dis. 2022 Jun;81(6):889-897. doi: 10.1136/annrheumdis-2021-221916.
14. Tornero C, Pastor E, Garzando MDM, Orduña J, Forner MJ, Bocigas I, Cedeño DL, Vallejo R, McClure CK, Czura CJ, Liebler EJ, Staats P. Non-invasive Vagus Nerve Stimulation for COVID-19: Results From a Randomized Controlled Trial (SAVIOR I). Front Neurol. 2022 Apr 8;13:820864. doi: 10.3389/fneur.2022.820864.
15. Hernandez-Cardenas C, Thirion-Romero I, Rodríguez-Llamazares S, Rivera-Martinez NE, Meza-Meneses P, Remigio-Luna A, Perez-Padilla R; Research Group on hydroxychloroquine for COVID-19. Hydroxychloroquine for the treatment of severe respiratory infection by COVID-19: A randomized controlled trial. PLoS One. 2021 Sep 28;16(9):e0257238. doi: 10.1371/journal.pone.0257238.
16. Mok CKP, Chen C, Yiu K, Chan TO, Lai KC, Ling KC, Sun Y, Hui DS, Cheng SMS, Peiris M. A Randomized Clinical Trial Using CoronaVac or BNT162b2 Vaccine as a Third Dose in Adults Vaccinated with Two Doses of CoronaVac. Am J Respir Crit Care Med. 2022 Apr 1;205(7):844-847. doi: 10.1164/rccm.202111-2655LE.
17. Portal-Celhay C, Forleo-Neto E, Eagan W, Musser BJ, Davis JD, Turner KC, Norton T, Hooper AT, Hamilton JD, Pan C, Mahmood A, Baum A, Kyratsous CA, Kim Y, Parrino J, Kampman W, Roque-Guerrero L, Stoici R, Fatakia A, Soo Y, Geba GP, Kowal B, DiCioccio AT, Stahl N, Lipsich L, Braunstein N, Herman GA, Yancopoulos GD, Weinreich DM; COVID-19 Phase 2 Dose-Ranging Study Team. Virologic Efficacy of Casirivimab and Imdevimab COVID-19 Antibody Combination in Outpatients With SARS-CoV-2 Infection: A Phase 2 Dose-Ranging Randomized Clinical Trial. JAMA Netw Open. 2022 Aug 1;5(8):e2225411. doi: 10.1001/jamanetworkopen.2022.25411.
18. Singh D, Bogus M, Moskalenko V, Lord R, Moran EJ, Crater GD, Bourdet DL, Pfeifer ND, Woo J, Kaufman E, Lombardi DA, Weng EY, Nguyen T, Woodcock A, Haumann B, Saggar R. A phase 2 multiple ascending dose study of the inhaled pan-JAK inhibitor nezulcitinib (TD-0903) in severe COVID-19. Eur Respir J. 2021 Oct 14;58(4):2100673.
19. Sullivan DJ, Gebo KA, Shoham S, Bloch EM, Lau B, Shenoy AG, Mosnaim GS, Gniadek TJ, Fukuta Y, Patel B, Heath SL, Levine AC, Meisenberg BR, Spivak ES, Anjan S, Huaman MA, Blair JE, Currier JS, Paxton JH, Gerber JM, Petrini JR, Broderick PB, Rausch W, Cordisco ME, Hammel J, Greenblatt B, Cluzet VC, Cruser D, Oei K, Abinante M, Hammitt LL, Sutcliffe CG, Forthal DN, Zand MS, Cachay ER, Raval JS, Kassaye SG, Foster EC, Roth M, Marshall CE, Yarava A, Lane K, McBee NA, Gawad AL, Karlen N, Singh A, Ford DE, Jabs DA, Appel LJ, Shade DM, Ehrhardt S, Baksh SN, Laeyendecker O, Pekosz A, Klein SL, Casadevall A, Tobian AAR, Hanley DF. Early Outpatient Treatment for Covid-19 with Convalescent Plasma. N Engl J Med. 2022 May 5;386(18):1700-1711. doi: 10.1056/NEJMoa2119657.
20. Huang DT, McCreary EK, Bariola JR, Minnier TE, Wadas RJ, Shovel JA, Albin D, Marroquin OC, Kip KE, Collins K, Schmidhofer M, Wisniewski MK, Nace DA, Sullivan C, Axe M, Meyers R, Weissman A, Garrard W, Peck-Palmer OM, Wells A, Bart RD, Yang A, Berry LR, Berry S, Crawford AM,

© 2023 Bai AD et al. *JAMA Network Open*.

McGlothlin A, Khadem T, Linstrum K, Montgomery SK, Ricketts D, Kennedy JN, Pidro CJ, Nakayama A, Zapf RL, Kip PL, Haidar G, Snyder GM, McVerry BJ, Yealy DM, Angus DC, Seymour CW. Effectiveness of Casirivimab-Imdevimab and Sotrovimab During a SARS-CoV-2 Delta Variant Surge: A Cohort Study and Randomized Comparative Effectiveness Trial. JAMA Netw Open. 2022 Jul 1;5(7):e2220957. doi: 10.1001/jamanetworkopen.2022.20957.

21. Weinreich DM, Sivapalasingam S, Norton T, Ali S, Gao H, Bhore R, Xiao J, Hooper AT, Hamilton JD, Musser BJ, Rofail D, Hussein M, Im J, Atmodjo DY, Perry C, Pan C, Mahmood A, Hosain R, Davis JD, Turner KC, Baum A, Kyratsous CA, Kim Y, Cook A, Kampman W, Roque-Guerrero L, Acloque G, Aazami H, Cannon K, Simón-Campos JA, Bocchini JA, Kowal B, DiCioccio AT, Soo Y, Geba GP, Stahl N, Lipsich L, Braunstein N, Herman G, Yancopoulos GD; Trial Investigators. REGEN-COV Antibody Combination and Outcomes in Outpatients with Covid-19. N Engl J Med. 2021 Dec 2;385(23):e81.
22. Parikh D, Chaturvedi A, Shah N, Patel P, Patel R, Ray S. Safety and Efficacy of COVID-19 Hyperimmune Globulin (HIG) Solution in the Treatment of Active COVID-19 infection-Findings from a Prospective, Randomized, Controlled, Multi-Centric Trial. The Indian Practitioner. 2021 Nov;74(11):15.
23. Ely EW, Ramanan AV, Kartman CE, de Bono S, Liao R, Piruzeli MLB, Goldman JD, Saraiva JFK, Chakladar S, Marconi VC; COV-BARRIER Study Group. Efficacy and safety of baricitinib plus standard of care for the treatment of critically ill hospitalised adults with COVID-19 on invasive mechanical ventilation or extracorporeal membrane oxygenation: an exploratory, randomised, placebo-controlled trial. Lancet Respir Med. 2022 Apr;10(4):327-336. doi: 10.1016/S2213-2600(22)00006-6.
24. Fedrizzi EN, Girondi JB, Sakae TM, Steffens SM, de Souza Silvestrin AN, Claro GS, Iskenderian HA, Hillmann B, Gervasi L, Trapani A, de Amorim Rodrigues P. (2022) Efficacy of the Measles-Mumps-Rubella (MMR) Vaccine in the Reducing the Severity of Covid-19: An Interim Analysis of a Randomised Controlled Clinical Trial. J Clin Trials. S14:009.
25. Kolev E, Mircheva L, Edwards MR, Johnston SL, Kalinov K, Stange R, Gancitano G, Berghe WV, Kreft S. *Echinacea Purpurea* For the Long-Term Prevention of Viral Respiratory Tract Infections During Covid-19 Pandemic: A Randomized, Open, Controlled, Exploratory Clinical Study. Front Pharmacol. 2022 Apr 26;13:856410. doi: 10.3389/fphar.2022.856410.
26. Breza E, Stanford FC, Alsan M, Alsan B, Banerjee A, Chandrasekhar AG, Eichmeyer S, Glushko T, Goldsmith-Pinkham P, Holland K, Hoppe E, Karnani M, Liegl S, Loisel T, Ogbu-Nwobodo L, Olken BA, Torres C, Vautrey PL, Warner ET, Wootton S, Duflo E. Effects of a large-scale social media advertising campaign on holiday travel and COVID-19 infections: a cluster randomized controlled trial. Nat Med. 2021 Sep;27(9):1622-1628. doi: 10.1038/s41591-021-01487-3.
27. Lattman E, Bhalerao P, ShashiBhushan BL, Nargundkar N, Lattmann P, Balaram PN. Randomized, Comparative, Clinical Trial to Evaluate Efficacy and Safety of PNB001 in Moderate COVID-19 Patients. Med J Clin Trials Case Stud 2021, 5(4): 000297. doi: 10.23880/mjccs-16000297.
28. Gaughan EE, Quinn TM, Mills A, Bruce AM, Antonelli J, MacKinnon A, Aslanis V, Li F, O'Connor R, Boz C, Mills R, Emanuel P, Burgess M, Rinaldi G, Valanciute A, Mills B, Scholefield E, Hardisty G, Gwyer Findlay E, Parker RA, Norrie J, Dear JW, Akram AR, Koch O, Templeton K, Dockrell DH, Walsh TS, Partridge S, Humphries D, Wang-Jairaj J, Slack RJ, Schambye H, Phung D, Gravelle L, Lindmark B, Shankar-Hari M, Hirani N, Sethi T, Dhaliwal K. An Inhaled Galectin-3 Inhibitor in COVID-19 Pneumonitis (DEFINE): A Phase Ib/IIa Randomised Controlled Trial. Am J Respir Crit Care Med. 2022 Aug 16. doi: 10.1164/rccm.202203-0477OC.
29. McCreary EK, Bariola JR, Minnier TE, Wadas RJ, Shovel JA, Albin D, Marroquin OC, Kip KE, Collins K, Schmidhofer M, Wisniewski MK, Nace DA, Sullivan C, Axe M, Meyers R, Weissman A, Garrard W, Peck-Palmer OM, Wells A, Bart RD, Yang A, Berry LR, Berry S, Crawford AM, McGlothlin A,

© 2023 Bai AD et al. *JAMA Network Open*.

Khadem T, Linstrum K, Montgomery SK, Ricketts D, Kennedy JN, Pidro CJ, Haidar G, Snyder GM, McVerry BJ, Yealy DM, Angus DC, Nakayama A, Zapf RL, Kip PL, Seymour CW, Huang DT. The comparative effectiveness of COVID-19 monoclonal antibodies: A learning health system randomized clinical trial. Contemp Clin Trials. 2022 Aug;119:106822. doi: 10.1016/j.cct.2022.106822.

30. Kyriazopoulou E, Poulakou G, Milionis H, Metallidis S, Adamis G, Tsiakos K, Fragkou A, Rapti A, Damoulari C, Fantoni M, Kalomenidis I, Chrysos G, Angheben A, Kainis I, Alexiou Z, Castelli F, Serino FS, Tsilika M, Bakakos P, Nicastri E, Tzavara V, Kostis E, Dagna L, Koufargyris P, Dimakou K, Savvanis S, Tzatzagou G, Chini M, Cavalli G, Bassetti M, Katrini K, Kotsis V, Tsoukalas G, Selmi C, Bliziotis I, Samarkos M, Doumas M, Ktena S, Masgala A, Papanikolaou I, Kosmidou M, Myrodia DM, Argyraki A, Cardellino CS, Koliakou K, Katsigianni EI, Rapti V, Giannitsioti E, Cingolani A, Micha S, Akinosoglou K, Liatsis-Douvitsas O, Symbardi S, Gatselis N, Mouktaroudi M, Ippolito G, Florou E, Kotsaki A, Netea MG, Eugen-Olsen J, Kyprianou M, Panagopoulos P, Dalekos GN, Giamarellos-Bourboulis EJ. Early treatment of COVID-19 with anakinra guided by soluble urokinase plasminogen receptor plasma levels: a double-blind, randomized controlled phase 3 trial. Nat Med. 2021 Oct;27(10):1752-1760. doi: 10.1038/s41591-021-01499-z.

31. Mikhaylov EN, Lyubimtseva TA, Vakhrushev AD, Stepanov D, Lebedev DS, Vasilieva EY, Konradi AO, Shlyakhto EV. Bromhexine Hydrochloride Prophylaxis of COVID-19 for Medical Personnel: A Randomized Open-Label Study. Interdiscip Perspect Infect Dis. 2022 Jan 29;2022:4693121. doi: 10.1155/2022/4693121.

32. REMAP-CAP Investigators; ACTIV-4a Investigators; ATTACC Investigators, Goligher EC, Bradbury CA, McVerry BJ, Lawler PR, Berger JS, Gong MN, Carrier M, Reynolds HR, Kumar A, Turgeon AF, Kornblith LZ, Kahn SR, Marshall JC, Kim KS, Houston BL, Derde LPG, Cushman M, Tritschler T, Angus DC, Godoy LC, McQuilten Z, Kirwan BA, Farkouh ME, Brooks MM, Lewis RJ, Berry LR, Lorenzi E, Gordon AC, Ahuja T, Al-Beidh F, Annane D, Arabi YM, Aryal D, Baumann Kreuziger L, Beane A, Bhimani Z, Bihari S, Billett HH, Bond L, Bonten M, Brunkhorst F, Buxton M, Buzgau A, Castellucci LA, Chekuri S, Chen JT, Cheng AC, Chkhikvadze T, Coiffard B, Contreras A, Costantini TW, de Brouwer S, Detry MA, Duggal A, Džavík V, Effron MB, Eng HF, Escobedo J, Estcourt LJ, Everett BM, Fergusson DA, Fitzgerald M, Fowler RA, Froess JD, Fu Z, Galanaud JP, Galen BT, Gandotra S, Girard TD, Goodman AL, Goossens H, Green C, Greenstein YY, Gross PL, Haniffa R, Hegde SM, Hendrickson CM, Higgins AM, Hindenburg AA, Hope AA, Horowitz JM, Horvat CM, Huang DT, Hudock K, Hunt BJ, Husain M, Hyzy RC, Jacobson JR, Jayakumar D, Keller NM, Khan A, Kim Y, Kindzelski A, King AJ, Knudson MM, Kornblith AE, Kutcher ME, Laffan MA, Lamontagne F, Le Gal G, Leeper CM, Leifer ES, Lim G, Gallego Lima F, Linstrum K, Litton E, Lopez-Sendon J, Lother SA, Marten N, Saud Marinez A, Martinez M, Mateos Garcia E, Mavromichalis S, McAuley DF, McDonald EG, McGlothlin A, McGuinness SP, Middeldorp S, Montgomery SK, Mouncey PR, Murthy S, Nair GB, Nair R, Nichol AD, Nicolau JC, Nunez-Garcia B, Park JJ, Park PK, Parke RL, Parker JC, Parnia S, Paul JD, Pompilio M, Quigley JG, Rosenson RS, Rost NS, Rowan K, Santos FO, Santos M, Santos MO, Satterwhite L, Saunders CT, Schreiber J, Schutgens REG, Seymour CW, Siegal DM, Silva DG Jr, Singhal AB, Slutsky AS, Solvason D, Stanworth SJ, Turner AM, van Bentum-Puijk W, van de Veerdonk FL, van Diepen S, Vazquez-Grande G, Wahid L, Wareham V, Widmer RJ, Wilson JG, Yuriditsky E, Zhong Y, Berry SM, McArthur CJ, Neal MD, Hochman JS, Webb SA, Zarychanski R. Therapeutic Anticoagulation with Heparin in Critically Ill Patients with Covid-19. N Engl J Med. 2021 Aug 26;385(9):777-789. doi: 10.1056/NEJMoa2103417.

33. Syed F, Hassan M, Arif MA, Batool S, Niazi R, Laila UE, Ashraf S, Arshad J. Pre-exposure Prophylaxis With Various Doses of Hydroxychloroquine Among Healthcare Personnel With High-Risk Exposure to COVID-19: A Randomized Controlled Trial. Cureus. 2021 Dec 21;13(12):e20572. doi: 10.7759/cureus.20572.

34. Cadegiani FA, Zimerman RA, Fonseca DN, Correia MN, Muller MP, Bet DL, Slaviero MR, Zardo I, Benites PR, Barros RN, Paulain RW, Onety DC, Israel KCP, Gustavo Wambier C, Goren A. Final Results of a Randomized, Placebo-Controlled, Two-Arm, Parallel Clinical Trial of Proxalutamide

© 2023 Bai AD et al. *JAMA Network Open*.

for Hospitalized COVID-19 Patients: A Multiregional, Joint Analysis of the Proxa-Rescue AndroCoV Trial. Cureus. 2021 Dec 25;13(12):e20691. doi: 10.7759/cureus.20691.
35. Ader F, Peiffer-Smadja N, Poissy J, Bouscambert-Duchamp M, Belhadi D, Diallo A, Delmas C, Saillard J, Dechanet A, Mercier N, Dupont A, Alfaiate T, Lescure FX, Raffi F, Goehringer F, Kimmoun A, Jaureguiberry S, Reignier J, Nseir S, Danion F, Clere-Jehl R, Bouiller K, Navellou JC, Tolsma V, Cabié A, Dubost C, Courjon J, Leroy S, Mootien J, Gaci R, Mourvillier B, Faure E, Pourcher V, Gallien S, Launay O, Lacombe K, Lanoix JP, Makinson A, Martin-Blondel G, Bouadma L, Botelho-Nevers E, Gagneux-Brunon A, Epaulard O, Piroth L, Wallet F, Richard JC, Reuter J, Staub T, Lina B, Noret M, Andrejak C, Lê MP, Peytavin G, Hites M, Costagliola D, Yazdanpanah Y, Burdet C, Mentré F; DisCoVeRy study group. An open-label randomized controlled trial of the effect of lopinavir/ritonavir, lopinavir/ritonavir plus IFN-β-1a and hydroxychloroquine in hospitalized patients with COVID-19. Clin Microbiol Infect. 2021 Dec;27(12):1826-1837. doi: 10.1016/j.cmi.2021.05.020.
36. Fralick M, Colacci M, Munshi L, Venus K, Fidler L, Hussein H, Britto K, Fowler R, da Costa BR, Dhalla I, Dunbar-Yaffe R, Branfield Day L, MacMillan TE, Zipursky J, Carpenter T, Tang T, Cooke A, Hensel R, Bregger M, Gordon A, Worndl E, Go S, Mandelzweig K, Castellucci LA, Tamming D, Razak F, Verma AA; COVID Prone Study Investigators. Prone positioning of patients with moderate hypoxaemia due to covid-19: multicentre pragmatic randomised trial (COVID-PRONE). BMJ. 2022 Mar 23;376:e068585. doi: 10.1136/bmj-2021-068585.
37. Hernández-Bernal F, Ricardo-Cobas MC, Martín-Bauta Y, Navarro-Rodríguez Z, Piñera-Martínez M, Quintana-Guerra J, Urrutia-Pérez K, Urrutia-Pérez K, Chávez-Chong CO, Azor-Hernández JL, Rodríguez-Reinoso JL, Lobaina-Lambert L, Colina-Ávila E, Bizet-Almeida J, Rodríguez-Nuviola J, Del Valle-Piñera S, Ramírez-Domínguez M, Tablada-Ferreiro E, Alonso-Valdés M, Lemos-Pérez G, Guillén-Nieto GE, Palenzuela-Díaz A, Noa-Romero E, Limonta-Fernández M, Fernández-Ávila JM, Ali-Mros NA, Del Toro-Lahera L, Remedios-Reyes R, Ayala-Ávila M, Muzio-González VL. Safety, tolerability, and immunogenicity of a SARS-CoV-2 recombinant spike RBD protein vaccine: A randomised, double-blind, placebo-controlled, phase 1-2 clinical trial (ABDALA Study). EClinicalMedicine. 2022 Apr;46:101383. doi: 10.1016/j.eclinm.2022.101383.
38. Lescure FX, Honda H, Fowler RA, Lazar JS, Shi G, Wung P, Patel N, Hagino O; Sarilumab COVID-19 Global Study Group. Sarilumab in patients admitted to hospital with severe or critical COVID-19: a randomised, double-blind, placebo-controlled, phase 3 trial. Lancet Respir Med. 2021 May;9(5):522-532. doi: 10.1016/S2213-2600(21)00099-0.
39. Perkins GD, Ji C, Connolly BA, Couper K, Lall R, Baillie JK, Bradley JM, Dark P, Dave C, De Soyza A, Dennis AV, Devrell A, Fairbairn S, Ghani H, Gorman EA, Green CA, Hart N, Hee SW, Kimbley Z, Madathil S, McGowan N, Messer B, Naisbitt J, Norman C, Parekh D, Parkin EM, Patel J, Regan SE, Ross C, Rostron AJ, Saim M, Simonds AK, Skilton E, Stallard N, Steiner M, Vancheeswaran R, Yeung J, McAuley DF; RECOVERY-RS Collaborators. Effect of Noninvasive Respiratory Strategies on Intubation or Mortality Among Patients With Acute Hypoxemic Respiratory Failure and COVID-19: The RECOVERY-RS Randomized Clinical Trial. JAMA. 2022 Feb 8;327(6):546-558. doi: 10.1001/jama.2022.0028.
40. Tornling G, Batta R, Porter JC, Williams B, Bengtsson T, Parmar K, Kashiva R, Hallberg A, Cohrt AK, Westergaard K, Dalsgaard CJ, Raud J. Seven days treatment with the angiotensin II type 2 receptor agonist C21 in hospitalized COVID-19 patients; a placebo-controlled randomised multi-centre double-blind phase 2 trial. EClinicalMedicine. 2021 Nov;41:101152. doi: 10.1016/j.eclinm.2021.101152.
41. Resende GG, da Cruz Lage R, Lobê SQ, Medeiros AF, Costa E Silva AD, Nogueira Sá AT, Oliveira AJA, Sousa D, Guimarães HC, Gomes IC, Souza RP, Aguiar RS, Tunala R, Forestiero F, Bueno Filho JSS, Teixeira MM. Blockade of interleukin seventeen (IL-17A) with secukinumab in hospitalized COVID-19 patients - the BISHOP study. Infect Dis (Lond). 2022 Apr 29:1-9. doi: 10.1080/23744235.2022.2066171.
42. Dai H, Saccardo S, Han MA, Roh L, Raja N, Vangala S, Modi H, Pandya S, Sloyan M, Croymans DM. Behavioural nudges increase COVID-19 vaccinations. Nature. 2021 Sep;597(7876):404-409. doi: 10.1038/s41586-021-03843-2.

© 2023 Bai AD et al. *JAMA Network Open*.

43. Gaitán-Duarte HG, Álvarez-Moreno C, Rincón-Rodríguez CJ, Yomayusa-González N, Cortés JA, Villar JC, Bravo-Ojeda JS, García-Peña A, Adarme-Jaimes W, Rodríguez-Romero VA, Villate-Soto SL, Buitrago G, Chacón-Sarmiento J, Macias-Quintero M, Vaca CP, Gómez-Restrepo C, Rodríguez-Malagón N. Effectiveness of rosuvastatin plus colchicine, emtricitabine/tenofovir and combinations thereof in hospitalized patients with COVID-19: a pragmatic, open-label randomized trial. EClinicalMedicine. 2022 Jan;43:101242. doi: 10.1016/j.eclinm.2021.101242.
44. Arnardottir H, Pawelzik SC, Sarajlic P, Quaranta A, Kolmert J, Religa D, Wheelock CE, Bäck M. Immunomodulation by intravenous omega-3 fatty acid treatment in older subjects hospitalized for COVID-19: A single-blind randomized controlled trial. Clin Transl Med. 2022 Sep;12(9):e895. doi: 10.1002/ctm2.895.
45. Pan HX, Liu JK, Huang BY, Li GF, Chang XY, Liu YF, Wang WL, Chu K, Hu JL, Li JX, Zhu DD, Wu JL, Xu XY, Zhang L, Wang M, Tan WJ, Huang WJ, Zhu FC. Immunogenicity and safety of a severe acute respiratory syndrome coronavirus 2 inactivated vaccine in healthy adults: randomized, double-blind, and placebo-controlled phase 1 and phase 2 clinical trials. Chin Med J (Engl). 2021 Apr 28;134(11):1289-1298. doi: 10.1097/CM9.0000000000001573.
46. Almanza-Reyes H, Moreno S, Plascencia-López I, Alvarado-Vera M, Patrón-Romero L, Borrego B, Reyes-Escamilla A, Valencia-Manzo D, Brun A, Pestryakov A, Bogdanchikova N. Evaluation of silver nanoparticles for the prevention of SARS-CoV-2 infection in health workers: In vitro and in vivo. PLoS One. 2021 Aug 19;16(8):e0256401. doi: 10.1371/journal.pone.0256401.
47. Tardif JC, Bouabdallaoui N, L'Allier PL, Gaudet D, Shah B, Pillinger MH, Lopez-Sendon J, da Luz P, Verret L, Audet S, Dupuis J, Denault A, Pelletier M, Tessier PA, Samson S, Fortin D, Tardif JD, Busseuil D, Goulet E, Lacoste C, Dubois A, Joshi AY, Waters DD, Hsue P, Lepor NE, Lesage F, Sainturet N, Roy-Clavel E, Bassevitch Z, Orfanos A, Stamatescu G, Grégoire JC, Busque L, Lavallée C, Hétu PO, Paquette JS, Deftereos SG, Levesque S, Cossette M, Nozza A, Chabot-Blanchet M, Dubé MP, Guertin MC, Boivin G; COLCORONA Investigators. Colchicine for community-treated patients with COVID-19 (COLCORONA): a phase 3, randomised, double-blinded, adaptive, placebo-controlled, multicentre trial. Lancet Respir Med. 2021 Aug;9(8):924-932. doi: 10.1016/S2213-2600(21)00222-8.
48. Rossignol JF, Bardin MC, Fulgencio J, Mogelnicki D, Bréchot C. A randomized double-blind placebo-controlled clinical trial of nitazoxanide for treatment of mild or moderate COVID-19. EClinicalMedicine. 2022 Feb 28;45:101310. doi: 10.1016/j.eclinm.2022.101310.
49. ACTIV-3/TICO Bamlanivimab Study Group, Lundgren JD, Grund B, Barkauskas CE, Holland TL, Gottlieb RL, Sandkovsky U, Brown SM, Knowlton KU, Self WH, Files DC, Jain MK, Benfield T, Bowdish ME, Leshnower BG, Baker JV, Jensen JU, Gardner EM, Ginde AA, Harris ES, Johansen IS, Markowitz N, Matthay MA, Østergaard L, Chang CC, Goodman AL, Chang W, Dewar RL, Gerry NP, Higgs ES, Highbarger H, Murray DD, Murray TA, Natarajan V, Paredes R, Parmar MKB, Phillips AN, Reilly C, Rupert AW, Sharma S, Shaw-Saliba K, Sherman BT, Teitelbaum M, Wentworth D, Cao H, Klekotka P, Babiker AG, Davey VJ, Gelijns AC, Kan VL, Polizzotto MN, Thompson BT, Lane HC, Neaton JD. Responses to a Neutralizing Monoclonal Antibody for Hospitalized Patients With COVID-19 According to Baseline Antibody and Antigen Levels : A Randomized Controlled Trial. Ann Intern Med. 2022 Feb;175(2):234-243. doi: 10.7326/M21-3507.
50. Liu J, Yang W, Liu Y, Lu C, Ruan L, Zhao C, Huo R, Shen X, Miao Q, Lv W, Li H, Shi H, Hu L, Yang Z, Zhang L, Wang B, Dong G, Xian Y, Li B, Zhou Z, Xu C, Chen Y, Bian Y, Guo J, Yang J, Wang J, Qi W, Chen S, Chen Y, Yan B, Wang W, Li J, Xie X, Xu M, Jiang J, Wang G, Cong X, Zhu H, Shi J, Leng L, Li D, Guo L, Huang L. Combination of Hua Shi Bai Du granule (Q-14) and standard care in the treatment of patients with coronavirus disease 2019 (COVID-19): A single-center, open-label, randomized controlled trial. Phytomedicine. 2021 Oct;91:153671. doi: 10.1016/j.phymed.2021.153671.

© 2023 Bai AD et al. *JAMA Network Open*.

51. Li J, Hou L, Guo X, Jin P, Wu S, Zhu J, Pan H, Wang X, Song Z, Wan J, Cui L, Li J, Chen Y, Wang X, Jin L, Liu J, Shi F, Xu X, Zhu T, Chen W, Zhu F. Heterologous AD5-nCOV plus CoronaVac versus homologous CoronaVac vaccination: a randomized phase 4 trial. Nat Med. 2022 Feb;28(2):401-409. doi: 10.1038/s41591-021-01677-z.
52. Rojas-Serrano J, Portillo-Vásquez AM, Thirion-Romero I, Vázquez-Pérez J, Mejía-Nepomuceno F, Ramírez-Venegas A, Pérez-Kawabe KM, Pérez-Padilla R. Hydroxychloroquine for prophylaxis of COVID-19 in health workers: A randomized clinical trial. PLoS One. 2022 Feb 9;17(2):e0261980. doi: 10.1371/journal.pone.0261980.
53. Beltran Gonzalez JL, González Gámez M, Mendoza Enciso EA, Esparza Maldonado RJ, Hernández Palacios D, Dueñas Campos S, Robles IO, Macías Guzmán MJ, García Díaz AL, Gutiérrez Peña CM, Martinez Medina L, Monroy Colin VA, Arreola Guerra JM. Efficacy and Safety of Ivermectin and Hydroxychloroquine in Patients with Severe COVID-19: A Randomized Controlled Trial. Infect Dis Rep. 2022 Mar 3;14(2):160-168. doi: 10.3390/idr14020020.
54. Figueroa JM, Lombardo ME, Dogliotti A, Flynn LP, Giugliano R, Simonelli G, Valentini R, Ramos A, Romano P, Marcote M, Michelini A, Salvado A, Sykora E, Kniz C, Kobelinsky M, Salzberg DM, Jerusalinsky D, Uchitel O. Efficacy of a Nasal Spray Containing Iota-Carrageenan in the Postexposure Prophylaxis of COVID-19 in Hospital Personnel Dedicated to Patients Care with COVID-19 Disease. Int J Gen Med. 2021 Oct 1;14:6277-6286. doi: 10.2147/IJGM.S328486. PMID: 34629893; PMCID: PMC8493111.
55. Winthrop KL, Skolnick AW, Rafiq AM, Beegle SH, Suszanski J, Koehne G, Barnett-Griness O, Bibliowicz A, Fathi R, Anderson P, Raday G, Eagle G, Ben-Yair VK, Minkowitz HS, Levitt ML, Gordon MS. Opaganib in Coronavirus Disease 2019 Pneumonia: Results of a Randomized, Placebo-Controlled Phase 2a Trial. Open Forum Infect Dis. 2022 May 11;9(7):ofac232. doi: 10.1093/ofid/ofac232.
56. Wanaratna K, Leethong P, Inchai N, Chueawiang W, Sriraksa P, Tabmee A, Sirinavin S. Efficacy and Safety of Andrographis Paniculata Extract in Patients with Mild COVID-19: A Randomized Controlled Trial. Archives of Internal Medicine Research 5 (2022): 423-427.
57. Damle L, Damle H, Br B. Plant formulation ATRICOV 452 in improving the level of COVID-19 specific inflammatory markers in patients. Contemp Clin Trials Commun. 2022 Jul 4;28:100961. doi: 10.1016/j.conctc.2022.100961.
58. Dunkle LM, Kotloff KL, Gay CL, Áñez G, Adelglass JM, Barrat Hernández AQ, Harper WL, Duncanson DM, McArthur MA, Florescu DF, McClelland RS, Garcia-Fragoso V, Riesenberg RA, Musante DB, Fried DL, Safirstein BE, McKenzie M, Jeanfreau RJ, Kingsley JK, Henderson JA, Lane DC, Ruíz-Palacios GM, Corey L, Neuzil KM, Coombs RW, Greninger AL, Hutter J, Ake JA, Smith K, Woo W, Cho I, Glenn GM, Dubovsky F; 2019nCoV-301 Study Group. Efficacy and Safety of NVX-CoV2373 in Adults in the United States and Mexico. N Engl J Med. 2022;386(6):531-543. doi: 10.1056/NEJMoa2116185.
59. Maskin LP, Bonelli I, Olarte GL, Palizas F Jr, Velo AE, Lurbet MF, Lovazzano P, Kotsias S, Attie S, Lopez Saubidet I, Baredes ND, Setten M, Rodriguez PO. High- Versus Low-Dose Dexamethasone for the Treatment of COVID-19-Related Acute Respiratory Distress Syndrome: A Multicenter, Randomized Open-Label Clinical Trial. J Intensive Care Med. 2022 Apr;37(4):491-499. doi: 10.1177/08850666211066799.
60. Silveira MAD, De Jong D, Berretta AA, Galvão EBDS, Ribeiro JC, Cerqueira-Silva T, Amorim TC, Conceição LFMRD, Gomes MMD, Teixeira MB, Souza SP, Santos MHCAD, San Martin RLA, Silva MO, Lírio M, Moreno L, Sampaio JCM, Mendonça R, Ultchak SS, Amorim FS, Ramos JGR, Batista PBP, Guarda SNFD, Mendes AVA, Passos RDH; BeeCovid Team. Efficacy of Brazilian green propolis (EPP-AF®) as an adjunct treatment for hospitalized COVID-19 patients: A randomized, controlled clinical trial. Biomed Pharmacother. 2021;138:111526. doi: 10.1016/j.biopha.2021.111526.

© 2023 Bai AD et al. *JAMA Network Open*.

61. Eugenia-Toledo-Romaní M, Verdecia-Sánchez L, Rodríguez-González M, Rodríguez-Noda L, Valenzuela-Silva C, Paredes-Moreno B, Sánchez-Ramírez B, Pérez-Nicado R, González-Mugica R, Hernández-García T, Bergado-Baez G, Pi-Estopiñán F, Cruz-Sui O, Fraga-Quintero A, García-Montero M, Palenzuela-Díaz A, Baró-Román G, Mendoza-Hernández I, Fernandez-Castillo S, Climent-Ruiz Y, Santana-Mederos D, Ramírez Gonzalez U, García-Vega Y, Pérez-Massón B, Guang-Wu-Chen, Boggiano-Ayo T, Ojito-Magaz E, Rivera DG, Valdés-Balbín Y, García-Rivera D, Vérez-Bencomo V; SOBERANA Research Group, Gómez-Maceo Y, Reyes-Matienzo R, Manuel Coviella-Artime J, Morffi-Cinta I, Martínez-Pérez M, Castillo-Quintana I, Garcés-Hechavarría A, Valera-Fernández R, Martínez-Bedoya D, Garrido-Arteaga R, Cardoso-SanJorge F, Quintero Moreno L, Ontivero-Pino I, Teresa Pérez-Guevara M, Morales-García M, Noa-Romero E, Orosa-Vázquez I, Díaz-Hernández M, Rojas G, Tundidor Y, García-López E, Muñoz-Morejon Y, Galano-Frutos E, Rodríguez-Alvarez J, Arteaga A, Medina Nápoles M, Espi Ávila J, Fontanies Fernández M. Safety and immunogenicity of anti-SARS CoV-2 vaccine SOBERANA 02 in homologous or heterologous scheme: Open label phase I and phase IIa clinical trials. Vaccine. 2022 Jul 29;40(31):4220-4230. doi: 10.1016/j.vaccine.2022.05.082.
62. Tsilika M, Taks E, Dolianitis K, Kotsaki A, Leventogiannis K, Damoulari C, Kostoula M, Paneta M, Adamis G, Papanikolaou I, Stamatelopoulos K, Bolanou A, Katsaros K, Delavinia C, Perdios I, Pandi A, Tsiakos K, Proios N, Kalogianni E, Delis I, Skliros E, Akinosoglou K, Perdikouli A, Poulakou G, Milionis H, Athanassopoulou E, Kalpaki E, Efstratiou L, Perraki V, Papadopoulos A, Netea MG, Giamarellos-Bourboulis EJ. ACTIVATE-2: A Double-Blind Randomized Trial of BCG Vaccination Against COVID-19 in Individuals at Risk. Front Immunol. 2022 Jul 5;13:873067. doi: 10.3389/fimmu.2022.873067.
63. Holubar M, Subramanian A, Purington N, Hedlin H, Bunning B, Walter KS, Bonilla H, Boumis A, Chen M, Clinton K, Dewhurst L, Epstein C, Jagannathan P, Kaszynski RH, Panu L, Parsonnet J, Ponder EL, Quintero O, Sefton E, Singh U, Soberanis L, Truong H, Andrews JR, Desai M, Khosla C, Maldonado Y. Favipiravir for treatment of outpatients with asymptomatic or uncomplicated COVID-19: a double-blind randomized, placebo-controlled, phase 2 trial. Clin Infect Dis. 2022 Apr 21:ciac312. doi: 10.1093/cid/ciac312.
64. O'Donnell MR, Grinsztejn B, Cummings MJ, Justman JE, Lamb MR, Eckhardt CM, Philip NM, Cheung YK, Gupta V, João E, Pilotto JH, Diniz MP, Cardoso SW, Abrams D, Rajagopalan KN, Borden SE, Wolf A, Sidi LC, Vizzoni A, Veloso VG, Bitan ZC, Scotto DE, Meyer BJ, Jacobson SD, Kantor A, Mishra N, Chauhan LV, Stone EF, Dei Zotti F, La Carpia F, Hudson KE, Ferrara SA, Schwartz J, Stotler BA, Lin WW, Wontakal SN, Shaz B, Briese T, Hod EA, Spitalnik SL, Eisenberger A, Lipkin WI. A randomized double-blind controlled trial of convalescent plasma in adults with severe COVID-19. J Clin Invest. 2021 Jul 1;131(13):e150646. doi: 10.1172/JCI150646.
65. O'Brien MP, Forleo-Neto E, Musser BJ, Isa F, Chan KC, Sarkar N, Bar KJ, Barnabas RV, Barouch DH, Cohen MS, Hurt CB, Burwen DR, Marovich MA, Hou P, Heirman I, Davis JD, Turner KC, Ramesh D, Mahmood A, Hooper AT, Hamilton JD, Kim Y, Purcell LA, Baum A, Kyratsous CA, Krainson J, Perez-Perez R, Mohseni R, Kowal B, DiCioccio AT, Stahl N, Lipsich L, Braunstein N, Herman G, Yancopoulos GD, Weinreich DM; Covid-19 Phase 3 Prevention Trial Team. Subcutaneous REGEN-COV Antibody Combination to Prevent Covid-19. N Engl J Med. 2021 Sep 23;385(13):1184-1195. doi: 10.1056/NEJMoa2109682.
66. O'Brien MP, Forleo-Neto E, Sarkar N, Isa F, Hou P, Chan KC, Musser BJ, Bar KJ, Barnabas RV, Barouch DH, Cohen MS, Hurt CB, Burwen DR, Marovich MA, Brown ER, Heirman I, Davis JD, Turner KC, Ramesh D, Mahmood A, Hooper AT, Hamilton JD, Kim Y, Purcell LA, Baum A, Kyratsous CA, Krainson J, Perez-Perez R, Mohseni R, Kowal B, DiCioccio AT, Geba GP, Stahl N, Lipsich L, Braunstein N, Herman G, Yancopoulos GD, Weinreich DM; COVID-19 Phase 3 Prevention Trial Team. Effect of Subcutaneous Casirivimab and Imdevimab Antibody Combination vs Placebo on Development of Symptomatic COVID-19 in Early Asymptomatic SARS-CoV-2 Infection: A Randomized Clinical Trial. JAMA. 2022 Feb 1;327(5):432-441. doi: 10.1001/jama.2021.24939.

© 2023 Bai AD et al. *JAMA Network Open*.

67. Puskarich MA, Ingraham NE, Merck LH, Driver BE, Wacker DA, Black LP, Jones AE, Fletcher CV, South AM, Murray TA, Lewandowski C, Farhat J, Benoit JL, Biros MH, Cherabuddi K, Chipman JG, Schacker TW, Guirgis FW, Voelker HT, Koopmeiners JS, Tignanelli CJ; Angiotensin Receptor Blocker Based Lung Protective Strategies for Inpatients With COVID-19 (ALPS-IP) Investigators. Efficacy of Losartan in Hospitalized Patients With COVID-19-Induced Lung Injury: A Randomized Clinical Trial. JAMA Netw Open. 2022 Mar 1;5(3):e222735. doi: 10.1001/jamanetworkopen.2022.2735.
68. Bonelli M, Mrak D, Tobudic S, Sieghart D, Koblischke M, Mandl P, Kornek B, Simader E, Radner H, Perkmann T, Haslacher H, Mayer M, Hofer P, Redlich K, Husar-Memmer E, Fritsch-Stork R, Thalhammer R, Stiasny K, Winkler S, Smolen JS, Aberle JH, Zeitlinger M, Heinz LX, Aletaha D. Additional heterologous versus homologous booster vaccination in immunosuppressed patients without SARS-CoV-2 antibody seroconversion after primary mRNA vaccination: a randomised controlled trial. Ann Rheum Dis. 2022;81(5):687-694. doi: 10.1136/annrheumdis-2021-221558.
69. Sholzberg M, Tang GH, Rahhal H, AlHamzah M, Kreuziger LB, Áinle FN, Alomran F, Alayed K, Alsheef M, AlSumait F, Pompilio CE, Sperlich C, Tangri S, Tang T, Jaksa P, Suryanarayan D, Almarshoodi M, Castellucci LA, James PD, Lillicrap D, Carrier M, Beckett A, Colovos C, Jayakar J, Arsenault MP, Wu C, Doyon K, Andreou ER, Dounaevskaia V, Tseng EK, Lim G, Fralick M, Middeldorp S, Lee AYY, Zuo F, da Costa BR, Thorpe KE, Negri EM, Cushman M, Jüni P; RAPID trial investigators. Effectiveness of therapeutic heparin versus prophylactic heparin on death, mechanical ventilation, or intensive care unit admission in moderately ill patients with covid-19 admitted to hospital: RAPID randomised clinical trial. BMJ. 2021 Oct 14;375:n2400. doi: 10.1136/bmj.n2400.
70. Zeng G, Wu Q, Pan H, Li M, Yang J, Wang L, Wu Z, Jiang D, Deng X, Chu K, Zheng W, Wang L, Lu W, Han B, Zhao Y, Zhu F, Yu H, Yin W. Immunogenicity and safety of a third dose of CoronaVac, and immune persistence of a two-dose schedule, in healthy adults: interim results from two single-centre, double-blind, randomised, placebo-controlled phase 2 clinical trials. Lancet Infect Dis. 2022 Apr;22(4):483-495. doi: 10.1016/S1473-3099(21)00681-2.
71. Elgohary MA, Hasan EM, Ibrahim AA, Abdelsalam MFA, Abdel-Rahman RZ, Zaki AI, Elaatar MB, Elnagar MT, Emam ME, Hamada MM, Abdel-Hamid TM, Abdel-Hafez AS, Seadawy MG, Fatoh AR, Elsaied MA, Sakr MA, Elkady AO, Shehata MM, Nawar OM, Selem MA, Abd-Aal MS, Lotfy HH, Elnagdy TR, Helmy S, Mubark MA. Efficacy of Sofosbuvir plus Ledipasvir in Egyptian patients with COVID-19 compared to standard treatment: a randomized controlled trial. J Med Life. 2022 Mar;15(3):350-358. doi: 10.25122/jml-2021-0175.
72. Formica N, Mallory R, Albert G, Robinson M, Plested JS, Cho I, Robertson A, Dubovsky F, Glenn GM; 2019nCoV-101 Study Group. Different dose regimens of a SARS-CoV-2 recombinant spike protein vaccine (NVX-CoV2373) in younger and older adults: A phase 2 randomized placebo-controlled trial. PLoS Med. 2021 Oct 1;18(10):e1003769. doi: 10.1371/journal.pmed.1003769.
73. Morici N, Podda G, Birocchi S, Bonacchini L, Merli M, Trezzi M, Massaini G, Agostinis M, Carioti G, Saverio Serino F, Gazzaniga G, Barberis D, Antolini L, Grazia Valsecchi M, Cattaneo M. Enoxaparin for thromboprophylaxis in hospitalized COVID-19 patients: The X-COVID-19 Randomized Trial. Eur J Clin Invest. 2022 May;52(5):e13735. doi: 10.1111/eci.13735.
74. Babalola OE, Bode CO, Ajayi AA, Alakaloko FM, Akase IE, Otrofanowei E, Salu OB, Adeyemo WL, Ademuyiwa AO, Omilabu S. Ivermectin shows clinical benefits in mild to moderate COVID19: a randomized controlled double-blind, dose-response study in Lagos. QJM. 2022 Jan 5;114(11):780-788. doi: 10.1093/qjmed/hcab035.
75. ATTACC Investigators; ACTIV-4a Investigators; REMAP-CAP Investigators, Lawler PR, Goligher EC, Berger JS, Neal MD, McVerry BJ, Nicolau JC, Gong MN, Carrier M, Rosenson RS, Reynolds HR, Turgeon AF, Escobedo J, Huang DT, Bradbury CA, Houston BL, Kornblith LZ, Kumar A, Kahn SR, Cushman M, McQuilten Z, Slutsky AS, Kim KS, Gordon AC, Kirwan BA, Brooks MM, Higgins AM, Lewis RJ, Lorenzi E, Berry SM, Berry LR, Aday AW,

© 2023 Bai AD et al. *JAMA Network Open*.

Al-Beidh F, Annane D, Arabi YM, Aryal D, Baumann Kreuziger L, Beane A, Bhimani Z, Bihari S, Billett HH, Bond L, Bonten M, Brunkhorst F, Buxton M, Buzgau A, Castellucci LA, Chekuri S, Chen JT, Cheng AC, Chkhikvadze T, Coiffard B, Costantini TW, de Brouwer S, Derde LPG, Detry MA, Duggal A, Džavík V, Effron MB, Estcourt LJ, Everett BM, Fergusson DA, Fitzgerald M, Fowler RA, Galanaud JP, Galen BT, Gandotra S, García-Madrona S, Girard TD, Godoy LC, Goodman AL, Goossens H, Green C, Greenstein YY, Gross PL, Hamburg NM, Haniffa R, Hanna G, Hanna N, Hegde SM, Hendrickson CM, Hite RD, Hindenburg AA, Hope AA, Horowitz JM, Horvat CM, Hudock K, Hunt BJ, Husain M, Hyzy RC, Iyer VN, Jacobson JR, Jayakumar D, Keller NM, Khan A, Kim Y, Kindzelski AL, King AJ, Knudson MM, Kornblith AE, Krishnan V, Kutcher ME, Laffan MA, Lamontagne F, Le Gal G, Leeper CM, Leifer ES, Lim G, Lima FG, Linstrum K, Litton E, Lopez-Sendon J, Lopez-Sendon Moreno JL, Lother SA, Malhotra S, Marcos M, Saud Marinez A, Marshall JC, Marten N, Matthay MA, McAuley DF, McDonald EG, McGlothlin A, McGuinness SP, Middeldorp S, Montgomery SK, Moore SC, Morillo Guerrero R, Mouncey PR, Murthy S, Nair GB, Nair R, Nichol AD, Nunez-Garcia B, Pandey A, Park PK, Parke RL, Parker JC, Parnia S, Paul JD, Pérez González YS, Pompilio M, Prekker ME, Quigley JG, Rost NS, Rowan K, Santos FO, Santos M, Olombrada Santos M, Satterwhite L, Saunders CT, Schutgens REG, Seymour CW, Siegal DM, Silva DG Jr, Shankar-Hari M, Sheehan JP, Singhal AB, Solvason D, Stanworth SJ, Tritschler T, Turner AM, van Bentum-Puijk W, van de Veerdonk FL, van Diepen S, Vazquez-Grande G, Wahid L, Wareham V, Wells BJ, Widmer RJ, Wilson JG, Yuriditsky E, Zampieri FG, Angus DC, McArthur CJ, Webb SA, Farkouh ME, Hochman JS, Zarychanski R. Therapeutic Anticoagulation with Heparin in Noncritically Ill Patients with Covid-19. N Engl J Med. 2021 Aug 26;385(9):790-802. doi: 10.1056/NEJMoa2105911.

76. Goepfert PA, Fu B, Chabanon AL, Bonaparte MI, Davis MG, Essink BJ, Frank I, Haney O, Janosczyk H, Keefer MC, Koutsoukos M, Kimmel MA, Masotti R, Savarino SJ, Schuerman L, Schwartz H, Sher LD, Smith J, Tavares-Da-Silva F, Gurunathan S, DiazGranados CA, de Bruyn G. Safety and immunogenicity of SARS-CoV-2 recombinant protein vaccine formulations in healthy adults: interim results of a randomised, placebo-controlled, phase 1-2, dose-ranging study. Lancet Infect Dis. 2021 Sep;21(9):1257-1270. doi: 10.1016/S1473-3099(21)00147-X.

77. Gutiérrez-Castrellón P, Gandara-Martí T, Abreu Y Abreu AT, Nieto-Rufino CD, López-Orduña E, Jiménez-Escobar I, Jiménez-Gutiérrez C, López-Velazquez G, Espadaler-Mazo J. Probiotic improves symptomatic and viral clearance in Covid19 outpatients: a randomized, quadruple-blinded, placebo-controlled trial. Gut Microbes. 2022;14(1):2018899. doi: 10.1080/19490976.2021.2018899.

78. RECOVERY Collaborative Group. Aspirin in patients admitted to hospital with COVID-19 (RECOVERY): a randomised, controlled, open-label, platform trial. Lancet. 2022 Jan 8;399(10320):143-151. doi: 10.1016/S0140-6736(21)01825-0.

79. RECOVERY Collaborative Group. Convalescent plasma in patients admitted to hospital with COVID-19 (RECOVERY): a randomised controlled, open-label, platform trial. Lancet. 2021 May 29;397(10289):2049-2059. doi: 10.1016/S0140-6736(21)00897-7.

80. RECOVERY Collaborative Group. Casirivimab and imdevimab in patients admitted to hospital with COVID-19 (RECOVERY): a randomised, controlled, open-label, platform trial. Lancet. 2022 Feb 12;399(10325):665-676. doi: 10.1016/S0140-6736(22)00163-5.

81. RECOVERY Collaborative Group. Colchicine in patients admitted to hospital with COVID-19 (RECOVERY): a randomised, controlled, open-label, platform trial. Lancet Respir Med. 2021 Dec;9(12):1419-1426. doi: 10.1016/S2213-2600(21)00435-5.

82. RECOVERY Collaborative Group. Tocilizumab in patients admitted to hospital with COVID-19 (RECOVERY): a randomised, controlled, open-label, platform trial. Lancet. 2021 May 1;397(10285):1637-1645. doi: 10.1016/S0140-6736(21)00676-0.

83. Bégin P, Callum J, Jamula E, Cook R, Heddle NM, Tinmouth A, Zeller MP, Beaudoin-Bussières G, Amorim L, Bazin R, Loftsgard KC, Carl R, Chassé M, Cushing MM, Daneman N, Devine DV, Dumaresq J, Fergusson DA, Gabe C, Glesby MJ, Li N, Liu Y, McGeer A, Robitaille N, Sachais BS, Scales DC, Schwartz L, Shehata N, Turgeon AF, Wood H, Zarychanski R, Finzi A; CONCOR-1 Study Group, Arnold DM. Convalescent plasma for

© 2023 Bai AD et al. *JAMA Network Open*.

hospitalized patients with COVID-19: an open-label, randomized controlled trial. Nat Med. 2021 Nov;27(11):2012-2024. doi: 10.1038/s41591-021-01488-2.
84. Mallory RM, Formica N, Pfeiffer S, Wilkinson B, Marcheschi A, Albert G, McFall H, Robinson M, Plested JS, Zhu M, Cloney-Clark S, Zhou B, Chau G, Robertson A, Maciejewski S, Hammond HL, Baracco L, Logue J, Frieman MB, Smith G, Patel N, Glenn GM; Novavax 2019nCoV101 Study Group. Safety and immunogenicity following a homologous booster dose of a SARS-CoV-2 recombinant spike protein vaccine (NVX-CoV2373): a secondary analysis of a randomised, placebo-controlled, phase 2 trial. Lancet Infect Dis. 2022 Aug 10:S1473-3099(22)00420-0. doi: 10.1016/S1473-3099(22)00420-0.
85. Ella R, Reddy S, Blackwelder W, Potdar V, Yadav P, Sarangi V, Aileni VK, Kanungo S, Rai S, Reddy P, Verma S, Singh C, Redkar S, Mohapatra S, Pandey A, Ranganadin P, Gumashta R, Multani M, Mohammad S, Bhatt P, Kumari L, Sapkal G, Gupta N, Abraham P, Panda S, Prasad S, Bhargava B, Ella K, Vadrevu KM; COVAXIN Study Group. Efficacy, safety, and lot-to-lot immunogenicity of an inactivated SARS-CoV-2 vaccine (BBV152): interim results of a randomised, double-blind, controlled, phase 3 trial. Lancet. 2021 Dec 11;398(10317):2173-2184.
86. Lazarus R, Taucher C, Brown C, Čorbic Ramljak I, Danon L, Dubischar K, Duncan CJA, Eder-Lingelbach S, Faust SN, Green C, Gokani K, Hochreiter R, Wright JK, Kwon D, Middleditch A, Munro APS, Naker K, Penciu F, Price D, Querton B, Riaz T, Ross-Russell A, Sanchez-Gonzalez A, Wardle H, Warren S, Finn A; Valneva Phase 1 Trial Group. Safety and immunogenicity of the inactivated whole-virus adjuvanted COVID-19 vaccine VLA2001: A randomized, dose escalation, double-blind phase 1/2 clinical trial in healthy adults. J Infect. 2022 Sep;85(3):306-317. doi: 10.1016/j.jinf.2022.06.009.
87. Ravikirti, Roy R, Pattadar C, Raj R, Agarwal N, Biswas B, Manjhi PK, Rai DK, Shyama, Kumar A, Sarfaraz A. Evaluation of Ivermectin as a Potential Treatment for Mild to Moderate COVID-19: A Double-Blind Randomized Placebo Controlled Trial in Eastern India. J Pharm Pharm Sci. 2021;24:343-350. doi: 10.18433/jpps32105.
88. Ravichandran R, Mohan SK, Sukumaran SK, Kamaraj D, Daivasuga SS, Ravi SOAS, Vijayaraghavalu S, Kumar RK. An open label randomized clinical trial of Indomethacin for mild and moderate hospitalised Covid-19 patients. Sci Rep. 2022;12(1):6413. doi: 10.1038/s41598-022-10370-1.
89. Elzein R, Abdel-Sater F, Fakhreddine S, Hanna PA, Feghali R, Hamad H, Ayoub F. In vivo evaluation of the virucidal efficacy of chlorhexidine and povidone-iodine mouthwashes against salivary SARS-CoV-2. A randomized-controlled clinical trial. J Evid Based Dent Pract. 2021 Sep;21(3):101584. doi: 10.1016/j.jebdp.2021.101584.
90. Sablerolles RSG, Rietdijk WJR, Goorhuis A, Postma DF, Visser LG, Geers D, Schmitz KS, Garcia Garrido HM, Koopmans MPG, Dalm VASH, Kootstra NA, Huckriede ALW, Lafeber M, van Baarle D, GeurtsvanKessel CH, de Vries RD, van der Kuy PHM; SWITCH Research Group. Immunogenicity and Reactogenicity of Vaccine Boosters after Ad26.COV2.S Priming. N Engl J Med. 2022 Mar 10;386(10):951-963. doi: 10.1056/NEJMoa2116747.
91. Chahla RE, Medina Ruiz L, Ortega ES, Morales Rn MF, Barreiro F, George A, Mancilla Rn C, D' Amato Rn S, Barrenechea G, Goroso DG, Peral de Bruno M. Intensive Treatment With Ivermectin and Iota-Carrageenan as Pre-exposure Prophylaxis for COVID-19 in Health Care Workers From Tucuman, Argentina. Am J Ther. 2021;28(5):e601-e604. doi: 10.1097/MJT.0000000000001433.
92. Izikson R, Brune D, Bolduc JS, Bourron P, Fournier M, Moore TM, Pandey A, Perez L, Sater N, Shrestha A, Wague S, Samson SI. Safety and immunogenicity of a high-dose quadrivalent influenza vaccine administered concomitantly with a third dose of the mRNA-1273 SARS-CoV-2 vaccine in adults aged ≥65 years: a phase 2, randomised, open-label study. Lancet Respir Med. 2022 Apr;10(4):392-402. doi: 10.1016/S2213-2600(21)00557-9.

© 2023 Bai AD et al. *JAMA Network Open*.

93. Ramakrishnan S, Nicolau Jr DV, Langford B, Mahdi M, Jeffers H, Mwasuku C, Krassowska K, Fox R, Binnian I, Glover V, Bright S. Inhaled budesonide in the treatment of early COVID-19 (STOIC): a phase 2, open-label, randomised controlled trial. The Lancet Respiratory Medicine. 2021 Jul 1;9(7):763-72. doi: 10.1016/S2213-2600(21)00160-0.
94. Sridhar S, Joaquin A, Bonaparte MI, Bueso A, Chabanon AL, Chen A, Chicz RM, Diemert D, Essink BJ, Fu B, Grunenberg NA, Janosczyk H, Keefer MC, Rivera M DM, Meng Y, Michael NL, Munsiff SS, Ogbuagu O, Raabe VN, Severance R, Rivas E, Romanyak N, Rouphael NG, Schuerman L, Sher LD, Walsh SR, White J, von Barbier D, de Bruyn G, Canter R, Grillet MH, Keshtkar-Jahromi M, Koutsoukos M, Lopez D, Masotti R, Mendoza S, Moreau C, Ceregido MA, Ramirez S, Said A, Tavares-Da-Silva F, Shi J, Tong T, Treanor J, Diazgranados CA, Savarino S. Safety and immunogenicity of an AS03-adjuvanted SARS-CoV-2 recombinant protein vaccine (CoV2 preS dTM) in healthy adults: interim findings from a phase 2, randomised, dose-finding, multicentre study. Lancet Infect Dis. 2022 May;22(5):636-648. doi: 10.1016/S1473-3099(21)00764-7.
95. Somersan-Karakaya S, Mylonakis E, Menon VP, Wells JC, Ali S, Sivapalasingam S, Sun Y, Bhore R, Mei J, Miller J, Cupelli L, Forleo-Neto E, Hooper AT, Hamilton JD, Pan C, Pham V, Zhao Y, Hosain R, Mahmood A, Davis JD, Turner KC, Kim Y, Cook A, Kowal B, Soo Y, DiCioccio AT, Geba GP, Stahl N, Lipsich L, Braunstein N, Herman GA, Yancopoulos GD, Weinreich DM; COVID-19 Phase 2/3 Hospitalized Trial Team. Casirivimab and Imdevimab for the Treatment of Hospitalized Patients With COVID-19. J Infect Dis. 2022 Jul 27:jiac320. doi: 10.1093/infdis/jiac320.
96. Heath PT, Galiza EP, Baxter DN, Boffito M, Browne D, Burns F, Chadwick DR, Clark R, Cosgrove C, Galloway J, Goodman AL, Heer A, Higham A, Iyengar S, Jamal A, Jeanes C, Kalra PA, Kyriakidou C, McAuley DF, Meyrick A, Minassian AM, Minton J, Moore P, Munsoor I, Nicholls H, Osanlou O, Packham J, Pretswell CH, San Francisco Ramos A, Saralaya D, Sheridan RP, Smith R, Soiza RL, Swift PA, Thomson EC, Turner J, Viljoen ME, Albert G, Cho I, Dubovsky F, Glenn G, Rivers J, Robertson A, Smith K, Toback S; 2019nCoV-302 Study Group. Safety and Efficacy of NVX-CoV2373 Covid-19 Vaccine. N Engl J Med. 2021 Sep 23;385(13):1172-1183. doi: 10.1056/NEJMoa2107659.
97. Madhi SA, Baillie V, Cutland CL, Voysey M, Koen AL, Fairlie L, Padayachee SD, Dheda K, Barnabas SL, Bhorat QE, Briner C, Kwatra G, Ahmed K, Aley P, Bhikha S, Bhiman JN, Bhorat AE, du Plessis J, Esmail A, Groenewald M, Horne E, Hwa SH, Jose A, Lambe T, Laubscher M, Malahleha M, Masenya M, Masilela M, McKenzie S, Molapo K, Moultrie A, Oelofse S, Patel F, Pillay S, Rhead S, Rodel H, Rossouw L, Taoushanis C, Tegally H, Thombrayil A, van Eck S, Wibmer CK, Durham NM, Kelly EJ, Villafana TL, Gilbert S, Pollard AJ, de Oliveira T, Moore PL, Sigal A, Izu A; NGS-SA Group; Wits-VIDA COVID Group. Efficacy of the ChAdOx1 nCoV-19 Covid-19 Vaccine against the B.1.351 Variant. N Engl J Med. 2021 May 20;384(20):1885-1898. doi: 10.1056/NEJMoa2102214.
98. Shoham S, Bloch EM, Casadevall A, Hanley D, Lau B, Gebo K, Cachay E, Kassaye SG, Paxton JH, Gerber J, Levine AC, Naeim A, Currier J, Patel B, Allen ES, Anjan S, Appel L, Baksh S, Blair PW, Bowen A, Broderick P, Caputo CA, Cluzet V, Elena MC, Cruser D, Ehrhardt S, Forthal D, Fukuta Y, Gawad AL, Gniadek T, Hammel J, Huaman MA, Jabs DA, Jedlicka A, Karlen N, Klein S, Laeyendecker O, Karen L, McBee N, Meisenberg B, Merlo C, Mosnaim G, Park HS, Pekosz A, Petrini J, Rausch W, Shade DM, Shapiro JR, Singleton RJ, Sutcliffe C, Thomas DL, Yarava A, Zand M, Zenilman JM, Tobian AAR, Sullivan DJ. Transfusing convalescent plasma as post-exposure prophylaxis against SARS-CoV-2 infection: a double-blinded, phase 2 randomized, controlled trial. Clin Infect Dis. 2022 May 17:ciac372. doi: 10.1093/cid/ciac372.
99. Méndez-Flores S, Priego-Ranero Á, Azamar-Llamas D, Olvera-Prado H, Rivas-Redonda KI, Ochoa-Hein E, Perez-Ortiz A, Rendón-Macías ME, Rojas-Castañeda E, Urbina-Terán S, Septién-Stute L, Hernández-Gilsoul T, Aguilar-Morgan AA, Fernández-Camargo DA, Olivares-Martínez E, Hernández-Ramírez DF, Torres-Villalobos G, Furuzawa-Carballeda J. Effect of polymerised type I collagen on hyperinflammation of adult outpatients with symptomatic COVID-19. Clin Transl Med. 2022 Mar;12(3):e763. doi: 10.1002/ctm2.763.

© 2023 Bai AD et al. *JAMA Network Open*.

100. Körper S, Weiss M, Zickler D, Wiesmann T, Zacharowski K, Corman VM, Grüner B, Ernst L, Spieth P, Lepper PM, Bentz M, Zinn S, Paul G, Kalbhenn J, Dollinger MM, Rosenberger P, Kirschning T, Thiele T, Appl T, Mayer B, Schmidt M, Drosten C, Wulf H, Kruse JM, Jungwirth B, Seifried E, Schrezenmeier H; CAPSID Clinical Trial Group. Results of the CAPSID randomized trial for high-dose convalescent plasma in patients with severe COVID-19. J Clin Invest. 2021 Oct 15;131(20):e152264. doi: 10.1172/JCI152264.
101. Thomas SJ, Moreira ED Jr, Kitchin N, Absalon J, Gurtman A, Lockhart S, Perez JL, Pérez Marc G, Polack FP, Zerbini C, Bailey R, Swanson KA, Xu X, Roychoudhury S, Koury K, Bouguermouh S, Kalina WV, Cooper D, Frenck RW Jr, Hammitt LL, Türeci Ö, Nell H, Schaefer A, Ünal S, Yang Q, Liberator P, Tresnan DB, Mather S, Dormitzer PR, Şahin U, Gruber WC, Jansen KU; C4591001 Clinical Trial Group. Safety and Efficacy of the BNT162b2 mRNA Covid-19 Vaccine through 6 Months. N Engl J Med. 2021 Nov 4;385(19):1761-1773. doi: 10.1056/NEJMoa2110345.
102. Sivapalasingam S, Lederer DJ, Bhore R, Hajizadeh N, Criner G, Hosain R, Mahmood A, Giannelou A, Somersan-Karakaya S, O'Brien MP, Boyapati A, Parrino J, Musser BJ, Labriola-Tompkins E, Ramesh D, Purcell LA, Gulabani D, Kampman W, Waldron A, Gong MN, Saggar S, Sperber SJ, Menon V, Stein DK, Sobieszczyk ME, Park W, Aberg JA, Brown SM, Kosmicki JA, Horowitz JE, Ferreira MA, Baras A, Kowal B, DiCioccio AT, Akinlade B, Nivens MC, Braunstein N, Herman GA, Yancopoulos GD, Weinreich DM; Sarilumab-COVID-19 Study Team. Efficacy and Safety of Sarilumab in Hospitalized Patients With COVID-19: A Randomized Clinical Trial. Clin Infect Dis. 2022 Feb 26:ciac153. doi: 10.1093/cid/ciac153.
103. Hsieh SM, Liu MC, Chen YH, Lee WS, Hwang SJ, Cheng SH, Ko WC, Hwang KP, Wang NC, Lee YL, Lin YL, Shih SR, Huang CG, Liao CC, Liang JJ, Chang CS, Chen C, Lien CE, Tai IC, Lin TY. Safety and immunogenicity of CpG 1018 and aluminium hydroxide-adjuvanted SARS-CoV-2 S-2P protein vaccine MVC-COV1901: interim results of a large-scale, double-blind, randomised, placebo-controlled phase 2 trial in Taiwan. Lancet Respir Med. 2021 Dec;9(12):1396-1406. doi: 10.1016/S2213-2600(21)00402-1.
104. COVID STEROID 2 Trial Group, Munch MW, Myatra SN, Vijayaraghavan BKT, Saseedharan S, Benfield T, Wahlin RR, Rasmussen BS, Andreasen AS, Poulsen LM, Cioccari L, Khan MS, Kapadia F, Divatia JV, Brøchner AC, Bestle MH, Helleberg M, Michelsen J, Padmanaban A, Bose N, Møller A, Borawake K, Kristiansen KT, Shukla U, Chew MS, Dixit S, Ulrik CS, Amin PR, Chawla R, Wamberg CA, Shah MS, Darfelt IS, Jørgensen VL, Smitt M, Granholm A, Kjær MN, Møller MH, Meyhoff TS, Vesterlund GK, Hammond NE, Micallef S, Bassi A, John O, Jha A, Cronhjort M, Jakob SM, Gluud C, Lange T, Kadam V, Marcussen KV, Hollenberg J, Hedman A, Nielsen H, Schjørring OL, Jensen MQ, Leistner JW, Jonassen TB, Kristensen CM, Clapp EC, Hjortsø CJS, Jensen TS, Halstad LS, Bak ERB, Zaabalawi R, Metcalf-Clausen M, Abdi S, Hatley EV, Aksnes TS, Gleipner-Andersen E, Alarcón AF, Yamin G, Heymowski A, Berggren A, La Cour K, Weihe S, Pind AH, Engstrøm J, Jha V, Venkatesh B, Perner A. Effect of 12 mg vs 6 mg of Dexamethasone on the Number of Days Alive Without Life Support in Adults With COVID-19 and Severe Hypoxemia: The COVID STEROID 2 Randomized Trial. JAMA. 2021;326(18):1807-1817. doi: 10.1001/jama.2021.18295.
105. Dorward J, Yu LM, Hayward G, Saville BR, Gbinigie O, Van Hecke O, Ogburn E, Evans PH, Thomas NP, Patel MG, Richards D, Berry N, Detry MA, Saunders C, Fitzgerald M, Harris V, Shanyinde M, de Lusignan S, Andersson MI, Butler CC, Hobbs FR; PRINCIPLE Trial Collaborative Group. Colchicine for COVID-19 in the community (PRINCIPLE): a randomised, controlled, adaptive platform trial. Br J Gen Pract. 2022:BJGP.2022.0083. doi: 10.3399/BJGP.2022.0083.
106. Yu LM, Bafadhel M, Dorward J, Hayward G, Saville BR, Gbinigie O, Van Hecke O, Ogburn E, Evans PH, Thomas NPB, Patel MG, Richards D, Berry N, Detry MA, Saunders C, Fitzgerald M, Harris V, Shanyinde M, de Lusignan S, Andersson MI, Barnes PJ, Russell REK, Nicolau DV Jr, Ramakrishnan S, Hobbs FDR, Butler CC; PRINCIPLE Trial Collaborative Group. Inhaled budesonide for COVID-19 in people at high risk of complications in the community in the UK (PRINCIPLE): a randomised, controlled, open-label, adaptive platform trial. Lancet. 2021 Sep 4;398(10303):843-855. doi: 10.1016/S0140-6736(21)01744-X.

© 2023 Bai AD et al. *JAMA Network Open*.

107. REMAP-CAP Investigators, Gordon AC, Mouncey PR, Al-Beidh F, Rowan KM, Nichol AD, Arabi YM, Annane D, Beane A, van Bentum-Puijk W, Berry LR, Bhimani Z, Bonten MJM, Bradbury CA, Brunkhorst FM, Buzgau A, Cheng AC, Detry MA, Duffy EJ, Estcourt LJ, Fitzgerald M, Goossens H, Haniffa R, Higgins AM, Hills TE, Horvat CM, Lamontagne F, Lawler PR, Leavis HL, Linstrum KM, Litton E, Lorenzi E, Marshall JC, Mayr FB, McAuley DF, McGlothlin A, McGuinness SP, McVerry BJ, Montgomery SK, Morpeth SC, Murthy S, Orr K, Parke RL, Parker JC, Patanwala AE, Pettilä V, Rademaker E, Santos MS, Saunders CT, Seymour CW, Shankar-Hari M, Sligl WI, Turgeon AF, Turner AM, van de Veerdonk FL, Zarychanski R, Green C, Lewis RJ, Angus DC, McArthur CJ, Berry S, Webb SA, Derde LPG. Interleukin-6 Receptor Antagonists in Critically Ill Patients with Covid-19. N Engl J Med. 2021 Apr 22;384(16):1491-1502. doi: 10.1056/NEJMoa2100433.
108. Writing Committee for the REMAP-CAP Investigators, Estcourt LJ, Turgeon AF, McQuilten ZK, McVerry BJ, Al-Beidh F, Annane D, Arabi YM, Arnold DM, Beane A, Bégin P, van Bentum-Puijk W, Berry LR, Bhimani Z, Birchall JE, Bonten MJM, Bradbury CA, Brunkhorst FM, Buxton M, Callum JL, Chassé M, Cheng AC, Cove ME, Daly J, Derde L, Detry MA, De Jong M, Evans A, Fergusson DA, Fish M, Fitzgerald M, Foley C, Goossens H, Gordon AC, Gosbell IB, Green C, Haniffa R, Harvala H, Higgins AM, Hills TE, Hoad VC, Horvat C, Huang DT, Hudson CL, Ichihara N, Laing E, Lamikanra AA, Lamontagne F, Lawler PR, Linstrum K, Litton E, Lorenzi E, MacLennan S, Marshall J, McAuley DF, McDyer JF, McGlothlin A, McGuinness S, Miflin G, Montgomery S, Mouncey PR, Murthy S, Nichol A, Parke R, Parker JC, Priddee N, Purcell DFJ, Reyes LF, Richardson P, Robitaille N, Rowan KM, Rynne J, Saito H, Santos M, Saunders CT, Serpa Neto A, Seymour CW, Silversides JA, Tinmouth AA, Triulzi DJ, Turner AM, van de Veerdonk F, Walsh TS, Wood EM, Berry S, Lewis RJ, Menon DK, McArthur C, Zarychanski R, Angus DC, Webb SA, Roberts DJ, Shankar-Hari M. Effect of Convalescent Plasma on Organ Support-Free Days in Critically Ill Patients With COVID-19: A Randomized Clinical Trial. JAMA. 2021 Nov 2;326(17):1690-1702. doi: 10.1001/jama.2021.18178.
109. Nguyen TP, Do Q, Phan LT, Dinh DV, Khong H, Hoang LV, Nguyen TV, Pham HN, Chu MV, Nguyen TT, Pham QD, Le TM, Trang TNT, Dinh TT, Vo TV, Vu TT, Nguyen QBP, Phan VT, Nguyen LV, Nguyen GT, Tran PM, Nghiem TD, Tran TV, Nguyen TG, Tran TQ, Nguyen LT, Do AT, Nguyen DD, Ho SA, Nguyen VT, Pham DT, Tran HB, Vu ST, Hoang SX, Do TM, Nguyen XT, Le GQ, Tran T, Cao TM, Dao HM, Nguyen TTT, Doan UY, Le VTT, Tran LP, Nguyen NM, Nguyen NT, Pham HTT, Nguyen QH, Nguyen HT, Nguyen HLK, Tran VT, Tran MTN, Nguyen TTT, Ha PT, Huynh HT, Nguyen KD, Thuan UT, Doan CC, Do SM. Safety and immunogenicity of Nanocovax, a SARS-CoV-2 recombinant spike protein vaccine: Interim results of a double-blind, randomised controlled phase 1 and 2 trial. Lancet Reg Health West Pac. 2022 May 16;24:100474. doi: 10.1016/j.lanwpc.2022.100474.
110. Hinks TSC, Cureton L, Knight R, Wang A, Cane JL, Barber VS, Black J, Dutton SJ, Melhorn J, Jabeen M, Moss P, Garlapati R, Baron T, Johnson G, Cantle F, Clarke D, Elkhodair S, Underwood J, Lasserson D, Pavord ID, Morgan S, Richards D. Azithromycin versus standard care in patients with mild-to-moderate COVID-19 (ATOMIC2): an open-label, randomised trial. Lancet Respir Med. 2021 Oct;9(10):1130-1140. doi: 10.1016/S2213-2600(21)00263-0.
111. Reis G, dos Santos Moreira-Silva EA, Silva DC, Thabane L, Milagres AC, Ferreira TS, Dos Santos CV, de Souza Campos VH, Nogueira AM, de Almeida AP, Callegari ED. Effect of early treatment with fluvoxamine on risk of emergency care and hospitalisation among patients with COVID-19: the TOGETHER randomised, platform clinical trial. The Lancet Global Health. 2022 Jan 1;10(1):e42-51.
112. Quinn TM, Gaughan EE, Bruce A, Antonelli J, O'Connor R, Li F, McNamara S, Koch O, MacKintosh C, Dockrell D, Walsh T, Blyth KG, Church C, Schwarze J, Boz C, Valanciute A, Burgess M, Emanuel P, Mills B, Rinaldi G, Hardisty G, Mills R, Findlay EG, Jabbal S, Duncan A, Plant S, Marshall ADL, Young I, Russell K, Scholefield E, Nimmo AF, Nazarov IB, Churchill GC, McCullagh JSO, Ebrahimi KH, Ferrett C, Templeton K, Rannard S, Owen A, Moore A, Finlayson K, Shankar-Hari M, Norrie J, Parker RA, Akram AR, Anthony DC, Dear JW, Hirani N, Dhaliwal K. Randomised

© 2023 Bai AD et al. *JAMA Network Open*.

controlled trial of intravenous nafamostat mesylate in COVID pneumonitis: Phase 1b/2a experimental study to investigate safety, Pharmacokinetics and Pharmacodynamics. EBioMedicine. 2022 Feb;76:103856. doi: 10.1016/j.ebiom.2022.103856.
113. Marconi VC, Ramanan AV, de Bono S, Kartman CE, Krishnan V, Liao R, Piruzeli MLB, Goldman JD, Alatorre-Alexander J, de Cassia Pellegrini R, Estrada V, Som M, Cardoso A, Chakladar S, Crowe B, Reis P, Zhang X, Adams DH, Ely EW; COV-BARRIER Study Group. Efficacy and safety of baricitinib for the treatment of hospitalised adults with COVID-19 (COV-BARRIER): a randomised, double-blind, parallel-group, placebo-controlled phase 3 trial. Lancet Respir Med. 2021;9(12):1407-1418. doi: 10.1016/S2213-2600(21)00331-3.
114. Shinde V, Bhikha S, Hoosain Z, Archary M, Bhorat Q, Fairlie L, Lalloo U, Masilela MSL, Moodley D, Hanley S, Fouche L, Louw C, Tameris M, Singh N, Goga A, Dheda K, Grobbelaar C, Kruger G, Carrim-Ganey N, Baillie V, de Oliveira T, Lombard Koen A, Lombaard JJ, Mngqibisa R, Bhorat AE, Benadé G, Lalloo N, Pitsi A, Vollgraaff PL, Luabeya A, Esmail A, Petrick FG, Oommen-Jose A, Foulkes S, Ahmed K, Thombrayil A, Fries L, Cloney-Clark S, Zhu M, Bennett C, Albert G, Faust E, Plested JS, Robertson A, Neal S, Cho I, Glenn GM, Dubovsky F, Madhi SA; 2019nCoV-501 Study Group. Efficacy of NVX-CoV2373 Covid-19 Vaccine against the B.1.351 Variant. N Engl J Med. 2021;384(20):1899-1909. doi: 10.1056/NEJMoa2103055.
115. Trieu V, Saund S, Rahate P, Barge V, Nalk S, Windlass H, Uckun F. Targeting TGF-β pathway with COVID-19 Drug Candidate ARTIVeda/PulmoHeal Accelerates Recovery from Mild-Moderate COVID-19. Clin Invest (Lond). 2021;11(1):10-18.
116. Fischer WA 2nd, Eron JJ Jr, Holman W, Cohen MS, Fang L, Szewczyk LJ, Sheahan TP, Baric R, Mollan KR, Wolfe CR, Duke ER, Azizad MM, Borroto-Esoda K, Wohl DA, Coombs RW, James Loftis A, Alabanza P, Lipansky F, Painter WP. A phase 2a clinical trial of molnupiravir in patients with COVID-19 shows accelerated SARS-CoV-2 RNA clearance and elimination of infectious virus. Sci Transl Med. 2022 Jan 19;14(628):eabl7430. doi: 10.1126/scitranslmed.abl7430.
117. Feng Y, Chen J, Yao T, Chang Y, Li X, Xing R, Li H, Xie R, Zhang X, Wei Z, Mu S, Liu L, Feng L, Wang S. Safety and immunogenicity of inactivated SARS-CoV-2 vaccine in high-risk occupational population: a randomized, parallel, controlled clinical trial. Infect Dis Poverty. 2021;10(1):138. doi: 10.1186/s40249-021-00924-2.
118. Temesgen Z, Burger CD, Baker J, Polk C, Libertin CR, Kelley CF, Marconi VC, Orenstein R, Catterson VM, Aronstein WS, Durrant C, Chappell D, Ahmed O, Chappell G, Badley AD; LIVE-AIR Study Group. Lenzilumab in hospitalised patients with COVID-19 pneumonia (LIVE-AIR): a phase 3, randomised, placebo-controlled trial. Lancet Respir Med. 2022;10(3):237-246. doi: 10.1016/S2213-2600(21)00494-X.
119. Zeng G, Wu Q, Pan H, Li M, Yang J, Wang L, Wu Z, Jiang D, Deng X, Chu K, Zheng W, Wang L, Lu W, Han B, Zhao Y, Zhu F, Yu H, Yin W. Immunogenicity and safety of a third dose of CoronaVac, and immune persistence of a two-dose schedule, in healthy adults: interim results from two single-centre, double-blind, randomised, placebo-controlled phase 2 clinical trials. Lancet Infect Dis. 2022 Apr;22(4):483-495. doi: 10.1016/S1473-3099(21)00681-2.

© 2023 Bai AD et al. *JAMA Network Open*.

eTable 5. Differences Between Preprints and Corresponding Journal Articles for Each Journal Impact Factor Quartile

	1st quartile IF 0 to 5.9 (N=30)	2nd quartile IF 5.0 to 19.1 (N=30)	3rd quartile IF 19.2 to 102.6 (N=34)	4th quartile IF 102.7 or higher (N=25)	P-value
Significant differences in					
Primary outcome	5 (16.7%)	0 (0%)	3 (8.8%)	0 (0%)	>0.99
Secondary outcome	11 (36.7%)	2 (6.7%)	7 (20.6%)	8 (32.0%)	0.52
Primary analysis for primary outcome	7 (23.3%)	3 (10.0%)	3 (8.8%)	3 (12.0%)	0.79
Secondary analyses for primary outcome	6 (20.0%)	6 (20.0%)	6 (17.7%)	8 (32.0%)	0.18
Subgroups analyzed	2 (6.7%)	0 (0%)	11 (32.3%)	7 (28.0%)	<0.001
Sample size	1 (3.3%)	0 (0%)	3 (8.8%)	3 (12.0%)	0.12
Result in terms of primary outcome	9 (30.0%)	7 (23.3%)	6 (17.7%)	11 (44.0%)	0.41
Study conclusion	1 (3.3%)	1 (3.3%)	0 (0%)	0 (0%)	0.51
Same in all of the aspects above	11 (36.7%)	21 (70.0%)	15 (44.1%)	7 (28.0%)	0.009

IF = impact factor

© 2023 Bai AD et al. *JAMA Network Open*.

eFigure. Time to Publication Based on Date of Preprint Posting

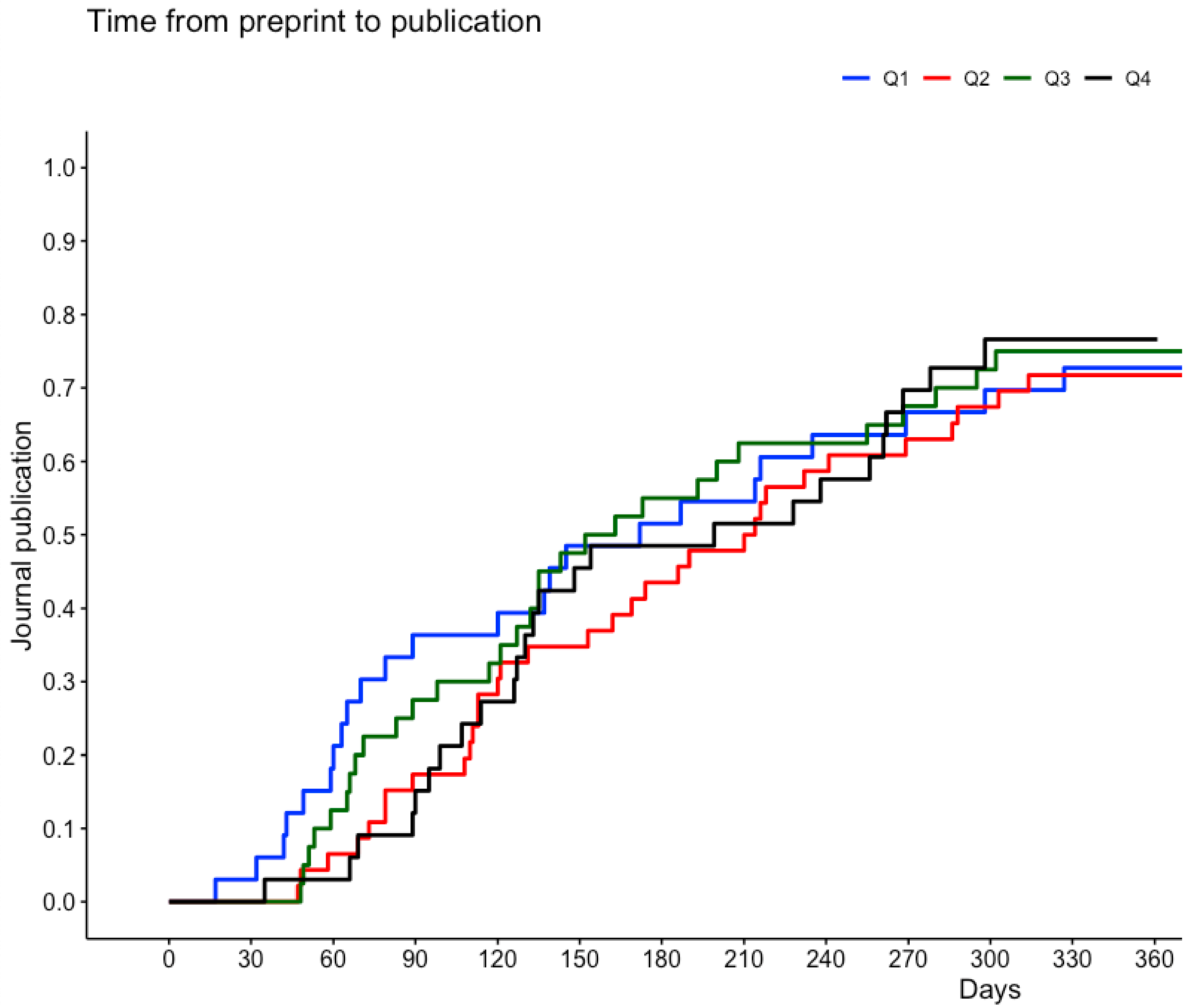

Q1 = Preprints posted during time period from January 1, 2021 to March 31, 2021
Q2 = Preprints posted during time period from April 1, 2021 to June 30, 2021
Q3 = Preprints posted during time period from July 1, 2021 to September 30, 2021
Q4 = Preprints posted during time period from October 1, 2021 to December 31, 2021

Log-rank test using generalization of the chi-square test for all 4 quarters (Q1, Q2, Q3 and Q4) had a P-value of 0.80

© 2023 Bai AD et al. *JAMA Network Open*.
