# Supplementary material for: Comparison of Preprint Postings of Randomized Clinical Trials on COVID-19 and Corresponding Published Journal Articles: A Systematic Review
Source: JAMA Netw Open. 2023 Jan 27;6(1):e2253301. doi: 10.1001/jamanetworkopen.2022.53301 (PMC12543410; doi:10.1001/jamanetworkopen.2022.53301)
Supplement: Supplement 2. — Data Sharing Statement [file jamanetwopen-e2253301-s002.pdf]

Data Sharing Statement

Bai. Comparison of Preprint Postings of Randomized Clinical Trials on COVID-19 and Corresponding Published Journal Articles. *JAMA Netw Open*. Published January 27, 2023. doi:10.1001/jamanetworkopen.2022.53301

Data
Data available: Yes
Data types: Data (not involving human participants)
How to access data: All available data analyzed are available in the supplementary materials
When available: With publication

Supporting Documents
Document types: None

Additional Information
Who can access the data: Anyone requesting the data
Types of analyses: For any purpose
Mechanisms of data availability: With investigator support
